# Global burden of heart failure attributable to atrial fibrillation and flutter, insights from GBD 2021

**DOI:** 10.1093/eschf/xvag094

**Published:** 2026-03-30

**Authors:** Junpeng Xiong, Shiyang Guan, Cheng Cheng, Yingying Zhang, Shuwen Chen, Jinfeng Wang, Xinlin Yang, Huili Wang, Faming Pan, Ronghui Yu

**Affiliations:** Department of Cardiology, National Cardiovascular Disease Regional Center for Anhui, The First Affiliated Hospital of Anhui Medical University, No.120, Wanshui Road, Shushan District, Hefei, Anhui 230088, China; Department of Epidemiology and Biostatistics, School of Public Health, Anhui Medical University, No.81, Meishan Road, Shushan District, Hefei, Anhui 230032, China; Department of Cardiology, National Cardiovascular Disease Regional Center for Anhui, The First Affiliated Hospital of Anhui Medical University, No.120, Wanshui Road, Shushan District, Hefei, Anhui 230088, China; Department of Cardiology, National Cardiovascular Disease Regional Center for Anhui, The First Affiliated Hospital of Anhui Medical University, No.120, Wanshui Road, Shushan District, Hefei, Anhui 230088, China; Department of Cardiology, National Cardiovascular Disease Regional Center for Anhui, The First Affiliated Hospital of Anhui Medical University, No.120, Wanshui Road, Shushan District, Hefei, Anhui 230088, China; Department of Cardiology, National Cardiovascular Disease Regional Center for Anhui, The First Affiliated Hospital of Anhui Medical University, No.120, Wanshui Road, Shushan District, Hefei, Anhui 230088, China; Department of Cardiology, National Cardiovascular Disease Regional Center for Anhui, The First Affiliated Hospital of Anhui Medical University, No.120, Wanshui Road, Shushan District, Hefei, Anhui 230088, China; Department of Cardiology, Lvliang First People's Hospital, Lvliang Hospital of Shanxi Medical University, Lvliang, Shanxi 033000, China; Department of Epidemiology and Biostatistics, School of Public Health, Anhui Medical University, No.81, Meishan Road, Shushan District, Hefei, Anhui 230032, China; Department of Cardiology, National Cardiovascular Disease Regional Center for Anhui, The First Affiliated Hospital of Anhui Medical University, No.120, Wanshui Road, Shushan District, Hefei, Anhui 230088, China

**Keywords:** Atrial fibrillation/flutter, Heart failure, Global burden of disease, Prevalence, Years lived with disability

## Abstract

**Aims:**

The aim of this study was to systematically quantify the global, regional, and national burden of heart failure (HF) attributable to atrial fibrillation/flutter (AF/AFL) from 1990 to 2021, and project trends to 2040.

**Methods and Results:**

Using Global Burden of Disease (GBD) 2021 data, we analysed AF/AFL-attributed HF prevalence and years lived with disability (YLDs) across 204 countries, stratified by age, sex, and socio-demographic index (SDI). Joinpoint regression identified temporal inflection points; decomposition analysis quantified contributions from population growth, ageing, and epidemiological change; Bayesian age-period-cohort (BAPC) models forecasted burden to 2040. Globally, AF-attributed HF cases increased 3.4-fold, from 162 561 (95% UI: 120 008–213 951) in 1990 to 714 137 (95% UI: 520 543–940 901) in 2021, with age-standardized prevalence rates (ASPRs) rising from 5.36 to 8.85 per 100 000 (EAPC: 1.76%, 95% UI: 1.66–1.85%). YLDs increased more than four-fold, from 14 615 (95% UI: 8848–23 114) to 63 943 (95% UI: 39 058–96 196; EAPC: 1.76%, 95% CI: 1.67–1.85%). High-SDI regions exhibited the highest burden (ASPR: 13.97 (95% UI: 10.31 to 18.36) per 100 000), with epidemiological changes contributing 44% of the absolute increase in high-SDI regions to case growth. Women outnumbered men beyond age 65 (female-to-male ratio: 1.1 at age 65–69, widening to 2.4 at ≥95 years), yet age-standardized rates showed no significant sex difference (female ASPR: 8.91 vs. male ASPR: 8.78 per 100 000). Joinpoint analysis revealed accelerated growth during 1990–2007 (APC: 2.12%, *P* < .05), which decelerated during 2007–2018 (APC: 1.41%, *P* < .05) and plateaued during 2018–2021 (APC: −0.36%, *P* > .05). Projections indicate a near-doubling of global cases to 1 307 469 (95% UI: 661 547–1 953 391) by 2040, corresponding to an ASPR of 8.45 per 100 000 (95% UI: 4.28–12.63).

**Conclusion:**

AF/AFL is a major and growing driver of global HF burden, projected to affect 1.3 million individuals by 2040, with disproportionate impact in older adults (≥65 years) and high-SDI regions. Integrating standardized AF management—including systematic screening, anticoagulation, and early rhythm control—into HF prevention pathways represents a critical strategy to mitigate projected increases.

## Graphical Abstract

**Graphical Abstract:**
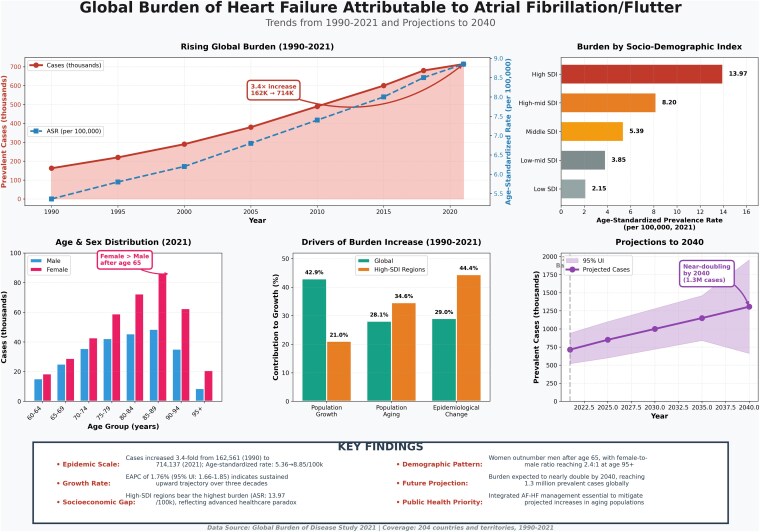

## Introduction

Atrial fibrillation (AF) and heart failure (HF) are among the most common and burdensome cardiovascular syndromes today. Their prevalence continues to expand against the backdrop of an ageing global population and increased exposure to metabolic risks.^1,2^ According to the latest data from the Global Burden of Disease Study (GBD 2021), the number of people with AF/flutter (AF/AFL) worldwide exceeded 59 million in 2023,^3^ and its incidence and disability burden are on the rise in most regions. Correspondingly, the number of people with HF worldwide in 2021 was approximately 55 million,^4^ and it has become a long-term source of pressure on the medical and socioeconomic systems of various countries.

There is a clear bidirectional pathophysiological relationship between AF and HF: AF can trigger or aggravate HF through mechanisms such as tachycardia-induced cardiomyopathy,^5,6^ loss of atrial pump function and restricted ventricular filling,^7^ and neurohumoral activation;^8^ while HF contributes to the occurrence and maintenance of AF due to increased atrial pressure and volume load,^9^ myocardial fibrosis and structural/electrical remodelling,^10^ and mitral regurgitation.^11^ Clinically, the coexistence of the two diseases not only significantly increases the risk of hospitalization and readmission, but is also associated with a higher all-cause mortality,^12^ suggesting that effective intervention in any link may achieve ‘spillover benefits’ in the other link.

From a macro-trend perspective, the global burden of HF increased overall between 1990 and 2021, with the increase being particularly significant in the elderly population. In terms of aetiology, ischaemic heart disease and hypertensive heart disease have long dominated, but the proportion of HF contributed by ‘age-related’ causes, including chronic kidney disease and AF/AFL, and YLDs are on the rise, suggesting that changes in the arrhythmia spectrum and the coexistence of multiple diseases are becoming important drivers of HF epidemiology.^4^ In addition, there are persistent and increasing differences in the burden of HF morbidity and disability in regions with different sociodemographic development indices (SDIs). Some evidence shows that causes such as AF/AFL are more concentrated in high-SDI regions,^13^ suggesting that the spectrum of arrhythmic HF in resource-rich regions needs to be managed in a targeted manner. Age-specific analysis suggests that the leading causes of HF in adolescents and young adults (10–24 years) are cardiomyopathy/myocarditis, congenital heart defects, and rheumatic heart disease,^14^ with AF/AFL accounting for a very low proportion. However, the relative importance of AF/AFL-related HF increases with age.^13^ This is consistent with the clinical epidemiological pattern that the prevalence of AF increases significantly with age, further highlighting the need for a specific assessment of the burden of HF caused by AF/AFL in the elderly.

Although previous GBD studies have comprehensively quantified the overall burden of HF and reported its aetiology, systematic studies on the global, regional, and national distribution, spatiotemporal evolution, age/sex, and SDI differences of ‘HF caused by AF/AFL,’ and the decomposition of the three factors of population growth, age structure, and rate change are still relatively insufficient. This has limited the evidence-based support and optimization of health resource allocation for incorporating ‘atrial fibrillation management and heart failure prevention’ into an integrated prevention and control pathway. Relying on the GBD 2021 database, this article aims to systematically quantify the burden trend of HF attributable to AF/AFL (Prevalence, YLDs) from 1990 to 2021 at the global, regional and national levels, and conduct stratified comparisons by SDI, gender and age, to provide actionable demographic basis and policy inspiration for AF management and HF prevention in different health resource scenarios.

## Methods

### Data sources and study design

This study is a secondary analysis based on the Global Burden of Disease, Injuries, and Risk Factors Study 2021 (GBD 2021) (https://vizhub.healthdata.org/gbd-results/). The GBD 2021 cause-of-death analysis estimated mortality rates and years of life lost (YLLs) for 288 causes of death from 1990 to 2021, stratified by age, sex, location, and year across 204 countries and territories, as well as 811 subnational locations. This analysis incorporated 56 604 data sources, including vital registration, verbal autopsy, surveys, censuses, surveillance systems, and cancer registries. As in previous GBD iterations, cause-specific mortality for most causes was modelled using the Cause of Death Ensemble model (CODEm), spatiotemporal Gaussian process regression, and the Bayesian meta-regression modelling tool (DisMod-MR 2.1). These approaches evaluated out-of-sample predictive validity across various statistical models and covariate combinations, combining results to generate cause-specific mortality estimates—along with alternative strategies for causes with limited data, significant changes reported during the study period, or epidemiological anomalies.^15^ The GBD utilizes de-identified aggregate data and has been approved for exemption from informed consent by the University of Washington Institutional Review Board; as a secondary analysis of publicly available data, this study requires no additional ethical approval or participant consent. This study adheres to the Guidelines for Accurate and Transparent Health Estimates Reporting (GATHER) to ensure transparency and reliability of results, with the GATHER checklist provided in Supplementary Table S1.

### Case definitions and severity stratification

The GBD models HF as a primary impairment, incorporating literature and surveillance data on HF diagnosed according to standardized clinical criteria (typically including Framingham and ESC standards). Since 2016, it has adopted the universal definition of HF at stage C and above to encompass both symptomatic cases and those with prior diagnoses but currently asymptomatic. HF severity is categorized into four levels based on clinical symptoms—‘treated, mild, moderate, severe’—for estimating prevalence and years lived with disability (YLDs).

In the HF attribution analysis, the GBD includes a predefined set of the most granular (Level 3/4) ‘underlying causes’ to ensure mutually exclusive and collectively exhaustive causal categories, explicitly incorporating ‘atrial fibrillation and flutter’ (AF/AFL). AF and flutter are defined as electrocardiographic findings showing irregular rhythm without P waves. According to the International Classification of Diseases, Ninth and Tenth Revisions, the codes for AF/AFL are as follows: ICD-9: 427.3; ICD-10: I48-I48.9.

### Key indicators

In this study, from the overall HF burden, we extracted the prevalence, YLDs, and their age-standardized rates (ASRs) with uncertainty intervals (UIs) attributable to AF/AFL, presented by sex, age group, geographic level, and socio-demographic index (SDI). Years lived with disability (YLDs) are calculated by multiplying the cause-age-sex-location-year-specific prevalence of each sequela from diseases and injuries by their respective disability weights. The socio-demographic index (SDI) is a composite indicator measuring socioeconomic development levels, correlated with health outcomes, ranging from 0 (lowest development) to 1 (highest development). Based on 2021 SDI quintiles, the 204 countries and territories were divided into five groups: low SDI (<0.466), low-middle SDI (≥0.466 and <0.619), middle SDI (≥0.619 and <0.720), high-middle SDI (≥0.720 and <0.810), and high SDI (≥0.810).^16^

### Statistical analysis

We employed joinpoint regression analysis to identify significant changes in temporal trends and compute the average annual percentage change (AAPC). Additionally, we calculated the estimated annual percentage change (EAPC) to quantify the average annual change in age-standardized rates (ASRs). To facilitate comparisons across populations with differing age structures, we computed ASRs using the GBD standard population. The applied age-standardization formula is as follows: $Age-StandardizedRate=\overset{n}{\underset{i=1}{\sum }}\;(Age-SpecificRate_{i}\times PopulationWeight)$. The EAPC quantifies the average annual change in ASR over a specified period and is calculated using the formula: EAPC=100×(exp(β)−1). where β is the slope coefficient from the linear regression model applied to the natural logarithm of the age-standardized rate. The annual percentage change (APC) reflects slope changes within each time segment, while the AAPC is derived by weighting segment lengths, capturing the overall trend across the entire period. If the 95% confidence interval (CI) for APC or AAPC includes 0, the trend is considered stable. A *P*-value <.05 indicates a significant upward or downward trend.

To examine socioeconomic disparities, we analysed the relationship between disease burden and the socio-demographic index (SDI), a composite measure of per capita income, educational attainment, and fertility rates. Furthermore, we conducted decomposition analysis to delineate the relative contributions of population ageing, population growth, and epidemiological changes to the observed trends in HF burden, explaining changes from 1990 to 2021.

We utilized Bayesian age-period-cohort (BAPC) analysis, as proposed by Riebler and Held,^17^ which simultaneously accounts for age, period, and cohort effects influencing disease burden trajectories. The BAPC model employs second-order random walk specifications for time-varying parameters, incorporating demographic shifts and epidemiological transitions. Projections to 2040 were generated under the assumption that current population trends (e.g. growth and ageing) and healthcare environments (e.g. diagnostic and treatment capacities) remain stable during the forecast period. Model fitting used an integrated nested Laplace approximation, with selection based on the deviance information criterion. Prediction intervals derive from the posterior distribution of model parameters, representing the 2.5th and 97.5th percentiles. This forecasted the future burden from 2022 to 2040. Population estimates were drawn from the 2019 revision of the United Nations World Population Prospects, stratified by year (up to 2100), age, and sex (https://population.un.org/wpp/downloads).

All statistical analyses were performed using R software (version 4.4.3), with significance set at *P* < .05. Uncertainty intervals (UIs) were computed at a 95% confidence level to account for estimate variability. This study adheres to the GBD methodological framework, ensuring consistency and comparability with existing GBD reports.

## Results

### Global and regional trends in AF-attributed heart failure burden

Between 1990 and 2021, both the global number of HF cases attributable to AF and the age-standardized prevalence rate (ASPR) showed a sustained upward trend. The global case number increased from approximately 162 561 cases (95% UI: 120 008–213 951) in 1990 to 714 137 cases (95% UI: 520 543–940 901) in 2021, representing a 3.4-fold increase. Meanwhile, the ASPR rose from 5.36 per 100 000 (95% UI: 3.88–7.05) to 8.85 per 100 000 (95% UI: 6.38–11.63), with an estimated annual percentage change (EAPC) of 1.76% (95% CI: 1.66–1.85). Similarly, years lived with disability (YLDs) number of cases increased more than four-fold, from 14 615 (95% UI: 8848–23 114) to 63 943 (95% UI: 39 058–96 196), with ASYR rising from 0.48 (95% UI: 0.30–0.74) to 0.79 (95% UI: 0.49–1.19) (EAPC: 1.76%, 95% CI: 1.67–1.85) (*Table 1*).

**Table 1:** Global and region of heart failure attributable to atrial fibrillation and flutter from 1990 to 2021: prevalence, years lived with disability (YLDs), age-standardized rates, and estimated annual percentage changes (EAPC)

Location	Prevalence					YLDs(years lived with disability rates)				
	Number of cases-1990	ASPR-1990	Number of cases-2021	ASPR-2021	EAPC (95%CI)	Number of cases-1990	ASYR-1990	Number of cases-2021	ASYR-2021	EAPC (95%CI)
Global	162561 (120008 to 213951)	5.36 (3.88 to 7.05)	714137 (520543 to 940901)	8.85 (6.38 to 11.63)	1.76 (1.66 to 1.85)	14615 (8848 to 23114)	0.48 (0.3 to 0.74)	63943 (39058 to 96196)	0.79 (0.49 to 1.19)	1.76 (1.67 to 1.85)
High SDI	80490 (57362 to 107939)	7.29 (5.27 to 9.67)	343217 (249216 to 452402)	13.97 (10.31 to 18.36)	2.39 (2.19 to 2.59)	7260 (4353 to 11551)	0.66 (0.4 to 1.04)	30856 (18841 to 46262)	1.26 (0.76 to 1.91)	2.39 (2.19 to 2.59)
High-middle SDI	32289 (23877 to 42991)	4.14 (3.04 to 5.49)	140138 (102365 to 185308)	7.24 (5.27 to 9.57)	2 (1.92 to 2.08)	2909 (1754 to 4570)	0.37 (0.23 to 0.57)	12562 (7527 to 19534)	0.65 (0.39 to 0.99)	2 (1.92 to 2.08)
Middle SDI	30247 (23494 to 38992)	4.92 (3.7 to 6.45)	155902 (116416 to 202196)	7.18 (5.28 to 9.31)	1.14 (1.09 to 1.2)	2705 (1701 to 4151)	0.43 (0.27 to 0.65)	13877 (8552 to 21104)	0.64 (0.39 to 0.97)	1.16 (1.1 to 1.21)
Low-middle SDI	13343 (10149 to 17411)	3.75 (2.78 to 4.93)	54797 (39832 to 71596)	5.39 (3.82 to 7.19)	1.09 (1.05 to 1.14)	1187 (743 to 1846)	0.33 (0.21 to 0.5)	4861 (2946 to 7545)	0.47 (0.29 to 0.72)	1.1 (1.06 to 1.15)
Low SDI	5979 (4093 to 8546)	4.93 (3.29 to 6.67)	19377 (13270 to 27678)	6.27 (4.21 to 8.63)	0.82 (0.72 to 0.92)	534 (309 to 894)	0.43 (0.25 to 0.7)	1723 (1024 to 2836)	0.55 (0.32 to 0.88)	0.83 (0.73 to 0.93)
High-income Asia Pacific	7724 (5169 to 10373)	4.34 (2.91 to 5.77)	49181 (34366 to 64950)	8.67 (6.33 to 11.24)	1.87 (1.55 to 2.18)	704 (393 to 1118)	0.39 (0.23 to 0.61)	4419 (2599 to 6728)	0.78 (0.48 to 1.19)	1.86 (1.55 to 2.18)
High-income North America	24730 (16907 to 34279)	6.65 (4.58 to 9.04)	92452 (65516 to 126087)	12.79 (9.1 to 17.54)	2.31 (2.13 to 2.49)	2233 (1281 to 3579)	0.6 (0.35 to 0.95)	8334 (4921 to 12788)	1.15 (0.68 to 1.79)	2.3 (2.12 to 2.47)
Western Europe	47250 (33998 to 63448)	7.89 (5.72 to 10.46)	195489 (140040 to 260363)	16.9 (12.28 to 22.38)	2.97 (2.71 to 3.23)	4257 (2582 to 6624)	0.71 (0.43 to 1.1)	17574 (10645 to 26744)	1.52 (0.93 to 2.32)	2.98 (2.72 to 3.24)
Australasia	2676 (1905 to 3694)	11.94 (8.45 to 16.46)	15468 (11242 to 20978)	25.24 (18.51 to 34.05)	2.8 (2.42 to 3.18)	241 (144 to 368)	1.07 (0.64 to 1.61)	1385 (812 to 2059)	2.26 (1.32 to 3.38)	2.8 (2.42 to 3.18)
Andean Latin America	1546 (1174 to 1985)	9.15 (6.88 to 11.82)	6748 (5006 to 8880)	12.14 (8.97 to 16.02)	1 (0.93 to 1.07)	138 (83 to 210)	0.81 (0.48 to 1.24)	603 (367 to 874)	1.08 (0.66 to 1.57)	1.03 (0.96 to 1.1)
Tropical Latin America	3503 (2615 to 4572)	5.94 (4.33 to 7.82)	25946 (18019 to 35262)	10.85 (7.45 to 14.82)	1.86 (1.79 to 1.93)	311 (192 to 479)	0.52 (0.33 to 0.81)	2300 (1375 to 3580)	0.96 (0.57 to 1.49)	1.87 (1.79 to 1.94)
Central Latin America	4859 (3787 to 6162)	7.61 (5.81 to 9.68)	23296 (17811 to 30234)	10.06 (7.67 to 13.08)	0.8 (0.73 to 0.86)	433 (271 to 652)	0.67 (0.43 to 1.01)	2079 (1287 to 3135)	0.9 (0.55 to 1.34)	0.81 (0.75 to 0.88)
Southern Latin America	1213 (850 to 1628)	3.1 (2.18 to 4.23)	4973 (3253 to 6884)	5.39 (3.55 to 7.44)	2.29 (2 to 2.58)	110 (62 to 173)	0.28 (0.16 to 0.44)	447 (250 to 713)	0.49 (0.27 to 0.77)	2.3 (2.01 to 2.58)
Caribbean	1606 (1215 to 2131)	7.3 (5.57 to 9.59)	5200 (3763 to 6951)	9.45 (6.84 to 12.69)	0.74 (0.59 to 0.89)	143 (86 to 222)	0.65 (0.41 to 1)	462 (279 to 690)	0.84 (0.51 to 1.27)	0.75 (0.59 to 0.9)
Central Europe	6278 (4615 to 8407)	4.94 (3.67 to 6.57)	18657 (13907 to 25103)	7.64 (5.72 to 10.19)	2.13 (1.75 to 2.51)	566 (341 to 893)	0.44 (0.27 to 0.7)	1677 (1010 to 2627)	0.69 (0.42 to 1.07)	2.13 (1.75 to 2.51)
Eastern Europe	8265 (5968 to 11358)	3.45 (2.5 to 4.72)	17275 (11587 to 24057)	4.72 (3.17 to 6.59)	1.39 (1.06 to 1.73)	744 (437 to 1188)	0.31 (0.18 to 0.48)	1547 (881 to 2555)	0.42 (0.24 to 0.69)	1.38 (1.05 to 1.72)
Central Asia	605 (419 to 852)	1.55 (1.07 to 2.19)	1181 (775 to 1640)	1.86 (1.2 to 2.64)	0.82 (0.41 to 1.23)	55 (32 to 85)	0.14 (0.08 to 0.22)	108 (59 to 173)	0.17 (0.09 to 0.27)	0.82 (0.42 to 1.23)
North Africa and Middle East	2880 (2279 to 3570)	2.55 (1.99 to 3.29)	13076 (10166 to 16510)	3.89 (2.93 to 5.01)	1.41 (1.38 to 1.43)	260 (163 to 386)	0.23 (0.14 to 0.34)	1178 (733 to 1708)	0.35 (0.21 to 0.5)	1.41 (1.39 to 1.44)
South Asia	9569 (7222 to 12426)	3 (2.22 to 3.97)	47727 (33254 to 65420)	4.57 (3.11 to 6.37)	1.31 (1.23 to 1.39)	852 (527 to 1323)	0.26 (0.16 to 0.39)	4222 (2531 to 6645)	0.4 (0.23 to 0.61)	1.32 (1.24 to 1.4)
Southeast Asia	8231 (6354 to 10658)	5.29 (3.97 to 6.85)	39748 (29983 to 51956)	8.81 (6.52 to 11.6)	1.69 (1.63 to 1.76)	727 (460 to 1087)	0.46 (0.29 to 0.67)	3513 (2154 to 5227)	0.77 (0.47 to 1.13)	1.71 (1.65 to 1.77)
East Asia	22539 (16868 to 30633)	4.57 (3.37 to 6.14)	132966 (94568 to 179236)	7.02 (4.92 to 9.41)	1.2 (1.07 to 1.33)	2032 (1229 to 3231)	0.41 (0.25 to 0.61)	11891 (7128 to 18740)	0.63 (0.38 to 0.97)	1.22 (1.09 to 1.35)
Oceania	64 (48 to 84)	4.13 (3.02 to 5.49)	220 (167 to 290)	4.93 (3.59 to 6.75)	0.54 (0.5 to 0.58)	6 (3 to 9)	0.37 (0.22 to 0.54)	20 (12 to 30)	0.44 (0.26 to 0.67)	0.53 (0.49 to 0.57)
Western Sub-Saharan Africa	3896 (2614 to 5364)	7.37 (4.86 to 10.22)	9600 (6462 to 13383)	8.31 (5.56 to 11.35)	0.28 (0.18 to 0.37)	345 (200 to 574)	0.65 (0.37 to 1.02)	852 (501 to 1396)	0.73 (0.43 to 1.18)	0.3 (0.2 to 0.39)
Eastern Sub-Saharan Africa	2448 (1601 to 3648)	6.07 (3.85 to 8.48)	7892 (5269 to 11602)	7.63 (4.94 to 10.68)	0.82 (0.72 to 0.92)	218 (122 to 378)	0.53 (0.29 to 0.86)	702 (405 to 1165)	0.67 (0.38 to 1.11)	0.83 (0.73 to 0.93)
Central Sub-Saharan Africa	953 (611 to 1404)	8.77 (5.51 to 12.49)	3113 (2047 to 4682)	10.48 (6.77 to 15.36)	0.64 (0.5 to 0.78)	86 (47 to 149)	0.78 (0.43 to 1.28)	279 (159 to 470)	0.93 (0.52 to 1.54)	0.66 (0.51 to 0.8)
Southern Sub-Saharan Africa	1726 (1155 to 2411)	8.66 (5.61 to 12.18)	3929 (2660 to 5613)	9.8 (6.4 to 13.64)	0.41 (0.29 to 0.52)	154 (88 to 247)	0.77 (0.44 to 1.22)	352 (203 to 573)	0.87 (0.5 to 1.39)	0.42 (0.3 to 0.53)

ASPR, Age-Standardized Prevalence Rates; ASYR, Age-Standardized YLDs (years lived with disability rates) Rates

**Figure 1:**
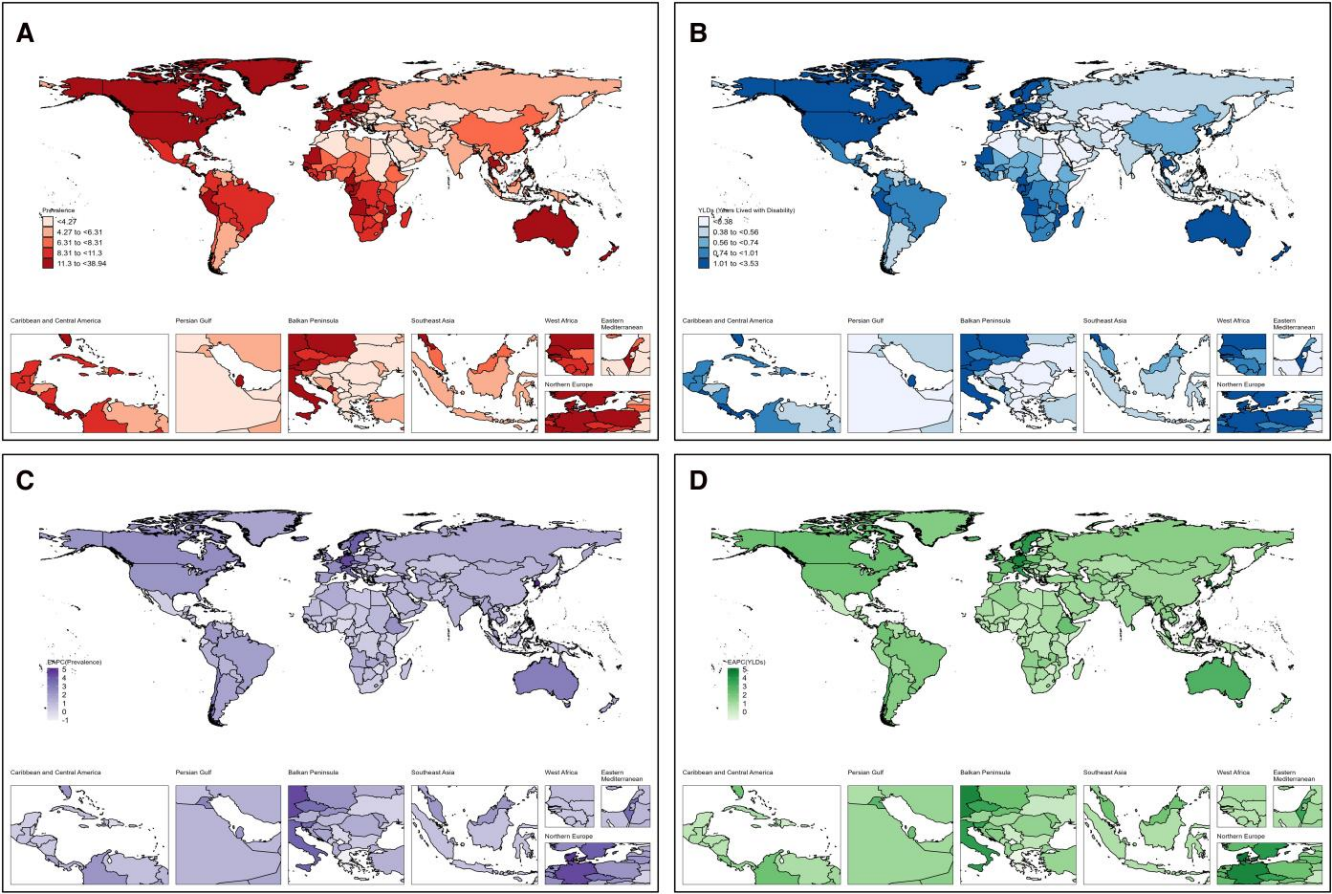
Global, regional, and national distribution of heart failure burden attributable to atrial fibrillation and flutter (AF/AFL), 1990–2021. (A) Age-standardized prevalence rate (ASPR per 100 000 population) in 2021. (B) Age-standardized years lived with disability (YLD) rates (ASYR per 100 000 population) in 2021. (C) Estimated annual percentage change (EAPC) in age-standardized prevalence rate from 1990 to 2021. (D) Estimated annual percentage change (EAPC) in age-standardized years lived with disability (YLD) rates from 1990 to 2021.

At the regional level, all GBD regions experienced increasing ASR of both prevalence and YLDs without evidence of decline. Western Europe, high-income North America, East Asia, and high-SDI regions already exhibited high burden in 1990 and continued to rank among the highest by 2021. For instance, in Western Europe, ASPR rose from 7.89 (95% UI: 5.72–10.46) in 1990 to 16.9 (95% UI: 12.28–22.38) in 2021 (EAPC: 2.97%, 95% CI: 2.71–3.23). Australasia showed the fastest relative increase, with prevalence cases rising nearly 4.8-fold (from 2676 to 15 468) and an EAPC of 2.8% (95% CI: 2.4–3.2). By contrast, some low-SDI regions such as Central Asia had relatively lower absolute levels but still exhibited upward trends; ASPR rose from 1.55 (95% UI: 1.07–2.19) in 1990 to 1.86 (95% UI: 1.20–2.64) in 2021(EAPC: 0.82%, 95% CI: 0.41–1.23) (*Table 1*, *Figs. 1A–D*).

At the national level, consistent with regional patterns, rapid increases were observed in certain countries. The Republic of Korea showed the largest relative increase, with prevalence cases rising from 816 (95% UI: 597–1076) in 1990 to 12 846 (95% UI: 9895–16 485) in 2021, representing a ∼15-fold increase; ASPR rose from 3.71(95% UI: 2.53–5.03) in 1990 to 13.77(95% UI: 10.62–17.60) in 2021 (EAPC: 5.15%, 95% CI: 4.76–5.55). China, despite its large population base, also demonstrated steady growth (EAPC: 1.19%, 95% CI: 1.04–1.34), with ASPR increasing from 4.51(95% UI: 3.33–6.03) in 1990 to 6.9(95% UI: 4.80–9.35) in 2021. In Northern and Western Europe, Switzerland (EAPC: 4.78%) and Sweden (EAPC: 3.89%) showed marked increases, with Sweden reaching the highest global ASPR (38.94 per 100 000). Germany also demonstrated a notable increase (EAPC: 4.41%) with cases rising from 6.42(95% UI: 4.54–8.63) to 15.47(95% UI: 10.35–21.49). Conversely, Greece exhibited the most evident decline, with EAPC of −1.02% (95% CI: −1.37 to −0.67) (*Figs. 1A–D*, Supplementary Table S2).

### Age and sex-specific trends

**Figure 2:**
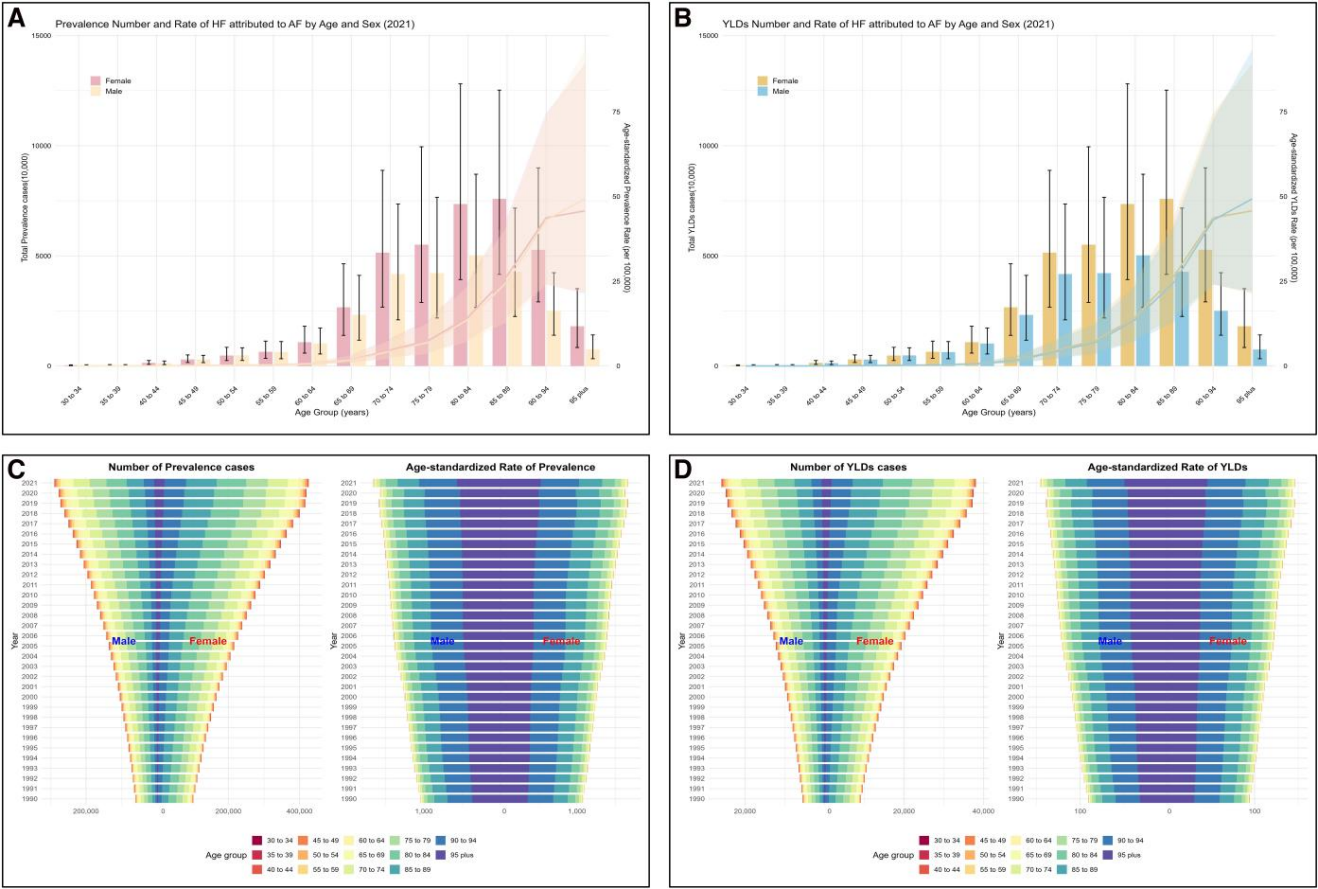
Burden of heart failure attributable to atrial fibrillation/flutter (AF/AFL) by age and sex. (A) Number of heart failure prevalence cases and age-standardized prevalence rate (ASR) attributable to AF/AFL by age group and sex. (B) Number of heart failure YLDs cases and age-standardized prevalence rate (ASR) attributable to AF/AFL by age group and sex. The shaded area represents the 95% uncertainty interval. (C) Butterfly plot of the number of Prevalence cases and ASR by 5-year age group and sex, 1990–2021. (D) Butterfly plot of the number of YLDs cases and ASR by 5-year age group and sex, 1990–2021.

The age distribution of AF-attributed HF prevalence (*Figure 2A, 2C*) demonstrated a clear concentration in individuals aged 60–95 years. Beyond age 65, women consistently outnumbered men, with the sex gap widening with increasing age. For instance, in the 65–69 age group, women accounted for 28 905 cases versus 25 144 in men (female-to-male ratio ≈ 1.1), whereas in the 85–89 age group the gap widened to 86 130 vs. 48 491 cases (ratio ≈ 1.8), and among those ≥95 years, women had 20 786 cases compared with 8602 in men (ratio ≈ 2.4). However, sex differences in ASPR were generally not significant, except for certain older age groups. For example, women aged 80–89 years had slightly higher prevalence (302.54 per 100 000) than men (281.07 per 100 000), while men ≥95 years had a higher prevalence (568.9 vs. 527.8 per 100 000).

YLDs displayed similar patterns (*Figure 2B, 2D*). In nearly all age groups, women had higher absolute YLDs, with the disparity most pronounced among older adults. For instance, in the 80–84 age group, women had 7351 YLDs (14.43 per 100 000) compared with 5028 (13.72 per 100 000) in men; in the 85–89 group, women had 7588 (26.65 per 100 000) vs. 4278 (24.79 per 100 000) in men. Notably, women ≥85 years experienced 46–77% higher absolute YLDs than men. Nevertheless, age-standardized YLD rates between sexes remained broadly comparable (*Table 2*).

**Table 2:** Age- and sex-specific prevalence and years lived with disability (YLDs) for heart failure attributable to atrial fibrillation and flutter in 2021

		Prevalence numbers	ASPR	YLDs numbers	ASYR
30–34 years	Male	354 (196 to 567)	0.12 (0.06 to 0.19)	33 (17 to 58)	0.01 (0.01 to 0.02)
	Female	325 (181 to 508)	0.11 (0.06 to 0.17)	30 (16 to 52)	0.01 (0.01 to 0.02)
35–39 years	Male	402 (231 to 636)	0.14 (0.08 to 0.22)	37 (19 to 64)	0.01 (0.01 to 0.02)
	Female	404 (235 to 629)	0.15 (0.08 to 0.23)	37 (20 to 65)	0.01 (0.01 to 0.02)
40–44 years	Male	1402 (779 to 2329)	0.56 (0.31 to 0.92)	130 (65 to 229)	0.05 (0.03 to 0.09)
	Female	1534 (860 to 2443)	0.62 (0.35 to 0.98)	142 (74 to 252)	0.06 (0.03 to 0.1)
45–49 years	Male	3137 (1832 to 4700)	1.32 (0.77 to 1.98)	291 (154 to 486)	0.12 (0.06 to 0.2)
	Female	3244 (1944 to 4856)	1.38 (0.82 to 2.06)	301 (161 to 504)	0.13 (0.07 to 0.21)
50–54 years	Male	5208 (3135 to 8034)	2.35 (1.41 to 3.62)	483 (252 to 828)	0.22 (0.11 to 0.37)
	Female	5158 (3129 to 8130)	2.31 (1.4 to 3.6)	478 (248 to 849)	0.21 (0.11 to 0.38)
55–59 years	Male	6843 (4125 to 10905)	3.51 (2.12 to 5.6)	633 (331 to 1111)	0.33 (0.17 to 0.57)
	Female	6991(4206 to 10989)	3.48 (2.09 to 5.47)	647 (348 to 1132)	0.32 (0.17 to 0.56)
60–64 years	Male	11088 (7112 to 16542)	7.13 (4.57 to 10.64)	1025 (546 to 1726)	0.66 (0.35 to 1.11)
	Female	11682 (7594 to 17563)	7.1 (4.62 to 10.68)	1081 (586 to 1808)	0.66 (0.36 to 1.1)
65–69 years	Male	25144 (14925 to 39466)	19.07 (11.32 to 29.94)	2316 (1173 to 4116)	1.76 (0.89 to 3.12)
	Female	28905 (17788 to 45014)	20.07 (12.35 to 31.26)	2661 (1392 to 4643)	1.85 (0.97 to 3.22)
70–74 years	Male	45709 (27452 to 73177)	47.42 (28.48 to 75.92)	4173 (2095 to 7353)	4.33 (2.17 to 7.63)
	Female	56471 (34469 to 90223)	51.6 (31.49 to 82.44)	5142 (2671 to 8884)	4.7 (2.44 to 8.12)
75–79 years	Male	46821 (26561 to 74103)	78.31 (44.43 to 123.94)	4212 (2180 to 7662)	7.05 (3.65 to 12.82)
	Female	61172 (36405 to 95406)	84.85 (50.49 to 132.33)	5511 (2886 to 9954)	7.64 (4 to 13.81)
80–84 years	Male	56539 (34088 to 86308)	154.26 (93 to 235.48)	5028 (2658 to 8707)	13.72 (7.25 to 23.76)
	Female	82554 (51338 to 126016)	162.09 (100.8 to 247.42)	7351 (3925 to 12815)	14.43 (7.71 to 25.16)
85–89 years	Male	48491 (29736 to 71016)	281.07 (172.36 to 411.62)	4278 (2249 to 7168)	24.79 (13.04 to 41.55)
	Female	86130 (54165 to 126133)	302.54 (190.26 to 443.05)	7588 (4160 to 12526)	26.65 (14.61 to 44)
90–94 years	Male	28760 (16838 to 44407)	493.43 (288.89 to 761.9)	2509 (1398 to 4238)	43.05 (23.98 to 72.71)
	Female	60281 (36243 to 92305)	499.81 (300.5 to 765.33)	5275 (2911 to 8998)	43.74 (24.13 to 74.6)
95+ years	Male	8602 (4126 to 15510)	568.9 (272.88 to 1025.76)	746 (331 to 1412)	49.31 (21.9 to 93.35)
	Female	20786 (10041 to 37214)	527.79 (254.95 to 944.93)	1804 (841 to 3511)	45.8 (21.34 to 89.15)

ASPR, Age-Standardized Prevalence Rates; ASYR, Age-Standardized YLDs (years lived with disability rates) Rates

### Joinpoint analysis

**Figure 3:**
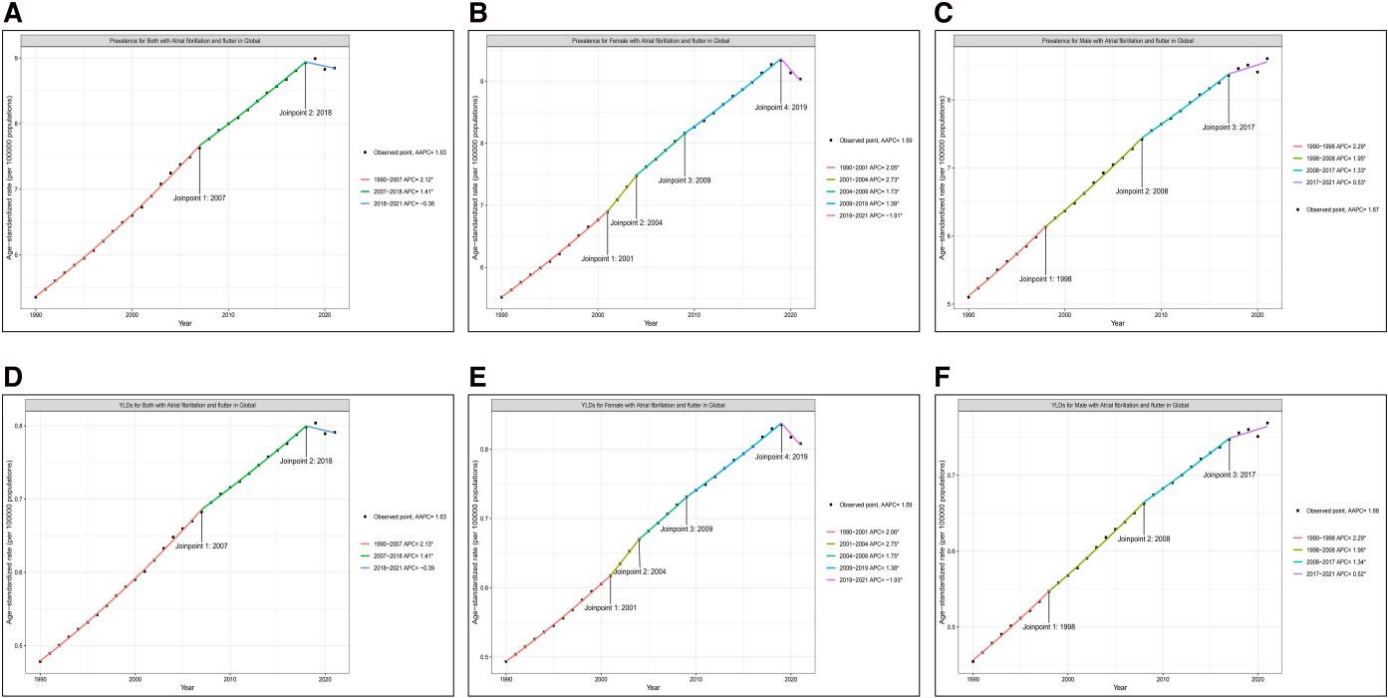
Joinpoint regression analysis of global age-standardized prevalence and YLDs of heart failure attributable to atrial fibrillation/flutter (AF/AFL), 1990–2021. (A–C) Trends in age-standardized prevalence rate of HF attributable to AF/AFL, shown globally (A), by sex (B: males; C: females). (D–F) Trends in age-standardized YLD rate of HF attributable to AF/AFL, shown globally (D), by sex (E: males; F: females). Solid lines represent estimated trends, with colored segments indicating distinct joinpoint periods; annual percent change (APC) values are annotated for each segment. Asterisks (*) denote statistically significant APC (*P* < .05).

Joinpoint regression identified distinct temporal inflection points in AF/AFL–attributed HF trends (*Figs. 3A–F*, Supplementary Table S3). Globally, prevalence rate increased during 1990–2007 (APC: 2.12%), 2007–2018 (APC: 1.41%), but plateaued with a slight decline from 2018–2021 (APC: −0.36%). Sex-specific trends revealed heterogeneity: among women, APC was 2.05% during 1990–2001, 2.73% during 2001–2004, and 1.73% during 2004–2009, followed by smaller increases or declines thereafter. In men, the burden continued to rise in most intervals, with APC ranging from 1.95% (1998–2008) to 0.53% (2017–2021). YLD trends largely paralleled those of prevalence.

### Trends by Socio-Demographic Index (SDI)

**Figure 4:**
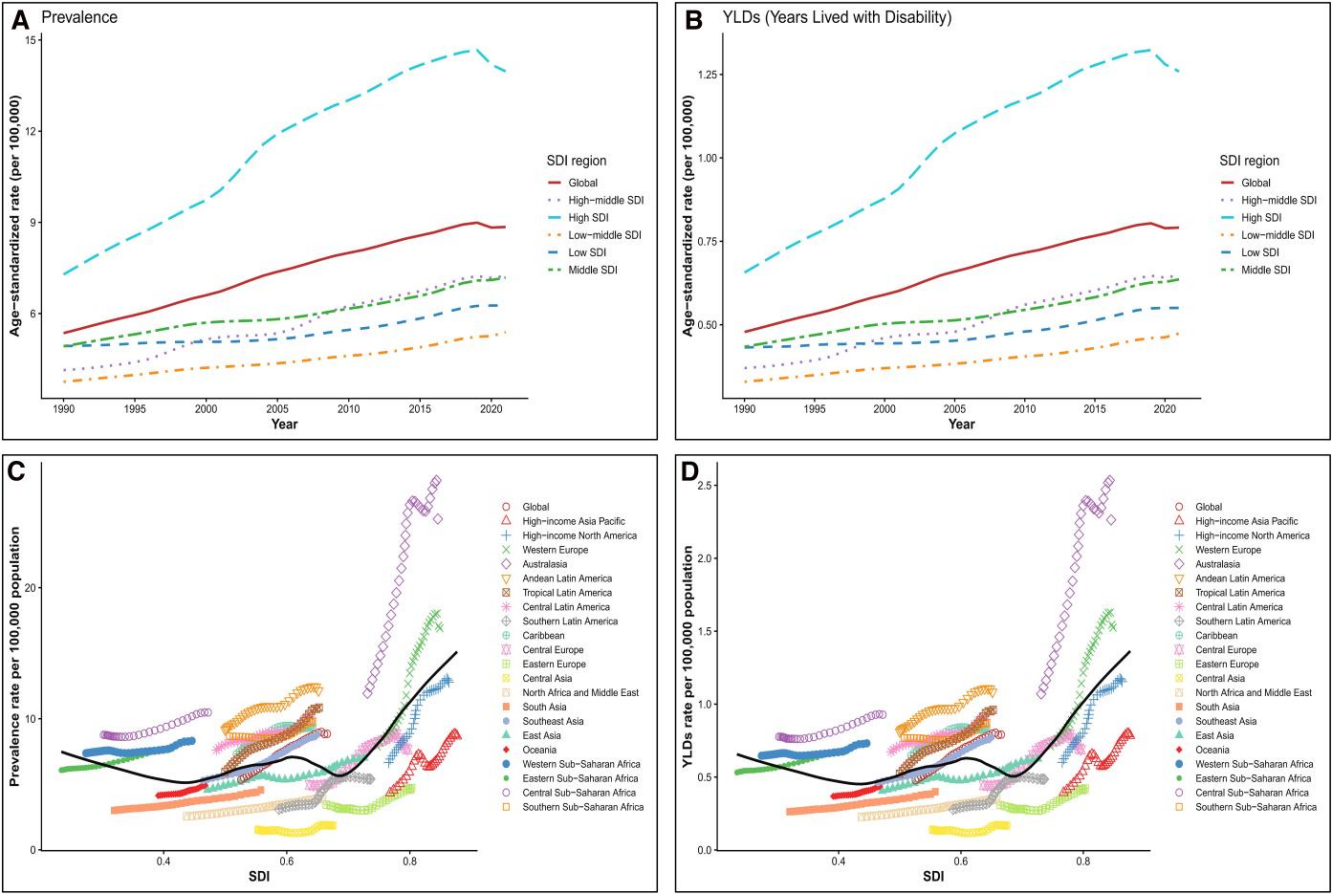
Socio-demographic index (SDI)–related trends of heart failure attributable to atrial fibrillation/flutter (AF/AFL), 1990–2021. (A) Global and SDI quintile trends in age-standardized prevalence rate of AF-attributed HF. (B) Global and SDI quintile trends in age-standardized YLD rate of AF-attributed HF. (C) Relationship between SDI and prevalence rates across 21 GBD regions, showing global and regional trajectories. (D) Relationship between SDI and YLD rates across 21 GBD regions.

Across SDI strata, high-SDI regions consistently exhibited the highest ASPR (13.97 95%UI: 10.31 to 18.36) and ASYR (1.26 95%UI: 0.76–1.91) of AF-attributed HF (*Figure 4A*), with EAPC of 2.39% (95% CI: 2.19–2.59). In contrast, low- and medium-SDI regions maintain the lowest burden levels. The 2021 ASPR was 5.39 (95% UI: 3.82–7.19), corresponding to an EAPC of 1.09 (95% CI: 1.05 to 1.14), and the ASYR was 0.47 (95% UI: 0.29–0.72), corresponding to an EAPC of 1.10 (95% CI: 1.06 to 1.15). Globally, both prevalence and YLDs increased steadily (*Figure 3B*). Correlation analysis confirmed a strong positive association between ASR and SDI for both prevalence and YLDs (r = 0.992, *P* < .001) (*Figs. 4C–D*, Supplementary Table S4). High-SDI regions (e.g. Western Europe, North America, Australasia) clustered in the upper-right quadrant, whereas low-SDI regions (e.g. Sub-Saharan Africa, Central Asia) clustered at the lower-left. Middle-SDI regions, such as Latin America, exhibited ‘catch-up’ patterns with increasing burden (EAPC: 1.14%, 95% CI: 1.09–1.20).

### Decomposition and inequality analysis

**Figure 5:**
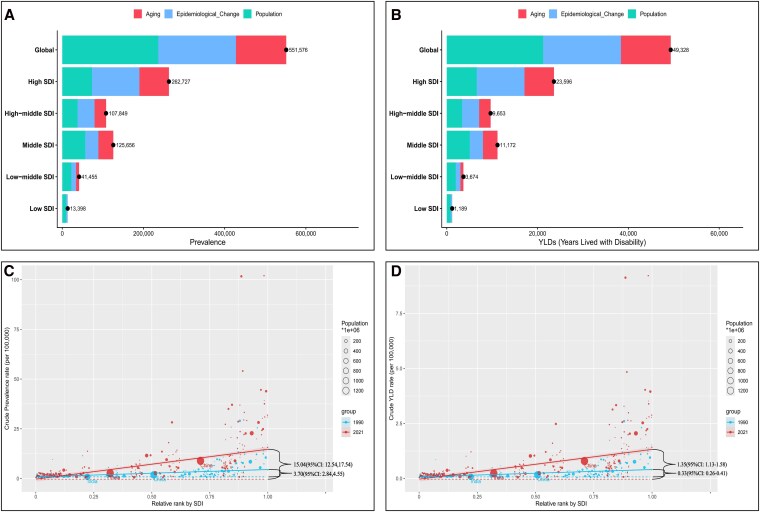
Decomposition and inequality analysis of heart failure (HF) burden attributable to atrial fibrillation/flutter (AF/AFL), 1990–2021. (A) Decomposition of changes in AF-attributed HF prevalence into contributions from population growth, population aging, and epidemiological change at global and SDI quintile levels. (B) Decomposition of changes in AF-attributed HF YLDs into the same three components. (C) Cross-national inequality in prevalence: relationship between SDI and age-standardized prevalence rates. (D) Cross-national inequality in YLDs: relationship between SDI and age-standardized YLD rates.

Decomposition analysis indicated that population growth was the primary driver of increased prevalence (contributing 42.91% of growth) and YLDs (43.03%), followed by ageing (particularly in middle-SDI regions: 29.08% for prevalence, 28.75% for YLDs) and epidemiological changes (predominant in high-SDI regions: 44.42% for prevalence, 44.56% for YLDs) (*Figs. 5A–B*, Supplementary Table S4). Notably, cross-national inequality widened substantially. In 1990, the absolute difference in prevalence between the highest- and lowest-SDI countries was 3.70 per 100 000 (95% CI: 2.84–4.55), which expanded to 15.04 per 100 000 (95% CI: 12.54–17.54) by 2021 (*Figs. 5C–D*, Supplementary Table S5).

### Forecast to 2040

**Figure 6:**
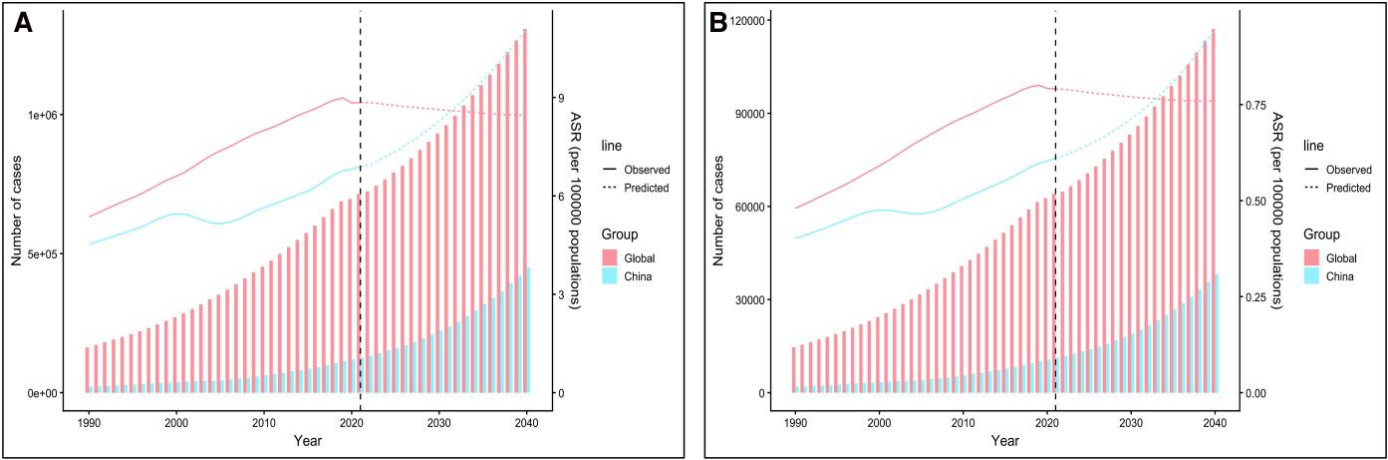
Projection of heart failure (HF) burden attributable to atrial fibrillation/flutter (AF/AFL), 1990–2040. (A) Observed (1990–2021) and predicted (2022–2040) number of prevalent HF cases attributable to AF/AFL globally and in China, with corresponding age-standardized prevalence rates (ASR) shown as solid lines. (B) Observed and predicted number of YLDs due to AF-attributed HF globally and in China, with corresponding age-standardized YLD rates shown as solid lines. Vertical dashed lines indicate the transition from observed to forecasted years.

Projections suggest that the burden of AF-attributed HF will continue to rise by 2040. The number of prevalent cases is estimated to reach 1 307 469 (95% UI: 661 547–1 953 391), corresponding to 8.45 per 100 000 (95% UI: 4.28–12.63). YLDs are projected to increase to 117 155 (95% UI: 54 971–179 339), or 0.76 per 100 000 (95% UI: 0.36–1.16). China is expected to maintain a steadily rising burden throughout the forecast horizon (*Figs. 6A–B*, Supplementary Tables S7).

## Discussion

### Major findings and global context

Using the standardized GBD 2021 framework, this study provides the first comprehensive three-level (global–regional–national) quantification of HF burden attributable to AF/flutter (AF/AFL) from 1990 to 2021. We found that both absolute case numbers and age-standardized rates—including prevalence and years lived with disability (YLDs)—showed a sustained upward trajectory, with no evidence of decline in any GBD region. The burden was disproportionately concentrated among older adults, and women outnumbered men beyond age 65. Decomposition analysis demonstrated that population growth and ageing were the primary global drivers, while in high–Socio-demographic Index (SDI) regions, epidemiological changes—reflecting risk factor transitions, accumulation of multimorbidity, and improved survival of chronic cardiovascular patients—were dominant. Joinpoint regression showed acceleration of growth before 2010, followed by a plateau or slight decline after 2018, though the overall trajectory remained upward. Forecasts to 2040 suggest that AF-attributed HF burden will continue to increase globally, with populous countries such as China playing a pivotal role in shaping future trends. Collectively, these findings provide compelling epidemiological justification for integrating standardized AF management into both primary and secondary prevention pathways for HF.

### Comparison with previous studies

Prior GBD analyses and multicentre surveys have largely focused on the overall burden of HF and its principal causes—such as ischaemic heart disease and hypertensive heart disease—or on specific age subgroups, while the direct contribution of AF/AFL to HF has received limited attention.^4,13,14^ Our study extends this evidence base in three important ways. First, by isolating AF/AFL as a distinct cause and describing its longitudinal evolution in age-standardized rates and YLDs, we highlight nuances in arrhythmia-related HF that aggregated analyses of ‘total HF’ cannot capture. Second, we identified stark inequalities: high-SDI regions had both higher absolute and relative burdens, and the gap between high- and low-SDI countries widened over time. These quantitative insights provide critical support for linking evidence-based practice, resource allocation, and health equity considerations. Third, we incorporated methodological innovations—including joinpoint regression, decomposition, and BAPC forecasting—into a unified ‘occurrence–drivers–outlook’ framework, yielding more actionable insights for policy and clinical practice. Our findings are consistent with cohort and registry studies showing that AF independently increases risks of HF hospitalization and mortality,^18,19^ but we extend the evidence by providing population-level, cross-national comparability. Furthermore, our results reinforce mechanistic understanding of the bidirectional AF–HF relationship, while population-level data underscore the necessity of integrated management to achieve reciprocal clinical benefits.^20–23^

### Age and sex patterns: women dominate in numbers but not in rates

The AF-attributed HF burden was concentrated among individuals aged 60–95 years, consistent with the well-established age-related rise in AF prevalence.^24,25^ Beyond 65 years, women consistently exceeded men in absolute cases and YLDs, with the female-to-male ratio rising from ∼1.1 in those aged 65–69 to 2.4 in those ≥95 years. This pattern diverges somewhat from prior overall HF estimates, which reported higher case counts in women only beyond 80 years,^4^ while the latest 2023 GBD report indicated higher cardiovascular mortality and DALY rates in men.^3^ Several factors may account for this discrepancy. Women’s longer life expectancy increases representation in the oldest age strata where AF and HF intersect.^26^ Women are also more prone to HF with preserved ejection fraction (HFpEF),^27,28^ which is more strongly associated with AF than HF with reduced ejection fraction (HFrEF).^29^ Additional contributions may come from sex differences in comorbidity profiles (hypertension, obesity, diabetes), health-seeking behaviour, and diagnostic patterns.^30,31^ However, age-standardized rates showed relatively minor sex differences, indicating that the observed numerical gap mainly reflects demographic and survival structures rather than intrinsic biological susceptibility. Clinically, this nuanced interpretation is important: while older women should be prioritized for AF screening, anticoagulation assessment, and rhythm management optimization, clinicians should avoid overinterpreting rate differences as evidence of inherent sex-specific vulnerability. Recent guidelines increasingly emphasize sex-specific risk stratification in AF complications,^32^ and our findings support an integrated approach that considers both biological and sociocultural determinants in addressing the female AF–HF burden.

### Socioeconomic gradient and decomposition insights: dual drivers of population dynamics and epidemiological transition

High-SDI regions consistently exhibited higher age-standardized prevalence and YLD rates of AF-attributed HF throughout the study period. Decomposition analysis revealed heterogeneous drivers across development levels. In high-SDI regions, epidemiological change was the major contributor—reflecting intensified exposure to cardiovascular risk factors (hypertension, diabetes, obesity),^2^ rising multimorbidity, and the paradox of improved survival from ischaemic heart disease and HF, which accumulates chronic disease burden (‘competing risks’).^33,34^ Several reinforcing mechanisms underlie this SDI-associated gradient. First, improved survival and the ‘competing risks’ paradox: effective management of ischaemic heart disease, hypertension, and HF in high-income settings has dramatically increased patient longevity, meaning more individuals survive long enough to develop AF, and those with AF live longer with residual cardiac dysfunction—directly inflating the prevalent pool of AF-attributed HF.^35^ Second, older demographic profiles in high-SDI countries amplify this effect: AF prevalence rises steeply with age, and the combination of high life expectancy and high AF incidence in older adults disproportionately concentrates AF-attributed HF burden in these regions. Third, greater diagnostic intensity in resource-rich settings—including widespread availability of ECG, Holter monitoring, echocardiography, and cardiac imaging—enables detection of AF and HF cases that would remain clinically silent or undetected in under-resourced environments, directly inflating apparent burden through ascertainment bias. Fourth, and conversely, systematic underdiagnosis in low-SDI regions—driven by limited ECG access, shortage of trained cardiologists, and incomplete civil registration—substantially underestimates the true burden in these settings, further widening the observed SDI gradient.^35^ By contrast, growth in middle- and low-SDI regions was primarily driven by population expansion and ageing, manifesting as a ‘quantitative surge’—consistent with decomposition estimates showing population growth and ageing together accounting for >70% of burden increase in these regions. These regions also face the dual challenge of epidemiological transition—declining infectious disease burden alongside rising non-communicable disease prevalence—within constrained healthcare infrastructure.^36^ The absolute prevalence gap between the highest and lowest SDI quintiles widened (from 3.70 per 100 000 [95% CI: 2.84–4.55] in 1990 to 15.04 per 100 000 [95% CI: 12.54–17.54] in 2021), reflecting disparities in incidence, diagnostic ascertainment, diagnostic capacity, treatment access, and survival.^37^ This dual-track dynamic demands tailored policy responses: high-SDI regions should prioritize improving rhythm-control quality (early AF ablation, optimized rate control), precision use of device therapy, and integrated multimorbidity management,^38,39^ while in low- and middle-SDI regions, emphasis should be placed on strengthening AF screening infrastructure, enhancing blood pressure and metabolic risk management in primary care, and expanding access to anticoagulation through generic warfarin or subsidized direct oral anticoagulants (DOACs).^40^ Critically, investment in ECG infrastructure and community-based AF screening is essential not only to close the treatment gap but also to correct the systematic underdiagnosis that currently masks the true burden in these populations. Investments in health information systems to improve AF and HF surveillance are crucial for evidence-based resource allocation.

### Temporal dynamics and forecasts: emerging plateaus amid continued growth

Joinpoint regression revealed nonlinear temporal trajectories, with accelerated growth from the late 1990s to early 2010s, followed by plateauing or mild decline in some indicators after 2018. This inflection may reflect the cumulative benefits of expanded anticoagulation coverage, rhythm-control strategies (including catheter ablation), and comprehensive HF programmes.^20,40^ Advances in guideline-directed HF therapy (RAAS inhibitors, β-blockers, SGLT2 inhibitors) and device therapy (CRT-D) may also have contributed to attenuating AF-related HF progression.^22,41^

Nevertheless, significant heterogeneity persists: trends remain upward among men and in several countries, underscoring uneven diffusion of therapeutic advances and a ‘management gap’.^42^ For example, although AF ablation has demonstrated efficacy in reducing HF hospitalization, its availability remains limited outside tertiary centres in high-income countries.^43^ Similarly, anticoagulation coverage varies widely across and within countries. BAPC projections to 2040 indicate that global case numbers will nearly double, driven by demographic inertia and the slow-changing epidemiology of AF. This trend does not signify therapeutic failure but reflects the immense challenge of ageing populations, especially in middle-income nations undergoing rapid demographic transitions.^44^ China, with its vast and rapidly ageing population, exemplifies this trajectory and will critically shape global estimates.^45^ Strategically, the implications are twofold. In regions already demonstrating benefit (e.g. Western Europe), focus should be on scaling proven interventions—population-based AF screening with wearable/ambulatory ECG,^46^ early rhythm-control strategies,^47^ and bundled care models for AF–HF multimorbidity.^48^ In resource-limited settings, emphasis must remain on establishing foundational diagnostic and anticoagulation capacity—‘infrastructure first’—to delay burden peaks and reduce preventable deaths.^49^

## Methodological strengths and limitations

### Strengths

This study leveraged the comprehensive GBD 2021 dataset and standardized methodology, enabling cross-national comparisons across multiple dimensions (age, sex, SDI, time). By integrating joinpoint regression, decomposition, and BAPC forecasting, we constructed a coherent framework tracing ‘trends–drivers–forecasts.’ By focusing on ‘AF/AFL-attributable HF’ as a distinct etiological entity, we address an evidence gap in overall HF burden studies and provide direct reference for integrated arrhythmia–HF management strategies.

### Limitations

Several limitations merit acknowledgment. First, data quality and heterogeneity. GBD estimates rely on heterogeneous data sources and statistical modelling frameworks. Sparse data availability in low-resource settings may introduce substantial uncertainty and systematic underestimation of true disease burden, as reflected in the wider uncertainty intervals observed for low-SDI regions. Second, diagnostic and attribution bias. Causal attribution of HF to AF/AFL depends on administrative and surveillance data, whose completeness and coding accuracy vary considerably across health systems. In settings with limited diagnostic infrastructure and civil registration, AF is likely to be systematically underdiagnosed, leading to under-attribution of AF-related HF burden. Furthermore, in patients with multiple comorbidities, primary-cause attribution may inadequately capture the true causal pathway, as the contributing role of AF may be obscured by coexisting conditions such as ischaemic heart disease or hypertension. Despite GBD's rigorous modelling framework, residual confounding and misclassification cannot be fully excluded. Third, causal inference constraints. The GBD framework attributes HF to AF/AFL in a unidirectional manner; however, the biological relationship between the two conditions is inherently bidirectional. Pre-existing HF promotes AF onset and maintenance through elevated atrial pressure, myocardial fibrosis, and structural remodelling, while AF in turn drives HF through tachycardia-induced cardiomyopathy and neurohumoral activation. Disentangling these reciprocal effects requires prospective individual-level data with careful temporal sequencing, which is beyond the scope of population-based burden estimation. Fourth, forecast assumptions. Our BAPC projections assume stable demographic trends and healthcare environments throughout the forecast horizon. These models may not adequately account for disruptive innovations—such as widespread adoption of pulsed-field ablation or wearable AF screening technologies—or for potential policy shifts and global health crises (e.g. pandemics) that could substantially alter AF and HF trajectories. Fifth, resolution of individual-level heterogeneity. Population-level analysis cannot disentangle individual-level variation, including the differential associations of AF with HF with preserved versus reduced ejection fraction (HFpEF vs. HFrEF), or the relative benefits of catheter ablation versus pharmacological rhythm control across patient subgroups. Similarly, the inability to distinguish AF phenotypes (paroxysmal, persistent, long-standing persistent) limits mechanistic interpretation and the clinical granularity of our findings.

### Implications for future research and practice

In low-resource settings, strengthening AF and HF surveillance and registry systems remains essential. Wearable and remote ECG monitoring could reduce diagnostic gaps. Prospective cohort studies and quasi-experimental designs (e.g. instrumental variable analyses of regional AF treatment intensity) are needed to clarify the causal effects of rhythm-control strategies on HF incidence and progression. Mobile health platforms and artificial intelligence could support community-based AF screening, remote anticoagulation monitoring, and decision support for rate and rhythm control. Future work should also assess the cost-effectiveness of integrated AF screening–anticoagulation–rhythm control–HF management pathways across SDI contexts. Policymakers may consider incentive mechanisms, such as rewarding early AF detection in high-risk elderly women, incorporating anticoagulation quality metrics into primary care, and coordinating integrated AF–HF care delivery.

## Conclusions

AF/AFL has emerged as a critical driver of the global HF burden, with ageing and epidemiological transitions compounding regional disparities. Integrating standardized AF management—including systematic screening, stroke prevention with anticoagulation, early rhythm-control strategies, and timely catheter ablation—into HF prevention and management pathways represents a high-leverage strategy to address the projected doubling of burden over the next two decades. Balancing population-level health system strengthening in low-resource settings with precision medicine optimization in high-resource settings will be essential for achieving equitable and effective outcomes. Our findings provide a robust epidemiological foundation for positioning AF–HF as a comprehensive cardiovascular health priority in global and national health agendas.

## Supplementary Material

xvag094_Supplementary_Data

## Declarations

### Data availability

All data can be performed at https://vizhub.healthdata.org/gbd-results/.

### Pre-registered Clinical Trial Number

None supplied.
