# Supplementary material for: Global burden of heart failure attributable to atrial fibrillation and flutter, insights from GBD 2021
Source: ESC Heart Fail. 2026 Mar 30;13(2):xvag094. doi: 10.1093/eschf/xvag094 (PMC13122623; doi:10.1093/eschf/xvag094)

**Supplementary Materials**

**contents**

[TableS1 Checklist of Information That should be Included in New Reports of Global Health Estimates 2](#_Toc3979)

[TableS2 204 Countries of Heart Failure Attributable to Atrial Fibrillation and Flutter From 1990 to 2021: Prevalence, Years Lived With Disability (YLDs), Age-Standardized Rates, and Estimated Annual Percentage Changes (EAPC) 4](#_Toc25588)

[TableS3 Joinpoint Analysis of Burden of Heart Failure Attributable to Atrial Fibrillation and Flutter From 1990 to 2021. 25](#_Toc2201)

[TableS4 Correlation Coefficients (r) and P-Values Between Age-Standardized Prevalence Rates (ASPR), Age-Standardized Years Lived with Disability Rates (ASYR), and Socio-Demographic Index (SDI) by Global Burden of Disease Region, 1990–2021 132](#_Toc10877)

[TableS5 Cross-National Inequality in Age-Standardized Prevalence of Heart Failure Attributable to Atrial Fibrillation and Flutter: Absolute Differences. 134](#_Toc29959)

[TableS6 Decomposition Analysis of Changes in the Burden of Heart Failure Attributable to Atrial Fibrillation and Flutter, 1990–2021: Contributions of Aging, Population Growth, and Epidemiological Changes by Socio-Demographic Index (SDI) Level, Sex, and Measure (Prevalence and Years Lived with Disability [YLDs]) 155](#_Toc9662)

[TableS7 Projected Global and China Burden of Heart Failure Attributable to Atrial Fibrillation and Flutter, 2022–2040 158](#_Toc16328)

[FigureS1 Global Burden of Heart Failure Attributed to Atrial fibrillation and Atrial Flutter, 1990 160](#_Toc5516)

#
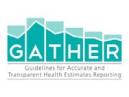
TableS1 **Checklist of Information That should be Included in New Reports of Global Health Estimates**

| **Item #** | **Checklist item**  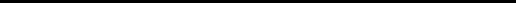 | **Reported**  **on page #** |
| --- | --- | --- |
| **Objectives and funding** | | |
| **1** | Define the indicator(s), populations (including age, sex, and geographic entities), and time period(s) for which estimates were made. | 4,5 |
| **2** | List the funding sources for the work. | 17 |
| **Data Inputs** | | |
| *For all data inputs from multiple sources that are synthesized as part of the study:* | | |
| **3** | Describe how the data were identified and how the data were accessed. | 4 |
| **4** | Specify the inclusion and exclusion criteria. Identify all ad-hoc exclusions. | 5 |
| **5** | Provide information on all included data sources and their main characteristics. For each data source used, report reference information or contact name/institution, population represented, data collection method, year(s) of data collection, sex and age range,  diagnostic criteria or measurement method, and sample size, as relevant. | 5 |
| **6** | Identify and describe any categories of input data that have potentially important biases (e.g., based on characteristics listed in item 5). | 5 |
| *For data inputs that contribute to the analysis but were not synthesized as part of the study:* | | |
| **7** | Describe and give sources for any other data inputs. | 5 |
| *For all data inputs:* | | |
| **8** | Provide all data inputs in a file format from which data can be efficiently extracted (e.g., a spreadsheet rather than a PDF), including all relevant meta-data listed in item 5. For any data inputs that cannot be shared because of ethical or legal reasons, such as third-party ownership, provide a contact name or the name of the institution that retains the right to the data. | 5 |
| **Data analysis** | | |
| **9** | Provide a conceptual overview of the data analysis method. A diagram may be helpful. | 6 |
| **10** | Provide a detailed description of all steps of the analysis, including mathematical  formulae. This description should cover, as relevant, data cleaning, data pre-processing, data adjustments and weighting of data sources, and mathematical or statistical  model(s). | 6 |
| **11** | Describe how candidate models were evaluated and how the final model(s) were selected. | 6-7 |
| **12** | Provide the results of an evaluation of model performance, if done, as well as the results of any relevant sensitivity analysis. | 6-7 |
| **13** | Describe methods for calculating uncertainty of the estimates. State which sources of uncertainty were, and were not, accounted for in the uncertainty analysis. | 6-7 |
| **14** | State how analytic or statistical source code used to generate estimates can be accessed. |  |
| **Results and Discussion** | | |
| **15** | Provide published estimates in a file format from which data can be efficiently extracted. | 7-11 |
| **16** | Report a quantitative measure of the uncertainty of the estimates (e.g. uncertainty intervals). | 7-11 |
| **17** | Interpret results in light of existing evidence. If updating a previous set of estimates, describe the reasons for changes in estimates. | 7-11 |
| **18** | Discuss limitations of the estimates. Include a discussion of any modelling assumptions or data limitations that affect interpretation of the estimates. | 11-16 |

*This checklist should be used in conjunction with the GATHER statement and Explanation and Elaboration document, found on gather-statement.org*

# TableS2 204 Countries of Heart Failure Attributable to Atrial Fibrillation and Flutter From 1990 to 2021: Prevalence, Years Lived With Disability (YLDs), Age-Standardized Rates, and Estimated Annual Percentage Changes (EAPC)

| **Location** | **Prevalence** | | | |  | **YLDs(years lived with disability rates)** | | | | **Location** |
| --- | --- | --- | --- | --- | --- | --- | --- | --- | --- | --- |
|  | **Number of cases-1990** | **ASPR-1990** | **Number of cases-2021** | **ASPR-2021** | **EAPC(95%CI)** | **Number of cases-1990** | **ASPR-1990** | **Number of cases-2021** | **ASPR-2021** | **EAPC(95%CI)** |
| Indonesia | 2398 (1823 to 3106) | 4.41 (3.22 to 5.78) | 7544 (5478 to 10296) | 5.84 (4.04 to 7.91) | 0.76 (0.7 to 0.82) | 213 (136 to 325) | 0.39 (0.24 to 0.58) | 675 (410 to 1055) | 0.51 (0.3 to 0.78) | 0.78 (0.72 to 0.84) |
| Lao People's Democratic Republic | 28 (21 to 37) | 2.84 (2.06 to 3.84) | 114 (84 to 154) | 4.12 (2.9 to 5.64) | 1.27 (1.25 to 1.3) | 3 (1 to 4) | 0.25 (0.15 to 0.39) | 10 (6 to 16) | 0.37 (0.21 to 0.56) | 1.28 (1.25 to 1.31) |
| China | 21375 (15718 to 29223) | 4.51 (3.33 to 6.03) | 125841 (88459 to 171262) | 6.9 (4.8 to 9.35) | 1.19 (1.04 to 1.34) | 1927 (1163 to 3082) | 0.4 (0.25 to 0.61) | 11251 (6661 to 17770) | 0.61 (0.36 to 0.95) | 1.21 (1.06 to 1.35) |
| Malaysia | 341 (259 to 456) | 4.62 (3.47 to 6.22) | 1663 (1237 to 2210) | 8.09 (5.88 to 10.93) | 2.06 (1.98 to 2.14) | 30 (19 to 44) | 0.4 (0.25 to 0.6) | 148 (91 to 223) | 0.71 (0.45 to 1.08) | 2.08 (2 to 2.16) |
| Maldives | 1 (1 to 2) | 3.74 (2.74 to 5.11) | 17 (13 to 22) | 6.75 (4.9 to 8.99) | 2 (1.89 to 2.11) | 0 (0 to 0) | 0.33 (0.2 to 0.49) | 2 (1 to 2) | 0.6 (0.37 to 0.92) | 2.03 (1.91 to 2.14) |
| Democratic People's Republic of Korea | 427 (321 to 563) | 4.31 (3.22 to 5.67) | 1383 (1023 to 1867) | 5.16 (3.8 to 6.95) | 0.58 (0.52 to 0.64) | 38 (23 to 57) | 0.38 (0.23 to 0.57) | 124 (70 to 189) | 0.46 (0.27 to 0.69) | 0.6 (0.53 to 0.67) |
| Myanmar | 357 (271 to 474) | 2.91 (2.13 to 3.99) | 1545 (1139 to 2133) | 4.56 (3.25 to 6.19) | 1.61 (1.53 to 1.69) | 32 (20 to 47) | 0.25 (0.16 to 0.38) | 137 (80 to 210) | 0.4 (0.23 to 0.62) | 1.64 (1.56 to 1.73) |
| Taiwan (Province of China) | 738 (554 to 970) | 7.34 (5.48 to 9.86) | 5742 (4164 to 7592) | 12.86 (9.39 to 17.07) | 1.4 (0.94 to 1.87) | 66 (40 to 101) | 0.65 (0.41 to 0.98) | 515 (315 to 778) | 1.16 (0.7 to 1.76) | 1.41 (0.94 to 1.88) |
| Philippines | 735 (547 to 985) | 4.26 (3.07 to 5.71) | 2815 (1949 to 3849) | 5.2 (3.5 to 7.23) | 0.59 (0.54 to 0.64) | 66 (40 to 103) | 0.37 (0.23 to 0.56) | 250 (148 to 392) | 0.46 (0.27 to 0.7) | 0.59 (0.54 to 0.64) |
| Cambodia | 73 (54 to 97) | 3.15 (2.26 to 4.26) | 313 (234 to 415) | 4.33 (3.13 to 5.92) | 1.12 (1.07 to 1.18) | 7 (4 to 10) | 0.27 (0.17 to 0.41) | 28 (16 to 42) | 0.38 (0.22 to 0.57) | 1.14 (1.09 to 1.19) |
| Thailand | 2592 (1966 to 3369) | 11.07 (8.2 to 14.8) | 18838 (14107 to 25217) | 17.37 (12.99 to 23.31) | 1.5 (1.43 to 1.56) | 227 (142 to 342) | 0.96 (0.61 to 1.43) | 1654 (1002 to 2445) | 1.53 (0.92 to 2.27) | 1.52 (1.45 to 1.58) |
| Micronesia (Federated States of) | 2 (1 to 2) | 4.7 (3.44 to 6.39) | 2 (2 to 3) | 5.88 (4.16 to 8.22) | 0.72 (0.67 to 0.76) | 0 (0 to 0) | 0.42 (0.24 to 0.65) | 0 (0 to 0) | 0.52 (0.31 to 0.82) | 0.72 (0.67 to 0.77) |
| Timor-Leste | 5 (3 to 6) | 3.24 (2.33 to 4.42) | 21 (16 to 29) | 3.86 (2.73 to 5.32) | 0.41 (0.36 to 0.47) | 0 (0 to 1) | 0.28 (0.17 to 0.42) | 2 (1 to 3) | 0.34 (0.2 to 0.51) | 0.44 (0.38 to 0.49) |
| Papua New Guinea | 34 (25 to 45) | 3.63 (2.67 to 4.84) | 129 (97 to 171) | 4.34 (3.13 to 5.94) | 0.49 (0.43 to 0.56) | 3 (2 to 5) | 0.32 (0.2 to 0.49) | 12 (7 to 18) | 0.38 (0.22 to 0.59) | 0.48 (0.42 to 0.55) |
| Viet Nam | 1462 (1077 to 1947) | 4.71 (3.44 to 6.35) | 5423 (3995 to 7107) | 7.32 (5.26 to 9.68) | 1.48 (1.45 to 1.51) | 129 (78 to 188) | 0.41 (0.25 to 0.6) | 480 (290 to 734) | 0.64 (0.39 to 1) | 1.49 (1.46 to 1.53) |
| Sri Lanka | 204 (153 to 268) | 3.05 (2.27 to 4.11) | 1295 (957 to 1722) | 6.06 (4.39 to 8.15) | 2.64 (2.49 to 2.79) | 18 (11 to 27) | 0.27 (0.16 to 0.4) | 115 (69 to 172) | 0.53 (0.33 to 0.79) | 2.63 (2.48 to 2.77) |
| Samoa | 3 (2 to 4) | 5.4 (3.88 to 7.36) | 7 (5 to 9) | 6.23 (4.52 to 8.49) | 0.4 (0.34 to 0.45) | 0 (0 to 0) | 0.48 (0.29 to 0.71) | 1 (0 to 1) | 0.55 (0.32 to 0.84) | 0.38 (0.32 to 0.44) |
| Solomon Islands | 2 (1 to 2) | 2.67 (1.92 to 3.61) | 7 (5 to 10) | 3.33 (2.34 to 4.65) | 0.75 (0.71 to 0.79) | 0 (0 to 0) | 0.24 (0.14 to 0.36) | 1 (0 to 1) | 0.3 (0.16 to 0.46) | 0.74 (0.71 to 0.78) |
| Fiji | 9 (7 to 11) | 4.27 (3.16 to 5.66) | 26 (20 to 36) | 5.75 (4.11 to 7.93) | 1.2 (1.09 to 1.31) | 1 (0 to 1) | 0.38 (0.23 to 0.58) | 2 (1 to 4) | 0.51 (0.3 to 0.78) | 1.18 (1.08 to 1.29) |
| Tonga | 3 (2 to 4) | 7.79 (5.82 to 10.37) | 7 (5 to 10) | 9.83 (7.09 to 13.38) | 0.69 (0.66 to 0.73) | 0 (0 to 0) | 0.69 (0.42 to 1.05) | 1 (0 to 1) | 0.87 (0.51 to 1.37) | 0.7 (0.66 to 0.73) |
| Kiribati | 1 (1 to 1) | 3.57 (2.53 to 4.88) | 2 (1 to 2) | 4.23 (2.96 to 5.83) | 0.49 (0.44 to 0.54) | 0 (0 to 0) | 0.32 (0.19 to 0.5) | 0 (0 to 0) | 0.37 (0.21 to 0.58) | 0.49 (0.43 to 0.54) |
| Marshall Islands | 0 (0 to 1) | 4.21 (3.03 to 5.7) | 1 (1 to 1) | 5.15 (3.69 to 7.13) | 0.59 (0.55 to 0.63) | 0 (0 to 0) | 0.37 (0.21 to 0.56) | 0 (0 to 0) | 0.46 (0.28 to 0.7) | 0.6 (0.55 to 0.65) |
| Vanuatu | 1 (1 to 2) | 3.38 (2.48 to 4.58) | 4 (3 to 6) | 3.99 (2.87 to 5.5) | 0.56 (0.53 to 0.6) | 0 (0 to 0) | 0.3 (0.18 to 0.45) | 0 (0 to 1) | 0.36 (0.2 to 0.55) | 0.55 (0.51 to 0.58) |
| Armenia | 28 (20 to 39) | 1.29 (0.92 to 1.77) | 129 (95 to 175) | 2.96 (2.16 to 4) | 3.75 (3.29 to 4.21) | 3 (1 to 4) | 0.12 (0.07 to 0.18) | 12 (7 to 18) | 0.27 (0.15 to 0.43) | 3.69 (3.24 to 4.15) |
| Tajikistan | 21 (14 to 29) | 0.91 (0.6 to 1.29) | 35 (25 to 49) | 0.87 (0.59 to 1.23) | 0.17 (0.02 to 0.33) | 2 (1 to 3) | 0.08 (0.04 to 0.13) | 3 (2 to 5) | 0.08 (0.05 to 0.12) | 0.19 (0.03 to 0.34) |
| Turkmenistan | 22 (15 to 31) | 1.58 (1.06 to 2.22) | 64 (44 to 87) | 2.13 (1.42 to 2.97) | 1.66 (1.35 to 1.97) | 2 (1 to 3) | 0.14 (0.08 to 0.24) | 6 (3 to 9) | 0.19 (0.11 to 0.31) | 1.64 (1.33 to 1.94) |
| Azerbaijan | 43 (30 to 60) | 1.1 (0.75 to 1.52) | 105 (72 to 148) | 1.42 (0.95 to 2.03) | 1.21 (1.03 to 1.4) | 4 (2 to 6) | 0.1 (0.05 to 0.16) | 10 (5 to 15) | 0.13 (0.07 to 0.21) | 1.2 (1.02 to 1.38) |
| Georgia | 147 (99 to 205) | 2.65 (1.79 to 3.72) | 229 (102 to 354) | 3.56 (1.6 to 5.49) | 0.58 (-0.16 to 1.34) | 13 (7 to 20) | 0.24 (0.14 to 0.37) | 21 (8 to 37) | 0.32 (0.13 to 0.58) | 0.63 (-0.11 to 1.37) |
| Uzbekistan | 92 (63 to 128) | 0.91 (0.61 to 1.27) | 207 (134 to 290) | 1.08 (0.67 to 1.54) | 1.02 (0.52 to 1.51) | 9 (5 to 14) | 0.08 (0.05 to 0.13) | 19 (10 to 30) | 0.1 (0.05 to 0.16) | 1.02 (0.53 to 1.52) |
| Kazakhstan | 192 (132 to 264) | 1.87 (1.29 to 2.64) | 305 (211 to 428) | 2.22 (1.5 to 3.09) | 0.8 (0.33 to 1.26) | 18 (10 to 28) | 0.17 (0.1 to 0.27) | 28 (15 to 44) | 0.2 (0.11 to 0.32) | 0.8 (0.34 to 1.26) |
| Kyrgyzstan | 48 (34 to 66) | 1.91 (1.34 to 2.64) | 76 (55 to 100) | 2.08 (1.48 to 2.8) | 0.68 (0.3 to 1.06) | 4 (2 to 7) | 0.17 (0.1 to 0.27) | 7 (4 to 11) | 0.19 (0.11 to 0.29) | 0.69 (0.32 to 1.07) |
| Albania | 63 (45 to 85) | 3.86 (2.71 to 5.26) | 241 (168 to 335) | 5.58 (3.93 to 7.66) | 1.29 (1.14 to 1.45) | 6 (3 to 9) | 0.35 (0.21 to 0.53) | 22 (12 to 37) | 0.5 (0.27 to 0.84) | 1.31 (1.16 to 1.46) |
| Mongolia | 11 (7 to 16) | 1.33 (0.85 to 1.91) | 30 (19 to 43) | 1.9 (1.16 to 2.78) | 1.31 (1.09 to 1.52) | 1 (1 to 2) | 0.12 (0.07 to 0.19) | 3 (1 to 5) | 0.17 (0.09 to 0.29) | 1.3 (1.09 to 1.5) |
| Czechia | 584 (402 to 816) | 4.46 (3.14 to 6.2) | 2508 (1652 to 3617) | 10.36 (6.95 to 14.71) | 3.51 (3.14 to 3.88) | 52 (29 to 83) | 0.4 (0.22 to 0.61) | 225 (121 to 380) | 0.93 (0.51 to 1.56) | 3.52 (3.15 to 3.89) |
| Bosnia and Herzegovina | 97 (67 to 134) | 3.2 (2.18 to 4.46) | 291 (191 to 417) | 4.5 (2.96 to 6.39) | 1.61 (1.41 to 1.8) | 9 (5 to 14) | 0.29 (0.16 to 0.45) | 26 (14 to 43) | 0.41 (0.22 to 0.67) | 1.58 (1.39 to 1.77) |
| Slovakia | 283 (204 to 378) | 5.1 (3.71 to 6.79) | 817 (574 to 1173) | 8.37 (5.92 to 11.92) | 2.29 (2.04 to 2.54) | 25 (15 to 39) | 0.46 (0.28 to 0.71) | 74 (43 to 120) | 0.75 (0.45 to 1.21) | 2.3 (2.05 to 2.54) |
| Hungary | 349 (225 to 513) | 2.56 (1.67 to 3.68) | 794 (500 to 1159) | 3.57 (2.27 to 5.16) | 0.98 (0.84 to 1.12) | 32 (16 to 53) | 0.23 (0.13 to 0.38) | 72 (38 to 119) | 0.33 (0.17 to 0.53) | 0.98 (0.84 to 1.12) |
| North Macedonia | 45 (32 to 60) | 2.93 (2.04 to 4.02) | 109 (74 to 158) | 3.94 (2.69 to 5.62) | 1.34 (1.24 to 1.44) | 4 (2 to 6) | 0.27 (0.15 to 0.41) | 10 (5 to 17) | 0.36 (0.19 to 0.6) | 1.33 (1.23 to 1.43) |
| Bulgaria | 273 (185 to 373) | 2.59 (1.83 to 3.47) | 438 (288 to 641) | 2.76 (1.85 to 3.96) | 0.47 (0.11 to 0.83) | 25 (14 to 39) | 0.24 (0.14 to 0.36) | 40 (22 to 65) | 0.25 (0.14 to 0.4) | 0.45 (0.09 to 0.8) |
| Montenegro | 73 (52 to 99) | 12.85 (9.17 to 17.74) | 128 (88 to 176) | 13.74 (9.66 to 18.92) | 0.52 (0.38 to 0.66) | 7 (4 to 10) | 1.15 (0.65 to 1.82) | 12 (6 to 18) | 1.24 (0.7 to 1.94) | 0.52 (0.38 to 0.67) |
| Slovenia | 69 (43 to 98) | 2.89 (1.81 to 4.15) | 349 (216 to 511) | 6.85 (4.25 to 9.86) | 3.57 (3.21 to 3.94) | 6 (3 to 10) | 0.26 (0.13 to 0.43) | 32 (17 to 51) | 0.62 (0.33 to 1.02) | 3.56 (3.21 to 3.92) |
| Poland | 3694 (2718 to 4942) | 9.39 (6.96 to 12.36) | 10936 (8131 to 15232) | 13.88 (10.32 to 19.37) | 2.18 (1.58 to 2.8) | 332 (199 to 500) | 0.84 (0.5 to 1.28) | 979 (585 to 1530) | 1.24 (0.74 to 1.95) | 2.19 (1.58 to 2.8) |
| Croatia | 89 (58 to 125) | 1.72 (1.12 to 2.44) | 316 (203 to 454) | 3.09 (2.02 to 4.37) | 1.74 (1.05 to 2.43) | 8 (4 to 13) | 0.16 (0.08 to 0.24) | 29 (15 to 48) | 0.28 (0.15 to 0.47) | 1.71 (1.04 to 2.38) |
| Romania | 367 (230 to 533) | 1.58 (0.99 to 2.3) | 946 (559 to 1400) | 2.25 (1.35 to 3.3) | 1.4 (1.18 to 1.62) | 34 (17 to 53) | 0.14 (0.08 to 0.22) | 86 (43 to 147) | 0.2 (0.1 to 0.35) | 1.4 (1.19 to 1.61) |
| Belarus | 363 (254 to 496) | 3.01 (2.1 to 4.14) | 707 (493 to 970) | 4.25 (2.95 to 5.86) | 1.34 (1.05 to 1.62) | 33 (18 to 53) | 0.27 (0.15 to 0.43) | 64 (34 to 102) | 0.38 (0.21 to 0.63) | 1.35 (1.06 to 1.63) |
| Serbia | 192 (117 to 271) | 2.21 (1.29 to 3.22) | 513 (291 to 757) | 2.91 (1.69 to 4.22) | -0.05 (-0.72 to 0.62) | 17 (9 to 28) | 0.2 (0.11 to 0.32) | 47 (22 to 79) | 0.27 (0.13 to 0.45) | -0.02 (-0.68 to 0.64) |
| Republic of Moldova | 82 (58 to 115) | 2.44 (1.72 to 3.37) | 265 (170 to 386) | 4.33 (2.77 to 6.34) | 1.76 (1.54 to 1.98) | 7 (4 to 12) | 0.22 (0.13 to 0.34) | 24 (13 to 41) | 0.39 (0.21 to 0.67) | 1.74 (1.52 to 1.96) |
| Estonia | 63 (44 to 87) | 3.23 (2.25 to 4.49) | 193 (114 to 288) | 5.81 (3.5 to 8.7) | 1.38 (1.14 to 1.63) | 6 (3 to 9) | 0.29 (0.17 to 0.46) | 17 (9 to 31) | 0.52 (0.26 to 0.92) | 1.4 (1.15 to 1.64) |
| Russian Federation | 5492 (3914 to 7557) | 3.66 (2.63 to 4.98) | 12870 (8582 to 18171) | 5.22 (3.5 to 7.42) | 1.63 (1.26 to 2.01) | 494 (291 to 785) | 0.33 (0.19 to 0.5) | 1150 (659 to 1910) | 0.47 (0.27 to 0.77) | 1.62 (1.25 to 1.99) |
| Republic of Korea | 816 (597 to 1076) | 3.71 (2.53 to 5.03) | 12846 (9895 to 16485) | 13.77 (10.62 to 17.6) | 5.15 (4.76 to 5.55) | 75 (45 to 110) | 0.33 (0.2 to 0.51) | 1157 (730 to 1679) | 1.24 (0.79 to 1.79) | 5.13 (4.74 to 5.53) |
| Ukraine | 1998 (1406 to 2799) | 3.12 (2.26 to 4.26) | 2549 (1686 to 3689) | 3.12 (2.08 to 4.47) | 0.15 (-0.11 to 0.41) | 180 (99 to 296) | 0.28 (0.16 to 0.46) | 230 (124 to 387) | 0.28 (0.15 to 0.47) | 0.16 (-0.1 to 0.42) |
| Latvia | 114 (78 to 161) | 3.29 (2.26 to 4.59) | 245 (157 to 364) | 5.04 (3.27 to 7.42) | 1.82 (1.54 to 2.1) | 10 (6 to 17) | 0.3 (0.17 to 0.48) | 22 (11 to 37) | 0.45 (0.23 to 0.76) | 1.79 (1.52 to 2.07) |
| Singapore | 28 (19 to 38) | 1.69 (1.08 to 2.4) | 333 (233 to 454) | 4.02 (2.81 to 5.54) | 2.43 (2.22 to 2.65) | 3 (1 to 4) | 0.15 (0.09 to 0.24) | 30 (18 to 47) | 0.36 (0.21 to 0.57) | 2.44 (2.23 to 2.65) |
| Lithuania | 152 (106 to 217) | 3.43 (2.41 to 4.81) | 446 (290 to 639) | 6.46 (4.25 to 9.16) | 2.25 (2.01 to 2.48) | 14 (7 to 23) | 0.31 (0.17 to 0.5) | 40 (22 to 66) | 0.58 (0.31 to 0.96) | 2.24 (2.01 to 2.46) |
| Brunei Darussalam | 3 (2 to 4) | 3.15 (1.68 to 4.7) | 12 (7 to 17) | 4.62 (2.5 to 6.84) | 1.15 (1.08 to 1.22) | 0 (0 to 0) | 0.28 (0.13 to 0.46) | 1 (1 to 2) | 0.42 (0.21 to 0.69) | 1.13 (1.06 to 1.21) |
| Australia | 2234 (1581 to 3089) | 11.97 (8.46 to 16.46) | 13946 (10226 to 18780) | 26.87 (19.81 to 35.97) | 2.95 (2.51 to 3.39) | 201 (121 to 310) | 1.07 (0.63 to 1.64) | 1248 (732 to 1847) | 2.41 (1.41 to 3.59) | 2.96 (2.51 to 3.4) |
| Austria | 1016 (750 to 1331) | 8.23 (6.16 to 10.61) | 2983 (2041 to 3936) | 13.54 (9.41 to 18.13) | 1.29 (0.9 to 1.69) | 92 (55 to 140) | 0.74 (0.44 to 1.12) | 268 (152 to 413) | 1.22 (0.71 to 1.86) | 1.3 (0.91 to 1.69) |
| Japan | 6877 (4550 to 9294) | 4.45 (2.96 to 5.98) | 35991 (23876 to 49261) | 7.44 (5.17 to 9.91) | 1.08 (0.68 to 1.48) | 627 (345 to 1002) | 0.4 (0.23 to 0.62) | 3231 (1857 to 5021) | 0.67 (0.39 to 1.07) | 1.07 (0.68 to 1.47) |
| Belgium | 808 (547 to 1118) | 5.15 (3.59 to 7.01) | 2539 (1851 to 3467) | 8.71 (6.49 to 11.6) | 2.7 (2.4 to 3.01) | 73 (40 to 114) | 0.46 (0.27 to 0.72) | 229 (140 to 340) | 0.79 (0.48 to 1.16) | 2.71 (2.41 to 3.01) |
| New Zealand | 443 (298 to 616) | 11.79 (7.91 to 16.38) | 1522 (1032 to 2158) | 16.3 (11.11 to 23.07) | 1.56 (1.39 to 1.72) | 40 (23 to 61) | 1.06 (0.61 to 1.59) | 137 (77 to 223) | 1.46 (0.83 to 2.39) | 1.56 (1.39 to 1.72) |
| Cyprus | 32 (20 to 46) | 5.1 (3.23 to 7.32) | 162 (110 to 231) | 7.85 (5.34 to 10.83) | 1.36 (1.15 to 1.57) | 3 (1 to 5) | 0.46 (0.25 to 0.74) | 15 (8 to 23) | 0.71 (0.42 to 1.12) | 1.36 (1.15 to 1.57) |
| Greece | 596 (420 to 797) | 4.14 (2.92 to 5.56) | 1192 (781 to 1671) | 3.82 (2.59 to 5.26) | -1.02 (-1.37 to -0.67) | 54 (31 to 83) | 0.37 (0.22 to 0.57) | 108 (59 to 170) | 0.35 (0.2 to 0.54) | -0.98 (-1.33 to -0.63) |
| Denmark | 378 (256 to 518) | 4.34 (2.98 to 5.84) | 1812 (1205 to 2549) | 13.23 (8.95 to 18.5) | 4.78 (4.2 to 5.37) | 34 (20 to 54) | 0.39 (0.23 to 0.62) | 164 (94 to 265) | 1.2 (0.68 to 1.95) | 4.8 (4.21 to 5.39) |
| Iceland | 11 (6 to 15) | 3.49 (2.11 to 4.99) | 55 (34 to 77) | 8.34 (5.35 to 11.76) | 3.03 (2.68 to 3.37) | 1 (1 to 2) | 0.32 (0.17 to 0.49) | 5 (3 to 8) | 0.75 (0.42 to 1.21) | 3.03 (2.68 to 3.37) |
| Finland | 602 (377 to 823) | 8.42 (5.4 to 11.33) | 1615 (1070 to 2297) | 10.46 (7.04 to 14.68) | 0.73 (0.04 to 1.44) | 54 (29 to 86) | 0.75 (0.43 to 1.18) | 145 (77 to 236) | 0.94 (0.51 to 1.53) | 0.75 (0.06 to 1.45) |
| Andorra | 3 (2 to 5) | 7.19 (5.12 to 9.8) | 17 (12 to 23) | 10.06 (7.03 to 13.78) | 1.2 (1.07 to 1.34) | 0 (0 to 0) | 0.65 (0.38 to 1.01) | 2 (1 to 2) | 0.91 (0.52 to 1.43) | 1.21 (1.08 to 1.35) |
| Ireland | 199 (136 to 277) | 5.1 (3.52 to 6.82) | 1088 (748 to 1510) | 12.66 (8.77 to 17.5) | 3.46 (3.18 to 3.73) | 18 (10 to 28) | 0.46 (0.27 to 0.7) | 98 (59 to 157) | 1.14 (0.69 to 1.81) | 3.43 (3.16 to 3.71) |
| France | 16747 (12236 to 22438) | 18.49 (13.83 to 24.36) | 67506 (48194 to 92094) | 38.17 (27.77 to 51.34) | 2.49 (2.33 to 2.66) | 1503 (911 to 2348) | 1.66 (1.01 to 2.55) | 6061 (3754 to 9343) | 3.44 (2.1 to 5.29) | 2.52 (2.36 to 2.69) |
| Israel | 253 (162 to 364) | 5.64 (3.76 to 7.99) | 1966 (1375 to 2660) | 14.18 (9.99 to 19.24) | 3.54 (3.26 to 3.82) | 23 (13 to 37) | 0.51 (0.3 to 0.77) | 177 (106 to 270) | 1.28 (0.76 to 1.95) | 3.54 (3.27 to 3.82) |
| Netherlands | 1855 (1373 to 2437) | 8.95 (6.65 to 11.62) | 6466 (4316 to 9105) | 16.23 (10.97 to 22.82) | 2.41 (2.17 to 2.65) | 168 (100 to 258) | 0.81 (0.49 to 1.24) | 584 (325 to 937) | 1.47 (0.82 to 2.35) | 2.42 (2.18 to 2.66) |
| Germany | 8416 (5835 to 11426) | 6.42 (4.54 to 8.63) | 37474 (24502 to 52708) | 15.47 (10.35 to 21.49) | 4.41 (3.76 to 5.07) | 760 (427 to 1187) | 0.58 (0.33 to 0.9) | 3371 (1953 to 5353) | 1.39 (0.82 to 2.21) | 4.42 (3.77 to 5.08) |
| Norway | 589 (330 to 882) | 7.74 (4.47 to 11.36) | 1727 (1036 to 2513) | 14.7 (8.91 to 21.17) | 2.55 (2.24 to 2.87) | 53 (26 to 89) | 0.7 (0.36 to 1.15) | 156 (82 to 260) | 1.33 (0.71 to 2.23) | 2.58 (2.26 to 2.89) |
| Italy | 4355 (3007 to 6046) | 4.96 (3.48 to 6.69) | 22179 (15413 to 31187) | 12.33 (8.77 to 17.34) | 3.78 (3.22 to 4.35) | 394 (226 to 632) | 0.45 (0.26 to 0.71) | 1994 (1182 to 3138) | 1.11 (0.66 to 1.75) | 3.78 (3.22 to 4.35) |
| Portugal | 662 (497 to 863) | 5.31 (3.97 to 6.94) | 2890 (2024 to 3948) | 9.48 (6.81 to 12.86) | 2.3 (2.14 to 2.46) | 60 (33 to 95) | 0.48 (0.28 to 0.73) | 260 (157 to 408) | 0.86 (0.52 to 1.34) | 2.31 (2.15 to 2.47) |
| Spain | 3672 (2538 to 4963) | 6.84 (4.77 to 9.22) | 15941 (12294 to 20358) | 12.91 (10.07 to 16.65) | 1.91 (1.78 to 2.04) | 330 (196 to 511) | 0.61 (0.37 to 0.94) | 1428 (935 to 2117) | 1.16 (0.76 to 1.72) | 1.91 (1.78 to 2.05) |
| Argentina | 753 (521 to 1023) | 2.8 (1.9 to 3.83) | 2522 (1623 to 3536) | 4.27 (2.77 to 5.97) | 1.69 (1.4 to 1.98) | 68 (38 to 106) | 0.25 (0.14 to 0.39) | 227 (127 to 372) | 0.39 (0.22 to 0.63) | 1.7 (1.4 to 1.99) |
| Luxembourg | 37 (22 to 52) | 6.93 (4.33 to 9.7) | 195 (131 to 275) | 16.41 (11.04 to 23.03) | 2.97 (2.36 to 3.59) | 3 (2 to 5) | 0.62 (0.32 to 0.99) | 18 (10 to 28) | 1.48 (0.83 to 2.37) | 2.98 (2.37 to 3.6) |
| Sweden | 2186 (1544 to 2994) | 12.82 (9.11 to 17.38) | 10582 (6874 to 15052) | 38.94 (25.81 to 55.09) | 3.89 (3.51 to 4.28) | 198 (113 to 316) | 1.16 (0.67 to 1.82) | 956 (534 to 1531) | 3.53 (1.98 to 5.68) | 3.9 (3.51 to 4.29) |
| Malta | 19 (14 to 26) | 4.96 (3.6 to 6.87) | 114 (78 to 160) | 9.97 (6.92 to 13.77) | 3.47 (3.09 to 3.85) | 2 (1 to 3) | 0.45 (0.27 to 0.69) | 10 (6 to 16) | 0.9 (0.52 to 1.42) | 3.46 (3.09 to 3.84) |
| Chile | 342 (238 to 463) | 4.08 (2.85 to 5.49) | 2111 (1445 to 2931) | 7.98 (5.49 to 11.06) | 2.99 (2.61 to 3.37) | 31 (18 to 48) | 0.37 (0.21 to 0.58) | 190 (105 to 300) | 0.72 (0.4 to 1.14) | 2.99 (2.61 to 3.37) |
| Switzerland | 299 (188 to 431) | 2.61 (1.68 to 3.69) | 1399 (930 to 1967) | 6.43 (4.4 to 8.95) | 4.19 (3.54 to 4.83) | 27 (15 to 43) | 0.24 (0.13 to 0.37) | 127 (71 to 207) | 0.58 (0.33 to 0.95) | 4.18 (3.54 to 4.82) |
| Uruguay | 118 (81 to 165) | 3.14 (2.17 to 4.4) | 340 (208 to 491) | 5.08 (3.16 to 7.28) | 1.96 (1.78 to 2.15) | 11 (6 to 17) | 0.28 (0.16 to 0.44) | 31 (16 to 50) | 0.46 (0.24 to 0.75) | 1.95 (1.77 to 2.13) |
| United Kingdom | 4468 (2948 to 6298) | 4.65 (3.1 to 6.42) | 15389 (10110 to 21510) | 9.78 (6.49 to 13.56) | 2.28 (2.18 to 2.38) | 403 (223 to 656) | 0.42 (0.24 to 0.66) | 1381 (762 to 2191) | 0.88 (0.49 to 1.39) | 2.28 (2.18 to 2.38) |
| Antigua and Barbuda | 5 (4 to 6) | 8.41 (6.39 to 10.53) | 6 (5 to 9) | 7.38 (5.37 to 9.99) | -0.42 (-0.67 to -0.17) | 0 (0 to 1) | 0.75 (0.46 to 1.11) | 1 (0 to 1) | 0.66 (0.38 to 1.02) | -0.42 (-0.68 to -0.17) |
| Bahamas | 7 (5 to 9) | 6.03 (4.45 to 7.91) | 20 (14 to 27) | 6.3 (4.45 to 8.65) | 0.1 (-0.04 to 0.23) | 1 (0 to 1) | 0.54 (0.32 to 0.8) | 2 (1 to 3) | 0.56 (0.33 to 0.85) | 0.1 (-0.03 to 0.24) |
| Dominican Republic | 207 (156 to 267) | 7.62 (5.76 to 9.94) | 795 (567 to 1118) | 8.39 (5.98 to 11.82) | -0.15 (-0.25 to -0.05) | 18 (11 to 28) | 0.67 (0.41 to 1.01) | 71 (42 to 110) | 0.75 (0.45 to 1.16) | -0.15 (-0.26 to -0.05) |
| Canada | 3309 (2406 to 4497) | 10.11 (7.4 to 13.67) | 16703 (11768 to 22871) | 20.52 (14.42 to 28.11) | 2.44 (2.05 to 2.83) | 299 (174 to 453) | 0.91 (0.54 to 1.37) | 1510 (898 to 2367) | 1.86 (1.12 to 2.93) | 2.42 (2.04 to 2.81) |
| Grenada | 6 (4 to 8) | 6.56 (4.94 to 8.88) | 7 (5 to 9) | 7.76 (5.62 to 10.62) | 0.37 (0.13 to 0.6) | 1 (0 to 1) | 0.58 (0.34 to 0.87) | 1 (0 to 1) | 0.7 (0.41 to 1.07) | 0.37 (0.14 to 0.61) |
| United States of America | 21419 (14461 to 30079) | 6.32 (4.36 to 8.69) | 75742 (52555 to 105360) | 11.81 (8.23 to 16.52) | 2.21 (2.07 to 2.34) | 1933 (1085 to 3115) | 0.57 (0.33 to 0.92) | 6823 (4027 to 10590) | 1.07 (0.64 to 1.67) | 2.19 (2.06 to 2.33) |
| Barbados | 27 (20 to 36) | 8.37 (6.37 to 10.95) | 58 (42 to 79) | 11.17 (8.16 to 15.18) | 0.91 (0.68 to 1.13) | 2 (1 to 4) | 0.75 (0.46 to 1.14) | 5 (3 to 8) | 1 (0.58 to 1.5) | 0.91 (0.68 to 1.14) |
| Guyana | 8 (6 to 10) | 2.91 (2.15 to 3.96) | 20 (15 to 27) | 4.51 (3.27 to 6.13) | 1.06 (0.86 to 1.27) | 1 (0 to 1) | 0.26 (0.15 to 0.39) | 2 (1 to 3) | 0.4 (0.25 to 0.63) | 1.06 (0.85 to 1.27) |
| Belize | 7 (5 to 9) | 7.8 (5.93 to 10.32) | 19 (14 to 25) | 7.78 (5.67 to 10.54) | -0.01 (-0.15 to 0.13) | 1 (0 to 1) | 0.69 (0.42 to 1.02) | 2 (1 to 3) | 0.7 (0.41 to 1.09) | 0 (-0.14 to 0.15) |
| Cuba | 715 (533 to 950) | 7.5 (5.65 to 10.03) | 2032 (1472 to 2717) | 9.46 (6.91 to 12.65) | 0.83 (0.68 to 0.97) | 64 (38 to 98) | 0.67 (0.41 to 1.01) | 181 (107 to 272) | 0.84 (0.5 to 1.27) | 0.83 (0.68 to 0.98) |
| Haiti | 74 (55 to 98) | 4.26 (3.2 to 5.65) | 221 (168 to 293) | 5.45 (4.03 to 7.42) | 0.81 (0.71 to 0.9) | 7 (4 to 10) | 0.38 (0.23 to 0.57) | 20 (11 to 30) | 0.48 (0.27 to 0.72) | 0.8 (0.7 to 0.89) |
| Suriname | 16 (12 to 21) | 7.53 (5.65 to 9.94) | 53 (39 to 73) | 9.63 (7.02 to 13.19) | 0.85 (0.79 to 0.92) | 1 (1 to 2) | 0.67 (0.4 to 1.02) | 5 (3 to 7) | 0.85 (0.51 to 1.31) | 0.85 (0.78 to 0.92) |
| Dominica | 3 (2 to 4) | 5.86 (4.4 to 7.68) | 5 (4 to 7) | 7.12 (5.01 to 9.66) | 0.65 (0.5 to 0.8) | 0 (0 to 0) | 0.52 (0.32 to 0.8) | 0 (0 to 1) | 0.63 (0.37 to 0.96) | 0.66 (0.51 to 0.81) |
| Trinidad and Tobago | 37 (28 to 49) | 5.65 (4.19 to 7.41) | 165 (122 to 224) | 9.3 (6.83 to 12.57) | 1.88 (1.72 to 2.03) | 3 (2 to 5) | 0.5 (0.3 to 0.76) | 15 (9 to 23) | 0.83 (0.49 to 1.28) | 1.87 (1.72 to 2.02) |
| Colombia | 706 (539 to 894) | 5.36 (4.05 to 6.98) | 5732 (4321 to 7633) | 10.19 (7.63 to 13.66) | 2.18 (2.09 to 2.28) | 63 (39 to 97) | 0.48 (0.3 to 0.73) | 510 (318 to 759) | 0.91 (0.56 to 1.37) | 2.2 (2.11 to 2.29) |
| Jamaica | 139 (106 to 180) | 7.46 (5.69 to 9.57) | 339 (251 to 451) | 9.63 (7.11 to 12.89) | 0.63 (0.28 to 0.98) | 12 (7 to 19) | 0.66 (0.4 to 1.02) | 30 (19 to 45) | 0.85 (0.52 to 1.28) | 0.63 (0.28 to 0.98) |
| Bolivia (Plurinational State of) | 136 (102 to 179) | 6.09 (4.58 to 7.93) | 625 (465 to 830) | 8.82 (6.61 to 11.9) | 1.25 (1.17 to 1.34) | 12 (7 to 18) | 0.54 (0.33 to 0.82) | 56 (34 to 85) | 0.79 (0.49 to 1.19) | 1.27 (1.19 to 1.36) |
| Saint Lucia | 6 (4 to 7) | 7.62 (5.73 to 10.14) | 21 (15 to 28) | 9.02 (6.48 to 12.14) | 0.35 (0.13 to 0.57) | 0 (0 to 1) | 0.67 (0.4 to 1.04) | 2 (1 to 3) | 0.8 (0.48 to 1.23) | 0.35 (0.13 to 0.57) |
| Costa Rica | 113 (85 to 146) | 7.19 (5.39 to 9.37) | 634 (463 to 852) | 11.42 (8.32 to 15.34) | 1.7 (1.6 to 1.8) | 10 (6 to 15) | 0.64 (0.4 to 0.94) | 56 (34 to 87) | 1.02 (0.61 to 1.56) | 1.72 (1.62 to 1.81) |
| Saint Vincent and the Grenadines | 4 (3 to 6) | 6.53 (4.91 to 8.78) | 8 (6 to 12) | 6.68 (4.76 to 9.04) | -0.02 (-0.18 to 0.14) | 0 (0 to 1) | 0.58 (0.35 to 0.91) | 1 (0 to 1) | 0.59 (0.36 to 0.91) | -0.02 (-0.18 to 0.13) |
| Ecuador | 331 (248 to 425) | 7.6 (5.67 to 9.82) | 1221 (907 to 1677) | 7.92 (5.87 to 10.8) | -0.08 (-0.26 to 0.09) | 30 (17 to 45) | 0.68 (0.4 to 1.04) | 110 (63 to 169) | 0.71 (0.41 to 1.09) | -0.07 (-0.24 to 0.1) |
| El Salvador | 295 (224 to 383) | 10.68 (8.09 to 13.94) | 884 (655 to 1180) | 12.58 (9.37 to 16.78) | 0.63 (0.59 to 0.67) | 26 (16 to 37) | 0.94 (0.59 to 1.37) | 78 (47 to 115) | 1.11 (0.67 to 1.65) | 0.66 (0.62 to 0.7) |
| Panama | 120 (93 to 162) | 9.32 (7.18 to 12.48) | 611 (456 to 803) | 13.31 (9.86 to 17.65) | 1.24 (1.09 to 1.39) | 11 (7 to 16) | 0.82 (0.53 to 1.25) | 54 (34 to 80) | 1.18 (0.73 to 1.78) | 1.26 (1.11 to 1.41) |
| Peru | 1079 (818 to 1395) | 10.45 (7.85 to 13.59) | 4903 (3621 to 6393) | 14.83 (10.95 to 19.49) | 1.29 (1.19 to 1.39) | 96 (57 to 146) | 0.93 (0.54 to 1.42) | 437 (269 to 627) | 1.32 (0.81 to 1.92) | 1.32 (1.22 to 1.42) |
| Guatemala | 160 (119 to 215) | 7.41 (5.4 to 9.94) | 913 (672 to 1227) | 9.49 (6.92 to 12.89) | 0.72 (0.58 to 0.86) | 14 (8 to 22) | 0.65 (0.39 to 0.97) | 82 (49 to 126) | 0.84 (0.51 to 1.3) | 0.75 (0.6 to 0.89) |
| Venezuela (Bolivarian Republic of) | 385 (292 to 501) | 5.15 (3.82 to 6.76) | 1662 (1251 to 2189) | 6.19 (4.62 to 8.18) | 0.8 (0.63 to 0.97) | 34 (21 to 52) | 0.46 (0.28 to 0.68) | 149 (92 to 226) | 0.55 (0.34 to 0.86) | 0.82 (0.65 to 0.99) |
| Honduras | 70 (53 to 91) | 4.79 (3.56 to 6.29) | 260 (189 to 345) | 5.75 (4.2 to 7.63) | 0.6 (0.52 to 0.69) | 6 (4 to 9) | 0.42 (0.26 to 0.63) | 23 (14 to 35) | 0.51 (0.31 to 0.76) | 0.61 (0.52 to 0.7) |
| Algeria | 223 (166 to 290) | 2.79 (2.15 to 3.61) | 1128 (841 to 1498) | 4.15 (3.09 to 5.54) | 1.49 (1.4 to 1.58) | 20 (12 to 31) | 0.25 (0.15 to 0.37) | 102 (62 to 156) | 0.37 (0.22 to 0.57) | 1.49 (1.4 to 1.59) |
| Brazil | 3367 (2509 to 4415) | 5.9 (4.3 to 7.78) | 25424 (17640 to 34613) | 10.87 (7.46 to 14.87) | 1.88 (1.81 to 1.95) | 299 (185 to 458) | 0.52 (0.32 to 0.81) | 2253 (1340 to 3505) | 0.96 (0.57 to 1.49) | 1.89 (1.82 to 1.96) |
| Mexico | 2926 (2230 to 3797) | 8.96 (6.82 to 11.49) | 12250 (9038 to 16254) | 10.85 (7.87 to 14.42) | 0.32 (0.18 to 0.46) | 261 (163 to 395) | 0.79 (0.5 to 1.2) | 1096 (656 to 1699) | 0.97 (0.58 to 1.49) | 0.34 (0.19 to 0.48) |
| Bahrain | 6 (5 to 8) | 6.49 (4.92 to 8.6) | 68 (54 to 84) | 12.38 (9.36 to 16.12) | 2.56 (2.4 to 2.71) | 1 (0 to 1) | 0.57 (0.36 to 0.84) | 6 (4 to 9) | 1.1 (0.68 to 1.66) | 2.55 (2.4 to 2.71) |
| Nicaragua | 83 (64 to 106) | 6.85 (5.3 to 8.89) | 349 (259 to 467) | 8.32 (6.13 to 11.15) | 0.51 (0.47 to 0.55) | 7 (5 to 11) | 0.61 (0.38 to 0.92) | 31 (19 to 48) | 0.74 (0.45 to 1.13) | 0.52 (0.48 to 0.56) |
| Paraguay | 135 (101 to 175) | 7.21 (5.36 to 9.39) | 522 (386 to 697) | 10.14 (7.38 to 13.72) | 1.04 (0.96 to 1.12) | 12 (7 to 18) | 0.63 (0.4 to 0.95) | 46 (27 to 70) | 0.9 (0.52 to 1.38) | 1.06 (0.98 to 1.14) |
| Egypt | 228 (176 to 291) | 1.61 (1.21 to 2.1) | 666 (507 to 864) | 2.15 (1.55 to 2.91) | 0.81 (0.73 to 0.89) | 21 (12 to 31) | 0.14 (0.09 to 0.22) | 61 (38 to 89) | 0.19 (0.11 to 0.29) | 0.82 (0.74 to 0.9) |
| Iran (Islamic Republic of) | 455 (348 to 588) | 2.97 (2.19 to 3.9) | 2785 (2003 to 3731) | 4.33 (3.07 to 5.87) | 1.26 (1.23 to 1.3) | 41 (25 to 61) | 0.26 (0.16 to 0.4) | 249 (153 to 392) | 0.39 (0.23 to 0.62) | 1.28 (1.24 to 1.32) |
| Lebanon | 84 (65 to 107) | 5.12 (3.94 to 6.74) | 459 (344 to 591) | 6.91 (5.24 to 8.79) | 0.96 (0.87 to 1.05) | 7 (5 to 11) | 0.45 (0.27 to 0.67) | 41 (26 to 61) | 0.62 (0.39 to 0.91) | 0.99 (0.89 to 1.08) |
| Syrian Arab Republic | 101 (77 to 130) | 2.63 (1.97 to 3.45) | 288 (214 to 380) | 3.21 (2.36 to 4.28) | 0.7 (0.59 to 0.81) | 9 (6 to 13) | 0.23 (0.14 to 0.35) | 26 (16 to 39) | 0.29 (0.17 to 0.43) | 0.71 (0.6 to 0.83) |
| Libya | 57 (44 to 74) | 3.63 (2.77 to 4.79) | 172 (131 to 220) | 4.32 (3.2 to 5.68) | 0.61 (0.53 to 0.69) | 5 (3 to 8) | 0.32 (0.2 to 0.5) | 15 (10 to 23) | 0.38 (0.23 to 0.59) | 0.63 (0.55 to 0.71) |
| Morocco | 111 (84 to 143) | 1.04 (0.77 to 1.35) | 317 (237 to 423) | 1.22 (0.91 to 1.61) | 0.52 (0.42 to 0.61) | 10 (6 to 15) | 0.09 (0.06 to 0.14) | 29 (17 to 45) | 0.11 (0.07 to 0.17) | 0.52 (0.43 to 0.62) |
| Palestine | 19 (14 to 24) | 2.81 (2.15 to 3.7) | 72 (56 to 91) | 4.1 (3.12 to 5.18) | 1.32 (1.21 to 1.42) | 2 (1 to 2) | 0.25 (0.15 to 0.38) | 7 (4 to 10) | 0.37 (0.23 to 0.55) | 1.33 (1.23 to 1.44) |
| Iraq | 187 (145 to 243) | 2.62 (1.99 to 3.45) | 547 (433 to 680) | 3.22 (2.48 to 4.19) | 0.78 (0.74 to 0.82) | 17 (10 to 25) | 0.23 (0.14 to 0.35) | 50 (31 to 70) | 0.29 (0.18 to 0.42) | 0.8 (0.76 to 0.85) |
| Oman | 13 (11 to 16) | 2.75 (2.18 to 3.45) | 57 (45 to 71) | 4.37 (3.33 to 5.66) | 1.58 (1.47 to 1.69) | 1 (1 to 2) | 0.25 (0.15 to 0.37) | 5 (3 to 8) | 0.39 (0.24 to 0.58) | 1.58 (1.47 to 1.69) |
| Qatar | 6 (5 to 8) | 8.34 (6.34 to 11.09) | 95 (76 to 120) | 13.94 (10.69 to 18.23) | 2.21 (2.04 to 2.37) | 1 (0 to 1) | 0.74 (0.46 to 1.08) | 9 (6 to 13) | 1.24 (0.79 to 1.82) | 2.24 (2.08 to 2.4) |
| Kuwait | 12 (10 to 15) | 3.12 (2.34 to 4.05) | 131 (101 to 165) | 5.76 (4.33 to 7.55) | 2.71 (2.49 to 2.94) | 1 (1 to 2) | 0.28 (0.17 to 0.42) | 12 (7 to 17) | 0.52 (0.3 to 0.76) | 2.72 (2.5 to 2.94) |
| Jordan | 24 (19 to 30) | 2.75 (2.1 to 3.61) | 177 (138 to 222) | 3.54 (2.66 to 4.62) | 1.09 (0.98 to 1.21) | 2 (1 to 3) | 0.25 (0.15 to 0.38) | 16 (10 to 23) | 0.32 (0.2 to 0.48) | 1.09 (0.98 to 1.2) |
| Tunisia | 113 (85 to 146) | 3.41 (2.61 to 4.51) | 512 (387 to 658) | 4.55 (3.4 to 5.86) | 1.03 (0.99 to 1.07) | 10 (6 to 16) | 0.3 (0.18 to 0.46) | 46 (28 to 69) | 0.41 (0.25 to 0.61) | 1.04 (1 to 1.07) |
| Saudi Arabia | 84 (67 to 106) | 2.11 (1.6 to 2.74) | 303 (239 to 374) | 2.91 (2.19 to 3.73) | 1.16 (1.11 to 1.2) | 8 (5 to 11) | 0.18 (0.12 to 0.28) | 27 (18 to 40) | 0.26 (0.16 to 0.39) | 1.19 (1.15 to 1.24) |
| Turkey | 914 (715 to 1148) | 3.44 (2.65 to 4.49) | 4485 (3459 to 5778) | 5.38 (4.12 to 7.06) | 1.27 (1.18 to 1.36) | 82 (51 to 126) | 0.31 (0.19 to 0.47) | 404 (233 to 592) | 0.48 (0.28 to 0.7) | 1.28 (1.19 to 1.37) |
| United Arab Emirates | 9 (7 to 10) | 3.17 (2.4 to 4.16) | 100 (77 to 128) | 4.14 (3.14 to 5.35) | 1.07 (0.99 to 1.14) | 1 (0 to 1) | 0.28 (0.18 to 0.42) | 9 (6 to 14) | 0.37 (0.22 to 0.56) | 1.09 (1.01 to 1.18) |
| Afghanistan | 65 (49 to 84) | 1.58 (1.19 to 2.09) | 117 (86 to 154) | 1.96 (1.43 to 2.61) | 0.89 (0.74 to 1.03) | 6 (4 to 9) | 0.14 (0.08 to 0.22) | 11 (6 to 16) | 0.17 (0.1 to 0.27) | 0.85 (0.71 to 0.99) |
| Bangladesh | 1190 (893 to 1551) | 3.76 (2.8 to 4.93) | 6939 (4951 to 9288) | 6.8 (4.77 to 9.11) | 1.95 (1.81 to 2.09) | 105 (64 to 156) | 0.33 (0.2 to 0.49) | 611 (342 to 924) | 0.6 (0.34 to 0.9) | 1.95 (1.82 to 2.09) |
| Bhutan | 4 (3 to 5) | 2.9 (2.19 to 3.86) | 28 (21 to 37) | 5.45 (4.04 to 7.23) | 2.15 (2.08 to 2.23) | 0 (0 to 1) | 0.26 (0.16 to 0.39) | 2 (2 to 4) | 0.48 (0.3 to 0.73) | 2.15 (2.08 to 2.23) |
| Yemen | 50 (38 to 64) | 1.8 (1.33 to 2.35) | 224 (169 to 289) | 2.49 (1.82 to 3.28) | 1.22 (1.13 to 1.31) | 5 (3 to 7) | 0.16 (0.1 to 0.24) | 20 (12 to 30) | 0.22 (0.13 to 0.34) | 1.23 (1.14 to 1.32) |
| Angola | 152 (98 to 232) | 8.38 (5.14 to 11.9) | 762 (502 to 1165) | 12.36 (7.98 to 18.07) | 1.47 (1.39 to 1.55) | 14 (8 to 23) | 0.74 (0.39 to 1.22) | 68 (38 to 115) | 1.09 (0.61 to 1.8) | 1.49 (1.41 to 1.57) |
| India | 7198 (5403 to 9431) | 2.92 (2.12 to 3.91) | 37731 (25831 to 52857) | 4.41 (2.95 to 6.27) | 1.29 (1.24 to 1.34) | 642 (395 to 1000) | 0.25 (0.15 to 0.39) | 3339 (1953 to 5442) | 0.39 (0.22 to 0.6) | 1.3 (1.25 to 1.35) |
| Nepal | 99 (76 to 129) | 1.95 (1.46 to 2.56) | 466 (347 to 614) | 2.95 (2.19 to 3.97) | 1.2 (1.13 to 1.27) | 9 (5 to 13) | 0.17 (0.11 to 0.27) | 42 (25 to 63) | 0.26 (0.15 to 0.39) | 1.21 (1.15 to 1.28) |
| Pakistan | 1077 (790 to 1437) | 2.85 (2.07 to 3.81) | 2563 (1843 to 3439) | 3.52 (2.44 to 4.82) | 0.66 (0.61 to 0.7) | 96 (59 to 150) | 0.25 (0.15 to 0.39) | 227 (134 to 365) | 0.31 (0.18 to 0.48) | 0.67 (0.62 to 0.71) |
| Central African Republic | 36 (22 to 55) | 7.25 (4.53 to 10.35) | 70 (45 to 108) | 7.56 (4.81 to 10.74) | 0.17 (0.15 to 0.2) | 3 (2 to 6) | 0.64 (0.36 to 1.13) | 6 (3 to 11) | 0.67 (0.37 to 1.09) | 0.18 (0.15 to 0.21) |
| Congo | 57 (35 to 86) | 10.16 (6.57 to 14.1) | 184 (122 to 274) | 12.54 (8.18 to 17.83) | 0.77 (0.71 to 0.82) | 5 (3 to 9) | 0.9 (0.49 to 1.48) | 16 (9 to 28) | 1.11 (0.59 to 1.82) | 0.77 (0.72 to 0.83) |
| Burundi | 58 (35 to 89) | 4.09 (2.51 to 5.98) | 167 (110 to 245) | 6.14 (3.83 to 8.84) | 1.43 (1.37 to 1.49) | 5 (3 to 9) | 0.36 (0.19 to 0.6) | 15 (8 to 27) | 0.54 (0.29 to 0.93) | 1.45 (1.38 to 1.51) |
| Comoros | 8 (5 to 12) | 7.05 (4.44 to 10.23) | 28 (18 to 42) | 8.28 (5.21 to 11.99) | 0.57 (0.54 to 0.59) | 1 (0 to 1) | 0.62 (0.33 to 1.08) | 3 (1 to 4) | 0.73 (0.38 to 1.17) | 0.57 (0.55 to 0.6) |
| Democratic Republic of the Congo | 649 (415 to 979) | 8.65 (5.41 to 12.56) | 1961 (1301 to 2966) | 9.74 (6.27 to 14.43) | 0.42 (0.26 to 0.59) | 59 (31 to 102) | 0.77 (0.41 to 1.28) | 176 (99 to 296) | 0.86 (0.48 to 1.44) | 0.44 (0.26 to 0.61) |
| Djibouti | 5 (3 to 7) | 8.12 (4.96 to 11.63) | 28 (18 to 41) | 8.64 (5.44 to 12.46) | 0.28 (0.18 to 0.38) | 0 (0 to 1) | 0.71 (0.4 to 1.2) | 2 (1 to 4) | 0.76 (0.41 to 1.26) | 0.3 (0.2 to 0.4) |
| Equatorial Guinea | 9 (5 to 13) | 8.42 (5.26 to 11.88) | 46 (31 to 70) | 14.94 (9.57 to 22.02) | 2.13 (1.85 to 2.41) | 1 (0 to 1) | 0.73 (0.38 to 1.23) | 4 (2 to 7) | 1.32 (0.73 to 2.23) | 2.19 (1.9 to 2.47) |
| Eritrea | 23 (15 to 35) | 5.83 (3.57 to 8.59) | 95 (61 to 143) | 7.11 (4.49 to 10.54) | 0.57 (0.51 to 0.63) | 2 (1 to 4) | 0.51 (0.27 to 0.88) | 9 (5 to 15) | 0.62 (0.32 to 1.04) | 0.6 (0.53 to 0.66) |
| Ethiopia | 350 (234 to 530) | 4.02 (2.58 to 5.75) | 2149 (1416 to 3136) | 7.24 (4.71 to 10.17) | 2.26 (2.13 to 2.39) | 32 (18 to 55) | 0.35 (0.2 to 0.57) | 190 (108 to 321) | 0.63 (0.35 to 1.05) | 2.28 (2.15 to 2.41) |
| Gabon | 50 (31 to 75) | 12.19 (7.85 to 17.06) | 91 (59 to 137) | 13.73 (8.87 to 19.9) | 0.34 (0.3 to 0.37) | 4 (3 to 8) | 1.08 (0.62 to 1.79) | 8 (5 to 14) | 1.22 (0.69 to 2) | 0.36 (0.32 to 0.4) |
| Malawi | 97 (63 to 147) | 4.76 (2.97 to 6.95) | 272 (175 to 409) | 6.21 (3.77 to 9.07) | 0.98 (0.85 to 1.11) | 9 (5 to 15) | 0.42 (0.23 to 0.7) | 24 (13 to 42) | 0.54 (0.29 to 0.91) | 0.99 (0.85 to 1.12) |
| Mauritius | 23 (16 to 30) | 4.9 (3.5 to 6.79) | 102 (74 to 135) | 6.37 (4.6 to 8.51) | 1.24 (1.01 to 1.47) | 2 (1 to 3) | 0.43 (0.25 to 0.67) | 9 (5 to 14) | 0.56 (0.34 to 0.86) | 1.24 (1.01 to 1.47) |
| Kenya | 310 (206 to 454) | 5.79 (3.72 to 8.32) | 887 (598 to 1322) | 6.55 (4.26 to 9.45) | -0.01 (-0.22 to 0.2) | 28 (16 to 47) | 0.51 (0.29 to 0.82) | 79 (46 to 132) | 0.58 (0.32 to 0.95) | 0 (-0.21 to 0.21) |
| Mozambique | 327 (215 to 475) | 9.83 (6.38 to 14.2) | 754 (496 to 1095) | 12.3 (7.96 to 16.99) | 0.7 (0.62 to 0.77) | 29 (16 to 48) | 0.85 (0.47 to 1.38) | 67 (37 to 113) | 1.07 (0.58 to 1.78) | 0.71 (0.64 to 0.79) |
| Uganda | 206 (131 to 311) | 5.16 (3.16 to 7.36) | 593 (374 to 867) | 6.36 (3.87 to 9.05) | 0.74 (0.6 to 0.88) | 18 (10 to 32) | 0.45 (0.24 to 0.78) | 53 (27 to 89) | 0.56 (0.28 to 0.95) | 0.74 (0.61 to 0.88) |
| Zambia | 103 (65 to 158) | 6.39 (3.86 to 9.21) | 373 (248 to 562) | 9.39 (5.97 to 13.72) | 1.54 (1.32 to 1.76) | 9 (5 to 16) | 0.56 (0.3 to 0.97) | 33 (18 to 57) | 0.83 (0.44 to 1.42) | 1.53 (1.31 to 1.76) |
| Madagascar | 263 (167 to 396) | 8.34 (5.08 to 11.91) | 575 (382 to 823) | 10.17 (6.33 to 14.78) | 0.76 (0.69 to 0.84) | 23 (12 to 40) | 0.73 (0.39 to 1.21) | 52 (28 to 90) | 0.9 (0.48 to 1.5) | 0.79 (0.71 to 0.87) |
| Rwanda | 74 (47 to 111) | 5.11 (3.1 to 7.33) | 232 (152 to 343) | 6.24 (3.87 to 8.85) | 0.79 (0.71 to 0.87) | 7 (4 to 12) | 0.45 (0.24 to 0.73) | 21 (11 to 35) | 0.55 (0.3 to 0.92) | 0.79 (0.71 to 0.87) |
| Seychelles | 2 (1 to 2) | 3.05 (2.23 to 4.14) | 4 (3 to 5) | 4.21 (3.02 to 5.79) | 1.32 (1.21 to 1.42) | 0 (0 to 0) | 0.27 (0.16 to 0.41) | 0 (0 to 1) | 0.37 (0.22 to 0.58) | 1.32 (1.22 to 1.42) |
| Botswana | 20 (13 to 29) | 6.73 (4.2 to 9.71) | 77 (51 to 111) | 8.58 (5.49 to 12.22) | 0.86 (0.82 to 0.9) | 2 (1 to 3) | 0.6 (0.32 to 1) | 7 (4 to 12) | 0.76 (0.41 to 1.27) | 0.88 (0.83 to 0.92) |
| Somalia | 46 (30 to 69) | 4.36 (2.57 to 6.34) | 103 (67 to 157) | 4.08 (2.55 to 5.86) | -0.2 (-0.28 to -0.13) | 4 (2 to 7) | 0.38 (0.2 to 0.64) | 9 (5 to 16) | 0.36 (0.2 to 0.61) | -0.19 (-0.27 to -0.11) |
| Eswatini | 10 (7 to 15) | 6.29 (3.83 to 8.82) | 21 (13 to 32) | 7.38 (4.65 to 10.63) | 0.49 (0.34 to 0.64) | 1 (1 to 2) | 0.56 (0.3 to 0.91) | 2 (1 to 3) | 0.66 (0.35 to 1.07) | 0.49 (0.33 to 0.66) |
| Lesotho | 33 (22 to 48) | 5.53 (3.51 to 7.92) | 36 (23 to 54) | 6.02 (3.78 to 8.69) | 0.17 (0.1 to 0.24) | 3 (2 to 5) | 0.49 (0.27 to 0.79) | 3 (2 to 6) | 0.54 (0.29 to 0.91) | 0.16 (0.08 to 0.24) |
| United Republic of Tanzania | 471 (297 to 713) | 7.3 (4.42 to 10.49) | 1492 (958 to 2189) | 8.6 (5.42 to 12.54) | 0.66 (0.57 to 0.75) | 42 (22 to 77) | 0.64 (0.34 to 1.08) | 133 (73 to 221) | 0.76 (0.41 to 1.23) | 0.68 (0.59 to 0.77) |
| Zimbabwe | 167 (109 to 243) | 7.11 (4.43 to 10.06) | 253 (167 to 379) | 7.21 (4.46 to 10.48) | -0.28 (-0.42 to -0.13) | 15 (8 to 25) | 0.63 (0.33 to 1.04) | 23 (12 to 38) | 0.64 (0.34 to 1.04) | -0.27 (-0.42 to -0.13) |
| Chad | 128 (77 to 192) | 6.6 (3.99 to 9.43) | 254 (158 to 372) | 7.94 (4.78 to 11.29) | 0.63 (0.57 to 0.7) | 11 (6 to 20) | 0.58 (0.31 to 0.98) | 23 (13 to 37) | 0.7 (0.38 to 1.16) | 0.66 (0.59 to 0.73) |
| Namibia | 22 (14 to 34) | 6.31 (4.06 to 9.29) | 74 (50 to 108) | 8.65 (5.61 to 12.12) | 1.12 (1.01 to 1.22) | 2 (1 to 3) | 0.56 (0.29 to 0.91) | 7 (4 to 11) | 0.77 (0.42 to 1.23) | 1.13 (1.02 to 1.23) |
| Coted'Ivoire | 151 (99 to 228) | 8.8 (5.47 to 12.72) | 615 (403 to 892) | 10.62 (6.67 to 14.99) | 0.55 (0.5 to 0.59) | 14 (7 to 23) | 0.78 (0.43 to 1.25) | 55 (30 to 93) | 0.94 (0.51 to 1.56) | 0.56 (0.51 to 0.6) |
| South Africa | 1474 (984 to 2064) | 9.14 (5.95 to 12.8) | 3468 (2339 to 4934) | 10.21 (6.71 to 14.2) | 0.39 (0.28 to 0.51) | 131 (76 to 210) | 0.81 (0.47 to 1.29) | 311 (180 to 506) | 0.91 (0.53 to 1.46) | 0.4 (0.28 to 0.52) |
| Benin | 83 (51 to 119) | 5.76 (3.49 to 8.3) | 249 (157 to 355) | 7.69 (4.84 to 10.89) | 0.95 (0.9 to 1) | 7 (4 to 13) | 0.5 (0.27 to 0.83) | 22 (12 to 35) | 0.67 (0.36 to 1.09) | 0.98 (0.93 to 1.02) |
| Gambia | 16 (10 to 24) | 8.35 (5.18 to 12.08) | 66 (41 to 99) | 10.33 (6.43 to 15.01) | 0.67 (0.62 to 0.73) | 1 (1 to 2) | 0.73 (0.39 to 1.19) | 6 (3 to 10) | 0.91 (0.51 to 1.51) | 0.68 (0.62 to 0.73) |
| Burkina Faso | 211 (127 to 324) | 8.63 (5.22 to 12.48) | 658 (412 to 963) | 11.22 (6.98 to 15.95) | 1.09 (0.99 to 1.18) | 19 (10 to 33) | 0.76 (0.42 to 1.31) | 58 (32 to 98) | 0.99 (0.55 to 1.61) | 1.11 (1.01 to 1.21) |
| Ghana | 175 (111 to 262) | 5.44 (3.36 to 8.03) | 486 (300 to 734) | 5.26 (3.15 to 7.74) | -0.57 (-0.77 to -0.37) | 16 (8 to 27) | 0.48 (0.25 to 0.79) | 44 (23 to 77) | 0.47 (0.23 to 0.8) | -0.55 (-0.74 to -0.35) |
| Cameroon | 228 (142 to 334) | 9.09 (5.69 to 13.11) | 825 (532 to 1175) | 11.92 (7.55 to 16.78) | 0.72 (0.64 to 0.8) | 20 (11 to 35) | 0.8 (0.43 to 1.34) | 73 (39 to 121) | 1.05 (0.57 to 1.7) | 0.74 (0.66 to 0.82) |
| Mali | 122 (76 to 181) | 6.14 (3.73 to 8.8) | 357 (226 to 551) | 7.5 (4.62 to 10.8) | 0.59 (0.54 to 0.64) | 11 (6 to 19) | 0.54 (0.29 to 0.91) | 32 (17 to 57) | 0.66 (0.35 to 1.14) | 0.6 (0.56 to 0.65) |
| Guinea | 172 (107 to 245) | 7.58 (4.68 to 10.87) | 347 (224 to 496) | 9.21 (5.82 to 13.07) | 0.58 (0.54 to 0.61) | 15 (9 to 25) | 0.67 (0.37 to 1.13) | 31 (17 to 52) | 0.81 (0.44 to 1.37) | 0.57 (0.54 to 0.6) |
| Mauritania | 60 (37 to 87) | 9.16 (5.7 to 13.16) | 185 (115 to 266) | 12.23 (7.7 to 17.59) | 0.96 (0.92 to 1.01) | 5 (3 to 9) | 0.8 (0.43 to 1.32) | 16 (9 to 28) | 1.08 (0.58 to 1.85) | 0.98 (0.94 to 1.02) |
| Cabo Verde | 34 (20 to 49) | 13.91 (8.45 to 19.82) | 64 (40 to 90) | 16.12 (10.15 to 22.56) | 0.6 (0.5 to 0.7) | 3 (2 to 5) | 1.23 (0.67 to 2.1) | 6 (3 to 9) | 1.43 (0.77 to 2.41) | 0.61 (0.51 to 0.71) |
| Guinea-Bissau | 13 (8 to 20) | 7.07 (4.4 to 10.47) | 28 (18 to 42) | 8.86 (5.56 to 12.77) | 0.67 (0.62 to 0.72) | 1 (1 to 2) | 0.62 (0.33 to 1.06) | 2 (1 to 4) | 0.78 (0.42 to 1.26) | 0.69 (0.64 to 0.74) |
| Togo | 56 (36 to 82) | 8.37 (5.24 to 12.19) | 188 (123 to 274) | 10.09 (6.43 to 14.36) | 0.53 (0.48 to 0.58) | 5 (3 to 9) | 0.74 (0.4 to 1.24) | 17 (9 to 28) | 0.89 (0.5 to 1.44) | 0.53 (0.48 to 0.59) |
| Niger | 80 (49 to 120) | 6.2 (3.77 to 8.9) | 303 (191 to 447) | 7.25 (4.55 to 10.41) | 0.43 (0.38 to 0.48) | 7 (4 to 13) | 0.55 (0.3 to 0.88) | 27 (15 to 48) | 0.64 (0.35 to 1.07) | 0.45 (0.4 to 0.49) |
| Liberia | 55 (34 to 83) | 7.68 (4.72 to 10.93) | 113 (72 to 163) | 9.41 (5.95 to 13.21) | 0.72 (0.68 to 0.77) | 5 (3 to 8) | 0.68 (0.36 to 1.12) | 10 (5 to 16) | 0.83 (0.46 to 1.33) | 0.73 (0.69 to 0.77) |
| Nigeria | 2018 (1422 to 2757) | 7.24 (5.02 to 9.66) | 4020 (2824 to 5330) | 7.32 (5.1 to 9.72) | -0.16 (-0.33 to 0.01) | 178 (107 to 289) | 0.63 (0.37 to 1) | 355 (216 to 573) | 0.64 (0.39 to 1) | -0.14 (-0.31 to 0.03) |
| Greenland | 2 (1 to 3) | 8.39 (4.74 to 12.58) | 6 (3 to 9) | 11.89 (6.48 to 18.14) | 1.74 (1.58 to 1.9) | 0 (0 to 0) | 0.75 (0.36 to 1.23) | 1 (0 to 1) | 1.08 (0.5 to 1.78) | 1.74 (1.59 to 1.9) |
| Sao Tome and Principe | 3 (2 to 4) | 5.76 (3.63 to 8.18) | 6 (4 to 8) | 8.06 (5.13 to 11.4) | 1.17 (1.13 to 1.2) | 0 (0 to 0) | 0.51 (0.27 to 0.88) | 1 (0 to 1) | 0.71 (0.39 to 1.19) | 1.16 (1.11 to 1.2) |
| Senegal | 185 (119 to 263) | 9.12 (5.81 to 12.86) | 607 (394 to 849) | 11.81 (7.61 to 16.22) | 0.74 (0.7 to 0.78) | 16 (9 to 29) | 0.8 (0.44 to 1.32) | 54 (30 to 88) | 1.04 (0.58 to 1.69) | 0.77 (0.73 to 0.8) |
| Guam | 2 (2 to 3) | 5.59 (4.05 to 7.45) | 15 (11 to 21) | 6.73 (4.75 to 9.39) | 0.71 (0.62 to 0.81) | 0 (0 to 0) | 0.5 (0.3 to 0.75) | 1 (1 to 2) | 0.6 (0.35 to 0.95) | 0.69 (0.59 to 0.79) |
| American Samoa | 1 (1 to 1) | 6.84 (5.05 to 9.32) | 3 (2 to 4) | 7.81 (5.57 to 10.75) | 0.58 (0.51 to 0.65) | 0 (0 to 0) | 0.6 (0.35 to 0.93) | 0 (0 to 0) | 0.69 (0.4 to 1.05) | 0.6 (0.53 to 0.67) |
| Sierra Leone | 105 (65 to 154) | 7.38 (4.59 to 10.76) | 229 (147 to 333) | 9.72 (6.14 to 13.83) | 0.81 (0.73 to 0.89) | 9 (5 to 16) | 0.65 (0.34 to 1.12) | 20 (12 to 36) | 0.86 (0.48 to 1.46) | 0.82 (0.74 to 0.91) |
| Bermuda | 4 (3 to 6) | 8.15 (6.03 to 10.85) | 19 (13 to 26) | 11.82 (8.55 to 16.12) | 1.17 (0.93 to 1.41) | 0 (0 to 1) | 0.73 (0.45 to 1.12) | 2 (1 to 3) | 1.06 (0.65 to 1.62) | 1.18 (0.94 to 1.42) |
| Palau | 0 (0 to 0) | 4.27 (3.13 to 5.77) | 1 (0 to 1) | 4.26 (3.03 to 5.91) | -0.12 (-0.19 to -0.05) | 0 (0 to 0) | 0.38 (0.23 to 0.59) | 0 (0 to 0) | 0.38 (0.21 to 0.6) | -0.11 (-0.18 to -0.04) |
| Cook Islands | 0 (0 to 1) | 4.24 (3.04 to 5.77) | 2 (1 to 2) | 6.57 (4.65 to 9.21) | 1.61 (1.54 to 1.68) | 0 (0 to 0) | 0.37 (0.22 to 0.6) | 0 (0 to 0) | 0.58 (0.34 to 0.9) | 1.61 (1.54 to 1.68) |
| Puerto Rico | 279 (209 to 366) | 8.45 (6.37 to 11.3) | 1217 (855 to 1647) | 13.07 (9.37 to 17.57) | 1.22 (1.01 to 1.43) | 25 (15 to 39) | 0.75 (0.47 to 1.15) | 108 (64 to 168) | 1.17 (0.68 to 1.8) | 1.24 (1.03 to 1.44) |
| Monaco | 5 (4 to 7) | 6.12 (4.41 to 8.19) | 12 (8 to 17) | 9.58 (6.71 to 13.43) | 1.69 (1.47 to 1.9) | 0 (0 to 1) | 0.55 (0.33 to 0.87) | 1 (1 to 2) | 0.86 (0.51 to 1.38) | 1.69 (1.48 to 1.91) |
| Saint Kitts and Nevis | 2 (2 to 3) | 6.78 (5.12 to 9.32) | 4 (3 to 5) | 8.27 (5.82 to 11.1) | 0.73 (0.47 to 0.99) | 0 (0 to 0) | 0.6 (0.37 to 0.91) | 0 (0 to 1) | 0.74 (0.44 to 1.08) | 0.75 (0.49 to 1) |
| Nauru | 0 (0 to 0) | 4.18 (2.99 to 5.64) | 0 (0 to 0) | 4.9 (3.46 to 6.74) | 0.67 (0.48 to 0.85) | 0 (0 to 0) | 0.37 (0.21 to 0.55) | 0 (0 to 0) | 0.43 (0.25 to 0.69) | 0.66 (0.48 to 0.84) |
| San Marino | 4 (3 to 5) | 9.67 (6.91 to 13.34) | 13 (9 to 18) | 12.91 (9.23 to 17.69) | 1.16 (1.04 to 1.29) | 0 (0 to 1) | 0.87 (0.52 to 1.37) | 1 (1 to 2) | 1.16 (0.69 to 1.86) | 1.16 (1.03 to 1.29) |
| Niue | 0 (0 to 0) | 5.97 (4.36 to 8.21) | 0 (0 to 0) | 6.98 (4.96 to 9.69) | 0.51 (0.48 to 0.54) | 0 (0 to 0) | 0.53 (0.31 to 0.81) | 0 (0 to 0) | 0.62 (0.35 to 0.98) | 0.52 (0.49 to 0.56) |
| Northern Mariana Islands | 1 (1 to 1) | 10.06 (7.36 to 13.35) | 4 (3 to 5) | 11.91 (8.64 to 16.11) | 0.45 (0.26 to 0.64) | 0 (0 to 0) | 0.9 (0.54 to 1.39) | 0 (0 to 1) | 1.05 (0.62 to 1.68) | 0.44 (0.25 to 0.63) |
| Tokelau | 0 (0 to 0) | 5.6 (4.12 to 7.46) | 0 (0 to 0) | 7 (4.92 to 9.58) | 0.74 (0.71 to 0.76) | 0 (0 to 0) | 0.49 (0.3 to 0.76) | 0 (0 to 0) | 0.62 (0.36 to 0.97) | 0.76 (0.74 to 0.79) |
| South Sudan | 105 (65 to 157) | 6.41 (3.94 to 9.1) | 135 (90 to 197) | 6.29 (3.89 to 9.06) | 0.01 (-0.04 to 0.06) | 9 (5 to 16) | 0.56 (0.29 to 0.91) | 12 (7 to 21) | 0.55 (0.3 to 0.95) | 0.03 (-0.02 to 0.08) |
| Tuvalu | 0 (0 to 0) | 4.19 (3.07 to 5.72) | 0 (0 to 1) | 5.62 (4.04 to 7.68) | 0.88 (0.84 to 0.92) | 0 (0 to 0) | 0.37 (0.22 to 0.55) | 0 (0 to 0) | 0.5 (0.29 to 0.8) | 0.86 (0.82 to 0.91) |
| United States Virgin Islands | 4 (3 to 5) | 6.8 (5.05 to 8.96) | 15 (11 to 21) | 8.22 (5.91 to 11.29) | 0.49 (0.37 to 0.6) | 0 (0 to 1) | 0.61 (0.37 to 0.9) | 1 (1 to 2) | 0.74 (0.44 to 1.15) | 0.5 (0.38 to 0.61) |
| Sudan | 118 (89 to 153) | 1.96 (1.46 to 2.6) | 358 (273 to 467) | 2.6 (1.94 to 3.48) | 1.06 (1.01 to 1.1) | 11 (6 to 16) | 0.17 (0.1 to 0.26) | 32 (20 to 48) | 0.23 (0.14 to 0.35) | 1.09 (1.04 to 1.13) |

# TableS3 Joinpoint Analysis of Burden of Heart Failure Attributable to Atrial Fibrillation and Flutter From 1990 to 2021.

| **Measure** | **Location** | **Sex** | **joinpoint** | **Start.Obs** | **End.Obs** | **Test.Statistic** | **AAPC**  **(95CI%)** | **P.Value** |
| --- | --- | --- | --- | --- | --- | --- | --- | --- |
| Prevalence | Afghanistan | Both | 5 | 1990 | 2021 | 25.123033978963 | 0.69 (0.64 to 0.75) | 0 |
| Prevalence | Afghanistan | Female | 4 | 1990 | 2021 | 20.468662035429 | 0.75 (0.68 to 0.82) | 0 |
| Prevalence | Afghanistan | Male | 5 | 1990 | 2021 | 26.734541625072 | 0.6 (0.56 to 0.65) | 0 |
| Prevalence | Albania | Both | 4 | 1990 | 2021 | 29.661240944674 | 1.17 (1.09 to 1.25) | 0 |
| Prevalence | Albania | Female | 5 | 1990 | 2021 | 39.571877313797 | 1.2 (1.14 to 1.26) | 0 |
| Prevalence | Albania | Male | 5 | 1990 | 2021 | 52.69854961 | 1.31 (1.26 to 1.36) | 0 |
| Prevalence | Algeria | Both | 5 | 1990 | 2021 | 41.889735933935 | 1.3 (1.23 to 1.36) | 0 |
| Prevalence | Algeria | Female | 5 | 1990 | 2021 | 67.11907284 | 1.2 (1.17 to 1.24) | 0 |
| Prevalence | Algeria | Male | 5 | 1990 | 2021 | 38.149024379563 | 1.38 (1.3 to 1.45) | 0 |
| Prevalence | American Samoa | Both | 5 | 1990 | 2021 | 25.198352977944 | 0.43 (0.4 to 0.46) | 0 |
| Prevalence | American Samoa | Female | 5 | 1990 | 2021 | 11.670826133893 | 0.34 (0.28 to 0.39) | 0 |
| Prevalence | American Samoa | Male | 3 | 1990 | 2021 | 7.340713956 | 0.65 (0.48 to 0.83) | 0 |
| Prevalence | Andean Latin America | Both | 4 | 1990 | 2021 | 14.886863224771 | 0.95 (0.82 to 1.07) | 0 |
| Prevalence | Andean Latin America | Female | 5 | 1990 | 2021 | 27.27208182 | 1.13 (1.05 to 1.21) | 0 |
| Prevalence | Andean Latin America | Male | 5 | 1990 | 2021 | 24.516043120894 | 0.75 (0.69 to 0.81) | 0 |
| Prevalence | Andorra | Both | 5 | 1990 | 2021 | 36.969175051683 | 1.09 (1.03 to 1.15) | 0 |
| Prevalence | Andorra | Female | 3 | 1990 | 2021 | 30.726462010459 | 0.83 (0.78 to 0.88) | 0 |
| Prevalence | Andorra | Male | 2 | 1990 | 2021 | 30.837111564649 | 1.27 (1.19 to 1.35) | 0 |
| Prevalence | Angola | Both | 4 | 1990 | 2021 | 71.229001975044 | 1.27 (1.23 to 1.3) | 0 |
| Prevalence | Angola | Female | 5 | 1990 | 2021 | 65.312503907843 | 1.25 (1.21 to 1.29) | 0 |
| Prevalence | Angola | Male | 4 | 1990 | 2021 | 91.577839084324 | 1.2 (1.17 to 1.22) | 0 |
| Prevalence | Antigua and Barbuda | Both | 5 | 1990 | 2021 | -1.575520563 | -0.34 (-0.77 to 0.08) | 0.115 |
| Prevalence | Antigua and Barbuda | Female | 3 | 1990 | 2021 | -2.460347003 | -0.29 (-0.52 to -0.06) | 0.014 |
| Prevalence | Antigua and Barbuda | Male | 3 | 1990 | 2021 | -5.374798109 | -0.6 (-0.81 to -0.38) | 0 |
| Prevalence | Argentina | Both | 5 | 1990 | 2021 | 13.412124624271 | 1.35 (1.15 to 1.55) | 0 |
| Prevalence | Argentina | Female | 5 | 1990 | 2021 | 12.583572052243 | 1.41 (1.19 to 1.63) | 0 |
| Prevalence | Argentina | Male | 5 | 1990 | 2021 | 15.23947256 | 1.26 (1.1 to 1.42) | 0 |
| Prevalence | Armenia | Both | 4 | 1990 | 2021 | 34.217385788532 | 2.76 (2.6 to 2.92) | 0 |
| Prevalence | Armenia | Female | 5 | 1990 | 2021 | 20.484069414476 | 2.51 (2.26 to 2.75) | 0 |
| Prevalence | Armenia | Male | 4 | 1990 | 2021 | 19.189981646687 | 3.21 (2.88 to 3.55) | 0 |
| Prevalence | Australasia | Both | 4 | 1990 | 2021 | 40.590762141117 | 2.53 (2.41 to 2.66) | 0 |
| Prevalence | Australasia | Female | 3 | 1990 | 2021 | 14.817338661021 | 2.57 (2.22 to 2.91) | 0 |
| Prevalence | Australasia | Male | 5 | 1990 | 2021 | 69.001563820919 | 2.5 (2.42 to 2.57) | 0 |
| Prevalence | Australia | Both | 4 | 1990 | 2021 | 44.839439418715 | 2.72 (2.6 to 2.84) | 0 |
| Prevalence | Australia | Female | 4 | 1990 | 2021 | 15.73833304 | 2.87 (2.51 to 3.24) | 0 |
| Prevalence | Australia | Male | 5 | 1990 | 2021 | 59.400419486202 | 2.53 (2.45 to 2.62) | 0 |
| Prevalence | Austria | Both | 5 | 1990 | 2021 | 22.571000278082 | 1.53 (1.39 to 1.66) | 0 |
| Prevalence | Austria | Female | 5 | 1990 | 2021 | 32.785833121359 | 1.33 (1.25 to 1.41) | 0 |
| Prevalence | Austria | Male | 5 | 1990 | 2021 | 23.824697159103 | 2.05 (1.88 to 2.22) | 0 |
| Prevalence | Azerbaijan | Both | 5 | 1990 | 2021 | 23.535979923579 | 0.84 (0.77 to 0.91) | 0 |
| Prevalence | Azerbaijan | Female | 5 | 1990 | 2021 | 16.647022728676 | 0.98 (0.86 to 1.1) | 0 |
| Prevalence | Azerbaijan | Male | 5 | 1990 | 2021 | 7.287426472 | 0.55 (0.4 to 0.7) | 0 |
| Prevalence | Bahamas | Both | 4 | 1990 | 2021 | 3.332626776 | 0.16 (0.07 to 0.26) | 0.001 |
| Prevalence | Bahamas | Female | 5 | 1990 | 2021 | -0.456951348 | -0.05 (-0.25 to 0.15) | 0.648 |
| Prevalence | Bahamas | Male | 4 | 1990 | 2021 | 7.813538868 | 0.3 (0.23 to 0.38) | 0 |
| Prevalence | Bahrain | Both | 3 | 1990 | 2021 | 71.566219206027 | 2.12 (2.06 to 2.18) | 0 |
| Prevalence | Bahrain | Female | 4 | 1990 | 2021 | 68.45970358 | 2.07 (2.01 to 2.13) | 0 |
| Prevalence | Bahrain | Male | 5 | 1990 | 2021 | 37.295953885341 | 2.17 (2.05 to 2.28) | 0 |
| Prevalence | Bangladesh | Both | 3 | 1990 | 2021 | 35.559990083434 | 1.87 (1.76 to 1.97) | 0 |
| Prevalence | Bangladesh | Female | 3 | 1990 | 2021 | 118.90271889415 | 1.6 (1.58 to 1.63) | 0 |
| Prevalence | Bangladesh | Male | 4 | 1990 | 2021 | 51.932177323455 | 2.18 (2.09 to 2.26) | 0 |
| Prevalence | Barbados | Both | 5 | 1990 | 2021 | 26.040323606422 | 0.95 (0.88 to 1.02) | 0 |
| Prevalence | Barbados | Female | 5 | 1990 | 2021 | 19.181883620328 | 0.72 (0.65 to 0.79) | 0 |
| Prevalence | Barbados | Male | 4 | 1990 | 2021 | 30.571960402655 | 1.01 (0.95 to 1.08) | 0 |
| Prevalence | Belarus | Both | 5 | 1990 | 2021 | 6.312628591 | 1.07 (0.74 to 1.41) | 0 |
| Prevalence | Belarus | Female | 5 | 1990 | 2021 | 8.276858459 | 1.01 (0.77 to 1.25) | 0 |
| Prevalence | Belarus | Male | 5 | 1990 | 2021 | 15.177198111578 | 1.41 (1.23 to 1.6) | 0 |
| Prevalence | Belgium | Both | 3 | 1990 | 2021 | 9.956642886 | 1.63 (1.3 to 1.95) | 0 |
| Prevalence | Belgium | Female | 3 | 1990 | 2021 | 21.143542470153 | 1.31 (1.19 to 1.43) | 0 |
| Prevalence | Belgium | Male | 4 | 1990 | 2021 | 20.274427547319 | 2.44 (2.2 to 2.68) | 0 |
| Prevalence | Belize | Both | 5 | 1990 | 2021 | 0.415662592 | 0.06 (-0.23 to 0.36) | 0.678 |
| Prevalence | Belize | Female | 5 | 1990 | 2021 | 4.497493285 | 0.29 (0.16 to 0.41) | 0 |
| Prevalence | Belize | Male | 5 | 1990 | 2021 | -1.994679374 | -0.28 (-0.55 to 0) | 0.046 |
| Prevalence | Benin | Both | 5 | 1990 | 2021 | 36.8827852 | 0.93 (0.88 to 0.98) | 0 |
| Prevalence | Benin | Female | 3 | 1990 | 2021 | 47.433783069283 | 1.08 (1.03 to 1.12) | 0 |
| Prevalence | Benin | Male | 5 | 1990 | 2021 | 19.619564792055 | 0.84 (0.76 to 0.92) | 0 |
| Prevalence | Bermuda | Both | 5 | 1990 | 2021 | 84.257396912033 | 1.2 (1.17 to 1.22) | 0 |
| Prevalence | Bermuda | Female | 5 | 1990 | 2021 | 41.516932518703 | 1.16 (1.1 to 1.21) | 0 |
| Prevalence | Bermuda | Male | 5 | 1990 | 2021 | 42.714612937036 | 1.3 (1.24 to 1.36) | 0 |
| Prevalence | Bhutan | Both | 3 | 1990 | 2021 | 80.400856785631 | 2.05 (2 to 2.1) | 0 |
| Prevalence | Bhutan | Female | 5 | 1990 | 2021 | 46.292081768122 | 2.19 (2.1 to 2.29) | 0 |
| Prevalence | Bhutan | Male | 3 | 1990 | 2021 | 39.574372964584 | 1.75 (1.66 to 1.83) | 0 |
| Prevalence | Bolivia (Plurinational State of) | Both | 5 | 1990 | 2021 | 71.324995445282 | 1.21 (1.17 to 1.24) | 0 |
| Prevalence | Bolivia (Plurinational State of) | Female | 5 | 1990 | 2021 | 88.750773160058 | 1.32 (1.29 to 1.35) | 0 |
| Prevalence | Bolivia (Plurinational State of) | Male | 2 | 1990 | 2021 | 62.454388822195 | 1.03 (1 to 1.06) | 0 |
| Prevalence | Bosnia and Herzegovina | Both | 5 | 1990 | 2021 | 19.23877311 | 1.12 (1.01 to 1.24) | 0 |
| Prevalence | Bosnia and Herzegovina | Female | 4 | 1990 | 2021 | 25.148429025106 | 1.35 (1.25 to 1.46) | 0 |
| Prevalence | Bosnia and Herzegovina | Male | 5 | 1990 | 2021 | 6.896090635 | 0.79 (0.57 to 1.02) | 0 |
| Prevalence | Botswana | Both | 5 | 1990 | 2021 | 44.940525558359 | 0.78 (0.75 to 0.82) | 0 |
| Prevalence | Botswana | Female | 5 | 1990 | 2021 | 91.584837982339 | 1.02 (0.99 to 1.04) | 0 |
| Prevalence | Botswana | Male | 5 | 1990 | 2021 | 6.203431481 | 0.34 (0.24 to 0.45) | 0 |
| Prevalence | Brazil | Both | 5 | 1990 | 2021 | 61.176607768034 | 1.99 (1.92 to 2.05) | 0 |
| Prevalence | Brazil | Female | 5 | 1990 | 2021 | 89.14358535 | 2.14 (2.09 to 2.19) | 0 |
| Prevalence | Brazil | Male | 4 | 1990 | 2021 | 57.811380582328 | 1.78 (1.72 to 1.84) | 0 |
| Prevalence | Brunei Darussalam | Both | 5 | 1990 | 2021 | 35.544010375409 | 1.24 (1.17 to 1.31) | 0 |
| Prevalence | Brunei Darussalam | Female | 4 | 1990 | 2021 | 57.452100893402 | 1.06 (1.03 to 1.1) | 0 |
| Prevalence | Brunei Darussalam | Male | 5 | 1990 | 2021 | 13.81171722 | 1.39 (1.19 to 1.59) | 0 |
| Prevalence | Bulgaria | Both | 5 | 1990 | 2021 | 0.805028082 | 0.24 (-0.34 to 0.82) | 0.421 |
| Prevalence | Bulgaria | Female | 5 | 1990 | 2021 | -0.488685758 | -0.08 (-0.4 to 0.24) | 0.625 |
| Prevalence | Bulgaria | Male | 5 | 1990 | 2021 | 3.361007214 | 0.7 (0.29 to 1.11) | 0.001 |
| Prevalence | Burkina Faso | Both | 5 | 1990 | 2021 | 16.046296031397 | 0.86 (0.75 to 0.96) | 0 |
| Prevalence | Burkina Faso | Female | 5 | 1990 | 2021 | 27.491120727834 | 1.12 (1.04 to 1.21) | 0 |
| Prevalence | Burkina Faso | Male | 5 | 1990 | 2021 | 42.51381359 | 0.59 (0.56 to 0.62) | 0 |
| Prevalence | Burundi | Both | 5 | 1990 | 2021 | 92.281055450937 | 1.32 (1.29 to 1.34) | 0 |
| Prevalence | Burundi | Female | 5 | 1990 | 2021 | 97.756803064677 | 1.71 (1.68 to 1.75) | 0 |
| Prevalence | Burundi | Male | 5 | 1990 | 2021 | 83.633285055105 | 0.87 (0.85 to 0.89) | 0 |
| Prevalence | Cabo Verde | Both | 5 | 1990 | 2021 | 8.666808187 | 0.46 (0.35 to 0.56) | 0 |
| Prevalence | Cabo Verde | Female | 5 | 1990 | 2021 | 40.122340374337 | 0.68 (0.65 to 0.71) | 0 |
| Prevalence | Cabo Verde | Male | 5 | 1990 | 2021 | -0.530873631 | -0.04 (-0.18 to 0.11) | 0.596 |
| Prevalence | Cambodia | Both | 4 | 1990 | 2021 | 133.887775529288 | 1.03 (1.02 to 1.05) | 0 |
| Prevalence | Cambodia | Female | 5 | 1990 | 2021 | 127.060525792797 | 0.98 (0.96 to 0.99) | 0 |
| Prevalence | Cambodia | Male | 5 | 1990 | 2021 | 73.905142029318 | 1.12 (1.09 to 1.15) | 0 |
| Prevalence | Cameroon | Both | 5 | 1990 | 2021 | 29.512156728972 | 0.88 (0.82 to 0.94) | 0 |
| Prevalence | Cameroon | Female | 4 | 1990 | 2021 | 28.229623239949 | 0.97 (0.9 to 1.04) | 0 |
| Prevalence | Cameroon | Male | 5 | 1990 | 2021 | 33.469687625376 | 0.69 (0.65 to 0.73) | 0 |
| Prevalence | Canada | Both | 4 | 1990 | 2021 | 13.049112844778 | 2.25 (1.91 to 2.6) | 0 |
| Prevalence | Canada | Female | 5 | 1990 | 2021 | 13.986567131199 | 2.21 (1.89 to 2.52) | 0 |
| Prevalence | Canada | Male | 4 | 1990 | 2021 | 28.135384028066 | 2.34 (2.18 to 2.51) | 0 |
| Prevalence | Caribbean | Both | 5 | 1990 | 2021 | 23.10356762 | 0.81 (0.74 to 0.88) | 0 |
| Prevalence | Caribbean | Female | 5 | 1990 | 2021 | 27.452130835315 | 0.96 (0.89 to 1.03) | 0 |
| Prevalence | Caribbean | Male | 5 | 1990 | 2021 | 11.829991251005 | 0.71 (0.6 to 0.83) | 0 |
| Prevalence | Central African Republic | Both | 5 | 1990 | 2021 | 9.740394316 | 0.14 (0.11 to 0.17) | 0 |
| Prevalence | Central African Republic | Female | 5 | 1990 | 2021 | 7.663231424 | 0.04 (0.03 to 0.05) | 0 |
| Prevalence | Central African Republic | Male | 4 | 1990 | 2021 | 11.691253820386 | 0.22 (0.18 to 0.25) | 0 |
| Prevalence | Central Asia | Both | 5 | 1990 | 2021 | 7.753151596 | 0.61 (0.46 to 0.77) | 0 |
| Prevalence | Central Asia | Female | 5 | 1990 | 2021 | 3.841881463 | 0.51 (0.25 to 0.77) | 0 |
| Prevalence | Central Asia | Male | 5 | 1990 | 2021 | 10.268606049059 | 0.84 (0.68 to 1.01) | 0 |
| Prevalence | Central Europe | Both | 4 | 1990 | 2021 | 19.021250632258 | 1.4 (1.26 to 1.55) | 0 |
| Prevalence | Central Europe | Female | 5 | 1990 | 2021 | 20.318454542355 | 1.49 (1.34 to 1.63) | 0 |
| Prevalence | Central Europe | Male | 4 | 1990 | 2021 | 18.856942319702 | 1.38 (1.24 to 1.53) | 0 |
| Prevalence | Central Latin America | Both | 5 | 1990 | 2021 | 34.224434602321 | 0.9 (0.85 to 0.95) | 0 |
| Prevalence | Central Latin America | Female | 5 | 1990 | 2021 | 51.360033463263 | 1.14 (1.1 to 1.18) | 0 |
| Prevalence | Central Latin America | Male | 5 | 1990 | 2021 | 11.0875915 | 0.72 (0.59 to 0.85) | 0 |
| Prevalence | Central Sub-Saharan Africa | Both | 5 | 1990 | 2021 | 43.956390115948 | 0.58 (0.55 to 0.6) | 0 |
| Prevalence | Central Sub-Saharan Africa | Female | 5 | 1990 | 2021 | 38.042356958591 | 0.44 (0.41 to 0.46) | 0 |
| Prevalence | Central Sub-Saharan Africa | Male | 3 | 1990 | 2021 | 50.202693731413 | 0.76 (0.73 to 0.79) | 0 |
| Prevalence | Chad | Both | 4 | 1990 | 2021 | 36.788803986952 | 0.6 (0.56 to 0.63) | 0 |
| Prevalence | Chad | Female | 4 | 1990 | 2021 | 67.150378355736 | 0.56 (0.54 to 0.58) | 0 |
| Prevalence | Chad | Male | 3 | 1990 | 2021 | 23.691676099034 | 0.6 (0.55 to 0.65) | 0 |
| Prevalence | Chile | Both | 5 | 1990 | 2021 | 18.156026512239 | 2.2 (1.96 to 2.44) | 0 |
| Prevalence | Chile | Female | 4 | 1990 | 2021 | 9.803201851 | 2.1 (1.67 to 2.52) | 0 |
| Prevalence | Chile | Male | 3 | 1990 | 2021 | 37.105570590798 | 2.37 (2.24 to 2.49) | 0 |
| Prevalence | China | Both | 4 | 1990 | 2021 | 22.893001120878 | 1.39 (1.27 to 1.51) | 0 |
| Prevalence | China | Female | 4 | 1990 | 2021 | 48.071835982913 | 1.41 (1.35 to 1.46) | 0 |
| Prevalence | China | Male | 5 | 1990 | 2021 | 48.119499115744 | 1.53 (1.47 to 1.6) | 0 |
| Prevalence | Colombia | Both | 5 | 1990 | 2021 | 50.778327536967 | 2.1 (2.02 to 2.18) | 0 |
| Prevalence | Colombia | Female | 5 | 1990 | 2021 | 36.013604101507 | 2.39 (2.26 to 2.52) | 0 |
| Prevalence | Colombia | Male | 4 | 1990 | 2021 | 102.369770134699 | 1.78 (1.74 to 1.81) | 0 |
| Prevalence | Comoros | Both | 5 | 1990 | 2021 | 71.128713592002 | 0.52 (0.51 to 0.54) | 0 |
| Prevalence | Comoros | Female | 5 | 1990 | 2021 | 46.590670015314 | 0.46 (0.44 to 0.48) | 0 |
| Prevalence | Comoros | Male | 5 | 1990 | 2021 | 24.647112631459 | 0.52 (0.48 to 0.57) | 0 |
| Prevalence | Congo | Both | 5 | 1990 | 2021 | 65.258163481419 | 0.68 (0.66 to 0.7) | 0 |
| Prevalence | Congo | Female | 4 | 1990 | 2021 | 22.514956583053 | 0.59 (0.54 to 0.64) | 0 |
| Prevalence | Congo | Male | 5 | 1990 | 2021 | 89.850891228885 | 0.87 (0.85 to 0.89) | 0 |
| Prevalence | Cook Islands | Both | 4 | 1990 | 2021 | 81.133165087243 | 1.43 (1.39 to 1.46) | 0 |
| Prevalence | Cook Islands | Female | 5 | 1990 | 2021 | 79.862639579527 | 1.74 (1.69 to 1.78) | 0 |
| Prevalence | Cook Islands | Male | 5 | 1990 | 2021 | 53.594111214383 | 0.97 (0.93 to 1.01) | 0 |
| Prevalence | Costa Rica | Both | 5 | 1990 | 2021 | 45.537885905117 | 1.5 (1.44 to 1.57) | 0 |
| Prevalence | Costa Rica | Female | 5 | 1990 | 2021 | 54.493053652184 | 1.85 (1.78 to 1.92) | 0 |
| Prevalence | Costa Rica | Male | 4 | 1990 | 2021 | 19.719523607349 | 1.17 (1.05 to 1.29) | 0 |
| Prevalence | Coted'Ivoire | Both | 3 | 1990 | 2021 | 31.199639966882 | 0.6 (0.56 to 0.64) | 0 |
| Prevalence | Coted'Ivoire | Female | 5 | 1990 | 2021 | 75.276181866673 | 0.55 (0.54 to 0.57) | 0 |
| Prevalence | Coted'Ivoire | Male | 3 | 1990 | 2021 | 26.4514634 | 0.62 (0.57 to 0.66) | 0 |
| Prevalence | Croatia | Both | 3 | 1990 | 2021 | 13.97324592 | 2 (1.72 to 2.28) | 0 |
| Prevalence | Croatia | Female | 5 | 1990 | 2021 | 23.399334334425 | 2.21 (2.03 to 2.4) | 0 |
| Prevalence | Croatia | Male | 3 | 1990 | 2021 | 4.4523014 | 1.68 (0.94 to 2.42) | 0 |
| Prevalence | Cuba | Both | 5 | 1990 | 2021 | 21.287613807869 | 0.77 (0.7 to 0.84) | 0 |
| Prevalence | Cuba | Female | 5 | 1990 | 2021 | 38.178954392666 | 0.86 (0.81 to 0.9) | 0 |
| Prevalence | Cuba | Male | 4 | 1990 | 2021 | 36.417994910335 | 0.75 (0.71 to 0.79) | 0 |
| Prevalence | Cyprus | Both | 5 | 1990 | 2021 | 4.160574712 | 1.49 (0.79 to 2.2) | 0 |
| Prevalence | Cyprus | Female | 4 | 1990 | 2021 | 3.58034126 | 1.42 (0.64 to 2.21) | 0 |
| Prevalence | Cyprus | Male | 5 | 1990 | 2021 | 4.344558041 | 1.56 (0.85 to 2.27) | 0 |
| Prevalence | Czechia | Both | 3 | 1990 | 2021 | 9.383979719 | 2.65 (2.09 to 3.21) | 0 |
| Prevalence | Czechia | Female | 3 | 1990 | 2021 | 9.084253255 | 2.68 (2.1 to 3.27) | 0 |
| Prevalence | Czechia | Male | 2 | 1990 | 2021 | 12.933773273802 | 2.74 (2.32 to 3.17) | 0 |
| Prevalence | Democratic People's Republic of Korea | Both | 5 | 1990 | 2021 | 17.189528140914 | 0.58 (0.52 to 0.65) | 0 |
| Prevalence | Democratic People's Republic of Korea | Female | 5 | 1990 | 2021 | 25.944577231457 | 0.55 (0.51 to 0.6) | 0 |
| Prevalence | Democratic People's Republic of Korea | Male | 4 | 1990 | 2021 | 13.368973085866 | 0.55 (0.47 to 0.63) | 0 |
| Prevalence | Democratic Republic of the Congo | Both | 5 | 1990 | 2021 | 23.147659241764 | 0.38 (0.35 to 0.41) | 0 |
| Prevalence | Democratic Republic of the Congo | Female | 4 | 1990 | 2021 | 17.469145511073 | 0.22 (0.2 to 0.25) | 0 |
| Prevalence | Democratic Republic of the Congo | Male | 5 | 1990 | 2021 | 41.12862801 | 0.65 (0.62 to 0.68) | 0 |
| Prevalence | Denmark | Both | 2 | 1990 | 2021 | 30.441458437308 | 3.84 (3.59 to 4.1) | 0 |
| Prevalence | Denmark | Female | 2 | 1990 | 2021 | 25.435441622076 | 3.45 (3.18 to 3.73) | 0 |
| Prevalence | Denmark | Male | 3 | 1990 | 2021 | 19.891692222757 | 4.32 (3.89 to 4.76) | 0 |
| Prevalence | Djibouti | Both | 5 | 1990 | 2021 | 23.336127070736 | 0.21 (0.19 to 0.23) | 0 |
| Prevalence | Djibouti | Female | 5 | 1990 | 2021 | 20.556173964257 | 0.3 (0.28 to 0.33) | 0 |
| Prevalence | Djibouti | Male | 3 | 1990 | 2021 | 5.280661411 | 0.08 (0.05 to 0.11) | 0 |
| Prevalence | Dominica | Both | 5 | 1990 | 2021 | 28.060999466111 | 0.64 (0.6 to 0.69) | 0 |
| Prevalence | Dominica | Female | 5 | 1990 | 2021 | 71.145575769561 | 0.92 (0.89 to 0.94) | 0 |
| Prevalence | Dominica | Male | 5 | 1990 | 2021 | 18.445097006439 | 0.36 (0.32 to 0.39) | 0 |
| Prevalence | Dominican Republic | Both | 3 | 1990 | 2021 | 1.878798679 | 0.25 (-0.01 to 0.51) | 0.06 |
| Prevalence | Dominican Republic | Female | 2 | 1990 | 2021 | 15.785240732814 | 0.68 (0.6 to 0.77) | 0 |
| Prevalence | Dominican Republic | Male | 5 | 1990 | 2021 | -1.701354289 | -0.1 (-0.22 to 0.02) | 0.089 |
| Prevalence | East Asia | Both | 4 | 1990 | 2021 | 25.145791673112 | 1.41 (1.3 to 1.52) | 0 |
| Prevalence | East Asia | Female | 5 | 1990 | 2021 | 35.979069673009 | 1.42 (1.34 to 1.5) | 0 |
| Prevalence | East Asia | Male | 5 | 1990 | 2021 | 62.879314272526 | 1.54 (1.49 to 1.59) | 0 |
| Prevalence | Eastern Europe | Both | 5 | 1990 | 2021 | 10.42658035 | 1 (0.81 to 1.19) | 0 |
| Prevalence | Eastern Europe | Female | 4 | 1990 | 2021 | 8.224635806 | 0.93 (0.71 to 1.15) | 0 |
| Prevalence | Eastern Europe | Male | 4 | 1990 | 2021 | 18.445124894874 | 1.53 (1.37 to 1.7) | 0 |
| Prevalence | Eastern Sub-Saharan Africa | Both | 3 | 1990 | 2021 | 46.385575201096 | 0.75 (0.71 to 0.78) | 0 |
| Prevalence | Eastern Sub-Saharan Africa | Female | 5 | 1990 | 2021 | 40.25907346 | 0.71 (0.68 to 0.75) | 0 |
| Prevalence | Eastern Sub-Saharan Africa | Male | 2 | 1990 | 2021 | 63.871195672002 | 0.8 (0.78 to 0.83) | 0 |
| Prevalence | Ecuador | Both | 5 | 1990 | 2021 | 3.260461986 | 0.12 (0.05 to 0.19) | 0.001 |
| Prevalence | Ecuador | Female | 5 | 1990 | 2021 | 17.301137950057 | 0.86 (0.76 to 0.96) | 0 |
| Prevalence | Ecuador | Male | 5 | 1990 | 2021 | -9.290454422 | -0.5 (-0.6 to -0.39) | 0 |
| Prevalence | Egypt | Both | 5 | 1990 | 2021 | 34.027093478218 | 0.95 (0.89 to 1) | 0 |
| Prevalence | Egypt | Female | 4 | 1990 | 2021 | 47.392637878332 | 1.05 (1.01 to 1.09) | 0 |
| Prevalence | Egypt | Male | 5 | 1990 | 2021 | 7.983507004 | 0.57 (0.43 to 0.71) | 0 |
| Prevalence | El Salvador | Both | 4 | 1990 | 2021 | 31.056309112269 | 0.55 (0.51 to 0.58) | 0 |
| Prevalence | El Salvador | Female | 5 | 1990 | 2021 | 57.910299058583 | 0.82 (0.79 to 0.85) | 0 |
| Prevalence | El Salvador | Male | 5 | 1990 | 2021 | 7.639989302 | 0.18 (0.13 to 0.23) | 0 |
| Prevalence | Equatorial Guinea | Both | 5 | 1990 | 2021 | 28.28215241 | 1.86 (1.73 to 1.99) | 0 |
| Prevalence | Equatorial Guinea | Female | 5 | 1990 | 2021 | 50.9857623 | 1.97 (1.89 to 2.05) | 0 |
| Prevalence | Equatorial Guinea | Male | 5 | 1990 | 2021 | 29.683040085603 | 1.64 (1.53 to 1.75) | 0 |
| Prevalence | Eritrea | Both | 3 | 1990 | 2021 | 32.069340379296 | 0.65 (0.61 to 0.69) | 0 |
| Prevalence | Eritrea | Female | 4 | 1990 | 2021 | 22.564248820472 | 0.67 (0.61 to 0.73) | 0 |
| Prevalence | Eritrea | Male | 3 | 1990 | 2021 | 24.69686604 | 0.57 (0.53 to 0.62) | 0 |
| Prevalence | Estonia | Both | 3 | 1990 | 2021 | 7.07506158 | 1.79 (1.29 to 2.29) | 0 |
| Prevalence | Estonia | Female | 3 | 1990 | 2021 | 7.335475992 | 1.61 (1.18 to 2.05) | 0 |
| Prevalence | Estonia | Male | 4 | 1990 | 2021 | 5.199180405 | 2.29 (1.42 to 3.17) | 0 |
| Prevalence | Eswatini | Both | 5 | 1990 | 2021 | 17.440517488494 | 0.51 (0.45 to 0.56) | 0 |
| Prevalence | Eswatini | Female | 5 | 1990 | 2021 | 25.646387581359 | 0.7 (0.65 to 0.75) | 0 |
| Prevalence | Eswatini | Male | 5 | 1990 | 2021 | -3.244033796 | -0.16 (-0.25 to -0.06) | 0.001 |
| Prevalence | Ethiopia | Both | 5 | 1990 | 2021 | 70.68774331 | 1.93 (1.87 to 1.98) | 0 |
| Prevalence | Ethiopia | Female | 5 | 1990 | 2021 | 92.980520181623 | 1.36 (1.33 to 1.39) | 0 |
| Prevalence | Ethiopia | Male | 5 | 1990 | 2021 | 76.213411403965 | 2.24 (2.18 to 2.3) | 0 |
| Prevalence | Fiji | Both | 4 | 1990 | 2021 | 23.30066495 | 0.95 (0.87 to 1.03) | 0 |
| Prevalence | Fiji | Female | 4 | 1990 | 2021 | 37.632963871037 | 1.13 (1.07 to 1.19) | 0 |
| Prevalence | Fiji | Male | 4 | 1990 | 2021 | 36.060771602822 | 0.62 (0.58 to 0.65) | 0 |
| Prevalence | Finland | Both | 5 | 1990 | 2021 | 5.905094409 | 0.63 (0.42 to 0.83) | 0 |
| Prevalence | Finland | Female | 5 | 1990 | 2021 | 3.099566433 | 0.32 (0.12 to 0.53) | 0.002 |
| Prevalence | Finland | Male | 3 | 1990 | 2021 | 6.43111604 | 1.77 (1.23 to 2.31) | 0 |
| Prevalence | France | Both | 5 | 1990 | 2021 | 16.369119168037 | 2.29 (2.01 to 2.57) | 0 |
| Prevalence | France | Female | 5 | 1990 | 2021 | 26.593153851263 | 2.28 (2.11 to 2.45) | 0 |
| Prevalence | France | Male | 5 | 1990 | 2021 | 18.586307923527 | 2.5 (2.23 to 2.76) | 0 |
| Prevalence | Gabon | Both | 5 | 1990 | 2021 | 17.67296993 | 0.39 (0.35 to 0.44) | 0 |
| Prevalence | Gabon | Female | 5 | 1990 | 2021 | 23.199843069409 | 0.39 (0.36 to 0.43) | 0 |
| Prevalence | Gabon | Male | 5 | 1990 | 2021 | 55.916142403156 | 0.32 (0.3 to 0.33) | 0 |
| Prevalence | Gambia | Both | 5 | 1990 | 2021 | 53.157225876604 | 0.69 (0.67 to 0.72) | 0 |
| Prevalence | Gambia | Female | 5 | 1990 | 2021 | 55.612022054669 | 0.76 (0.74 to 0.79) | 0 |
| Prevalence | Gambia | Male | 3 | 1990 | 2021 | 12.340452593556 | 0.53 (0.44 to 0.61) | 0 |
| Prevalence | Georgia | Both | 4 | 1990 | 2021 | 7.940063821 | 0.93 (0.7 to 1.17) | 0 |
| Prevalence | Georgia | Female | 4 | 1990 | 2021 | 3.648675616 | 0.71 (0.33 to 1.09) | 0 |
| Prevalence | Georgia | Male | 4 | 1990 | 2021 | 13.495380720567 | 1.49 (1.27 to 1.71) | 0 |
| Prevalence | Germany | Both | 5 | 1990 | 2021 | 18.665318757251 | 2.78 (2.48 to 3.07) | 0 |
| Prevalence | Germany | Female | 5 | 1990 | 2021 | 17.093797439322 | 2.69 (2.38 to 3.01) | 0 |
| Prevalence | Germany | Male | 4 | 1990 | 2021 | 18.217419 | 3.1 (2.76 to 3.44) | 0 |
| Prevalence | Ghana | Both | 5 | 1990 | 2021 | -1.735284571 | -0.12 (-0.25 to 0.02) | 0.083 |
| Prevalence | Ghana | Female | 5 | 1990 | 2021 | -1.798291857 | -0.13 (-0.28 to 0.01) | 0.072 |
| Prevalence | Ghana | Male | 5 | 1990 | 2021 | -5.955270455 | -0.3 (-0.39 to -0.2) | 0 |
| Prevalence | Global | Both | 2 | 1990 | 2021 | 64.944440911085 | 1.63 (1.58 to 1.67) | 0 |
| Prevalence | Global | Female | 4 | 1990 | 2021 | 51.487794642772 | 1.59 (1.53 to 1.65) | 0 |
| Prevalence | Global | Male | 3 | 1990 | 2021 | 43.41001376 | 1.67 (1.59 to 1.75) | 0 |
| Prevalence | Greece | Both | 4 | 1990 | 2021 | -0.421871986 | -0.16 (-0.89 to 0.58) | 0.673 |
| Prevalence | Greece | Female | 4 | 1990 | 2021 | -0.41284749 | -0.2 (-1.13 to 0.74) | 0.68 |
| Prevalence | Greece | Male | 4 | 1990 | 2021 | -0.600399361 | -0.17 (-0.71 to 0.38) | 0.548 |
| Prevalence | Greenland | Both | 3 | 1990 | 2021 | 14.431608524786 | 1.19 (1.03 to 1.36) | 0 |
| Prevalence | Greenland | Female | 3 | 1990 | 2021 | 16.342996921218 | 1.43 (1.26 to 1.6) | 0 |
| Prevalence | Greenland | Male | 4 | 1990 | 2021 | 18.104454221113 | 0.82 (0.73 to 0.91) | 0 |
| Prevalence | Grenada | Both | 5 | 1990 | 2021 | 5.590634778 | 0.57 (0.37 to 0.77) | 0 |
| Prevalence | Grenada | Female | 5 | 1990 | 2021 | 12.055469105343 | 0.71 (0.6 to 0.83) | 0 |
| Prevalence | Grenada | Male | 5 | 1990 | 2021 | 9.04199361 | 0.57 (0.45 to 0.7) | 0 |
| Prevalence | Guam | Both | 4 | 1990 | 2021 | 12.162000448085 | 0.59 (0.49 to 0.68) | 0 |
| Prevalence | Guam | Female | 5 | 1990 | 2021 | 7.156775234 | 0.61 (0.44 to 0.78) | 0 |
| Prevalence | Guam | Male | 4 | 1990 | 2021 | 7.622891658 | 0.3 (0.22 to 0.38) | 0 |
| Prevalence | Guatemala | Both | 4 | 1990 | 2021 | 10.8847364 | 0.79 (0.64 to 0.93) | 0 |
| Prevalence | Guatemala | Female | 5 | 1990 | 2021 | 9.644197168 | 0.85 (0.68 to 1.02) | 0 |
| Prevalence | Guatemala | Male | 3 | 1990 | 2021 | 7.527300776 | 0.67 (0.5 to 0.85) | 0 |
| Prevalence | Guinea | Both | 5 | 1990 | 2021 | 18.683771879654 | 0.63 (0.56 to 0.69) | 0 |
| Prevalence | Guinea | Female | 5 | 1990 | 2021 | 24.230098382261 | 0.72 (0.66 to 0.78) | 0 |
| Prevalence | Guinea | Male | 5 | 1990 | 2021 | 19.959093558537 | 0.57 (0.51 to 0.62) | 0 |
| Prevalence | Guinea-Bissau | Both | 4 | 1990 | 2021 | 41.225392759838 | 0.73 (0.69 to 0.76) | 0 |
| Prevalence | Guinea-Bissau | Female | 5 | 1990 | 2021 | 47.370168989272 | 0.65 (0.62 to 0.68) | 0 |
| Prevalence | Guinea-Bissau | Male | 5 | 1990 | 2021 | 28.798174456075 | 0.72 (0.67 to 0.76) | 0 |
| Prevalence | Guyana | Both | 4 | 1990 | 2021 | 16.397697502776 | 1.43 (1.26 to 1.6) | 0 |
| Prevalence | Guyana | Female | 5 | 1990 | 2021 | 8.547678932 | 1.16 (0.89 to 1.42) | 0 |
| Prevalence | Guyana | Male | 5 | 1990 | 2021 | 22.845475903182 | 1.72 (1.57 to 1.87) | 0 |
| Prevalence | Haiti | Both | 5 | 1990 | 2021 | 33.785921882616 | 0.8 (0.76 to 0.85) | 0 |
| Prevalence | Haiti | Female | 3 | 1990 | 2021 | 28.288565826363 | 0.49 (0.45 to 0.52) | 0 |
| Prevalence | Haiti | Male | 5 | 1990 | 2021 | 23.936146748523 | 0.68 (0.63 to 0.74) | 0 |
| Prevalence | High SDI | Both | 5 | 1990 | 2021 | 69.751175293559 | 2.11 (2.05 to 2.17) | 0 |
| Prevalence | High SDI | Female | 5 | 1990 | 2021 | 68.188298697278 | 2.06 (2 to 2.12) | 0 |
| Prevalence | High SDI | Male | 4 | 1990 | 2021 | 31.345243057454 | 2.25 (2.1 to 2.39) | 0 |
| Prevalence | High-income Asia Pacific | Both | 4 | 1990 | 2021 | 22.95149852 | 2.3 (2.1 to 2.5) | 0 |
| Prevalence | High-income Asia Pacific | Female | 4 | 1990 | 2021 | 24.249434291954 | 2.52 (2.31 to 2.73) | 0 |
| Prevalence | High-income Asia Pacific | Male | 5 | 1990 | 2021 | 26.269126727752 | 1.96 (1.81 to 2.1) | 0 |
| Prevalence | High-income North America | Both | 5 | 1990 | 2021 | 28.291057856159 | 2.12 (1.98 to 2.27) | 0 |
| Prevalence | High-income North America | Female | 5 | 1990 | 2021 | 26.581306221698 | 2.12 (1.97 to 2.28) | 0 |
| Prevalence | High-income North America | Male | 5 | 1990 | 2021 | 32.937102355462 | 2.06 (1.94 to 2.19) | 0 |
| Prevalence | High-middle SDI | Both | 5 | 1990 | 2021 | 35.883071617238 | 1.82 (1.72 to 1.92) | 0 |
| Prevalence | High-middle SDI | Female | 5 | 1990 | 2021 | 21.508110213156 | 1.83 (1.66 to 2) | 0 |
| Prevalence | High-middle SDI | Male | 5 | 1990 | 2021 | 50.35279586 | 1.88 (1.8 to 1.95) | 0 |
| Prevalence | Honduras | Both | 5 | 1990 | 2021 | 23.569910765817 | 0.61 (0.56 to 0.66) | 0 |
| Prevalence | Honduras | Female | 5 | 1990 | 2021 | 73.982700634057 | 0.98 (0.96 to 1.01) | 0 |
| Prevalence | Honduras | Male | 5 | 1990 | 2021 | 8.113908138 | 0.13 (0.1 to 0.16) | 0 |
| Prevalence | Hungary | Both | 5 | 1990 | 2021 | 26.597920036449 | 1.05 (0.97 to 1.13) | 0 |
| Prevalence | Hungary | Female | 3 | 1990 | 2021 | 17.897606865597 | 1.12 (0.99 to 1.24) | 0 |
| Prevalence | Hungary | Male | 5 | 1990 | 2021 | 39.011725575425 | 0.97 (0.92 to 1.02) | 0 |
| Prevalence | Iceland | Both | 5 | 1990 | 2021 | 10.680022143185 | 2.63 (2.14 to 3.12) | 0 |
| Prevalence | Iceland | Female | 5 | 1990 | 2021 | 12.725064042592 | 2.46 (2.08 to 2.85) | 0 |
| Prevalence | Iceland | Male | 5 | 1990 | 2021 | 9.986521979 | 2.85 (2.28 to 3.41) | 0 |
| Prevalence | India | Both | 3 | 1990 | 2021 | 28.653647238108 | 1.31 (1.22 to 1.4) | 0 |
| Prevalence | India | Female | 4 | 1990 | 2021 | 24.55513933 | 1.43 (1.31 to 1.54) | 0 |
| Prevalence | India | Male | 4 | 1990 | 2021 | 44.226922158181 | 1.32 (1.26 to 1.38) | 0 |
| Prevalence | Indonesia | Both | 3 | 1990 | 2021 | 32.921741569488 | 0.9 (0.85 to 0.96) | 0 |
| Prevalence | Indonesia | Female | 5 | 1990 | 2021 | 32.027679215667 | 1.15 (1.08 to 1.22) | 0 |
| Prevalence | Indonesia | Male | 3 | 1990 | 2021 | 11.571974495323 | 0.46 (0.38 to 0.53) | 0 |
| Prevalence | Iran (Islamic Republic of) | Both | 5 | 1990 | 2021 | 91.923276865331 | 1.22 (1.2 to 1.25) | 0 |
| Prevalence | Iran (Islamic Republic of) | Female | 5 | 1990 | 2021 | 47.956705475756 | 1.2 (1.15 to 1.25) | 0 |
| Prevalence | Iran (Islamic Republic of) | Male | 4 | 1990 | 2021 | 70.580792218581 | 1.22 (1.19 to 1.26) | 0 |
| Prevalence | Iraq | Both | 5 | 1990 | 2021 | 39.456227516871 | 0.67 (0.64 to 0.71) | 0 |
| Prevalence | Iraq | Female | 5 | 1990 | 2021 | 52.22379769 | 0.88 (0.85 to 0.91) | 0 |
| Prevalence | Iraq | Male | 5 | 1990 | 2021 | 15.640311545371 | 0.29 (0.25 to 0.32) | 0 |
| Prevalence | Ireland | Both | 5 | 1990 | 2021 | 37.515292654606 | 3.02 (2.86 to 3.19) | 0 |
| Prevalence | Ireland | Female | 5 | 1990 | 2021 | 35.936333451714 | 2.97 (2.8 to 3.13) | 0 |
| Prevalence | Ireland | Male | 5 | 1990 | 2021 | 54.520297873303 | 3.14 (3.02 to 3.25) | 0 |
| Prevalence | Israel | Both | 4 | 1990 | 2021 | 27.043176544754 | 3.08 (2.85 to 3.31) | 0 |
| Prevalence | Israel | Female | 4 | 1990 | 2021 | 28.789948391043 | 3.11 (2.89 to 3.32) | 0 |
| Prevalence | Israel | Male | 5 | 1990 | 2021 | 65.461781497386 | 3.04 (2.95 to 3.14) | 0 |
| Prevalence | Italy | Both | 4 | 1990 | 2021 | 28.205564180005 | 2.96 (2.75 to 3.17) | 0 |
| Prevalence | Italy | Female | 5 | 1990 | 2021 | 16.428877385338 | 2.76 (2.42 to 3.09) | 0 |
| Prevalence | Italy | Male | 5 | 1990 | 2021 | 25.466259752115 | 3.58 (3.3 to 3.86) | 0 |
| Prevalence | Jamaica | Both | 4 | 1990 | 2021 | 7.692941761 | 0.86 (0.64 to 1.08) | 0 |
| Prevalence | Jamaica | Female | 5 | 1990 | 2021 | 13.06296371 | 0.93 (0.79 to 1.07) | 0 |
| Prevalence | Jamaica | Male | 5 | 1990 | 2021 | 6.349412029 | 0.73 (0.5 to 0.95) | 0 |
| Prevalence | Japan | Both | 4 | 1990 | 2021 | 15.77433828 | 1.7 (1.49 to 1.91) | 0 |
| Prevalence | Japan | Female | 5 | 1990 | 2021 | 19.830675658009 | 1.84 (1.65 to 2.02) | 0 |
| Prevalence | Japan | Male | 5 | 1990 | 2021 | 19.272797973781 | 1.43 (1.29 to 1.58) | 0 |
| Prevalence | Jordan | Both | 3 | 1990 | 2021 | 24.730652193512 | 0.84 (0.77 to 0.9) | 0 |
| Prevalence | Jordan | Female | 4 | 1990 | 2021 | 93.181972804338 | 0.92 (0.9 to 0.94) | 0 |
| Prevalence | Jordan | Male | 3 | 1990 | 2021 | 25.194576645732 | 0.76 (0.7 to 0.81) | 0 |
| Prevalence | Kazakhstan | Both | 3 | 1990 | 2021 | 4.088903497 | 0.52 (0.27 to 0.78) | 0 |
| Prevalence | Kazakhstan | Female | 3 | 1990 | 2021 | 4.390261997 | 0.62 (0.34 to 0.89) | 0 |
| Prevalence | Kazakhstan | Male | 5 | 1990 | 2021 | 3.933059858 | 0.63 (0.32 to 0.95) | 0 |
| Prevalence | Kenya | Both | 5 | 1990 | 2021 | 6.434563681 | 0.38 (0.27 to 0.5) | 0 |
| Prevalence | Kenya | Female | 5 | 1990 | 2021 | 11.527558410609 | 0.73 (0.61 to 0.86) | 0 |
| Prevalence | Kenya | Male | 5 | 1990 | 2021 | 7.87505795 | 0.37 (0.28 to 0.46) | 0 |
| Prevalence | Kiribati | Both | 3 | 1990 | 2021 | 24.393250695013 | 0.54 (0.5 to 0.59) | 0 |
| Prevalence | Kiribati | Female | 3 | 1990 | 2021 | 17.574846402418 | 0.58 (0.52 to 0.65) | 0 |
| Prevalence | Kiribati | Male | 3 | 1990 | 2021 | 12.358380494822 | 0.19 (0.16 to 0.22) | 0 |
| Prevalence | Kuwait | Both | 3 | 1990 | 2021 | 41.667343866573 | 2.05 (1.95 to 2.14) | 0 |
| Prevalence | Kuwait | Female | 4 | 1990 | 2021 | 44.807405548095 | 2.33 (2.22 to 2.43) | 0 |
| Prevalence | Kuwait | Male | 4 | 1990 | 2021 | 41.577428093585 | 1.78 (1.7 to 1.87) | 0 |
| Prevalence | Kyrgyzstan | Both | 5 | 1990 | 2021 | 7.37454821 | 0.29 (0.21 to 0.37) | 0 |
| Prevalence | Kyrgyzstan | Female | 4 | 1990 | 2021 | -0.058148635 | 0 (-0.1 to 0.09) | 0.954 |
| Prevalence | Kyrgyzstan | Male | 4 | 1990 | 2021 | 14.948976516928 | 1.47 (1.27 to 1.66) | 0 |
| Prevalence | Lao People's Democratic Republic | Both | 5 | 1990 | 2021 | 115.286589446128 | 1.2 (1.18 to 1.22) | 0 |
| Prevalence | Lao People's Democratic Republic | Female | 5 | 1990 | 2021 | 44.952668626999 | 1.2 (1.14 to 1.25) | 0 |
| Prevalence | Lao People's Democratic Republic | Male | 2 | 1990 | 2021 | 41.699717877573 | 1.23 (1.17 to 1.29) | 0 |
| Prevalence | Latvia | Both | 5 | 1990 | 2021 | 15.043087660149 | 1.4 (1.22 to 1.59) | 0 |
| Prevalence | Latvia | Female | 4 | 1990 | 2021 | 15.701502802766 | 1.26 (1.1 to 1.42) | 0 |
| Prevalence | Latvia | Male | 5 | 1990 | 2021 | 11.236570458794 | 2 (1.65 to 2.36) | 0 |
| Prevalence | Lebanon | Both | 5 | 1990 | 2021 | 16.136275225176 | 0.96 (0.84 to 1.08) | 0 |
| Prevalence | Lebanon | Female | 4 | 1990 | 2021 | 15.720482670418 | 0.88 (0.77 to 0.99) | 0 |
| Prevalence | Lebanon | Male | 5 | 1990 | 2021 | 31.458338203153 | 1.03 (0.97 to 1.1) | 0 |
| Prevalence | Lesotho | Both | 5 | 1990 | 2021 | 11.564206535403 | 0.27 (0.22 to 0.32) | 0 |
| Prevalence | Lesotho | Female | 4 | 1990 | 2021 | 26.973719906726 | 0.48 (0.44 to 0.51) | 0 |
| Prevalence | Lesotho | Male | 5 | 1990 | 2021 | -15.57292404 | -0.42 (-0.48 to -0.37) | 0 |
| Prevalence | Liberia | Both | 4 | 1990 | 2021 | 17.649493765845 | 0.66 (0.59 to 0.74) | 0 |
| Prevalence | Liberia | Female | 5 | 1990 | 2021 | 24.533099482141 | 0.59 (0.54 to 0.63) | 0 |
| Prevalence | Liberia | Male | 4 | 1990 | 2021 | 12.809843649525 | 0.68 (0.58 to 0.79) | 0 |
| Prevalence | Libya | Both | 5 | 1990 | 2021 | 48.13982142 | 0.58 (0.55 to 0.6) | 0 |
| Prevalence | Libya | Female | 4 | 1990 | 2021 | 25.706975936681 | 0.77 (0.71 to 0.83) | 0 |
| Prevalence | Libya | Male | 5 | 1990 | 2021 | 6.80563513 | 0.26 (0.18 to 0.33) | 0 |
| Prevalence | Lithuania | Both | 3 | 1990 | 2021 | 13.713323998907 | 2.08 (1.78 to 2.38) | 0 |
| Prevalence | Lithuania | Female | 4 | 1990 | 2021 | 13.402353281889 | 2.03 (1.73 to 2.33) | 0 |
| Prevalence | Lithuania | Male | 5 | 1990 | 2021 | 10.941876459226 | 1.99 (1.63 to 2.35) | 0 |
| Prevalence | Low SDI | Both | 5 | 1990 | 2021 | 24.322191419978 | 0.78 (0.72 to 0.85) | 0 |
| Prevalence | Low SDI | Female | 4 | 1990 | 2021 | 32.202399145599 | 0.66 (0.62 to 0.7) | 0 |
| Prevalence | Low SDI | Male | 5 | 1990 | 2021 | 36.661426065617 | 0.91 (0.86 to 0.96) | 0 |
| Prevalence | Low-middle SDI | Both | 3 | 1990 | 2021 | 39.476370658764 | 1.17 (1.12 to 1.23) | 0 |
| Prevalence | Low-middle SDI | Female | 5 | 1990 | 2021 | 24.463124905752 | 1.17 (1.07 to 1.26) | 0 |
| Prevalence | Low-middle SDI | Male | 3 | 1990 | 2021 | 34.457317250721 | 1.19 (1.12 to 1.26) | 0 |
| Prevalence | Luxembourg | Both | 3 | 1990 | 2021 | 40.923469651055 | 2.68 (2.55 to 2.81) | 0 |
| Prevalence | Luxembourg | Female | 3 | 1990 | 2021 | 32.790894860172 | 2.43 (2.28 to 2.58) | 0 |
| Prevalence | Luxembourg | Male | 4 | 1990 | 2021 | 59.067447160893 | 3.4 (3.29 to 3.52) | 0 |
| Prevalence | Madagascar | Both | 3 | 1990 | 2021 | 27.065692236904 | 0.66 (0.61 to 0.71) | 0 |
| Prevalence | Madagascar | Female | 3 | 1990 | 2021 | 60.365435098396 | 0.69 (0.67 to 0.72) | 0 |
| Prevalence | Madagascar | Male | 4 | 1990 | 2021 | 15.249146769447 | 0.71 (0.62 to 0.8) | 0 |
| Prevalence | Malawi | Both | 4 | 1990 | 2021 | 42.32618794 | 0.87 (0.83 to 0.91) | 0 |
| Prevalence | Malawi | Female | 5 | 1990 | 2021 | 115.35305242108 | 0.97 (0.95 to 0.98) | 0 |
| Prevalence | Malawi | Male | 3 | 1990 | 2021 | 64.878992200703 | 1.05 (1.01 to 1.08) | 0 |
| Prevalence | Malaysia | Both | 4 | 1990 | 2021 | 61.306220617191 | 1.84 (1.78 to 1.9) | 0 |
| Prevalence | Malaysia | Female | 5 | 1990 | 2021 | 120.939143431016 | 2.04 (2.01 to 2.07) | 0 |
| Prevalence | Malaysia | Male | 5 | 1990 | 2021 | 46.433389098415 | 1.58 (1.52 to 1.65) | 0 |
| Prevalence | Maldives | Both | 5 | 1990 | 2021 | 133.182930884579 | 1.91 (1.88 to 1.93) | 0 |
| Prevalence | Maldives | Female | 5 | 1990 | 2021 | 260.176512562283 | 2.16 (2.14 to 2.18) | 0 |
| Prevalence | Maldives | Male | 4 | 1990 | 2021 | 46.505062998807 | 1.91 (1.83 to 1.99) | 0 |
| Prevalence | Mali | Both | 5 | 1990 | 2021 | 20.783521040595 | 0.65 (0.58 to 0.71) | 0 |
| Prevalence | Mali | Female | 2 | 1990 | 2021 | 28.029559188919 | 0.59 (0.54 to 0.63) | 0 |
| Prevalence | Mali | Male | 3 | 1990 | 2021 | 10.833513308431 | 0.7 (0.58 to 0.83) | 0 |
| Prevalence | Malta | Both | 3 | 1990 | 2021 | 6.36319928 | 2.19 (1.51 to 2.88) | 0 |
| Prevalence | Malta | Female | 3 | 1990 | 2021 | 11.173154123787 | 2.13 (1.75 to 2.5) | 0 |
| Prevalence | Malta | Male | 5 | 1990 | 2021 | 6.90743226 | 2.61 (1.86 to 3.36) | 0 |
| Prevalence | Marshall Islands | Both | 5 | 1990 | 2021 | 27.96361264 | 0.65 (0.6 to 0.69) | 0 |
| Prevalence | Marshall Islands | Female | 5 | 1990 | 2021 | 50.359819794289 | 0.79 (0.76 to 0.82) | 0 |
| Prevalence | Marshall Islands | Male | 3 | 1990 | 2021 | 26.646503006938 | 0.58 (0.54 to 0.62) | 0 |
| Prevalence | Mauritania | Both | 5 | 1990 | 2021 | 39.136712221778 | 0.93 (0.89 to 0.98) | 0 |
| Prevalence | Mauritania | Female | 5 | 1990 | 2021 | 98.401781590867 | 0.83 (0.82 to 0.85) | 0 |
| Prevalence | Mauritania | Male | 5 | 1990 | 2021 | 21.460001784189 | 1.05 (0.95 to 1.14) | 0 |
| Prevalence | Mauritius | Both | 5 | 1990 | 2021 | 8.191067986 | 0.87 (0.66 to 1.07) | 0 |
| Prevalence | Mauritius | Female | 5 | 1990 | 2021 | 9.16209991 | 0.78 (0.61 to 0.95) | 0 |
| Prevalence | Mauritius | Male | 5 | 1990 | 2021 | 14.748216661815 | 1.21 (1.05 to 1.37) | 0 |
| Prevalence | Mexico | Both | 5 | 1990 | 2021 | 6.514573751 | 0.59 (0.41 to 0.77) | 0 |
| Prevalence | Mexico | Female | 5 | 1990 | 2021 | 11.339454288351 | 0.87 (0.72 to 1.02) | 0 |
| Prevalence | Mexico | Male | 5 | 1990 | 2021 | 2.981542375 | 0.51 (0.17 to 0.84) | 0.003 |
| Prevalence | Micronesia (Federated States of) | Both | 5 | 1990 | 2021 | 32.701809849818 | 0.71 (0.67 to 0.76) | 0 |
| Prevalence | Micronesia (Federated States of) | Female | 5 | 1990 | 2021 | 40.944920451892 | 0.8 (0.76 to 0.84) | 0 |
| Prevalence | Micronesia (Federated States of) | Male | 2 | 1990 | 2021 | 17.395666985016 | 0.55 (0.48 to 0.61) | 0 |
| Prevalence | Middle SDI | Both | 4 | 1990 | 2021 | 55.791441259096 | 1.22 (1.18 to 1.26) | 0 |
| Prevalence | Middle SDI | Female | 4 | 1990 | 2021 | 61.110900628941 | 1.37 (1.33 to 1.42) | 0 |
| Prevalence | Middle SDI | Male | 5 | 1990 | 2021 | 35.853299696332 | 1 (0.95 to 1.06) | 0 |
| Prevalence | Monaco | Both | 5 | 1990 | 2021 | 33.702338348549 | 1.49 (1.4 to 1.57) | 0 |
| Prevalence | Monaco | Female | 5 | 1990 | 2021 | 31.099577143754 | 1.31 (1.22 to 1.39) | 0 |
| Prevalence | Monaco | Male | 5 | 1990 | 2021 | 40.118004523882 | 1.77 (1.68 to 1.86) | 0 |
| Prevalence | Mongolia | Both | 5 | 1990 | 2021 | 22.468369677494 | 1.14 (1.04 to 1.24) | 0 |
| Prevalence | Mongolia | Female | 5 | 1990 | 2021 | 45.663340256336 | 1.16 (1.11 to 1.21) | 0 |
| Prevalence | Mongolia | Male | 5 | 1990 | 2021 | 35.008591770298 | 1.13 (1.06 to 1.19) | 0 |
| Prevalence | Montenegro | Both | 4 | 1990 | 2021 | 13.70454117 | 0.23 (0.2 to 0.26) | 0 |
| Prevalence | Montenegro | Female | 5 | 1990 | 2021 | 12.552983400564 | 0.49 (0.41 to 0.57) | 0 |
| Prevalence | Montenegro | Male | 4 | 1990 | 2021 | -2.153328007 | -0.04 (-0.08 to 0) | 0.031 |
| Prevalence | Morocco | Both | 5 | 1990 | 2021 | 14.936231385211 | 0.51 (0.44 to 0.57) | 0 |
| Prevalence | Morocco | Female | 2 | 1990 | 2021 | 38.588044086568 | 0.56 (0.53 to 0.59) | 0 |
| Prevalence | Morocco | Male | 5 | 1990 | 2021 | 2.143756241 | 0.27 (0.02 to 0.52) | 0.032 |
| Prevalence | Mozambique | Both | 5 | 1990 | 2021 | 48.031984487456 | 0.73 (0.7 to 0.76) | 0 |
| Prevalence | Mozambique | Female | 5 | 1990 | 2021 | 61.309021809686 | 0.76 (0.74 to 0.79) | 0 |
| Prevalence | Mozambique | Male | 5 | 1990 | 2021 | 56.515184570779 | 0.68 (0.66 to 0.71) | 0 |
| Prevalence | Myanmar | Both | 4 | 1990 | 2021 | 60.16330982 | 1.46 (1.42 to 1.51) | 0 |
| Prevalence | Myanmar | Female | 5 | 1990 | 2021 | 44.672579650865 | 1.5 (1.43 to 1.57) | 0 |
| Prevalence | Myanmar | Male | 4 | 1990 | 2021 | 124.131012815196 | 1.41 (1.38 to 1.43) | 0 |
| Prevalence | Namibia | Both | 4 | 1990 | 2021 | 53.058330456262 | 1.02 (0.98 to 1.06) | 0 |
| Prevalence | Namibia | Female | 4 | 1990 | 2021 | 95.228909837969 | 1.19 (1.16 to 1.21) | 0 |
| Prevalence | Namibia | Male | 3 | 1990 | 2021 | 13.425675832265 | 0.7 (0.6 to 0.8) | 0 |
| Prevalence | Nauru | Both | 5 | 1990 | 2021 | 33.893681075926 | 0.52 (0.49 to 0.55) | 0 |
| Prevalence | Nauru | Female | 5 | 1990 | 2021 | 3.256602705 | 0.15 (0.06 to 0.24) | 0.001 |
| Prevalence | Nauru | Male | 3 | 1990 | 2021 | 7.930835325 | 0.3 (0.23 to 0.38) | 0 |
| Prevalence | Nepal | Both | 2 | 1990 | 2021 | 39.004967204756 | 1.28 (1.22 to 1.35) | 0 |
| Prevalence | Nepal | Female | 3 | 1990 | 2021 | 36.949543307037 | 1.31 (1.24 to 1.38) | 0 |
| Prevalence | Nepal | Male | 2 | 1990 | 2021 | 36.424017339246 | 1.35 (1.28 to 1.42) | 0 |
| Prevalence | Netherlands | Both | 5 | 1990 | 2021 | 14.333361809917 | 2.03 (1.75 to 2.31) | 0 |
| Prevalence | Netherlands | Female | 4 | 1990 | 2021 | 18.976976501467 | 1.59 (1.43 to 1.76) | 0 |
| Prevalence | Netherlands | Male | 5 | 1990 | 2021 | 23.002279255724 | 3.07 (2.81 to 3.34) | 0 |
| Prevalence | New Zealand | Both | 2 | 1990 | 2021 | 17.185686175867 | 1.13 (1 to 1.26) | 0 |
| Prevalence | New Zealand | Female | 2 | 1990 | 2021 | 11.81621845 | 0.83 (0.7 to 0.97) | 0 |
| Prevalence | New Zealand | Male | 5 | 1990 | 2021 | 41.948525047593 | 1.88 (1.8 to 1.97) | 0 |
| Prevalence | Nicaragua | Both | 3 | 1990 | 2021 | 30.287959945188 | 0.61 (0.57 to 0.65) | 0 |
| Prevalence | Nicaragua | Female | 5 | 1990 | 2021 | 92.75240305 | 0.65 (0.63 to 0.66) | 0 |
| Prevalence | Nicaragua | Male | 5 | 1990 | 2021 | 38.991851875245 | 0.61 (0.58 to 0.64) | 0 |
| Prevalence | Niger | Both | 5 | 1990 | 2021 | 14.391385219661 | 0.5 (0.44 to 0.57) | 0 |
| Prevalence | Niger | Female | 5 | 1990 | 2021 | 12.670280374683 | 0.21 (0.17 to 0.24) | 0 |
| Prevalence | Niger | Male | 5 | 1990 | 2021 | 42.357383845549 | 0.6 (0.58 to 0.63) | 0 |
| Prevalence | Nigeria | Both | 5 | 1990 | 2021 | 2.990299331 | 0.04 (0.01 to 0.06) | 0.003 |
| Prevalence | Nigeria | Female | 5 | 1990 | 2021 | 1.065435126 | 0.04 (-0.03 to 0.11) | 0.287 |
| Prevalence | Nigeria | Male | 5 | 1990 | 2021 | -1.191120644 | -0.02 (-0.05 to 0.01) | 0.234 |
| Prevalence | Niue | Both | 4 | 1990 | 2021 | 17.671524730825 | 0.5 (0.45 to 0.56) | 0 |
| Prevalence | Niue | Female | 5 | 1990 | 2021 | 33.92998643 | 0.7 (0.66 to 0.74) | 0 |
| Prevalence | Niue | Male | 2 | 1990 | 2021 | 3.211339468 | 0.1 (0.04 to 0.17) | 0.001 |
| Prevalence | North Africa and Middle East | Both | 4 | 1990 | 2021 | 54.116528823604 | 1.38 (1.33 to 1.43) | 0 |
| Prevalence | North Africa and Middle East | Female | 4 | 1990 | 2021 | 151.428586416157 | 1.43 (1.41 to 1.45) | 0 |
| Prevalence | North Africa and Middle East | Male | 5 | 1990 | 2021 | 96.088088997688 | 1.3 (1.27 to 1.32) | 0 |
| Prevalence | North Macedonia | Both | 5 | 1990 | 2021 | 24.062542708916 | 1 (0.92 to 1.08) | 0 |
| Prevalence | North Macedonia | Female | 3 | 1990 | 2021 | 40.223455626701 | 1.08 (1.03 to 1.13) | 0 |
| Prevalence | North Macedonia | Male | 5 | 1990 | 2021 | 6.051783504 | 0.91 (0.61 to 1.2) | 0 |
| Prevalence | Northern Mariana Islands | Both | 5 | 1990 | 2021 | 6.463273091 | 0.56 (0.39 to 0.74) | 0 |
| Prevalence | Northern Mariana Islands | Female | 5 | 1990 | 2021 | 4.789749141 | 0.76 (0.45 to 1.07) | 0 |
| Prevalence | Northern Mariana Islands | Male | 2 | 1990 | 2021 | 9.267531624 | 0.24 (0.19 to 0.29) | 0 |
| Prevalence | Norway | Both | 3 | 1990 | 2021 | 14.805368608649 | 2.19 (1.9 to 2.49) | 0 |
| Prevalence | Norway | Female | 4 | 1990 | 2021 | 19.86753756 | 1.6 (1.44 to 1.76) | 0 |
| Prevalence | Norway | Male | 3 | 1990 | 2021 | 19.703456835122 | 3.23 (2.91 to 3.56) | 0 |
| Prevalence | Oceania | Both | 5 | 1990 | 2021 | 31.82531191 | 0.57 (0.53 to 0.61) | 0 |
| Prevalence | Oceania | Female | 5 | 1990 | 2021 | 30.642191961267 | 0.61 (0.57 to 0.65) | 0 |
| Prevalence | Oceania | Male | 3 | 1990 | 2021 | 26.32072268 | 0.55 (0.51 to 0.59) | 0 |
| Prevalence | Oman | Both | 5 | 1990 | 2021 | 100.204512365035 | 1.51 (1.48 to 1.54) | 0 |
| Prevalence | Oman | Female | 5 | 1990 | 2021 | 68.995802836196 | 1.77 (1.72 to 1.82) | 0 |
| Prevalence | Oman | Male | 5 | 1990 | 2021 | 37.421958144589 | 0.99 (0.94 to 1.04) | 0 |
| Prevalence | Pakistan | Both | 3 | 1990 | 2021 | 30.779485998558 | 0.69 (0.65 to 0.74) | 0 |
| Prevalence | Pakistan | Female | 3 | 1990 | 2021 | 39.928498269688 | 0.75 (0.71 to 0.79) | 0 |
| Prevalence | Pakistan | Male | 3 | 1990 | 2021 | 28.538630562936 | 0.83 (0.78 to 0.89) | 0 |
| Prevalence | Palau | Both | 4 | 1990 | 2021 | -0.420839428 | -0.03 (-0.17 to 0.11) | 0.674 |
| Prevalence | Palau | Female | 5 | 1990 | 2021 | 6.03770236 | 0.41 (0.28 to 0.54) | 0 |
| Prevalence | Palau | Male | 4 | 1990 | 2021 | -2.358023331 | -0.15 (-0.28 to -0.03) | 0.018 |
| Prevalence | Palestine | Both | 5 | 1990 | 2021 | 25.416964471644 | 1.24 (1.15 to 1.34) | 0 |
| Prevalence | Palestine | Female | 4 | 1990 | 2021 | 94.899294963091 | 1.22 (1.19 to 1.24) | 0 |
| Prevalence | Palestine | Male | 5 | 1990 | 2021 | 56.641555945423 | 1.28 (1.24 to 1.33) | 0 |
| Prevalence | Panama | Both | 5 | 1990 | 2021 | 27.137137990414 | 1.18 (1.09 to 1.27) | 0 |
| Prevalence | Panama | Female | 3 | 1990 | 2021 | 27.419598997332 | 1.33 (1.24 to 1.43) | 0 |
| Prevalence | Panama | Male | 5 | 1990 | 2021 | 19.810388496493 | 1.01 (0.91 to 1.11) | 0 |
| Prevalence | Papua New Guinea | Both | 5 | 1990 | 2021 | 36.748933070908 | 0.58 (0.55 to 0.61) | 0 |
| Prevalence | Papua New Guinea | Female | 3 | 1990 | 2021 | 15.377731230826 | 0.63 (0.55 to 0.71) | 0 |
| Prevalence | Papua New Guinea | Male | 5 | 1990 | 2021 | 13.351231182881 | 0.51 (0.44 to 0.59) | 0 |
| Prevalence | Paraguay | Both | 4 | 1990 | 2021 | 42.504064668714 | 1.11 (1.06 to 1.16) | 0 |
| Prevalence | Paraguay | Female | 5 | 1990 | 2021 | 62.049893680461 | 1.41 (1.36 to 1.45) | 0 |
| Prevalence | Paraguay | Male | 5 | 1990 | 2021 | 51.208526391671 | 0.77 (0.74 to 0.8) | 0 |
| Prevalence | Peru | Both | 5 | 1990 | 2021 | 17.230173455475 | 1.16 (1.03 to 1.29) | 0 |
| Prevalence | Peru | Female | 5 | 1990 | 2021 | 26.290149982621 | 1.24 (1.14 to 1.33) | 0 |
| Prevalence | Peru | Male | 5 | 1990 | 2021 | 14.222101167558 | 1.08 (0.93 to 1.23) | 0 |
| Prevalence | Philippines | Both | 5 | 1990 | 2021 | 19.579393427951 | 0.63 (0.57 to 0.7) | 0 |
| Prevalence | Philippines | Female | 5 | 1990 | 2021 | 18.012680653106 | 0.79 (0.71 to 0.88) | 0 |
| Prevalence | Philippines | Male | 5 | 1990 | 2021 | 27.98618941 | 0.4 (0.37 to 0.42) | 0 |
| Prevalence | Poland | Both | 5 | 1990 | 2021 | 20.013045611217 | 1.31 (1.18 to 1.43) | 0 |
| Prevalence | Poland | Female | 4 | 1990 | 2021 | 12.740978071438 | 1.5 (1.26 to 1.73) | 0 |
| Prevalence | Poland | Male | 4 | 1990 | 2021 | 11.202117786498 | 1.09 (0.9 to 1.28) | 0 |
| Prevalence | Portugal | Both | 5 | 1990 | 2021 | 40.203511891334 | 1.96 (1.86 to 2.05) | 0 |
| Prevalence | Portugal | Female | 5 | 1990 | 2021 | 26.311341685913 | 1.64 (1.52 to 1.77) | 0 |
| Prevalence | Portugal | Male | 5 | 1990 | 2021 | 51.138429169691 | 2.25 (2.17 to 2.34) | 0 |
| Prevalence | Puerto Rico | Both | 5 | 1990 | 2021 | 41.432558031247 | 1.38 (1.31 to 1.45) | 0 |
| Prevalence | Puerto Rico | Female | 5 | 1990 | 2021 | 19.823808181443 | 1.4 (1.26 to 1.54) | 0 |
| Prevalence | Puerto Rico | Male | 4 | 1990 | 2021 | 38.599040347433 | 1.37 (1.3 to 1.44) | 0 |
| Prevalence | Qatar | Both | 5 | 1990 | 2021 | 18.041405126459 | 1.7 (1.51 to 1.88) | 0 |
| Prevalence | Qatar | Female | 5 | 1990 | 2021 | 39.712172446257 | 1.4 (1.33 to 1.47) | 0 |
| Prevalence | Qatar | Male | 5 | 1990 | 2021 | 12.668647075111 | 1.79 (1.51 to 2.07) | 0 |
| Prevalence | Republic of Korea | Both | 4 | 1990 | 2021 | 47.14091678 | 4.38 (4.19 to 4.56) | 0 |
| Prevalence | Republic of Korea | Female | 5 | 1990 | 2021 | 56.476335743357 | 4.27 (4.12 to 4.42) | 0 |
| Prevalence | Republic of Korea | Male | 5 | 1990 | 2021 | 35.069601365686 | 4.55 (4.29 to 4.81) | 0 |
| Prevalence | Republic of Moldova | Both | 5 | 1990 | 2021 | 7.925306905 | 1.85 (1.39 to 2.31) | 0 |
| Prevalence | Republic of Moldova | Female | 5 | 1990 | 2021 | 6.215012469 | 1.64 (1.12 to 2.17) | 0 |
| Prevalence | Republic of Moldova | Male | 5 | 1990 | 2021 | 7.905349031 | 2.24 (1.68 to 2.81) | 0 |
| Prevalence | Romania | Both | 5 | 1990 | 2021 | 23.606080938315 | 1.11 (1.02 to 1.2) | 0 |
| Prevalence | Romania | Female | 5 | 1990 | 2021 | 26.801270230337 | 1.36 (1.26 to 1.47) | 0 |
| Prevalence | Romania | Male | 5 | 1990 | 2021 | 19.538401213238 | 0.93 (0.84 to 1.03) | 0 |
| Prevalence | Russian Federation | Both | 5 | 1990 | 2021 | 13.470196406039 | 1.14 (0.98 to 1.31) | 0 |
| Prevalence | Russian Federation | Female | 4 | 1990 | 2021 | 11.232163346478 | 1.1 (0.91 to 1.29) | 0 |
| Prevalence | Russian Federation | Male | 4 | 1990 | 2021 | 21.130556085023 | 1.66 (1.5 to 1.81) | 0 |
| Prevalence | Rwanda | Both | 5 | 1990 | 2021 | 30.520636651253 | 0.66 (0.61 to 0.7) | 0 |
| Prevalence | Rwanda | Female | 5 | 1990 | 2021 | 19.367779161318 | 0.46 (0.42 to 0.51) | 0 |
| Prevalence | Rwanda | Male | 4 | 1990 | 2021 | 20.004663921191 | 0.95 (0.86 to 1.04) | 0 |
| Prevalence | Saint Kitts and Nevis | Both | 4 | 1990 | 2021 | 7.977374299 | 0.68 (0.51 to 0.84) | 0 |
| Prevalence | Saint Kitts and Nevis | Female | 4 | 1990 | 2021 | 7.964041623 | 0.61 (0.46 to 0.77) | 0 |
| Prevalence | Saint Kitts and Nevis | Male | 5 | 1990 | 2021 | 8.571255787 | 0.6 (0.46 to 0.74) | 0 |
| Prevalence | Saint Lucia | Both | 5 | 1990 | 2021 | 11.67126422 | 0.56 (0.47 to 0.66) | 0 |
| Prevalence | Saint Lucia | Female | 5 | 1990 | 2021 | 11.392517620003 | 0.6 (0.5 to 0.71) | 0 |
| Prevalence | Saint Lucia | Male | 3 | 1990 | 2021 | 5.425785696 | 0.41 (0.26 to 0.56) | 0 |
| Prevalence | Saint Vincent and the Grenadines | Both | 5 | 1990 | 2021 | 1.045003445 | 0.09 (-0.07 to 0.25) | 0.296 |
| Prevalence | Saint Vincent and the Grenadines | Female | 5 | 1990 | 2021 | 3.332214423 | 0.11 (0.05 to 0.18) | 0.001 |
| Prevalence | Saint Vincent and the Grenadines | Male | 5 | 1990 | 2021 | -6.439493717 | -0.36 (-0.47 to -0.25) | 0 |
| Prevalence | Samoa | Both | 3 | 1990 | 2021 | 13.086863214988 | 0.46 (0.39 to 0.53) | 0 |
| Prevalence | Samoa | Female | 5 | 1990 | 2021 | 26.983562433403 | 0.57 (0.53 to 0.62) | 0 |
| Prevalence | Samoa | Male | 2 | 1990 | 2021 | 8.300551513 | 0.32 (0.24 to 0.4) | 0 |
| Prevalence | San Marino | Both | 5 | 1990 | 2021 | 37.290528150901 | 0.96 (0.91 to 1.01) | 0 |
| Prevalence | San Marino | Female | 4 | 1990 | 2021 | 14.031082980508 | 0.6 (0.52 to 0.69) | 0 |
| Prevalence | San Marino | Male | 5 | 1990 | 2021 | 52.929072977472 | 1.4 (1.35 to 1.46) | 0 |
| Prevalence | Sao Tome and Principe | Both | 4 | 1990 | 2021 | 45.768332148586 | 1.09 (1.04 to 1.14) | 0 |
| Prevalence | Sao Tome and Principe | Female | 4 | 1990 | 2021 | 40.073144762572 | 1.1 (1.05 to 1.16) | 0 |
| Prevalence | Sao Tome and Principe | Male | 4 | 1990 | 2021 | 53.291285748041 | 1.05 (1.01 to 1.09) | 0 |
| Prevalence | Saudi Arabia | Both | 4 | 1990 | 2021 | 46.622637975126 | 1.04 (0.99 to 1.08) | 0 |
| Prevalence | Saudi Arabia | Female | 5 | 1990 | 2021 | 38.486014785924 | 1.39 (1.32 to 1.46) | 0 |
| Prevalence | Saudi Arabia | Male | 5 | 1990 | 2021 | 35.620653471246 | 0.75 (0.7 to 0.79) | 0 |
| Prevalence | Senegal | Both | 5 | 1990 | 2021 | 20.16951364 | 0.85 (0.77 to 0.94) | 0 |
| Prevalence | Senegal | Female | 5 | 1990 | 2021 | 47.927862234145 | 0.74 (0.71 to 0.77) | 0 |
| Prevalence | Senegal | Male | 5 | 1990 | 2021 | 25.480526767294 | 0.92 (0.85 to 0.99) | 0 |
| Prevalence | Serbia | Both | 3 | 1990 | 2021 | 2.727854224 | 1.03 (0.29 to 1.77) | 0.006 |
| Prevalence | Serbia | Female | 3 | 1990 | 2021 | 2.81855173 | 0.92 (0.28 to 1.57) | 0.005 |
| Prevalence | Serbia | Male | 3 | 1990 | 2021 | 2.453347638 | 1.12 (0.22 to 2.02) | 0.014 |
| Prevalence | Seychelles | Both | 5 | 1990 | 2021 | 15.868071526647 | 1.06 (0.93 to 1.19) | 0 |
| Prevalence | Seychelles | Female | 3 | 1990 | 2021 | 50.378075647698 | 1.16 (1.12 to 1.21) | 0 |
| Prevalence | Seychelles | Male | 5 | 1990 | 2021 | 11.362243051684 | 0.84 (0.69 to 0.99) | 0 |
| Prevalence | Sierra Leone | Both | 5 | 1990 | 2021 | 42.671215172905 | 0.89 (0.85 to 0.93) | 0 |
| Prevalence | Sierra Leone | Female | 5 | 1990 | 2021 | 40.327183294466 | 0.74 (0.7 to 0.77) | 0 |
| Prevalence | Sierra Leone | Male | 5 | 1990 | 2021 | 41.715218907325 | 1.02 (0.97 to 1.07) | 0 |
| Prevalence | Singapore | Both | 3 | 1990 | 2021 | 16.606419322648 | 2.76 (2.43 to 3.09) | 0 |
| Prevalence | Singapore | Female | 4 | 1990 | 2021 | 22.091500511201 | 2.85 (2.6 to 3.11) | 0 |
| Prevalence | Singapore | Male | 2 | 1990 | 2021 | 18.107448476224 | 2.57 (2.29 to 2.85) | 0 |
| Prevalence | Slovakia | Both | 3 | 1990 | 2021 | 7.019682944 | 1.52 (1.09 to 1.95) | 0 |
| Prevalence | Slovakia | Female | 3 | 1990 | 2021 | 6.358007753 | 1.38 (0.96 to 1.81) | 0 |
| Prevalence | Slovakia | Male | 2 | 1990 | 2021 | 12.508857539788 | 1.96 (1.65 to 2.27) | 0 |
| Prevalence | Slovenia | Both | 5 | 1990 | 2021 | 43.678732462468 | 2.83 (2.7 to 2.96) | 0 |
| Prevalence | Slovenia | Female | 5 | 1990 | 2021 | 27.259860075293 | 2.6 (2.41 to 2.79) | 0 |
| Prevalence | Slovenia | Male | 5 | 1990 | 2021 | 51.254178317797 | 3.37 (3.24 to 3.5) | 0 |
| Prevalence | Solomon Islands | Both | 3 | 1990 | 2021 | 22.992914127599 | 0.73 (0.66 to 0.79) | 0 |
| Prevalence | Solomon Islands | Female | 2 | 1990 | 2021 | 12.20126952 | 0.66 (0.56 to 0.77) | 0 |
| Prevalence | Solomon Islands | Male | 2 | 1990 | 2021 | 36.31250558 | 0.83 (0.79 to 0.88) | 0 |
| Prevalence | Somalia | Both | 4 | 1990 | 2021 | -16.97762293 | -0.21 (-0.23 to -0.18) | 0 |
| Prevalence | Somalia | Female | 4 | 1990 | 2021 | -28.24587022 | -0.19 (-0.2 to -0.17) | 0 |
| Prevalence | Somalia | Male | 2 | 1990 | 2021 | -6.081431518 | -0.1 (-0.14 to -0.07) | 0 |
| Prevalence | South Africa | Both | 5 | 1990 | 2021 | 10.140218964735 | 0.36 (0.29 to 0.43) | 0 |
| Prevalence | South Africa | Female | 5 | 1990 | 2021 | 12.336251074385 | 0.47 (0.39 to 0.54) | 0 |
| Prevalence | South Africa | Male | 3 | 1990 | 2021 | 7.810597375 | 0.15 (0.11 to 0.18) | 0 |
| Prevalence | South Asia | Both | 2 | 1990 | 2021 | 51.894064244784 | 1.33 (1.28 to 1.38) | 0 |
| Prevalence | South Asia | Female | 5 | 1990 | 2021 | 25.017644772441 | 1.45 (1.34 to 1.57) | 0 |
| Prevalence | South Asia | Male | 1 | 1990 | 2021 | 63.924115090088 | 1.33 (1.29 to 1.38) | 0 |
| Prevalence | South Sudan | Both | 4 | 1990 | 2021 | -2.892922266 | -0.05 (-0.09 to -0.02) | 0.004 |
| Prevalence | South Sudan | Female | 5 | 1990 | 2021 | -9.266995003 | -0.15 (-0.18 to -0.12) | 0 |
| Prevalence | South Sudan | Male | 3 | 1990 | 2021 | -4.354884083 | -0.11 (-0.15 to -0.06) | 0 |
| Prevalence | Southeast Asia | Both | 3 | 1990 | 2021 | 93.062903003552 | 1.65 (1.61 to 1.68) | 0 |
| Prevalence | Southeast Asia | Female | 5 | 1990 | 2021 | 70.419432327947 | 1.68 (1.63 to 1.73) | 0 |
| Prevalence | Southeast Asia | Male | 3 | 1990 | 2021 | 52.482421179806 | 1.6 (1.54 to 1.66) | 0 |
| Prevalence | Southern Latin America | Both | 5 | 1990 | 2021 | 34.579277327633 | 1.78 (1.68 to 1.89) | 0 |
| Prevalence | Southern Latin America | Female | 5 | 1990 | 2021 | 26.481558324014 | 1.69 (1.56 to 1.82) | 0 |
| Prevalence | Southern Latin America | Male | 5 | 1990 | 2021 | 35.363463138569 | 1.91 (1.81 to 2.02) | 0 |
| Prevalence | Southern Sub-Saharan Africa | Both | 4 | 1990 | 2021 | 10.059863813462 | 0.39 (0.31 to 0.47) | 0 |
| Prevalence | Southern Sub-Saharan Africa | Female | 5 | 1990 | 2021 | 11.285830137472 | 0.5 (0.41 to 0.59) | 0 |
| Prevalence | Southern Sub-Saharan Africa | Male | 3 | 1990 | 2021 | 7.360458928 | 0.16 (0.12 to 0.21) | 0 |
| Prevalence | Spain | Both | 5 | 1990 | 2021 | 23.022584862743 | 2.03 (1.86 to 2.2) | 0 |
| Prevalence | Spain | Female | 5 | 1990 | 2021 | 17.211007387355 | 2.18 (1.93 to 2.43) | 0 |
| Prevalence | Spain | Male | 4 | 1990 | 2021 | 17.423430508305 | 1.83 (1.62 to 2.04) | 0 |
| Prevalence | Sri Lanka | Both | 4 | 1990 | 2021 | 50.755653410311 | 2.24 (2.15 to 2.33) | 0 |
| Prevalence | Sri Lanka | Female | 5 | 1990 | 2021 | 64.648116548189 | 2.18 (2.11 to 2.24) | 0 |
| Prevalence | Sri Lanka | Male | 5 | 1990 | 2021 | 52.137982046289 | 2.11 (2.03 to 2.2) | 0 |
| Prevalence | Sudan | Both | 5 | 1990 | 2021 | 24.826429267787 | 0.92 (0.85 to 0.99) | 0 |
| Prevalence | Sudan | Female | 3 | 1990 | 2021 | 28.627450728613 | 1.28 (1.19 to 1.37) | 0 |
| Prevalence | Sudan | Male | 5 | 1990 | 2021 | 17.837892833927 | 0.56 (0.5 to 0.62) | 0 |
| Prevalence | Suriname | Both | 5 | 1990 | 2021 | 19.772478463935 | 0.8 (0.72 to 0.88) | 0 |
| Prevalence | Suriname | Female | 5 | 1990 | 2021 | 43.983472930064 | 0.92 (0.88 to 0.96) | 0 |
| Prevalence | Suriname | Male | 5 | 1990 | 2021 | 20.301203017747 | 0.71 (0.64 to 0.77) | 0 |
| Prevalence | Sweden | Both | 5 | 1990 | 2021 | 36.505768361463 | 3.63 (3.44 to 3.83) | 0 |
| Prevalence | Sweden | Female | 5 | 1990 | 2021 | 29.160054388974 | 3.26 (3.04 to 3.48) | 0 |
| Prevalence | Sweden | Male | 4 | 1990 | 2021 | 71.283134255849 | 4.25 (4.13 to 4.37) | 0 |
| Prevalence | Switzerland | Both | 5 | 1990 | 2021 | 38.683082759779 | 3.05 (2.89 to 3.21) | 0 |
| Prevalence | Switzerland | Female | 5 | 1990 | 2021 | 36.267568316367 | 2.7 (2.55 to 2.84) | 0 |
| Prevalence | Switzerland | Male | 3 | 1990 | 2021 | 25.996329295508 | 3.71 (3.42 to 3.99) | 0 |
| Prevalence | Syrian Arab Republic | Both | 5 | 1990 | 2021 | 10.999082548688 | 0.64 (0.53 to 0.76) | 0 |
| Prevalence | Syrian Arab Republic | Female | 5 | 1990 | 2021 | 36.31717226 | 0.81 (0.77 to 0.86) | 0 |
| Prevalence | Syrian Arab Republic | Male | 5 | 1990 | 2021 | 7.286028901 | 0.46 (0.33 to 0.58) | 0 |
| Prevalence | Taiwan (Province of China) | Both | 5 | 1990 | 2021 | 12.341449566243 | 1.85 (1.56 to 2.15) | 0 |
| Prevalence | Taiwan (Province of China) | Female | 5 | 1990 | 2021 | 27.195472321768 | 1.92 (1.78 to 2.06) | 0 |
| Prevalence | Taiwan (Province of China) | Male | 4 | 1990 | 2021 | 12.571731669671 | 1.62 (1.37 to 1.88) | 0 |
| Prevalence | Tajikistan | Both | 5 | 1990 | 2021 | -0.562591234 | -0.07 (-0.3 to 0.17) | 0.574 |
| Prevalence | Tajikistan | Female | 5 | 1990 | 2021 | -2.531717323 | -0.1 (-0.17 to -0.02) | 0.011 |
| Prevalence | Tajikistan | Male | 5 | 1990 | 2021 | 0.20610971 | 0.04 (-0.38 to 0.47) | 0.837 |
| Prevalence | Thailand | Both | 3 | 1990 | 2021 | 42.726222498546 | 1.42 (1.36 to 1.49) | 0 |
| Prevalence | Thailand | Female | 3 | 1990 | 2021 | 57.458106466993 | 1.42 (1.38 to 1.47) | 0 |
| Prevalence | Thailand | Male | 3 | 1990 | 2021 | 30.948748313034 | 1.44 (1.34 to 1.53) | 0 |
| Prevalence | Timor-Leste | Both | 4 | 1990 | 2021 | 16.527711897001 | 0.53 (0.47 to 0.59) | 0 |
| Prevalence | Timor-Leste | Female | 3 | 1990 | 2021 | 22.086647679543 | 0.74 (0.68 to 0.81) | 0 |
| Prevalence | Timor-Leste | Male | 2 | 1990 | 2021 | 4.892381012 | 0.26 (0.15 to 0.36) | 0 |
| Prevalence | Togo | Both | 5 | 1990 | 2021 | 32.615505901898 | 0.61 (0.57 to 0.64) | 0 |
| Prevalence | Togo | Female | 5 | 1990 | 2021 | 39.930973842616 | 0.59 (0.56 to 0.62) | 0 |
| Prevalence | Togo | Male | 5 | 1990 | 2021 | 6.383439985 | 0.57 (0.39 to 0.74) | 0 |
| Prevalence | Tokelau | Both | 1 | 1990 | 2021 | 57.607352 | 0.73 (0.71 to 0.76) | 0 |
| Prevalence | Tokelau | Female | 5 | 1990 | 2021 | 29.315351739232 | 0.91 (0.85 to 0.98) | 0 |
| Prevalence | Tokelau | Male | 1 | 1990 | 2021 | 25.740184525204 | 0.51 (0.47 to 0.55) | 0 |
| Prevalence | Tonga | Both | 3 | 1990 | 2021 | 18.262956867933 | 0.71 (0.64 to 0.79) | 0 |
| Prevalence | Tonga | Female | 5 | 1990 | 2021 | 9.54498711 | 0.67 (0.54 to 0.81) | 0 |
| Prevalence | Tonga | Male | 4 | 1990 | 2021 | 10.248885625952 | 0.74 (0.6 to 0.88) | 0 |
| Prevalence | Trinidad and Tobago | Both | 3 | 1990 | 2021 | 35.816437497921 | 1.62 (1.53 to 1.71) | 0 |
| Prevalence | Trinidad and Tobago | Female | 3 | 1990 | 2021 | 25.772918889498 | 1.46 (1.35 to 1.57) | 0 |
| Prevalence | Trinidad and Tobago | Male | 5 | 1990 | 2021 | 64.880827889805 | 1.88 (1.82 to 1.93) | 0 |
| Prevalence | Tropical Latin America | Both | 5 | 1990 | 2021 | 60.413919262904 | 1.96 (1.9 to 2.03) | 0 |
| Prevalence | Tropical Latin America | Female | 5 | 1990 | 2021 | 86.91783185 | 2.12 (2.07 to 2.17) | 0 |
| Prevalence | Tropical Latin America | Male | 4 | 1990 | 2021 | 57.52961791 | 1.75 (1.69 to 1.81) | 0 |
| Prevalence | Tunisia | Both | 5 | 1990 | 2021 | 39.773617951081 | 0.94 (0.89 to 0.98) | 0 |
| Prevalence | Tunisia | Female | 5 | 1990 | 2021 | 51.705325073201 | 0.97 (0.94 to 1.01) | 0 |
| Prevalence | Tunisia | Male | 4 | 1990 | 2021 | 16.194615441365 | 0.78 (0.69 to 0.88) | 0 |
| Prevalence | Turkey | Both | 5 | 1990 | 2021 | 20.403092787288 | 1.45 (1.31 to 1.59) | 0 |
| Prevalence | Turkey | Female | 5 | 1990 | 2021 | 24.060060770473 | 1.37 (1.25 to 1.48) | 0 |
| Prevalence | Turkey | Male | 5 | 1990 | 2021 | 15.820915968563 | 1.58 (1.39 to 1.78) | 0 |
| Prevalence | Turkmenistan | Both | 5 | 1990 | 2021 | 4.929354997 | 1.05 (0.63 to 1.47) | 0 |
| Prevalence | Turkmenistan | Female | 5 | 1990 | 2021 | 5.820864362 | 0.99 (0.65 to 1.32) | 0 |
| Prevalence | Turkmenistan | Male | 5 | 1990 | 2021 | 6.335678583 | 1.13 (0.78 to 1.49) | 0 |
| Prevalence | Tuvalu | Both | 4 | 1990 | 2021 | 42.10636885 | 0.95 (0.9 to 0.99) | 0 |
| Prevalence | Tuvalu | Female | 5 | 1990 | 2021 | 87.380425699003 | 1.09 (1.07 to 1.12) | 0 |
| Prevalence | Tuvalu | Male | 2 | 1990 | 2021 | 19.404143693482 | 0.79 (0.71 to 0.87) | 0 |
| Prevalence | Uganda | Both | 5 | 1990 | 2021 | 26.166421347752 | 0.68 (0.63 to 0.74) | 0 |
| Prevalence | Uganda | Female | 5 | 1990 | 2021 | 29.640992191131 | 0.81 (0.76 to 0.86) | 0 |
| Prevalence | Uganda | Male | 5 | 1990 | 2021 | 22.581239835955 | 0.66 (0.6 to 0.71) | 0 |
| Prevalence | Ukraine | Both | 5 | 1990 | 2021 | -0.218642379 | -0.02 (-0.18 to 0.14) | 0.827 |
| Prevalence | Ukraine | Female | 4 | 1990 | 2021 | -0.9687103 | -0.11 (-0.34 to 0.12) | 0.333 |
| Prevalence | Ukraine | Male | 5 | 1990 | 2021 | 6.908748304 | 0.52 (0.37 to 0.67) | 0 |
| Prevalence | United Arab Emirates | Both | 5 | 1990 | 2021 | 46.316682442636 | 0.86 (0.83 to 0.9) | 0 |
| Prevalence | United Arab Emirates | Female | 5 | 1990 | 2021 | 39.345872365728 | 1.38 (1.31 to 1.45) | 0 |
| Prevalence | United Arab Emirates | Male | 5 | 1990 | 2021 | 64.085772420208 | 1.22 (1.18 to 1.25) | 0 |
| Prevalence | United Kingdom | Both | 5 | 1990 | 2021 | 27.485283410098 | 2.43 (2.25 to 2.6) | 0 |
| Prevalence | United Kingdom | Female | 5 | 1990 | 2021 | 29.952169893681 | 2.46 (2.3 to 2.63) | 0 |
| Prevalence | United Kingdom | Male | 5 | 1990 | 2021 | 63.949229888067 | 2.84 (2.75 to 2.93) | 0 |
| Prevalence | United Republic of Tanzania | Both | 3 | 1990 | 2021 | 10.616099031174 | 0.53 (0.43 to 0.63) | 0 |
| Prevalence | United Republic of Tanzania | Female | 4 | 1990 | 2021 | 13.232514486724 | 0.57 (0.48 to 0.65) | 0 |
| Prevalence | United Republic of Tanzania | Male | 3 | 1990 | 2021 | 15.277241737568 | 0.53 (0.46 to 0.6) | 0 |
| Prevalence | United States Virgin Islands | Both | 3 | 1990 | 2021 | 24.067431214289 | 0.59 (0.54 to 0.63) | 0 |
| Prevalence | United States Virgin Islands | Female | 4 | 1990 | 2021 | 54.292236876003 | 0.94 (0.9 to 0.97) | 0 |
| Prevalence | United States Virgin Islands | Male | 3 | 1990 | 2021 | 1.237151464 | 0.06 (-0.04 to 0.16) | 0.216 |
| Prevalence | United States of America | Both | 3 | 1990 | 2021 | 43.6720045 | 2.07 (1.98 to 2.17) | 0 |
| Prevalence | United States of America | Female | 5 | 1990 | 2021 | 49.82192109 | 2.04 (1.96 to 2.12) | 0 |
| Prevalence | United States of America | Male | 4 | 1990 | 2021 | 34.756980129103 | 2 (1.88 to 2.11) | 0 |
| Prevalence | Uruguay | Both | 5 | 1990 | 2021 | 26.733681739666 | 1.58 (1.47 to 1.7) | 0 |
| Prevalence | Uruguay | Female | 5 | 1990 | 2021 | 20.37458692 | 1.47 (1.33 to 1.62) | 0 |
| Prevalence | Uruguay | Male | 5 | 1990 | 2021 | 53.265713371901 | 1.8 (1.74 to 1.87) | 0 |
| Prevalence | Uzbekistan | Both | 4 | 1990 | 2021 | 2.440618088 | 0.48 (0.1 to 0.88) | 0.015 |
| Prevalence | Uzbekistan | Female | 5 | 1990 | 2021 | 5.036810328 | 0.38 (0.23 to 0.53) | 0 |
| Prevalence | Uzbekistan | Male | 5 | 1990 | 2021 | 8.208057515 | 0.65 (0.49 to 0.8) | 0 |
| Prevalence | Vanuatu | Both | 3 | 1990 | 2021 | 51.111801319136 | 0.53 (0.51 to 0.56) | 0 |
| Prevalence | Vanuatu | Female | 5 | 1990 | 2021 | 55.676488752274 | 0.53 (0.51 to 0.55) | 0 |
| Prevalence | Vanuatu | Male | 2 | 1990 | 2021 | 26.044919287038 | 0.44 (0.41 to 0.48) | 0 |
| Prevalence | Venezuela (Bolivarian Republic of) | Both | 3 | 1990 | 2021 | 12.927241765701 | 0.65 (0.55 to 0.75) | 0 |
| Prevalence | Venezuela (Bolivarian Republic of) | Female | 2 | 1990 | 2021 | 20.539715849577 | 0.83 (0.75 to 0.91) | 0 |
| Prevalence | Venezuela (Bolivarian Republic of) | Male | 4 | 1990 | 2021 | 8.592861664 | 0.44 (0.34 to 0.55) | 0 |
| Prevalence | Viet Nam | Both | 5 | 1990 | 2021 | 96.070169841925 | 1.44 (1.41 to 1.47) | 0 |
| Prevalence | Viet Nam | Female | 5 | 1990 | 2021 | 78.679851763887 | 1.61 (1.57 to 1.65) | 0 |
| Prevalence | Viet Nam | Male | 2 | 1990 | 2021 | 66.832883245614 | 1.06 (1.03 to 1.09) | 0 |
| Prevalence | Western Europe | Both | 3 | 1990 | 2021 | 35.456835675044 | 2.45 (2.32 to 2.59) | 0 |
| Prevalence | Western Europe | Female | 4 | 1990 | 2021 | 35.51891108 | 2.33 (2.2 to 2.46) | 0 |
| Prevalence | Western Europe | Male | 3 | 1990 | 2021 | 21.022924958975 | 2.77 (2.51 to 3.03) | 0 |
| Prevalence | Western Sub-Saharan Africa | Both | 4 | 1990 | 2021 | 52.638989832684 | 0.38 (0.37 to 0.4) | 0 |
| Prevalence | Western Sub-Saharan Africa | Female | 5 | 1990 | 2021 | 25.032275880593 | 0.43 (0.4 to 0.47) | 0 |
| Prevalence | Western Sub-Saharan Africa | Male | 5 | 1990 | 2021 | 19.885864542464 | 0.3 (0.27 to 0.33) | 0 |
| Prevalence | Yemen | Both | 5 | 1990 | 2021 | 63.646151067332 | 1.05 (1.01 to 1.08) | 0 |
| Prevalence | Yemen | Female | 4 | 1990 | 2021 | 54.735570431082 | 1.06 (1.02 to 1.1) | 0 |
| Prevalence | Yemen | Male | 5 | 1990 | 2021 | 24.358013855712 | 0.74 (0.68 to 0.8) | 0 |
| Prevalence | Zambia | Both | 5 | 1990 | 2021 | 22.283219916449 | 1.27 (1.16 to 1.39) | 0 |
| Prevalence | Zambia | Female | 5 | 1990 | 2021 | 25.897213717373 | 1.29 (1.2 to 1.39) | 0 |
| Prevalence | Zambia | Male | 5 | 1990 | 2021 | 36.959821630327 | 1.17 (1.11 to 1.23) | 0 |
| Prevalence | Zimbabwe | Both | 4 | 1990 | 2021 | 0.84431856 | 0.03 (-0.04 to 0.09) | 0.398 |
| Prevalence | Zimbabwe | Female | 4 | 1990 | 2021 | 4.943898713 | 0.14 (0.08 to 0.19) | 0 |
| Prevalence | Zimbabwe | Male | 4 | 1990 | 2021 | -8.51452614 | -0.27 (-0.33 to -0.21) | 0 |
| YLDs (Years Lived with Disability) | Afghanistan | Both | 5 | 1990 | 2021 | 8.803245024 | 0.67 (0.52 to 0.82) | 0 |
| YLDs (Years Lived with Disability) | Afghanistan | Female | 2 | 1990 | 2021 | 14.960368020049 | 0.71 (0.61 to 0.8) | 0 |
| YLDs (Years Lived with Disability) | Afghanistan | Male | 5 | 1990 | 2021 | 7.388261392 | 0.58 (0.42 to 0.73) | 0 |
| YLDs (Years Lived with Disability) | Albania | Both | 4 | 1990 | 2021 | 25.320961549678 | 1.18 (1.09 to 1.28) | 0 |
| YLDs (Years Lived with Disability) | Albania | Female | 5 | 1990 | 2021 | 14.203519919847 | 1.21 (1.04 to 1.37) | 0 |
| YLDs (Years Lived with Disability) | Albania | Male | 4 | 1990 | 2021 | 10.625361561834 | 1.3 (1.06 to 1.54) | 0 |
| YLDs (Years Lived with Disability) | Algeria | Both | 4 | 1990 | 2021 | 26.587767369236 | 1.31 (1.21 to 1.41) | 0 |
| YLDs (Years Lived with Disability) | Algeria | Female | 4 | 1990 | 2021 | 14.776258296195 | 1.21 (1.05 to 1.37) | 0 |
| YLDs (Years Lived with Disability) | Algeria | Male | 4 | 1990 | 2021 | 23.173569549372 | 1.42 (1.3 to 1.55) | 0 |
| YLDs (Years Lived with Disability) | American Samoa | Both | 5 | 1990 | 2021 | 8.123367756 | 0.45 (0.34 to 0.55) | 0 |
| YLDs (Years Lived with Disability) | American Samoa | Female | 5 | 1990 | 2021 | 6.203260277 | 0.36 (0.25 to 0.47) | 0 |
| YLDs (Years Lived with Disability) | American Samoa | Male | 3 | 1990 | 2021 | 6.754923569 | 0.65 (0.46 to 0.84) | 0 |
| YLDs (Years Lived with Disability) | Andean Latin America | Both | 5 | 1990 | 2021 | 22.71003951 | 0.94 (0.86 to 1.03) | 0 |
| YLDs (Years Lived with Disability) | Andean Latin America | Female | 5 | 1990 | 2021 | 24.123232955277 | 1.14 (1.05 to 1.24) | 0 |
| YLDs (Years Lived with Disability) | Andean Latin America | Male | 4 | 1990 | 2021 | 14.220736408243 | 0.77 (0.66 to 0.88) | 0 |
| YLDs (Years Lived with Disability) | Andorra | Both | 5 | 1990 | 2021 | 17.623592238173 | 1.09 (0.96 to 1.21) | 0 |
| YLDs (Years Lived with Disability) | Andorra | Female | 3 | 1990 | 2021 | 19.756788503072 | 0.86 (0.77 to 0.94) | 0 |
| YLDs (Years Lived with Disability) | Andorra | Male | 3 | 1990 | 2021 | 22.363210594128 | 1.24 (1.13 to 1.34) | 0 |
| YLDs (Years Lived with Disability) | Angola | Both | 5 | 1990 | 2021 | 44.630946185858 | 1.28 (1.22 to 1.34) | 0 |
| YLDs (Years Lived with Disability) | Angola | Female | 5 | 1990 | 2021 | 23.743727697237 | 1.23 (1.13 to 1.33) | 0 |
| YLDs (Years Lived with Disability) | Angola | Male | 4 | 1990 | 2021 | 36.831145628867 | 1.25 (1.18 to 1.31) | 0 |
| YLDs (Years Lived with Disability) | Antigua and Barbuda | Both | 5 | 1990 | 2021 | -1.661678434 | -0.35 (-0.75 to 0.06) | 0.097 |
| YLDs (Years Lived with Disability) | Antigua and Barbuda | Female | 3 | 1990 | 2021 | -2.583718831 | -0.28 (-0.49 to -0.07) | 0.01 |
| YLDs (Years Lived with Disability) | Antigua and Barbuda | Male | 5 | 1990 | 2021 | -3.619565149 | -0.57 (-0.87 to -0.26) | 0 |
| YLDs (Years Lived with Disability) | Argentina | Both | 5 | 1990 | 2021 | 13.108660009208 | 1.35 (1.15 to 1.56) | 0 |
| YLDs (Years Lived with Disability) | Argentina | Female | 5 | 1990 | 2021 | 13.604115254431 | 1.42 (1.21 to 1.62) | 0 |
| YLDs (Years Lived with Disability) | Argentina | Male | 4 | 1990 | 2021 | 8.92470863 | 1.2 (0.94 to 1.47) | 0 |
| YLDs (Years Lived with Disability) | Armenia | Both | 4 | 1990 | 2021 | 39.64914865 | 2.73 (2.59 to 2.86) | 0 |
| YLDs (Years Lived with Disability) | Armenia | Female | 4 | 1990 | 2021 | 23.534706143614 | 2.49 (2.28 to 2.7) | 0 |
| YLDs (Years Lived with Disability) | Armenia | Male | 4 | 1990 | 2021 | 23.719862127009 | 3.17 (2.9 to 3.43) | 0 |
| YLDs (Years Lived with Disability) | Australasia | Both | 4 | 1990 | 2021 | 46.192428331184 | 2.53 (2.43 to 2.64) | 0 |
| YLDs (Years Lived with Disability) | Australasia | Female | 3 | 1990 | 2021 | 15.666634205736 | 2.56 (2.23 to 2.88) | 0 |
| YLDs (Years Lived with Disability) | Australasia | Male | 4 | 1990 | 2021 | 26.283395698678 | 2.47 (2.28 to 2.65) | 0 |
| YLDs (Years Lived with Disability) | Australia | Both | 4 | 1990 | 2021 | 43.631212868004 | 2.72 (2.6 to 2.85) | 0 |
| YLDs (Years Lived with Disability) | Australia | Female | 4 | 1990 | 2021 | 17.52222067 | 2.87 (2.54 to 3.19) | 0 |
| YLDs (Years Lived with Disability) | Australia | Male | 5 | 1990 | 2021 | 40.985335268913 | 2.55 (2.43 to 2.68) | 0 |
| YLDs (Years Lived with Disability) | Austria | Both | 5 | 1990 | 2021 | 19.671928444615 | 1.54 (1.38 to 1.69) | 0 |
| YLDs (Years Lived with Disability) | Austria | Female | 5 | 1990 | 2021 | 23.234747802737 | 1.32 (1.21 to 1.44) | 0 |
| YLDs (Years Lived with Disability) | Austria | Male | 5 | 1990 | 2021 | 21.942418459173 | 2.07 (1.88 to 2.25) | 0 |
| YLDs (Years Lived with Disability) | Azerbaijan | Both | 5 | 1990 | 2021 | 21.503050359888 | 0.83 (0.76 to 0.91) | 0 |
| YLDs (Years Lived with Disability) | Azerbaijan | Female | 5 | 1990 | 2021 | 11.577467309326 | 0.97 (0.8 to 1.13) | 0 |
| YLDs (Years Lived with Disability) | Azerbaijan | Male | 5 | 1990 | 2021 | 6.80754319 | 0.55 (0.39 to 0.71) | 0 |
| YLDs (Years Lived with Disability) | Bahamas | Both | 4 | 1990 | 2021 | 2.750573774 | 0.16 (0.05 to 0.28) | 0.006 |
| YLDs (Years Lived with Disability) | Bahamas | Female | 5 | 1990 | 2021 | -0.253439618 | -0.03 (-0.27 to 0.21) | 0.8 |
| YLDs (Years Lived with Disability) | Bahamas | Male | 4 | 1990 | 2021 | 4.461276243 | 0.29 (0.16 to 0.41) | 0 |
| YLDs (Years Lived with Disability) | Bahrain | Both | 4 | 1990 | 2021 | 45.506965731434 | 2.14 (2.04 to 2.23) | 0 |
| YLDs (Years Lived with Disability) | Bahrain | Female | 3 | 1990 | 2021 | 39.011025577246 | 2.12 (2.01 to 2.23) | 0 |
| YLDs (Years Lived with Disability) | Bahrain | Male | 4 | 1990 | 2021 | 33.47396281 | 2.17 (2.04 to 2.3) | 0 |
| YLDs (Years Lived with Disability) | Bangladesh | Both | 3 | 1990 | 2021 | 31.120336127797 | 1.87 (1.75 to 1.99) | 0 |
| YLDs (Years Lived with Disability) | Bangladesh | Female | 3 | 1990 | 2021 | 57.766998282254 | 1.61 (1.56 to 1.67) | 0 |
| YLDs (Years Lived with Disability) | Bangladesh | Male | 4 | 1990 | 2021 | 30.661413433003 | 2.17 (2.03 to 2.31) | 0 |
| YLDs (Years Lived with Disability) | Barbados | Both | 5 | 1990 | 2021 | 27.217899135716 | 0.95 (0.88 to 1.01) | 0 |
| YLDs (Years Lived with Disability) | Barbados | Female | 5 | 1990 | 2021 | 11.638732912965 | 0.74 (0.62 to 0.87) | 0 |
| YLDs (Years Lived with Disability) | Barbados | Male | 4 | 1990 | 2021 | 25.858022686011 | 1 (0.93 to 1.08) | 0 |
| YLDs (Years Lived with Disability) | Belarus | Both | 5 | 1990 | 2021 | 7.312828707 | 1.09 (0.8 to 1.39) | 0 |
| YLDs (Years Lived with Disability) | Belarus | Female | 5 | 1990 | 2021 | 7.937310703 | 1.03 (0.77 to 1.28) | 0 |
| YLDs (Years Lived with Disability) | Belarus | Male | 5 | 1990 | 2021 | 16.746078152897 | 1.41 (1.25 to 1.58) | 0 |
| YLDs (Years Lived with Disability) | Belgium | Both | 3 | 1990 | 2021 | 9.697090125 | 1.65 (1.32 to 1.99) | 0 |
| YLDs (Years Lived with Disability) | Belgium | Female | 3 | 1990 | 2021 | 15.120880040443 | 1.34 (1.16 to 1.51) | 0 |
| YLDs (Years Lived with Disability) | Belgium | Male | 4 | 1990 | 2021 | 20.812685439042 | 2.45 (2.22 to 2.69) | 0 |
| YLDs (Years Lived with Disability) | Belize | Both | 5 | 1990 | 2021 | 0.637613797 | 0.09 (-0.19 to 0.37) | 0.524 |
| YLDs (Years Lived with Disability) | Belize | Female | 5 | 1990 | 2021 | 3.469273504 | 0.33 (0.14 to 0.51) | 0.001 |
| YLDs (Years Lived with Disability) | Belize | Male | 5 | 1990 | 2021 | -2.049645785 | -0.24 (-0.46 to -0.01) | 0.04 |
| YLDs (Years Lived with Disability) | Benin | Both | 3 | 1990 | 2021 | 20.200123724889 | 0.94 (0.85 to 1.03) | 0 |
| YLDs (Years Lived with Disability) | Benin | Female | 4 | 1990 | 2021 | 18.308022497096 | 1.04 (0.93 to 1.16) | 0 |
| YLDs (Years Lived with Disability) | Benin | Male | 5 | 1990 | 2021 | 16.540311746153 | 0.88 (0.78 to 0.99) | 0 |
| YLDs (Years Lived with Disability) | Bermuda | Both | 5 | 1990 | 2021 | 29.541165654206 | 1.2 (1.12 to 1.29) | 0 |
| YLDs (Years Lived with Disability) | Bermuda | Female | 4 | 1990 | 2021 | 16.929620393042 | 1.16 (1.02 to 1.29) | 0 |
| YLDs (Years Lived with Disability) | Bermuda | Male | 5 | 1990 | 2021 | 27.819808281455 | 1.31 (1.22 to 1.41) | 0 |
| YLDs (Years Lived with Disability) | Bhutan | Both | 2 | 1990 | 2021 | 68.341086351515 | 2.03 (1.97 to 2.09) | 0 |
| YLDs (Years Lived with Disability) | Bhutan | Female | 3 | 1990 | 2021 | 44.93007712 | 2.15 (2.06 to 2.25) | 0 |
| YLDs (Years Lived with Disability) | Bhutan | Male | 3 | 1990 | 2021 | 23.971149458163 | 1.75 (1.61 to 1.9) | 0 |
| YLDs (Years Lived with Disability) | Bolivia (Plurinational State of) | Both | 4 | 1990 | 2021 | 30.468400119424 | 1.23 (1.15 to 1.31) | 0 |
| YLDs (Years Lived with Disability) | Bolivia (Plurinational State of) | Female | 4 | 1990 | 2021 | 24.414757580739 | 1.35 (1.24 to 1.46) | 0 |
| YLDs (Years Lived with Disability) | Bolivia (Plurinational State of) | Male | 1 | 1990 | 2021 | 52.089378745329 | 1.05 (1.01 to 1.09) | 0 |
| YLDs (Years Lived with Disability) | Bosnia and Herzegovina | Both | 5 | 1990 | 2021 | 11.947699476112 | 1.12 (0.93 to 1.3) | 0 |
| YLDs (Years Lived with Disability) | Bosnia and Herzegovina | Female | 5 | 1990 | 2021 | 12.598867969228 | 1.3 (1.1 to 1.5) | 0 |
| YLDs (Years Lived with Disability) | Bosnia and Herzegovina | Male | 3 | 1990 | 2021 | 12.383810098122 | 0.84 (0.71 to 0.97) | 0 |
| YLDs (Years Lived with Disability) | Botswana | Both | 5 | 1990 | 2021 | 17.571437149363 | 0.8 (0.71 to 0.89) | 0 |
| YLDs (Years Lived with Disability) | Botswana | Female | 4 | 1990 | 2021 | 22.372684021864 | 1.04 (0.95 to 1.13) | 0 |
| YLDs (Years Lived with Disability) | Botswana | Male | 5 | 1990 | 2021 | 5.449796526 | 0.34 (0.22 to 0.47) | 0 |
| YLDs (Years Lived with Disability) | Brazil | Both | 5 | 1990 | 2021 | 64.041638169448 | 2 (1.94 to 2.07) | 0 |
| YLDs (Years Lived with Disability) | Brazil | Female | 5 | 1990 | 2021 | 99.718800384011 | 2.15 (2.11 to 2.2) | 0 |
| YLDs (Years Lived with Disability) | Brazil | Male | 4 | 1990 | 2021 | 56.257720856179 | 1.8 (1.74 to 1.87) | 0 |
| YLDs (Years Lived with Disability) | Brunei Darussalam | Both | 5 | 1990 | 2021 | 23.588166319929 | 1.24 (1.14 to 1.35) | 0 |
| YLDs (Years Lived with Disability) | Brunei Darussalam | Female | 4 | 1990 | 2021 | 33.19108065 | 1.08 (1.02 to 1.15) | 0 |
| YLDs (Years Lived with Disability) | Brunei Darussalam | Male | 3 | 1990 | 2021 | 21.662215201798 | 1.43 (1.3 to 1.56) | 0 |
| YLDs (Years Lived with Disability) | Bulgaria | Both | 5 | 1990 | 2021 | 0.847690107 | 0.23 (-0.29 to 0.75) | 0.397 |
| YLDs (Years Lived with Disability) | Bulgaria | Female | 5 | 1990 | 2021 | -0.598362356 | -0.1 (-0.41 to 0.22) | 0.55 |
| YLDs (Years Lived with Disability) | Bulgaria | Male | 5 | 1990 | 2021 | 3.521455874 | 0.68 (0.3 to 1.06) | 0 |
| YLDs (Years Lived with Disability) | Burkina Faso | Both | 5 | 1990 | 2021 | 13.779648653758 | 0.87 (0.75 to 1) | 0 |
| YLDs (Years Lived with Disability) | Burkina Faso | Female | 3 | 1990 | 2021 | 19.184610191554 | 1.11 (0.99 to 1.22) | 0 |
| YLDs (Years Lived with Disability) | Burkina Faso | Male | 5 | 1990 | 2021 | 10.612489014416 | 0.61 (0.5 to 0.72) | 0 |
| YLDs (Years Lived with Disability) | Burundi | Both | 4 | 1990 | 2021 | 32.323350261491 | 1.34 (1.26 to 1.42) | 0 |
| YLDs (Years Lived with Disability) | Burundi | Female | 5 | 1990 | 2021 | 24.154397839336 | 1.76 (1.62 to 1.91) | 0 |
| YLDs (Years Lived with Disability) | Burundi | Male | 3 | 1990 | 2021 | 22.403771489645 | 0.91 (0.83 to 0.99) | 0 |
| YLDs (Years Lived with Disability) | Cabo Verde | Both | 4 | 1990 | 2021 | 11.684898180584 | 0.47 (0.39 to 0.55) | 0 |
| YLDs (Years Lived with Disability) | Cabo Verde | Female | 3 | 1990 | 2021 | 24.932295395265 | 0.69 (0.64 to 0.74) | 0 |
| YLDs (Years Lived with Disability) | Cabo Verde | Male | 4 | 1990 | 2021 | -0.581018343 | -0.06 (-0.27 to 0.15) | 0.561 |
| YLDs (Years Lived with Disability) | Cambodia | Both | 3 | 1990 | 2021 | 27.556881503431 | 1.04 (0.96 to 1.11) | 0 |
| YLDs (Years Lived with Disability) | Cambodia | Female | 3 | 1990 | 2021 | 17.672824151222 | 0.98 (0.87 to 1.09) | 0 |
| YLDs (Years Lived with Disability) | Cambodia | Male | 2 | 1990 | 2021 | 42.094370538135 | 1.12 (1.07 to 1.18) | 0 |
| YLDs (Years Lived with Disability) | Cameroon | Both | 4 | 1990 | 2021 | 23.321727169884 | 0.86 (0.79 to 0.93) | 0 |
| YLDs (Years Lived with Disability) | Cameroon | Female | 5 | 1990 | 2021 | 19.55675595 | 0.95 (0.86 to 1.05) | 0 |
| YLDs (Years Lived with Disability) | Cameroon | Male | 4 | 1990 | 2021 | 12.750191178941 | 0.68 (0.57 to 0.78) | 0 |
| YLDs (Years Lived with Disability) | Canada | Both | 4 | 1990 | 2021 | 12.672129933992 | 2.26 (1.91 to 2.62) | 0 |
| YLDs (Years Lived with Disability) | Canada | Female | 5 | 1990 | 2021 | 13.973551237977 | 2.21 (1.9 to 2.53) | 0 |
| YLDs (Years Lived with Disability) | Canada | Male | 4 | 1990 | 2021 | 19.673817288175 | 2.34 (2.1 to 2.57) | 0 |
| YLDs (Years Lived with Disability) | Caribbean | Both | 5 | 1990 | 2021 | 17.683021516546 | 0.82 (0.73 to 0.91) | 0 |
| YLDs (Years Lived with Disability) | Caribbean | Female | 5 | 1990 | 2021 | 23.78934887 | 0.97 (0.89 to 1.05) | 0 |
| YLDs (Years Lived with Disability) | Caribbean | Male | 5 | 1990 | 2021 | 8.314343681 | 0.72 (0.55 to 0.89) | 0 |
| YLDs (Years Lived with Disability) | Central African Republic | Both | 3 | 1990 | 2021 | 3.153512192 | 0.14 (0.05 to 0.23) | 0.002 |
| YLDs (Years Lived with Disability) | Central African Republic | Female | 3 | 1990 | 2021 | 1.100333266 | 0.04 (-0.03 to 0.1) | 0.271 |
| YLDs (Years Lived with Disability) | Central African Republic | Male | 3 | 1990 | 2021 | 5.47009402 | 0.24 (0.15 to 0.32) | 0 |
| YLDs (Years Lived with Disability) | Central Asia | Both | 5 | 1990 | 2021 | 6.709811501 | 0.61 (0.43 to 0.79) | 0 |
| YLDs (Years Lived with Disability) | Central Asia | Female | 5 | 1990 | 2021 | 3.579121166 | 0.5 (0.23 to 0.78) | 0 |
| YLDs (Years Lived with Disability) | Central Asia | Male | 5 | 1990 | 2021 | 13.376489444669 | 0.84 (0.72 to 0.96) | 0 |
| YLDs (Years Lived with Disability) | Central Europe | Both | 4 | 1990 | 2021 | 18.901409884482 | 1.41 (1.26 to 1.55) | 0 |
| YLDs (Years Lived with Disability) | Central Europe | Female | 5 | 1990 | 2021 | 20.349619196765 | 1.5 (1.35 to 1.64) | 0 |
| YLDs (Years Lived with Disability) | Central Europe | Male | 4 | 1990 | 2021 | 18.220332067541 | 1.39 (1.24 to 1.54) | 0 |
| YLDs (Years Lived with Disability) | Central Latin America | Both | 5 | 1990 | 2021 | 38.735248331606 | 0.92 (0.87 to 0.97) | 0 |
| YLDs (Years Lived with Disability) | Central Latin America | Female | 5 | 1990 | 2021 | 45.558609528243 | 1.16 (1.11 to 1.21) | 0 |
| YLDs (Years Lived with Disability) | Central Latin America | Male | 5 | 1990 | 2021 | 12.668922987975 | 0.74 (0.63 to 0.86) | 0 |
| YLDs (Years Lived with Disability) | Central Sub-Saharan Africa | Both | 5 | 1990 | 2021 | 16.056263771052 | 0.58 (0.51 to 0.65) | 0 |
| YLDs (Years Lived with Disability) | Central Sub-Saharan Africa | Female | 5 | 1990 | 2021 | 11.498237309045 | 0.43 (0.35 to 0.5) | 0 |
| YLDs (Years Lived with Disability) | Central Sub-Saharan Africa | Male | 3 | 1990 | 2021 | 26.431864547852 | 0.77 (0.72 to 0.83) | 0 |
| YLDs (Years Lived with Disability) | Chad | Both | 3 | 1990 | 2021 | 21.597404805836 | 0.61 (0.55 to 0.66) | 0 |
| YLDs (Years Lived with Disability) | Chad | Female | 1 | 1990 | 2021 | 28.797113284752 | 0.59 (0.55 to 0.63) | 0 |
| YLDs (Years Lived with Disability) | Chad | Male | 3 | 1990 | 2021 | 15.126235300036 | 0.62 (0.54 to 0.7) | 0 |
| YLDs (Years Lived with Disability) | Chile | Both | 5 | 1990 | 2021 | 19.567361391102 | 2.21 (1.98 to 2.43) | 0 |
| YLDs (Years Lived with Disability) | Chile | Female | 4 | 1990 | 2021 | 9.923942303 | 2.11 (1.69 to 2.54) | 0 |
| YLDs (Years Lived with Disability) | Chile | Male | 3 | 1990 | 2021 | 36.987409735015 | 2.37 (2.25 to 2.5) | 0 |
| YLDs (Years Lived with Disability) | China | Both | 4 | 1990 | 2021 | 27.948382571313 | 1.4 (1.3 to 1.5) | 0 |
| YLDs (Years Lived with Disability) | China | Female | 4 | 1990 | 2021 | 46.586956536135 | 1.42 (1.36 to 1.48) | 0 |
| YLDs (Years Lived with Disability) | China | Male | 5 | 1990 | 2021 | 42.229890316079 | 1.54 (1.47 to 1.61) | 0 |
| YLDs (Years Lived with Disability) | Colombia | Both | 5 | 1990 | 2021 | 40.916271770007 | 2.11 (2.01 to 2.22) | 0 |
| YLDs (Years Lived with Disability) | Colombia | Female | 5 | 1990 | 2021 | 43.964646781745 | 2.42 (2.31 to 2.53) | 0 |
| YLDs (Years Lived with Disability) | Colombia | Male | 4 | 1990 | 2021 | 39.554328234488 | 1.8 (1.71 to 1.89) | 0 |
| YLDs (Years Lived with Disability) | Comoros | Both | 3 | 1990 | 2021 | 23.530992157422 | 0.53 (0.49 to 0.58) | 0 |
| YLDs (Years Lived with Disability) | Comoros | Female | 5 | 1990 | 2021 | 10.689716435834 | 0.45 (0.37 to 0.54) | 0 |
| YLDs (Years Lived with Disability) | Comoros | Male | 4 | 1990 | 2021 | 17.56525777 | 0.54 (0.48 to 0.6) | 0 |
| YLDs (Years Lived with Disability) | Congo | Both | 3 | 1990 | 2021 | 29.06891109 | 0.7 (0.65 to 0.75) | 0 |
| YLDs (Years Lived with Disability) | Congo | Female | 3 | 1990 | 2021 | 21.056828836737 | 0.61 (0.55 to 0.66) | 0 |
| YLDs (Years Lived with Disability) | Congo | Male | 4 | 1990 | 2021 | 23.440490158164 | 0.88 (0.81 to 0.96) | 0 |
| YLDs (Years Lived with Disability) | Cook Islands | Both | 4 | 1990 | 2021 | 57.136645125984 | 1.43 (1.38 to 1.48) | 0 |
| YLDs (Years Lived with Disability) | Cook Islands | Female | 5 | 1990 | 2021 | 30.574869928195 | 1.74 (1.62 to 1.85) | 0 |
| YLDs (Years Lived with Disability) | Cook Islands | Male | 5 | 1990 | 2021 | 18.178624357289 | 0.98 (0.87 to 1.09) | 0 |
| YLDs (Years Lived with Disability) | Costa Rica | Both | 5 | 1990 | 2021 | 20.333804325509 | 1.52 (1.37 to 1.66) | 0 |
| YLDs (Years Lived with Disability) | Costa Rica | Female | 5 | 1990 | 2021 | 33.81144457 | 1.88 (1.77 to 1.99) | 0 |
| YLDs (Years Lived with Disability) | Costa Rica | Male | 4 | 1990 | 2021 | 14.672266284557 | 1.18 (1.02 to 1.33) | 0 |
| YLDs (Years Lived with Disability) | Coted'Ivoire | Both | 2 | 1990 | 2021 | 14.512299084391 | 0.59 (0.51 to 0.67) | 0 |
| YLDs (Years Lived with Disability) | Coted'Ivoire | Female | 3 | 1990 | 2021 | 9.198222316 | 0.54 (0.43 to 0.66) | 0 |
| YLDs (Years Lived with Disability) | Coted'Ivoire | Male | 3 | 1990 | 2021 | 9.0781274 | 0.62 (0.49 to 0.76) | 0 |
| YLDs (Years Lived with Disability) | Croatia | Both | 3 | 1990 | 2021 | 13.120110440507 | 1.98 (1.68 to 2.28) | 0 |
| YLDs (Years Lived with Disability) | Croatia | Female | 5 | 1990 | 2021 | 16.686465567504 | 2.18 (1.92 to 2.44) | 0 |
| YLDs (Years Lived with Disability) | Croatia | Male | 3 | 1990 | 2021 | 4.408935453 | 1.64 (0.91 to 2.38) | 0 |
| YLDs (Years Lived with Disability) | Cuba | Both | 5 | 1990 | 2021 | 23.345580940979 | 0.76 (0.7 to 0.83) | 0 |
| YLDs (Years Lived with Disability) | Cuba | Female | 3 | 1990 | 2021 | 27.468378392555 | 0.88 (0.82 to 0.95) | 0 |
| YLDs (Years Lived with Disability) | Cuba | Male | 4 | 1990 | 2021 | 17.196785571713 | 0.74 (0.65 to 0.82) | 0 |
| YLDs (Years Lived with Disability) | Cyprus | Both | 5 | 1990 | 2021 | 4.18851588 | 1.49 (0.79 to 2.2) | 0 |
| YLDs (Years Lived with Disability) | Cyprus | Female | 5 | 1990 | 2021 | 3.884615438 | 1.42 (0.7 to 2.14) | 0 |
| YLDs (Years Lived with Disability) | Cyprus | Male | 5 | 1990 | 2021 | 4.042069886 | 1.57 (0.8 to 2.34) | 0 |
| YLDs (Years Lived with Disability) | Czechia | Both | 3 | 1990 | 2021 | 9.46972945 | 2.64 (2.09 to 3.2) | 0 |
| YLDs (Years Lived with Disability) | Czechia | Female | 3 | 1990 | 2021 | 9.064620496 | 2.66 (2.08 to 3.25) | 0 |
| YLDs (Years Lived with Disability) | Czechia | Male | 2 | 1990 | 2021 | 12.997999458914 | 2.76 (2.34 to 3.18) | 0 |
| YLDs (Years Lived with Disability) | Democratic People's Republic of Korea | Both | 5 | 1990 | 2021 | 10.61659787 | 0.59 (0.48 to 0.7) | 0 |
| YLDs (Years Lived with Disability) | Democratic People's Republic of Korea | Female | 5 | 1990 | 2021 | 12.259985078481 | 0.55 (0.46 to 0.64) | 0 |
| YLDs (Years Lived with Disability) | Democratic People's Republic of Korea | Male | 4 | 1990 | 2021 | 7.435732036 | 0.59 (0.44 to 0.75) | 0 |
| YLDs (Years Lived with Disability) | Democratic Republic of the Congo | Both | 4 | 1990 | 2021 | 6.799433609 | 0.37 (0.26 to 0.47) | 0 |
| YLDs (Years Lived with Disability) | Democratic Republic of the Congo | Female | 5 | 1990 | 2021 | 3.908166383 | 0.21 (0.1 to 0.31) | 0 |
| YLDs (Years Lived with Disability) | Democratic Republic of the Congo | Male | 4 | 1990 | 2021 | 14.896318151324 | 0.64 (0.56 to 0.73) | 0 |
| YLDs (Years Lived with Disability) | Denmark | Both | 2 | 1990 | 2021 | 28.929062348675 | 3.85 (3.59 to 4.12) | 0 |
| YLDs (Years Lived with Disability) | Denmark | Female | 2 | 1990 | 2021 | 25.555435654623 | 3.46 (3.19 to 3.74) | 0 |
| YLDs (Years Lived with Disability) | Denmark | Male | 3 | 1990 | 2021 | 19.052940526058 | 4.32 (3.87 to 4.78) | 0 |
| YLDs (Years Lived with Disability) | Djibouti | Both | 4 | 1990 | 2021 | 4.008536076 | 0.21 (0.11 to 0.31) | 0 |
| YLDs (Years Lived with Disability) | Djibouti | Female | 4 | 1990 | 2021 | 4.916755761 | 0.31 (0.19 to 0.44) | 0 |
| YLDs (Years Lived with Disability) | Djibouti | Male | 3 | 1990 | 2021 | 1.620271797 | 0.09 (-0.02 to 0.2) | 0.105 |
| YLDs (Years Lived with Disability) | Dominica | Both | 5 | 1990 | 2021 | 21.943578876464 | 0.66 (0.6 to 0.71) | 0 |
| YLDs (Years Lived with Disability) | Dominica | Female | 3 | 1990 | 2021 | 21.588949825136 | 0.96 (0.87 to 1.04) | 0 |
| YLDs (Years Lived with Disability) | Dominica | Male | 4 | 1990 | 2021 | 7.100726519 | 0.38 (0.27 to 0.48) | 0 |
| YLDs (Years Lived with Disability) | Dominican Republic | Both | 3 | 1990 | 2021 | 3.039155586 | 0.27 (0.1 to 0.45) | 0.002 |
| YLDs (Years Lived with Disability) | Dominican Republic | Female | 2 | 1990 | 2021 | 16.676047433449 | 0.69 (0.61 to 0.77) | 0 |
| YLDs (Years Lived with Disability) | Dominican Republic | Male | 5 | 1990 | 2021 | -0.832897741 | -0.08 (-0.26 to 0.1) | 0.405 |
| YLDs (Years Lived with Disability) | East Asia | Both | 4 | 1990 | 2021 | 27.577266273792 | 1.42 (1.32 to 1.52) | 0 |
| YLDs (Years Lived with Disability) | East Asia | Female | 5 | 1990 | 2021 | 36.31240832 | 1.43 (1.35 to 1.51) | 0 |
| YLDs (Years Lived with Disability) | East Asia | Male | 5 | 1990 | 2021 | 52.612353436891 | 1.54 (1.48 to 1.6) | 0 |
| YLDs (Years Lived with Disability) | Eastern Europe | Both | 5 | 1990 | 2021 | 10.503496665551 | 1 (0.81 to 1.19) | 0 |
| YLDs (Years Lived with Disability) | Eastern Europe | Female | 4 | 1990 | 2021 | 8.645445438 | 0.93 (0.72 to 1.14) | 0 |
| YLDs (Years Lived with Disability) | Eastern Europe | Male | 5 | 1990 | 2021 | 18.395910101597 | 1.54 (1.38 to 1.71) | 0 |
| YLDs (Years Lived with Disability) | Eastern Sub-Saharan Africa | Both | 5 | 1990 | 2021 | 38.717560449198 | 0.76 (0.72 to 0.8) | 0 |
| YLDs (Years Lived with Disability) | Eastern Sub-Saharan Africa | Female | 5 | 1990 | 2021 | 27.302292957483 | 0.73 (0.67 to 0.78) | 0 |
| YLDs (Years Lived with Disability) | Eastern Sub-Saharan Africa | Male | 2 | 1990 | 2021 | 58.21695908 | 0.81 (0.78 to 0.84) | 0 |
| YLDs (Years Lived with Disability) | Ecuador | Both | 5 | 1990 | 2021 | 2.140033614 | 0.14 (0.01 to 0.27) | 0.032 |
| YLDs (Years Lived with Disability) | Ecuador | Female | 5 | 1990 | 2021 | 9.525887008 | 0.88 (0.7 to 1.06) | 0 |
| YLDs (Years Lived with Disability) | Ecuador | Male | 5 | 1990 | 2021 | -4.672936723 | -0.48 (-0.68 to -0.28) | 0 |
| YLDs (Years Lived with Disability) | Egypt | Both | 5 | 1990 | 2021 | 18.06787505 | 0.95 (0.85 to 1.06) | 0 |
| YLDs (Years Lived with Disability) | Egypt | Female | 4 | 1990 | 2021 | 30.292177992979 | 1.05 (0.98 to 1.12) | 0 |
| YLDs (Years Lived with Disability) | Egypt | Male | 4 | 1990 | 2021 | 7.209035329 | 0.55 (0.4 to 0.7) | 0 |
| YLDs (Years Lived with Disability) | El Salvador | Both | 4 | 1990 | 2021 | 14.274415313071 | 0.58 (0.5 to 0.65) | 0 |
| YLDs (Years Lived with Disability) | El Salvador | Female | 5 | 1990 | 2021 | 15.829724797513 | 0.84 (0.74 to 0.95) | 0 |
| YLDs (Years Lived with Disability) | El Salvador | Male | 5 | 1990 | 2021 | 2.913181927 | 0.22 (0.07 to 0.37) | 0.004 |
| YLDs (Years Lived with Disability) | Equatorial Guinea | Both | 4 | 1990 | 2021 | 34.739640862564 | 1.92 (1.81 to 2.03) | 0 |
| YLDs (Years Lived with Disability) | Equatorial Guinea | Female | 3 | 1990 | 2021 | 51.913988952747 | 2.03 (1.95 to 2.11) | 0 |
| YLDs (Years Lived with Disability) | Equatorial Guinea | Male | 5 | 1990 | 2021 | 20.708490308317 | 1.68 (1.52 to 1.84) | 0 |
| YLDs (Years Lived with Disability) | Eritrea | Both | 3 | 1990 | 2021 | 17.225044376948 | 0.68 (0.6 to 0.76) | 0 |
| YLDs (Years Lived with Disability) | Eritrea | Female | 3 | 1990 | 2021 | 11.442635206382 | 0.73 (0.6 to 0.86) | 0 |
| YLDs (Years Lived with Disability) | Eritrea | Male | 2 | 1990 | 2021 | 13.427108080194 | 0.64 (0.55 to 0.73) | 0 |
| YLDs (Years Lived with Disability) | Estonia | Both | 3 | 1990 | 2021 | 7.135965826 | 1.8 (1.3 to 2.3) | 0 |
| YLDs (Years Lived with Disability) | Estonia | Female | 3 | 1990 | 2021 | 7.503102962 | 1.63 (1.2 to 2.06) | 0 |
| YLDs (Years Lived with Disability) | Estonia | Male | 4 | 1990 | 2021 | 5.102293195 | 2.28 (1.4 to 3.17) | 0 |
| YLDs (Years Lived with Disability) | Eswatini | Both | 5 | 1990 | 2021 | 9.966366284 | 0.51 (0.41 to 0.61) | 0 |
| YLDs (Years Lived with Disability) | Eswatini | Female | 4 | 1990 | 2021 | 12.755965416929 | 0.69 (0.58 to 0.8) | 0 |
| YLDs (Years Lived with Disability) | Eswatini | Male | 5 | 1990 | 2021 | -3.134439698 | -0.17 (-0.28 to -0.06) | 0.002 |
| YLDs (Years Lived with Disability) | Ethiopia | Both | 5 | 1990 | 2021 | 40.929558301505 | 1.95 (1.85 to 2.04) | 0 |
| YLDs (Years Lived with Disability) | Ethiopia | Female | 5 | 1990 | 2021 | 28.513219097194 | 1.38 (1.28 to 1.47) | 0 |
| YLDs (Years Lived with Disability) | Ethiopia | Male | 5 | 1990 | 2021 | 36.278791774271 | 2.23 (2.11 to 2.35) | 0 |
| YLDs (Years Lived with Disability) | Fiji | Both | 4 | 1990 | 2021 | 18.770117564383 | 0.94 (0.84 to 1.04) | 0 |
| YLDs (Years Lived with Disability) | Fiji | Female | 4 | 1990 | 2021 | 14.722793900569 | 1.12 (0.97 to 1.27) | 0 |
| YLDs (Years Lived with Disability) | Fiji | Male | 5 | 1990 | 2021 | 8.738864933 | 0.62 (0.48 to 0.76) | 0 |
| YLDs (Years Lived with Disability) | Finland | Both | 5 | 1990 | 2021 | 6.767920627 | 0.64 (0.46 to 0.83) | 0 |
| YLDs (Years Lived with Disability) | Finland | Female | 5 | 1990 | 2021 | 3.007644923 | 0.36 (0.12 to 0.59) | 0.003 |
| YLDs (Years Lived with Disability) | Finland | Male | 3 | 1990 | 2021 | 6.476417875 | 1.95 (1.35 to 2.54) | 0 |
| YLDs (Years Lived with Disability) | France | Both | 5 | 1990 | 2021 | 17.127913990078 | 2.31 (2.04 to 2.58) | 0 |
| YLDs (Years Lived with Disability) | France | Female | 5 | 1990 | 2021 | 23.802921728386 | 2.31 (2.12 to 2.5) | 0 |
| YLDs (Years Lived with Disability) | France | Male | 5 | 1990 | 2021 | 17.130993394083 | 2.5 (2.21 to 2.79) | 0 |
| YLDs (Years Lived with Disability) | Gabon | Both | 5 | 1990 | 2021 | 11.627464924421 | 0.41 (0.34 to 0.48) | 0 |
| YLDs (Years Lived with Disability) | Gabon | Female | 5 | 1990 | 2021 | 10.456988951108 | 0.41 (0.33 to 0.48) | 0 |
| YLDs (Years Lived with Disability) | Gabon | Male | 4 | 1990 | 2021 | 5.80149551 | 0.35 (0.23 to 0.46) | 0 |
| YLDs (Years Lived with Disability) | Gambia | Both | 4 | 1990 | 2021 | 42.439259924958 | 0.7 (0.67 to 0.73) | 0 |
| YLDs (Years Lived with Disability) | Gambia | Female | 4 | 1990 | 2021 | 19.254299287587 | 0.77 (0.69 to 0.85) | 0 |
| YLDs (Years Lived with Disability) | Gambia | Male | 1 | 1990 | 2021 | 30.005841283053 | 0.57 (0.53 to 0.6) | 0 |
| YLDs (Years Lived with Disability) | Georgia | Both | 4 | 1990 | 2021 | 6.553642374 | 0.95 (0.66 to 1.23) | 0 |
| YLDs (Years Lived with Disability) | Georgia | Female | 4 | 1990 | 2021 | 3.075717007 | 0.72 (0.26 to 1.18) | 0.002 |
| YLDs (Years Lived with Disability) | Georgia | Male | 4 | 1990 | 2021 | 18.770290836497 | 1.47 (1.31 to 1.62) | 0 |
| YLDs (Years Lived with Disability) | Germany | Both | 4 | 1990 | 2021 | 23.639921848111 | 2.79 (2.56 to 3.03) | 0 |
| YLDs (Years Lived with Disability) | Germany | Female | 5 | 1990 | 2021 | 16.638207280403 | 2.71 (2.39 to 3.03) | 0 |
| YLDs (Years Lived with Disability) | Germany | Male | 4 | 1990 | 2021 | 17.703931986167 | 3.1 (2.76 to 3.45) | 0 |
| YLDs (Years Lived with Disability) | Ghana | Both | 5 | 1990 | 2021 | -1.061386369 | -0.09 (-0.25 to 0.07) | 0.289 |
| YLDs (Years Lived with Disability) | Ghana | Female | 4 | 1990 | 2021 | -1.63243441 | -0.14 (-0.3 to 0.03) | 0.103 |
| YLDs (Years Lived with Disability) | Ghana | Male | 4 | 1990 | 2021 | -2.632568308 | -0.24 (-0.42 to -0.06) | 0.008 |
| YLDs (Years Lived with Disability) | Global | Both | 2 | 1990 | 2021 | 64.731458392762 | 1.63 (1.58 to 1.68) | 0 |
| YLDs (Years Lived with Disability) | Global | Female | 4 | 1990 | 2021 | 52.573754218969 | 1.59 (1.53 to 1.65) | 0 |
| YLDs (Years Lived with Disability) | Global | Male | 3 | 1990 | 2021 | 42.703456773928 | 1.68 (1.6 to 1.76) | 0 |
| YLDs (Years Lived with Disability) | Greece | Both | 4 | 1990 | 2021 | -0.359755836 | -0.14 (-0.87 to 0.61) | 0.719 |
| YLDs (Years Lived with Disability) | Greece | Female | 4 | 1990 | 2021 | -0.369898205 | -0.17 (-1.09 to 0.75) | 0.711 |
| YLDs (Years Lived with Disability) | Greece | Male | 4 | 1990 | 2021 | -0.492736289 | -0.15 (-0.73 to 0.44) | 0.622 |
| YLDs (Years Lived with Disability) | Greenland | Both | 3 | 1990 | 2021 | 14.445637539676 | 1.22 (1.05 to 1.38) | 0 |
| YLDs (Years Lived with Disability) | Greenland | Female | 3 | 1990 | 2021 | 16.0462545 | 1.45 (1.27 to 1.63) | 0 |
| YLDs (Years Lived with Disability) | Greenland | Male | 4 | 1990 | 2021 | 9.58898064 | 0.84 (0.67 to 1.01) | 0 |
| YLDs (Years Lived with Disability) | Grenada | Both | 5 | 1990 | 2021 | 7.940774429 | 0.6 (0.45 to 0.74) | 0 |
| YLDs (Years Lived with Disability) | Grenada | Female | 5 | 1990 | 2021 | 8.325377897 | 0.72 (0.55 to 0.9) | 0 |
| YLDs (Years Lived with Disability) | Grenada | Male | 5 | 1990 | 2021 | 7.483745899 | 0.61 (0.45 to 0.77) | 0 |
| YLDs (Years Lived with Disability) | Guam | Both | 4 | 1990 | 2021 | 8.78833178 | 0.57 (0.44 to 0.7) | 0 |
| YLDs (Years Lived with Disability) | Guam | Female | 5 | 1990 | 2021 | 7.364668049 | 0.59 (0.43 to 0.75) | 0 |
| YLDs (Years Lived with Disability) | Guam | Male | 4 | 1990 | 2021 | 4.980738714 | 0.27 (0.17 to 0.38) | 0 |
| YLDs (Years Lived with Disability) | Guatemala | Both | 4 | 1990 | 2021 | 10.505820054674 | 0.82 (0.67 to 0.97) | 0 |
| YLDs (Years Lived with Disability) | Guatemala | Female | 4 | 1990 | 2021 | 12.833957237453 | 0.87 (0.74 to 1.01) | 0 |
| YLDs (Years Lived with Disability) | Guatemala | Male | 5 | 1990 | 2021 | 8.746363572 | 0.77 (0.6 to 0.95) | 0 |
| YLDs (Years Lived with Disability) | Guinea | Both | 3 | 1990 | 2021 | 21.643674892774 | 0.63 (0.58 to 0.69) | 0 |
| YLDs (Years Lived with Disability) | Guinea | Female | 4 | 1990 | 2021 | 15.592665304267 | 0.74 (0.65 to 0.84) | 0 |
| YLDs (Years Lived with Disability) | Guinea | Male | 5 | 1990 | 2021 | 16.174782727569 | 0.57 (0.5 to 0.63) | 0 |
| YLDs (Years Lived with Disability) | Guinea-Bissau | Both | 3 | 1990 | 2021 | 26.611877310166 | 0.73 (0.67 to 0.78) | 0 |
| YLDs (Years Lived with Disability) | Guinea-Bissau | Female | 3 | 1990 | 2021 | 12.767020045217 | 0.66 (0.56 to 0.76) | 0 |
| YLDs (Years Lived with Disability) | Guinea-Bissau | Male | 5 | 1990 | 2021 | 11.691835705528 | 0.73 (0.61 to 0.86) | 0 |
| YLDs (Years Lived with Disability) | Guyana | Both | 5 | 1990 | 2021 | 9.373879459 | 1.46 (1.15 to 1.77) | 0 |
| YLDs (Years Lived with Disability) | Guyana | Female | 5 | 1990 | 2021 | 7.190192211 | 1.16 (0.84 to 1.47) | 0 |
| YLDs (Years Lived with Disability) | Guyana | Male | 5 | 1990 | 2021 | 20.438038633056 | 1.72 (1.56 to 1.89) | 0 |
| YLDs (Years Lived with Disability) | Haiti | Both | 4 | 1990 | 2021 | 18.622048053035 | 0.79 (0.71 to 0.87) | 0 |
| YLDs (Years Lived with Disability) | Haiti | Female | 1 | 1990 | 2021 | 14.319869623515 | 0.57 (0.49 to 0.64) | 0 |
| YLDs (Years Lived with Disability) | Haiti | Male | 4 | 1990 | 2021 | 13.931395877583 | 0.67 (0.57 to 0.76) | 0 |
| YLDs (Years Lived with Disability) | High SDI | Both | 5 | 1990 | 2021 | 64.122230612569 | 2.11 (2.05 to 2.18) | 0 |
| YLDs (Years Lived with Disability) | High SDI | Female | 5 | 1990 | 2021 | 21.447856244597 | 2.04 (1.85 to 2.23) | 0 |
| YLDs (Years Lived with Disability) | High SDI | Male | 4 | 1990 | 2021 | 32.066471156169 | 2.24 (2.1 to 2.38) | 0 |
| YLDs (Years Lived with Disability) | High-income Asia Pacific | Both | 4 | 1990 | 2021 | 22.196043450563 | 2.29 (2.08 to 2.49) | 0 |
| YLDs (Years Lived with Disability) | High-income Asia Pacific | Female | 4 | 1990 | 2021 | 23.003544944705 | 2.51 (2.3 to 2.73) | 0 |
| YLDs (Years Lived with Disability) | High-income Asia Pacific | Male | 5 | 1990 | 2021 | 24.920477371858 | 1.95 (1.79 to 2.1) | 0 |
| YLDs (Years Lived with Disability) | High-income North America | Both | 5 | 1990 | 2021 | 33.644903051338 | 2.12 (1.99 to 2.24) | 0 |
| YLDs (Years Lived with Disability) | High-income North America | Female | 5 | 1990 | 2021 | 24.81735629 | 2.13 (1.96 to 2.3) | 0 |
| YLDs (Years Lived with Disability) | High-income North America | Male | 5 | 1990 | 2021 | 30.552010473588 | 2.06 (1.93 to 2.2) | 0 |
| YLDs (Years Lived with Disability) | High-middle SDI | Both | 5 | 1990 | 2021 | 34.477194057836 | 1.82 (1.72 to 1.92) | 0 |
| YLDs (Years Lived with Disability) | High-middle SDI | Female | 5 | 1990 | 2021 | 20.580041386723 | 1.83 (1.66 to 2.01) | 0 |
| YLDs (Years Lived with Disability) | High-middle SDI | Male | 5 | 1990 | 2021 | 48.55965919 | 1.88 (1.8 to 1.95) | 0 |
| YLDs (Years Lived with Disability) | Honduras | Both | 4 | 1990 | 2021 | 10.995444473919 | 0.62 (0.51 to 0.73) | 0 |
| YLDs (Years Lived with Disability) | Honduras | Female | 4 | 1990 | 2021 | 23.029474039447 | 0.98 (0.89 to 1.06) | 0 |
| YLDs (Years Lived with Disability) | Honduras | Male | 5 | 1990 | 2021 | 3.241859122 | 0.14 (0.06 to 0.23) | 0.001 |
| YLDs (Years Lived with Disability) | Hungary | Both | 5 | 1990 | 2021 | 15.344704387983 | 1.07 (0.94 to 1.21) | 0 |
| YLDs (Years Lived with Disability) | Hungary | Female | 3 | 1990 | 2021 | 10.945041466724 | 1.12 (0.92 to 1.33) | 0 |
| YLDs (Years Lived with Disability) | Hungary | Male | 4 | 1990 | 2021 | 9.368654948 | 1.02 (0.81 to 1.24) | 0 |
| YLDs (Years Lived with Disability) | Iceland | Both | 5 | 1990 | 2021 | 11.567341328309 | 2.63 (2.18 to 3.08) | 0 |
| YLDs (Years Lived with Disability) | Iceland | Female | 5 | 1990 | 2021 | 13.189688146827 | 2.45 (2.08 to 2.82) | 0 |
| YLDs (Years Lived with Disability) | Iceland | Male | 5 | 1990 | 2021 | 10.658338737706 | 2.85 (2.32 to 3.38) | 0 |
| YLDs (Years Lived with Disability) | India | Both | 3 | 1990 | 2021 | 27.70066145 | 1.32 (1.23 to 1.41) | 0 |
| YLDs (Years Lived with Disability) | India | Female | 4 | 1990 | 2021 | 23.75375093 | 1.44 (1.32 to 1.56) | 0 |
| YLDs (Years Lived with Disability) | India | Male | 4 | 1990 | 2021 | 44.058433867745 | 1.33 (1.27 to 1.39) | 0 |
| YLDs (Years Lived with Disability) | Indonesia | Both | 5 | 1990 | 2021 | 19.789788826119 | 0.92 (0.82 to 1.01) | 0 |
| YLDs (Years Lived with Disability) | Indonesia | Female | 5 | 1990 | 2021 | 29.742755318761 | 1.17 (1.09 to 1.25) | 0 |
| YLDs (Years Lived with Disability) | Indonesia | Male | 3 | 1990 | 2021 | 12.735021131845 | 0.48 (0.41 to 0.55) | 0 |
| YLDs (Years Lived with Disability) | Iran (Islamic Republic of) | Both | 4 | 1990 | 2021 | 73.554398052654 | 1.23 (1.2 to 1.27) | 0 |
| YLDs (Years Lived with Disability) | Iran (Islamic Republic of) | Female | 4 | 1990 | 2021 | 49.331463561179 | 1.2 (1.15 to 1.25) | 0 |
| YLDs (Years Lived with Disability) | Iran (Islamic Republic of) | Male | 4 | 1990 | 2021 | 55.603124420101 | 1.24 (1.19 to 1.28) | 0 |
| YLDs (Years Lived with Disability) | Iraq | Both | 4 | 1990 | 2021 | 18.597288126266 | 0.7 (0.63 to 0.77) | 0 |
| YLDs (Years Lived with Disability) | Iraq | Female | 4 | 1990 | 2021 | 16.250272330926 | 0.87 (0.77 to 0.98) | 0 |
| YLDs (Years Lived with Disability) | Iraq | Male | 5 | 1990 | 2021 | 5.423635623 | 0.35 (0.22 to 0.48) | 0 |
| YLDs (Years Lived with Disability) | Ireland | Both | 5 | 1990 | 2021 | 26.378831200119 | 3.03 (2.8 to 3.26) | 0 |
| YLDs (Years Lived with Disability) | Ireland | Female | 5 | 1990 | 2021 | 34.016231235608 | 2.98 (2.8 to 3.15) | 0 |
| YLDs (Years Lived with Disability) | Ireland | Male | 5 | 1990 | 2021 | 35.096437617255 | 3.11 (2.93 to 3.28) | 0 |
| YLDs (Years Lived with Disability) | Israel | Both | 4 | 1990 | 2021 | 27.365836886231 | 3.08 (2.85 to 3.3) | 0 |
| YLDs (Years Lived with Disability) | Israel | Female | 4 | 1990 | 2021 | 32.576099763051 | 3.13 (2.94 to 3.32) | 0 |
| YLDs (Years Lived with Disability) | Israel | Male | 5 | 1990 | 2021 | 36.990279267005 | 3.04 (2.88 to 3.21) | 0 |
| YLDs (Years Lived with Disability) | Italy | Both | 4 | 1990 | 2021 | 27.808489094248 | 2.97 (2.76 to 3.18) | 0 |
| YLDs (Years Lived with Disability) | Italy | Female | 3 | 1990 | 2021 | 18.182651352606 | 2.85 (2.54 to 3.16) | 0 |
| YLDs (Years Lived with Disability) | Italy | Male | 5 | 1990 | 2021 | 26.238267293639 | 3.58 (3.3 to 3.85) | 0 |
| YLDs (Years Lived with Disability) | Jamaica | Both | 4 | 1990 | 2021 | 7.642014284 | 0.86 (0.64 to 1.09) | 0 |
| YLDs (Years Lived with Disability) | Jamaica | Female | 5 | 1990 | 2021 | 11.700487280306 | 0.95 (0.79 to 1.11) | 0 |
| YLDs (Years Lived with Disability) | Jamaica | Male | 5 | 1990 | 2021 | 5.741236113 | 0.72 (0.47 to 0.97) | 0 |
| YLDs (Years Lived with Disability) | Japan | Both | 4 | 1990 | 2021 | 15.713156027714 | 1.69 (1.48 to 1.9) | 0 |
| YLDs (Years Lived with Disability) | Japan | Female | 5 | 1990 | 2021 | 19.966697438074 | 1.83 (1.65 to 2.01) | 0 |
| YLDs (Years Lived with Disability) | Japan | Male | 5 | 1990 | 2021 | 19.869694002467 | 1.42 (1.28 to 1.57) | 0 |
| YLDs (Years Lived with Disability) | Jordan | Both | 4 | 1990 | 2021 | 21.761044193334 | 0.85 (0.78 to 0.93) | 0 |
| YLDs (Years Lived with Disability) | Jordan | Female | 3 | 1990 | 2021 | 21.206307267038 | 0.96 (0.87 to 1.05) | 0 |
| YLDs (Years Lived with Disability) | Jordan | Male | 2 | 1990 | 2021 | 21.648866559873 | 0.79 (0.72 to 0.86) | 0 |
| YLDs (Years Lived with Disability) | Kazakhstan | Both | 2 | 1990 | 2021 | 9.101159175 | 0.53 (0.42 to 0.65) | 0 |
| YLDs (Years Lived with Disability) | Kazakhstan | Female | 3 | 1990 | 2021 | 8.519017854 | 0.59 (0.45 to 0.72) | 0 |
| YLDs (Years Lived with Disability) | Kazakhstan | Male | 5 | 1990 | 2021 | 3.707215729 | 0.61 (0.29 to 0.93) | 0 |
| YLDs (Years Lived with Disability) | Kenya | Both | 5 | 1990 | 2021 | 6.853691767 | 0.4 (0.28 to 0.51) | 0 |
| YLDs (Years Lived with Disability) | Kenya | Female | 5 | 1990 | 2021 | 16.317807303973 | 0.75 (0.66 to 0.84) | 0 |
| YLDs (Years Lived with Disability) | Kenya | Male | 5 | 1990 | 2021 | 8.324008877 | 0.38 (0.29 to 0.48) | 0 |
| YLDs (Years Lived with Disability) | Kiribati | Both | 3 | 1990 | 2021 | 17.452589109051 | 0.53 (0.47 to 0.59) | 0 |
| YLDs (Years Lived with Disability) | Kiribati | Female | 3 | 1990 | 2021 | 13.921605453733 | 0.57 (0.49 to 0.65) | 0 |
| YLDs (Years Lived with Disability) | Kiribati | Male | 3 | 1990 | 2021 | 5.677929624 | 0.22 (0.14 to 0.29) | 0 |
| YLDs (Years Lived with Disability) | Kuwait | Both | 3 | 1990 | 2021 | 33.106994840971 | 2.07 (1.94 to 2.19) | 0 |
| YLDs (Years Lived with Disability) | Kuwait | Female | 4 | 1990 | 2021 | 32.73477971 | 2.37 (2.22 to 2.51) | 0 |
| YLDs (Years Lived with Disability) | Kuwait | Male | 3 | 1990 | 2021 | 25.654265469062 | 1.83 (1.69 to 1.97) | 0 |
| YLDs (Years Lived with Disability) | Kyrgyzstan | Both | 5 | 1990 | 2021 | 4.233236595 | 0.28 (0.15 to 0.41) | 0 |
| YLDs (Years Lived with Disability) | Kyrgyzstan | Female | 4 | 1990 | 2021 | -0.321956585 | -0.02 (-0.16 to 0.11) | 0.747 |
| YLDs (Years Lived with Disability) | Kyrgyzstan | Male | 5 | 1990 | 2021 | 21.742845227151 | 1.4 (1.27 to 1.53) | 0 |
| YLDs (Years Lived with Disability) | Lao People's Democratic Republic | Both | 5 | 1990 | 2021 | 26.149068449544 | 1.2 (1.11 to 1.29) | 0 |
| YLDs (Years Lived with Disability) | Lao People's Democratic Republic | Female | 2 | 1990 | 2021 | 27.289743360622 | 1.18 (1.1 to 1.27) | 0 |
| YLDs (Years Lived with Disability) | Lao People's Democratic Republic | Male | 2 | 1990 | 2021 | 26.098684255472 | 1.25 (1.16 to 1.35) | 0 |
| YLDs (Years Lived with Disability) | Latvia | Both | 5 | 1990 | 2021 | 12.609412926235 | 1.38 (1.16 to 1.59) | 0 |
| YLDs (Years Lived with Disability) | Latvia | Female | 5 | 1990 | 2021 | 20.828875038836 | 1.23 (1.12 to 1.35) | 0 |
| YLDs (Years Lived with Disability) | Latvia | Male | 5 | 1990 | 2021 | 11.099557713838 | 1.98 (1.63 to 2.33) | 0 |
| YLDs (Years Lived with Disability) | Lebanon | Both | 4 | 1990 | 2021 | 22.000089638014 | 0.98 (0.89 to 1.07) | 0 |
| YLDs (Years Lived with Disability) | Lebanon | Female | 4 | 1990 | 2021 | 14.095507919176 | 0.9 (0.77 to 1.02) | 0 |
| YLDs (Years Lived with Disability) | Lebanon | Male | 5 | 1990 | 2021 | 18.975026634952 | 1.09 (0.97 to 1.2) | 0 |
| YLDs (Years Lived with Disability) | Lesotho | Both | 2 | 1990 | 2021 | 8.678383448 | 0.29 (0.22 to 0.35) | 0 |
| YLDs (Years Lived with Disability) | Lesotho | Female | 3 | 1990 | 2021 | 8.018402548 | 0.46 (0.35 to 0.58) | 0 |
| YLDs (Years Lived with Disability) | Lesotho | Male | 4 | 1990 | 2021 | -9.038514197 | -0.39 (-0.48 to -0.31) | 0 |
| YLDs (Years Lived with Disability) | Liberia | Both | 4 | 1990 | 2021 | 14.649599720603 | 0.68 (0.59 to 0.77) | 0 |
| YLDs (Years Lived with Disability) | Liberia | Female | 3 | 1990 | 2021 | 14.919080429149 | 0.63 (0.54 to 0.71) | 0 |
| YLDs (Years Lived with Disability) | Liberia | Male | 4 | 1990 | 2021 | 14.124183859726 | 0.68 (0.59 to 0.78) | 0 |
| YLDs (Years Lived with Disability) | Libya | Both | 5 | 1990 | 2021 | 16.16017293 | 0.59 (0.51 to 0.66) | 0 |
| YLDs (Years Lived with Disability) | Libya | Female | 4 | 1990 | 2021 | 16.556251261707 | 0.77 (0.68 to 0.86) | 0 |
| YLDs (Years Lived with Disability) | Libya | Male | 5 | 1990 | 2021 | 4.611030518 | 0.27 (0.15 to 0.38) | 0 |
| YLDs (Years Lived with Disability) | Lithuania | Both | 3 | 1990 | 2021 | 12.177036939142 | 2.06 (1.72 to 2.39) | 0 |
| YLDs (Years Lived with Disability) | Lithuania | Female | 4 | 1990 | 2021 | 12.65683543 | 2.02 (1.71 to 2.34) | 0 |
| YLDs (Years Lived with Disability) | Lithuania | Male | 5 | 1990 | 2021 | 9.547137352 | 1.98 (1.57 to 2.39) | 0 |
| YLDs (Years Lived with Disability) | Low SDI | Both | 5 | 1990 | 2021 | 22.339358043437 | 0.79 (0.72 to 0.86) | 0 |
| YLDs (Years Lived with Disability) | Low SDI | Female | 4 | 1990 | 2021 | 29.489893828466 | 0.67 (0.63 to 0.72) | 0 |
| YLDs (Years Lived with Disability) | Low SDI | Male | 4 | 1990 | 2021 | 27.068410602109 | 0.91 (0.84 to 0.98) | 0 |
| YLDs (Years Lived with Disability) | Low-middle SDI | Both | 3 | 1990 | 2021 | 39.881425267665 | 1.18 (1.13 to 1.24) | 0 |
| YLDs (Years Lived with Disability) | Low-middle SDI | Female | 5 | 1990 | 2021 | 24.354913579549 | 1.17 (1.08 to 1.27) | 0 |
| YLDs (Years Lived with Disability) | Low-middle SDI | Male | 3 | 1990 | 2021 | 33.900304636521 | 1.21 (1.14 to 1.28) | 0 |
| YLDs (Years Lived with Disability) | Luxembourg | Both | 3 | 1990 | 2021 | 38.293198406445 | 2.71 (2.56 to 2.85) | 0 |
| YLDs (Years Lived with Disability) | Luxembourg | Female | 3 | 1990 | 2021 | 32.14471665 | 2.43 (2.28 to 2.58) | 0 |
| YLDs (Years Lived with Disability) | Luxembourg | Male | 4 | 1990 | 2021 | 44.341788246331 | 3.42 (3.27 to 3.58) | 0 |
| YLDs (Years Lived with Disability) | Madagascar | Both | 5 | 1990 | 2021 | 13.740951046378 | 0.66 (0.57 to 0.75) | 0 |
| YLDs (Years Lived with Disability) | Madagascar | Female | 3 | 1990 | 2021 | 20.815113881182 | 0.71 (0.64 to 0.78) | 0 |
| YLDs (Years Lived with Disability) | Madagascar | Male | 4 | 1990 | 2021 | 8.296272163 | 0.71 (0.54 to 0.88) | 0 |
| YLDs (Years Lived with Disability) | Malawi | Both | 3 | 1990 | 2021 | 23.247452984415 | 0.85 (0.78 to 0.92) | 0 |
| YLDs (Years Lived with Disability) | Malawi | Female | 2 | 1990 | 2021 | 23.579133116293 | 0.94 (0.86 to 1.02) | 0 |
| YLDs (Years Lived with Disability) | Malawi | Male | 3 | 1990 | 2021 | 20.547164721031 | 1.03 (0.93 to 1.13) | 0 |
| YLDs (Years Lived with Disability) | Malaysia | Both | 5 | 1990 | 2021 | 52.091992171073 | 1.86 (1.79 to 1.93) | 0 |
| YLDs (Years Lived with Disability) | Malaysia | Female | 4 | 1990 | 2021 | 34.152812401346 | 2.06 (1.94 to 2.18) | 0 |
| YLDs (Years Lived with Disability) | Malaysia | Male | 5 | 1990 | 2021 | 25.665779087459 | 1.61 (1.48 to 1.73) | 0 |
| YLDs (Years Lived with Disability) | Maldives | Both | 3 | 1990 | 2021 | 44.669632514983 | 1.93 (1.84 to 2.01) | 0 |
| YLDs (Years Lived with Disability) | Maldives | Female | 4 | 1990 | 2021 | 40.499702001306 | 2.17 (2.06 to 2.27) | 0 |
| YLDs (Years Lived with Disability) | Maldives | Male | 3 | 1990 | 2021 | 34.564210067041 | 1.93 (1.82 to 2.04) | 0 |
| YLDs (Years Lived with Disability) | Mali | Both | 5 | 1990 | 2021 | 20.276039785067 | 0.67 (0.6 to 0.73) | 0 |
| YLDs (Years Lived with Disability) | Mali | Female | 2 | 1990 | 2021 | 22.300634845058 | 0.61 (0.56 to 0.67) | 0 |
| YLDs (Years Lived with Disability) | Mali | Male | 3 | 1990 | 2021 | 12.980794946248 | 0.72 (0.61 to 0.83) | 0 |
| YLDs (Years Lived with Disability) | Malta | Both | 5 | 1990 | 2021 | 11.346726067979 | 2.09 (1.72 to 2.45) | 0 |
| YLDs (Years Lived with Disability) | Malta | Female | 3 | 1990 | 2021 | 11.699010581013 | 2.14 (1.77 to 2.5) | 0 |
| YLDs (Years Lived with Disability) | Malta | Male | 5 | 1990 | 2021 | 6.61363338 | 2.58 (1.81 to 3.35) | 0 |
| YLDs (Years Lived with Disability) | Marshall Islands | Both | 3 | 1990 | 2021 | 25.05810273 | 0.69 (0.64 to 0.75) | 0 |
| YLDs (Years Lived with Disability) | Marshall Islands | Female | 3 | 1990 | 2021 | 13.525376501486 | 0.83 (0.71 to 0.95) | 0 |
| YLDs (Years Lived with Disability) | Marshall Islands | Male | 5 | 1990 | 2021 | 10.138346764783 | 0.62 (0.5 to 0.73) | 0 |
| YLDs (Years Lived with Disability) | Mauritania | Both | 3 | 1990 | 2021 | 30.402998260036 | 0.96 (0.9 to 1.03) | 0 |
| YLDs (Years Lived with Disability) | Mauritania | Female | 3 | 1990 | 2021 | 20.692623691869 | 0.86 (0.78 to 0.94) | 0 |
| YLDs (Years Lived with Disability) | Mauritania | Male | 4 | 1990 | 2021 | 22.771197058154 | 1.07 (0.98 to 1.16) | 0 |
| YLDs (Years Lived with Disability) | Mauritius | Both | 5 | 1990 | 2021 | 7.124165413 | 0.88 (0.64 to 1.12) | 0 |
| YLDs (Years Lived with Disability) | Mauritius | Female | 5 | 1990 | 2021 | 5.916991462 | 0.78 (0.52 to 1.05) | 0 |
| YLDs (Years Lived with Disability) | Mauritius | Male | 5 | 1990 | 2021 | 6.624257696 | 1.15 (0.81 to 1.49) | 0 |
| YLDs (Years Lived with Disability) | Mexico | Both | 5 | 1990 | 2021 | 7.537563915 | 0.63 (0.46 to 0.79) | 0 |
| YLDs (Years Lived with Disability) | Mexico | Female | 5 | 1990 | 2021 | 12.341925088274 | 0.91 (0.76 to 1.05) | 0 |
| YLDs (Years Lived with Disability) | Mexico | Male | 5 | 1990 | 2021 | 3.800028311 | 0.52 (0.25 to 0.79) | 0 |
| YLDs (Years Lived with Disability) | Micronesia (Federated States of) | Both | 3 | 1990 | 2021 | 18.112844651658 | 0.72 (0.65 to 0.8) | 0 |
| YLDs (Years Lived with Disability) | Micronesia (Federated States of) | Female | 3 | 1990 | 2021 | 26.754538833841 | 0.81 (0.75 to 0.87) | 0 |
| YLDs (Years Lived with Disability) | Micronesia (Federated States of) | Male | 2 | 1990 | 2021 | 12.446034293578 | 0.55 (0.47 to 0.64) | 0 |
| YLDs (Years Lived with Disability) | Middle SDI | Both | 4 | 1990 | 2021 | 56.012769169351 | 1.23 (1.19 to 1.28) | 0 |
| YLDs (Years Lived with Disability) | Middle SDI | Female | 4 | 1990 | 2021 | 63.660329692272 | 1.39 (1.34 to 1.43) | 0 |
| YLDs (Years Lived with Disability) | Middle SDI | Male | 4 | 1990 | 2021 | 51.719200683829 | 1.02 (0.98 to 1.06) | 0 |
| YLDs (Years Lived with Disability) | Monaco | Both | 5 | 1990 | 2021 | 25.956648386876 | 1.48 (1.37 to 1.59) | 0 |
| YLDs (Years Lived with Disability) | Monaco | Female | 4 | 1990 | 2021 | 18.905813786498 | 1.28 (1.15 to 1.41) | 0 |
| YLDs (Years Lived with Disability) | Monaco | Male | 5 | 1990 | 2021 | 25.811284954883 | 1.77 (1.63 to 1.9) | 0 |
| YLDs (Years Lived with Disability) | Mongolia | Both | 5 | 1990 | 2021 | 28.796945131051 | 1.14 (1.06 to 1.21) | 0 |
| YLDs (Years Lived with Disability) | Mongolia | Female | 5 | 1990 | 2021 | 38.00752189 | 1.15 (1.09 to 1.21) | 0 |
| YLDs (Years Lived with Disability) | Mongolia | Male | 5 | 1990 | 2021 | 28.074818513525 | 1.12 (1.04 to 1.2) | 0 |
| YLDs (Years Lived with Disability) | Montenegro | Both | 4 | 1990 | 2021 | 6.743327175 | 0.24 (0.17 to 0.32) | 0 |
| YLDs (Years Lived with Disability) | Montenegro | Female | 5 | 1990 | 2021 | 7.087997915 | 0.49 (0.35 to 0.62) | 0 |
| YLDs (Years Lived with Disability) | Montenegro | Male | 4 | 1990 | 2021 | -0.861032835 | -0.03 (-0.1 to 0.04) | 0.389 |
| YLDs (Years Lived with Disability) | Morocco | Both | 4 | 1990 | 2021 | 15.067419999088 | 0.5 (0.44 to 0.57) | 0 |
| YLDs (Years Lived with Disability) | Morocco | Female | 3 | 1990 | 2021 | 27.091808867483 | 0.58 (0.54 to 0.62) | 0 |
| YLDs (Years Lived with Disability) | Morocco | Male | 5 | 1990 | 2021 | 1.976155305 | 0.28 (0 to 0.55) | 0.048 |
| YLDs (Years Lived with Disability) | Mozambique | Both | 4 | 1990 | 2021 | 16.95840172 | 0.75 (0.66 to 0.84) | 0 |
| YLDs (Years Lived with Disability) | Mozambique | Female | 4 | 1990 | 2021 | 15.204408368723 | 0.78 (0.68 to 0.88) | 0 |
| YLDs (Years Lived with Disability) | Mozambique | Male | 4 | 1990 | 2021 | 11.984471161704 | 0.71 (0.59 to 0.83) | 0 |
| YLDs (Years Lived with Disability) | Myanmar | Both | 4 | 1990 | 2021 | 29.40925486 | 1.51 (1.41 to 1.61) | 0 |
| YLDs (Years Lived with Disability) | Myanmar | Female | 5 | 1990 | 2021 | 19.485786442215 | 1.55 (1.39 to 1.7) | 0 |
| YLDs (Years Lived with Disability) | Myanmar | Male | 3 | 1990 | 2021 | 29.91990699 | 1.45 (1.36 to 1.55) | 0 |
| YLDs (Years Lived with Disability) | Namibia | Both | 4 | 1990 | 2021 | 34.242576683589 | 1.03 (0.97 to 1.09) | 0 |
| YLDs (Years Lived with Disability) | Namibia | Female | 4 | 1990 | 2021 | 27.457550064355 | 1.21 (1.12 to 1.3) | 0 |
| YLDs (Years Lived with Disability) | Namibia | Male | 4 | 1990 | 2021 | 9.429641376 | 0.71 (0.56 to 0.86) | 0 |
| YLDs (Years Lived with Disability) | Nauru | Both | 4 | 1990 | 2021 | 8.592772988 | 0.53 (0.41 to 0.65) | 0 |
| YLDs (Years Lived with Disability) | Nauru | Female | 3 | 1990 | 2021 | 1.854591397 | 0.13 (-0.01 to 0.27) | 0.064 |
| YLDs (Years Lived with Disability) | Nauru | Male | 3 | 1990 | 2021 | 8.500118148 | 0.32 (0.25 to 0.39) | 0 |
| YLDs (Years Lived with Disability) | Nepal | Both | 3 | 1990 | 2021 | 26.829263827967 | 1.3 (1.2 to 1.39) | 0 |
| YLDs (Years Lived with Disability) | Nepal | Female | 3 | 1990 | 2021 | 20.962792684184 | 1.29 (1.17 to 1.41) | 0 |
| YLDs (Years Lived with Disability) | Nepal | Male | 2 | 1990 | 2021 | 40.563433396121 | 1.38 (1.31 to 1.45) | 0 |
| YLDs (Years Lived with Disability) | Netherlands | Both | 5 | 1990 | 2021 | 28.593627478098 | 1.99 (1.85 to 2.13) | 0 |
| YLDs (Years Lived with Disability) | Netherlands | Female | 4 | 1990 | 2021 | 12.616248400685 | 1.6 (1.35 to 1.85) | 0 |
| YLDs (Years Lived with Disability) | Netherlands | Male | 5 | 1990 | 2021 | 10.257644501821 | 3.16 (2.55 to 3.77) | 0 |
| YLDs (Years Lived with Disability) | New Zealand | Both | 2 | 1990 | 2021 | 16.772460349234 | 1.12 (0.99 to 1.26) | 0 |
| YLDs (Years Lived with Disability) | New Zealand | Female | 2 | 1990 | 2021 | 9.870245573 | 0.83 (0.66 to 0.99) | 0 |
| YLDs (Years Lived with Disability) | New Zealand | Male | 5 | 1990 | 2021 | 49.400108630139 | 1.9 (1.82 to 1.98) | 0 |
| YLDs (Years Lived with Disability) | Nicaragua | Both | 3 | 1990 | 2021 | 22.714059328976 | 0.63 (0.58 to 0.69) | 0 |
| YLDs (Years Lived with Disability) | Nicaragua | Female | 4 | 1990 | 2021 | 20.167822195113 | 0.66 (0.6 to 0.73) | 0 |
| YLDs (Years Lived with Disability) | Nicaragua | Male | 2 | 1990 | 2021 | 12.626356388593 | 0.59 (0.5 to 0.68) | 0 |
| YLDs (Years Lived with Disability) | Niger | Both | 5 | 1990 | 2021 | 11.357726655459 | 0.51 (0.42 to 0.6) | 0 |
| YLDs (Years Lived with Disability) | Niger | Female | 2 | 1990 | 2021 | 7.935119413 | 0.22 (0.16 to 0.27) | 0 |
| YLDs (Years Lived with Disability) | Niger | Male | 5 | 1990 | 2021 | 13.26893738 | 0.63 (0.53 to 0.72) | 0 |
| YLDs (Years Lived with Disability) | Nigeria | Both | 5 | 1990 | 2021 | 2.997341718 | 0.06 (0.02 to 0.1) | 0.003 |
| YLDs (Years Lived with Disability) | Nigeria | Female | 5 | 1990 | 2021 | 0.929576742 | 0.06 (-0.07 to 0.19) | 0.353 |
| YLDs (Years Lived with Disability) | Nigeria | Male | 5 | 1990 | 2021 | -0.0072378 | 0 (-0.03 to 0.03) | 0.994 |
| YLDs (Years Lived with Disability) | Niue | Both | 4 | 1990 | 2021 | 13.185481393832 | 0.52 (0.44 to 0.6) | 0 |
| YLDs (Years Lived with Disability) | Niue | Female | 4 | 1990 | 2021 | 14.814575024858 | 0.72 (0.63 to 0.82) | 0 |
| YLDs (Years Lived with Disability) | Niue | Male | 3 | 1990 | 2021 | 3.524824481 | 0.14 (0.06 to 0.22) | 0 |
| YLDs (Years Lived with Disability) | North Africa and Middle East | Both | 4 | 1990 | 2021 | 36.846610014327 | 1.38 (1.31 to 1.46) | 0 |
| YLDs (Years Lived with Disability) | North Africa and Middle East | Female | 2 | 1990 | 2021 | 64.117514970404 | 1.44 (1.4 to 1.48) | 0 |
| YLDs (Years Lived with Disability) | North Africa and Middle East | Male | 5 | 1990 | 2021 | 35.440685689978 | 1.32 (1.24 to 1.39) | 0 |
| YLDs (Years Lived with Disability) | North Macedonia | Both | 4 | 1990 | 2021 | 16.846492607542 | 0.99 (0.87 to 1.1) | 0 |
| YLDs (Years Lived with Disability) | North Macedonia | Female | 3 | 1990 | 2021 | 28.801872175405 | 1.07 (1 to 1.15) | 0 |
| YLDs (Years Lived with Disability) | North Macedonia | Male | 5 | 1990 | 2021 | 6.437792336 | 0.96 (0.67 to 1.26) | 0 |
| YLDs (Years Lived with Disability) | Northern Mariana Islands | Both | 5 | 1990 | 2021 | 9.704215501 | 0.53 (0.42 to 0.63) | 0 |
| YLDs (Years Lived with Disability) | Northern Mariana Islands | Female | 5 | 1990 | 2021 | 4.048976273 | 0.74 (0.38 to 1.1) | 0 |
| YLDs (Years Lived with Disability) | Northern Mariana Islands | Male | 2 | 1990 | 2021 | 7.89033025 | 0.25 (0.18 to 0.31) | 0 |
| YLDs (Years Lived with Disability) | Norway | Both | 3 | 1990 | 2021 | 14.43388104 | 2.22 (1.92 to 2.53) | 0 |
| YLDs (Years Lived with Disability) | Norway | Female | 4 | 1990 | 2021 | 22.909748055063 | 1.63 (1.49 to 1.77) | 0 |
| YLDs (Years Lived with Disability) | Norway | Male | 3 | 1990 | 2021 | 19.853026122533 | 3.25 (2.92 to 3.58) | 0 |
| YLDs (Years Lived with Disability) | Oceania | Both | 3 | 1990 | 2021 | 21.998825903682 | 0.55 (0.5 to 0.6) | 0 |
| YLDs (Years Lived with Disability) | Oceania | Female | 5 | 1990 | 2021 | 20.046954823966 | 0.6 (0.54 to 0.66) | 0 |
| YLDs (Years Lived with Disability) | Oceania | Male | 3 | 1990 | 2021 | 20.00376548 | 0.55 (0.49 to 0.6) | 0 |
| YLDs (Years Lived with Disability) | Oman | Both | 5 | 1990 | 2021 | 40.908070960987 | 1.51 (1.43 to 1.58) | 0 |
| YLDs (Years Lived with Disability) | Oman | Female | 5 | 1990 | 2021 | 27.07828141 | 1.75 (1.62 to 1.88) | 0 |
| YLDs (Years Lived with Disability) | Oman | Male | 5 | 1990 | 2021 | 14.315011077991 | 1.03 (0.89 to 1.18) | 0 |
| YLDs (Years Lived with Disability) | Pakistan | Both | 3 | 1990 | 2021 | 28.462785730545 | 0.68 (0.63 to 0.73) | 0 |
| YLDs (Years Lived with Disability) | Pakistan | Female | 3 | 1990 | 2021 | 24.320298070219 | 0.75 (0.69 to 0.81) | 0 |
| YLDs (Years Lived with Disability) | Pakistan | Male | 3 | 1990 | 2021 | 26.763097743299 | 0.82 (0.76 to 0.88) | 0 |
| YLDs (Years Lived with Disability) | Palau | Both | 4 | 1990 | 2021 | -0.244740179 | -0.02 (-0.18 to 0.14) | 0.807 |
| YLDs (Years Lived with Disability) | Palau | Female | 5 | 1990 | 2021 | 4.835042701 | 0.41 (0.24 to 0.57) | 0 |
| YLDs (Years Lived with Disability) | Palau | Male | 3 | 1990 | 2021 | -4.207892324 | -0.17 (-0.25 to -0.09) | 0 |
| YLDs (Years Lived with Disability) | Palestine | Both | 4 | 1990 | 2021 | 18.278674544897 | 1.26 (1.13 to 1.4) | 0 |
| YLDs (Years Lived with Disability) | Palestine | Female | 5 | 1990 | 2021 | 15.408984437799 | 1.24 (1.08 to 1.39) | 0 |
| YLDs (Years Lived with Disability) | Palestine | Male | 4 | 1990 | 2021 | 15.024257058261 | 1.32 (1.15 to 1.49) | 0 |
| YLDs (Years Lived with Disability) | Panama | Both | 5 | 1990 | 2021 | 25.534992304857 | 1.19 (1.1 to 1.28) | 0 |
| YLDs (Years Lived with Disability) | Panama | Female | 3 | 1990 | 2021 | 20.968547118845 | 1.35 (1.22 to 1.48) | 0 |
| YLDs (Years Lived with Disability) | Panama | Male | 5 | 1990 | 2021 | 22.025331652874 | 1.02 (0.92 to 1.11) | 0 |
| YLDs (Years Lived with Disability) | Papua New Guinea | Both | 5 | 1990 | 2021 | 10.613764918655 | 0.57 (0.46 to 0.67) | 0 |
| YLDs (Years Lived with Disability) | Papua New Guinea | Female | 3 | 1990 | 2021 | 11.650456074978 | 0.61 (0.51 to 0.71) | 0 |
| YLDs (Years Lived with Disability) | Papua New Guinea | Male | 3 | 1990 | 2021 | 11.383650527485 | 0.5 (0.42 to 0.59) | 0 |
| YLDs (Years Lived with Disability) | Paraguay | Both | 3 | 1990 | 2021 | 27.218152820631 | 1.12 (1.04 to 1.2) | 0 |
| YLDs (Years Lived with Disability) | Paraguay | Female | 5 | 1990 | 2021 | 21.756698879245 | 1.42 (1.3 to 1.55) | 0 |
| YLDs (Years Lived with Disability) | Paraguay | Male | 4 | 1990 | 2021 | 10.042288122468 | 0.79 (0.64 to 0.95) | 0 |
| YLDs (Years Lived with Disability) | Peru | Both | 5 | 1990 | 2021 | 24.023963875763 | 1.17 (1.08 to 1.27) | 0 |
| YLDs (Years Lived with Disability) | Peru | Female | 5 | 1990 | 2021 | 35.863472805191 | 1.24 (1.17 to 1.31) | 0 |
| YLDs (Years Lived with Disability) | Peru | Male | 5 | 1990 | 2021 | 10.777023896561 | 1.14 (0.94 to 1.35) | 0 |
| YLDs (Years Lived with Disability) | Philippines | Both | 5 | 1990 | 2021 | 17.915983197134 | 0.64 (0.57 to 0.71) | 0 |
| YLDs (Years Lived with Disability) | Philippines | Female | 5 | 1990 | 2021 | 16.814743744855 | 0.8 (0.7 to 0.89) | 0 |
| YLDs (Years Lived with Disability) | Philippines | Male | 5 | 1990 | 2021 | 24.564901903138 | 0.41 (0.37 to 0.44) | 0 |
| YLDs (Years Lived with Disability) | Poland | Both | 5 | 1990 | 2021 | 20.779435936498 | 1.31 (1.19 to 1.43) | 0 |
| YLDs (Years Lived with Disability) | Poland | Female | 4 | 1990 | 2021 | 13.488053080138 | 1.5 (1.28 to 1.72) | 0 |
| YLDs (Years Lived with Disability) | Poland | Male | 4 | 1990 | 2021 | 11.453364093507 | 1.09 (0.91 to 1.28) | 0 |
| YLDs (Years Lived with Disability) | Portugal | Both | 5 | 1990 | 2021 | 45.763728459427 | 1.97 (1.88 to 2.05) | 0 |
| YLDs (Years Lived with Disability) | Portugal | Female | 5 | 1990 | 2021 | 20.866727626346 | 1.65 (1.49 to 1.81) | 0 |
| YLDs (Years Lived with Disability) | Portugal | Male | 5 | 1990 | 2021 | 21.868309916928 | 2.26 (2.05 to 2.46) | 0 |
| YLDs (Years Lived with Disability) | Puerto Rico | Both | 5 | 1990 | 2021 | 34.460066786984 | 1.39 (1.31 to 1.47) | 0 |
| YLDs (Years Lived with Disability) | Puerto Rico | Female | 5 | 1990 | 2021 | 22.220189938681 | 1.41 (1.28 to 1.53) | 0 |
| YLDs (Years Lived with Disability) | Puerto Rico | Male | 4 | 1990 | 2021 | 24.509740568043 | 1.4 (1.29 to 1.51) | 0 |
| YLDs (Years Lived with Disability) | Qatar | Both | 5 | 1990 | 2021 | 17.16016482 | 1.72 (1.53 to 1.92) | 0 |
| YLDs (Years Lived with Disability) | Qatar | Female | 4 | 1990 | 2021 | 28.654038684108 | 1.42 (1.33 to 1.52) | 0 |
| YLDs (Years Lived with Disability) | Qatar | Male | 5 | 1990 | 2021 | 13.368114678067 | 1.82 (1.55 to 2.09) | 0 |
| YLDs (Years Lived with Disability) | Republic of Korea | Both | 3 | 1990 | 2021 | 68.746924981302 | 4.37 (4.25 to 4.5) | 0 |
| YLDs (Years Lived with Disability) | Republic of Korea | Female | 5 | 1990 | 2021 | 48.146677681515 | 4.26 (4.08 to 4.44) | 0 |
| YLDs (Years Lived with Disability) | Republic of Korea | Male | 5 | 1990 | 2021 | 37.647192433802 | 4.53 (4.29 to 4.77) | 0 |
| YLDs (Years Lived with Disability) | Republic of Moldova | Both | 5 | 1990 | 2021 | 7.85403148 | 1.84 (1.38 to 2.31) | 0 |
| YLDs (Years Lived with Disability) | Republic of Moldova | Female | 5 | 1990 | 2021 | 5.656748946 | 1.64 (1.07 to 2.22) | 0 |
| YLDs (Years Lived with Disability) | Republic of Moldova | Male | 5 | 1990 | 2021 | 7.891727705 | 2.22 (1.67 to 2.78) | 0 |
| YLDs (Years Lived with Disability) | Romania | Both | 5 | 1990 | 2021 | 25.823585035954 | 1.09 (1.01 to 1.17) | 0 |
| YLDs (Years Lived with Disability) | Romania | Female | 5 | 1990 | 2021 | 26.438818786988 | 1.34 (1.24 to 1.44) | 0 |
| YLDs (Years Lived with Disability) | Romania | Male | 5 | 1990 | 2021 | 19.109384098874 | 0.92 (0.83 to 1.02) | 0 |
| YLDs (Years Lived with Disability) | Russian Federation | Both | 5 | 1990 | 2021 | 13.000055510153 | 1.14 (0.97 to 1.31) | 0 |
| YLDs (Years Lived with Disability) | Russian Federation | Female | 4 | 1990 | 2021 | 10.957180374819 | 1.1 (0.9 to 1.3) | 0 |
| YLDs (Years Lived with Disability) | Russian Federation | Male | 4 | 1990 | 2021 | 20.464210769703 | 1.63 (1.47 to 1.78) | 0 |
| YLDs (Years Lived with Disability) | Rwanda | Both | 3 | 1990 | 2021 | 11.352520294051 | 0.69 (0.57 to 0.82) | 0 |
| YLDs (Years Lived with Disability) | Rwanda | Female | 5 | 1990 | 2021 | 8.148142262 | 0.47 (0.36 to 0.59) | 0 |
| YLDs (Years Lived with Disability) | Rwanda | Male | 3 | 1990 | 2021 | 17.598406092756 | 0.99 (0.88 to 1.1) | 0 |
| YLDs (Years Lived with Disability) | Saint Kitts and Nevis | Both | 3 | 1990 | 2021 | 6.950769588 | 0.66 (0.48 to 0.85) | 0 |
| YLDs (Years Lived with Disability) | Saint Kitts and Nevis | Female | 4 | 1990 | 2021 | 8.859400455 | 0.64 (0.5 to 0.78) | 0 |
| YLDs (Years Lived with Disability) | Saint Kitts and Nevis | Male | 5 | 1990 | 2021 | 4.595979798 | 0.59 (0.34 to 0.85) | 0 |
| YLDs (Years Lived with Disability) | Saint Lucia | Both | 4 | 1990 | 2021 | 5.999646953 | 0.6 (0.4 to 0.79) | 0 |
| YLDs (Years Lived with Disability) | Saint Lucia | Female | 4 | 1990 | 2021 | 5.978835313 | 0.65 (0.44 to 0.86) | 0 |
| YLDs (Years Lived with Disability) | Saint Lucia | Male | 4 | 1990 | 2021 | 5.36053008 | 0.38 (0.24 to 0.52) | 0 |
| YLDs (Years Lived with Disability) | Saint Vincent and the Grenadines | Both | 5 | 1990 | 2021 | 0.854031334 | 0.09 (-0.12 to 0.3) | 0.393 |
| YLDs (Years Lived with Disability) | Saint Vincent and the Grenadines | Female | 4 | 1990 | 2021 | 1.244874229 | 0.16 (-0.09 to 0.4) | 0.213 |
| YLDs (Years Lived with Disability) | Saint Vincent and the Grenadines | Male | 5 | 1990 | 2021 | -3.849246276 | -0.36 (-0.55 to -0.18) | 0 |
| YLDs (Years Lived with Disability) | Samoa | Both | 2 | 1990 | 2021 | 17.851657527222 | 0.46 (0.41 to 0.51) | 0 |
| YLDs (Years Lived with Disability) | Samoa | Female | 4 | 1990 | 2021 | 6.317803845 | 0.55 (0.38 to 0.73) | 0 |
| YLDs (Years Lived with Disability) | Samoa | Male | 3 | 1990 | 2021 | 7.151054243 | 0.35 (0.25 to 0.44) | 0 |
| YLDs (Years Lived with Disability) | San Marino | Both | 5 | 1990 | 2021 | 20.144806348663 | 0.96 (0.87 to 1.06) | 0 |
| YLDs (Years Lived with Disability) | San Marino | Female | 4 | 1990 | 2021 | 10.384357517897 | 0.61 (0.49 to 0.72) | 0 |
| YLDs (Years Lived with Disability) | San Marino | Male | 5 | 1990 | 2021 | 27.301527205341 | 1.4 (1.3 to 1.5) | 0 |
| YLDs (Years Lived with Disability) | Sao Tome and Principe | Both | 4 | 1990 | 2021 | 28.194878057318 | 1.07 (0.99 to 1.14) | 0 |
| YLDs (Years Lived with Disability) | Sao Tome and Principe | Female | 5 | 1990 | 2021 | 24.266834394208 | 1.07 (0.98 to 1.16) | 0 |
| YLDs (Years Lived with Disability) | Sao Tome and Principe | Male | 4 | 1990 | 2021 | 18.979755226073 | 1.06 (0.95 to 1.17) | 0 |
| YLDs (Years Lived with Disability) | Saudi Arabia | Both | 3 | 1990 | 2021 | 46.81244048 | 1.08 (1.03 to 1.12) | 0 |
| YLDs (Years Lived with Disability) | Saudi Arabia | Female | 4 | 1990 | 2021 | 43.345499483539 | 1.43 (1.36 to 1.49) | 0 |
| YLDs (Years Lived with Disability) | Saudi Arabia | Male | 4 | 1990 | 2021 | 20.871675685914 | 0.79 (0.71 to 0.86) | 0 |
| YLDs (Years Lived with Disability) | Senegal | Both | 5 | 1990 | 2021 | 17.407664972497 | 0.83 (0.74 to 0.92) | 0 |
| YLDs (Years Lived with Disability) | Senegal | Female | 5 | 1990 | 2021 | 12.025533846511 | 0.77 (0.64 to 0.9) | 0 |
| YLDs (Years Lived with Disability) | Senegal | Male | 4 | 1990 | 2021 | 14.350274846644 | 0.91 (0.78 to 1.03) | 0 |
| YLDs (Years Lived with Disability) | Serbia | Both | 3 | 1990 | 2021 | 2.861604806 | 1.05 (0.33 to 1.78) | 0.004 |
| YLDs (Years Lived with Disability) | Serbia | Female | 3 | 1990 | 2021 | 2.913784256 | 0.97 (0.32 to 1.62) | 0.004 |
| YLDs (Years Lived with Disability) | Serbia | Male | 3 | 1990 | 2021 | 2.617523717 | 1.13 (0.28 to 1.98) | 0.009 |
| YLDs (Years Lived with Disability) | Seychelles | Both | 5 | 1990 | 2021 | 16.241462965346 | 1.08 (0.95 to 1.21) | 0 |
| YLDs (Years Lived with Disability) | Seychelles | Female | 4 | 1990 | 2021 | 21.516264147093 | 1.17 (1.06 to 1.27) | 0 |
| YLDs (Years Lived with Disability) | Seychelles | Male | 5 | 1990 | 2021 | 9.332952222 | 0.86 (0.68 to 1.04) | 0 |
| YLDs (Years Lived with Disability) | Sierra Leone | Both | 4 | 1990 | 2021 | 21.220273556295 | 0.89 (0.81 to 0.97) | 0 |
| YLDs (Years Lived with Disability) | Sierra Leone | Female | 4 | 1990 | 2021 | 12.855146129195 | 0.75 (0.63 to 0.86) | 0 |
| YLDs (Years Lived with Disability) | Sierra Leone | Male | 4 | 1990 | 2021 | 34.816579712038 | 1.01 (0.95 to 1.07) | 0 |
| YLDs (Years Lived with Disability) | Singapore | Both | 3 | 1990 | 2021 | 22.363053976912 | 2.74 (2.5 to 2.98) | 0 |
| YLDs (Years Lived with Disability) | Singapore | Female | 3 | 1990 | 2021 | 26.581631224557 | 2.88 (2.66 to 3.09) | 0 |
| YLDs (Years Lived with Disability) | Singapore | Male | 2 | 1990 | 2021 | 17.954615073227 | 2.57 (2.28 to 2.85) | 0 |
| YLDs (Years Lived with Disability) | Slovakia | Both | 3 | 1990 | 2021 | 7.216488041 | 1.53 (1.11 to 1.95) | 0 |
| YLDs (Years Lived with Disability) | Slovakia | Female | 3 | 1990 | 2021 | 6.535422133 | 1.41 (0.99 to 1.84) | 0 |
| YLDs (Years Lived with Disability) | Slovakia | Male | 2 | 1990 | 2021 | 11.95840296 | 1.93 (1.61 to 2.25) | 0 |
| YLDs (Years Lived with Disability) | Slovenia | Both | 5 | 1990 | 2021 | 31.526513188172 | 2.84 (2.66 to 3.02) | 0 |
| YLDs (Years Lived with Disability) | Slovenia | Female | 5 | 1990 | 2021 | 30.680478664368 | 2.63 (2.46 to 2.8) | 0 |
| YLDs (Years Lived with Disability) | Slovenia | Male | 5 | 1990 | 2021 | 31.845758643517 | 3.36 (3.15 to 3.57) | 0 |
| YLDs (Years Lived with Disability) | Solomon Islands | Both | 4 | 1990 | 2021 | 36.125907958508 | 0.73 (0.69 to 0.77) | 0 |
| YLDs (Years Lived with Disability) | Solomon Islands | Female | 2 | 1990 | 2021 | 13.872417096269 | 0.67 (0.57 to 0.76) | 0 |
| YLDs (Years Lived with Disability) | Solomon Islands | Male | 2 | 1990 | 2021 | 23.851235000889 | 0.84 (0.77 to 0.91) | 0 |
| YLDs (Years Lived with Disability) | Somalia | Both | 2 | 1990 | 2021 | -4.94452597 | -0.15 (-0.21 to -0.09) | 0 |
| YLDs (Years Lived with Disability) | Somalia | Female | 2 | 1990 | 2021 | -2.980184032 | -0.13 (-0.21 to -0.04) | 0.003 |
| YLDs (Years Lived with Disability) | Somalia | Male | 3 | 1990 | 2021 | -1.465238687 | -0.06 (-0.14 to 0.02) | 0.143 |
| YLDs (Years Lived with Disability) | South Africa | Both | 5 | 1990 | 2021 | 8.627416524 | 0.36 (0.28 to 0.44) | 0 |
| YLDs (Years Lived with Disability) | South Africa | Female | 5 | 1990 | 2021 | 10.708558717494 | 0.47 (0.39 to 0.56) | 0 |
| YLDs (Years Lived with Disability) | South Africa | Male | 4 | 1990 | 2021 | 3.211159115 | 0.15 (0.06 to 0.25) | 0.001 |
| YLDs (Years Lived with Disability) | South Asia | Both | 2 | 1990 | 2021 | 52.376427737143 | 1.34 (1.29 to 1.39) | 0 |
| YLDs (Years Lived with Disability) | South Asia | Female | 5 | 1990 | 2021 | 24.022508469352 | 1.46 (1.34 to 1.58) | 0 |
| YLDs (Years Lived with Disability) | South Asia | Male | 1 | 1990 | 2021 | 61.755259332774 | 1.36 (1.31 to 1.4) | 0 |
| YLDs (Years Lived with Disability) | South Sudan | Both | 2 | 1990 | 2021 | -1.951820406 | -0.06 (-0.12 to 0) | 0.051 |
| YLDs (Years Lived with Disability) | South Sudan | Female | 3 | 1990 | 2021 | -2.894235102 | -0.14 (-0.23 to -0.05) | 0.004 |
| YLDs (Years Lived with Disability) | South Sudan | Male | 3 | 1990 | 2021 | -1.585261003 | -0.07 (-0.16 to 0.02) | 0.113 |
| YLDs (Years Lived with Disability) | Southeast Asia | Both | 3 | 1990 | 2021 | 84.474315187084 | 1.66 (1.62 to 1.7) | 0 |
| YLDs (Years Lived with Disability) | Southeast Asia | Female | 3 | 1990 | 2021 | 64.084378904878 | 1.7 (1.65 to 1.76) | 0 |
| YLDs (Years Lived with Disability) | Southeast Asia | Male | 3 | 1990 | 2021 | 52.453596628095 | 1.62 (1.56 to 1.68) | 0 |
| YLDs (Years Lived with Disability) | Southern Latin America | Both | 5 | 1990 | 2021 | 29.010931901933 | 1.78 (1.66 to 1.9) | 0 |
| YLDs (Years Lived with Disability) | Southern Latin America | Female | 5 | 1990 | 2021 | 23.052291546298 | 1.69 (1.54 to 1.83) | 0 |
| YLDs (Years Lived with Disability) | Southern Latin America | Male | 5 | 1990 | 2021 | 22.726721633371 | 1.92 (1.75 to 2.09) | 0 |
| YLDs (Years Lived with Disability) | Southern Sub-Saharan Africa | Both | 4 | 1990 | 2021 | 8.47790427 | 0.4 (0.3 to 0.49) | 0 |
| YLDs (Years Lived with Disability) | Southern Sub-Saharan Africa | Female | 5 | 1990 | 2021 | 10.08414764 | 0.51 (0.41 to 0.61) | 0 |
| YLDs (Years Lived with Disability) | Southern Sub-Saharan Africa | Male | 4 | 1990 | 2021 | 3.423813307 | 0.17 (0.07 to 0.27) | 0.001 |
| YLDs (Years Lived with Disability) | Spain | Both | 4 | 1990 | 2021 | 17.897374862085 | 1.99 (1.77 to 2.21) | 0 |
| YLDs (Years Lived with Disability) | Spain | Female | 5 | 1990 | 2021 | 15.529601560996 | 2.18 (1.9 to 2.46) | 0 |
| YLDs (Years Lived with Disability) | Spain | Male | 5 | 1990 | 2021 | 11.589694728874 | 1.84 (1.53 to 2.16) | 0 |
| YLDs (Years Lived with Disability) | Sri Lanka | Both | 5 | 1990 | 2021 | 43.765580448381 | 2.24 (2.14 to 2.35) | 0 |
| YLDs (Years Lived with Disability) | Sri Lanka | Female | 5 | 1990 | 2021 | 32.623953133127 | 2.17 (2.04 to 2.31) | 0 |
| YLDs (Years Lived with Disability) | Sri Lanka | Male | 4 | 1990 | 2021 | 38.79203901 | 2.11 (2 to 2.22) | 0 |
| YLDs (Years Lived with Disability) | Sudan | Both | 5 | 1990 | 2021 | 17.86951867 | 0.95 (0.85 to 1.06) | 0 |
| YLDs (Years Lived with Disability) | Sudan | Female | 5 | 1990 | 2021 | 16.669189256247 | 1.31 (1.15 to 1.46) | 0 |
| YLDs (Years Lived with Disability) | Sudan | Male | 5 | 1990 | 2021 | 5.04312137 | 0.6 (0.36 to 0.83) | 0 |
| YLDs (Years Lived with Disability) | Suriname | Both | 5 | 1990 | 2021 | 13.268903739048 | 0.77 (0.66 to 0.89) | 0 |
| YLDs (Years Lived with Disability) | Suriname | Female | 5 | 1990 | 2021 | 14.810483870617 | 0.92 (0.8 to 1.05) | 0 |
| YLDs (Years Lived with Disability) | Suriname | Male | 5 | 1990 | 2021 | 12.428747591516 | 0.69 (0.58 to 0.79) | 0 |
| YLDs (Years Lived with Disability) | Sweden | Both | 5 | 1990 | 2021 | 33.522474274807 | 3.63 (3.42 to 3.85) | 0 |
| YLDs (Years Lived with Disability) | Sweden | Female | 5 | 1990 | 2021 | 28.146851718368 | 3.26 (3.03 to 3.49) | 0 |
| YLDs (Years Lived with Disability) | Sweden | Male | 4 | 1990 | 2021 | 54.772642316948 | 4.26 (4.1 to 4.42) | 0 |
| YLDs (Years Lived with Disability) | Switzerland | Both | 5 | 1990 | 2021 | 25.045917183317 | 3.07 (2.82 to 3.31) | 0 |
| YLDs (Years Lived with Disability) | Switzerland | Female | 5 | 1990 | 2021 | 20.122971363847 | 2.7 (2.44 to 2.97) | 0 |
| YLDs (Years Lived with Disability) | Switzerland | Male | 3 | 1990 | 2021 | 28.605061827601 | 3.73 (3.47 to 3.99) | 0 |
| YLDs (Years Lived with Disability) | Syrian Arab Republic | Both | 4 | 1990 | 2021 | 11.836203685605 | 0.67 (0.56 to 0.79) | 0 |
| YLDs (Years Lived with Disability) | Syrian Arab Republic | Female | 5 | 1990 | 2021 | 18.674333204746 | 0.83 (0.74 to 0.91) | 0 |
| YLDs (Years Lived with Disability) | Syrian Arab Republic | Male | 5 | 1990 | 2021 | 6.319022024 | 0.47 (0.33 to 0.62) | 0 |
| YLDs (Years Lived with Disability) | Taiwan (Province of China) | Both | 5 | 1990 | 2021 | 13.06084522 | 1.87 (1.59 to 2.15) | 0 |
| YLDs (Years Lived with Disability) | Taiwan (Province of China) | Female | 5 | 1990 | 2021 | 24.829166681701 | 1.93 (1.78 to 2.09) | 0 |
| YLDs (Years Lived with Disability) | Taiwan (Province of China) | Male | 5 | 1990 | 2021 | 13.464643408297 | 1.69 (1.44 to 1.93) | 0 |
| YLDs (Years Lived with Disability) | Tajikistan | Both | 5 | 1990 | 2021 | -0.427310067 | -0.06 (-0.33 to 0.21) | 0.669 |
| YLDs (Years Lived with Disability) | Tajikistan | Female | 5 | 1990 | 2021 | -1.793905603 | -0.09 (-0.18 to 0.01) | 0.073 |
| YLDs (Years Lived with Disability) | Tajikistan | Male | 5 | 1990 | 2021 | 0.43768922 | 0.09 (-0.33 to 0.52) | 0.662 |
| YLDs (Years Lived with Disability) | Thailand | Both | 5 | 1990 | 2021 | 25.868230265316 | 1.46 (1.35 to 1.58) | 0 |
| YLDs (Years Lived with Disability) | Thailand | Female | 3 | 1990 | 2021 | 39.811393415669 | 1.44 (1.37 to 1.52) | 0 |
| YLDs (Years Lived with Disability) | Thailand | Male | 3 | 1990 | 2021 | 28.988545967653 | 1.46 (1.36 to 1.56) | 0 |
| YLDs (Years Lived with Disability) | Timor-Leste | Both | 4 | 1990 | 2021 | 10.875576479946 | 0.54 (0.45 to 0.64) | 0 |
| YLDs (Years Lived with Disability) | Timor-Leste | Female | 3 | 1990 | 2021 | 23.57785116 | 0.76 (0.69 to 0.82) | 0 |
| YLDs (Years Lived with Disability) | Timor-Leste | Male | 1 | 1990 | 2021 | 4.738813079 | 0.2 (0.12 to 0.29) | 0 |
| YLDs (Years Lived with Disability) | Togo | Both | 4 | 1990 | 2021 | 12.560162963152 | 0.59 (0.5 to 0.68) | 0 |
| YLDs (Years Lived with Disability) | Togo | Female | 2 | 1990 | 2021 | 26.812721796141 | 0.6 (0.56 to 0.65) | 0 |
| YLDs (Years Lived with Disability) | Togo | Male | 4 | 1990 | 2021 | 10.459858080669 | 0.58 (0.47 to 0.69) | 0 |
| YLDs (Years Lived with Disability) | Tokelau | Both | 2 | 1990 | 2021 | 31.050581338767 | 0.74 (0.69 to 0.78) | 0 |
| YLDs (Years Lived with Disability) | Tokelau | Female | 4 | 1990 | 2021 | 14.579822027534 | 0.95 (0.82 to 1.08) | 0 |
| YLDs (Years Lived with Disability) | Tokelau | Male | 1 | 1990 | 2021 | 22.551581491951 | 0.55 (0.5 to 0.6) | 0 |
| YLDs (Years Lived with Disability) | Tonga | Both | 3 | 1990 | 2021 | 15.896383145957 | 0.71 (0.62 to 0.8) | 0 |
| YLDs (Years Lived with Disability) | Tonga | Female | 3 | 1990 | 2021 | 11.470643595985 | 0.67 (0.55 to 0.78) | 0 |
| YLDs (Years Lived with Disability) | Tonga | Male | 5 | 1990 | 2021 | 13.763344216844 | 0.79 (0.68 to 0.91) | 0 |
| YLDs (Years Lived with Disability) | Trinidad and Tobago | Both | 3 | 1990 | 2021 | 28.507743188992 | 1.62 (1.51 to 1.73) | 0 |
| YLDs (Years Lived with Disability) | Trinidad and Tobago | Female | 3 | 1990 | 2021 | 19.884998516093 | 1.47 (1.32 to 1.61) | 0 |
| YLDs (Years Lived with Disability) | Trinidad and Tobago | Male | 5 | 1990 | 2021 | 33.629538752796 | 1.87 (1.76 to 1.98) | 0 |
| YLDs (Years Lived with Disability) | Tropical Latin America | Both | 5 | 1990 | 2021 | 63.666576028711 | 1.98 (1.92 to 2.04) | 0 |
| YLDs (Years Lived with Disability) | Tropical Latin America | Female | 5 | 1990 | 2021 | 95.097974713831 | 2.13 (2.09 to 2.18) | 0 |
| YLDs (Years Lived with Disability) | Tropical Latin America | Male | 4 | 1990 | 2021 | 56.124332428972 | 1.77 (1.71 to 1.84) | 0 |
| YLDs (Years Lived with Disability) | Tunisia | Both | 4 | 1990 | 2021 | 27.752645520403 | 0.95 (0.88 to 1.02) | 0 |
| YLDs (Years Lived with Disability) | Tunisia | Female | 3 | 1990 | 2021 | 19.692660706219 | 0.98 (0.88 to 1.08) | 0 |
| YLDs (Years Lived with Disability) | Tunisia | Male | 4 | 1990 | 2021 | 14.702858390365 | 0.78 (0.68 to 0.89) | 0 |
| YLDs (Years Lived with Disability) | Turkey | Both | 4 | 1990 | 2021 | 17.073975554913 | 1.45 (1.28 to 1.62) | 0 |
| YLDs (Years Lived with Disability) | Turkey | Female | 5 | 1990 | 2021 | 14.396332463143 | 1.38 (1.19 to 1.57) | 0 |
| YLDs (Years Lived with Disability) | Turkey | Male | 4 | 1990 | 2021 | 12.749544052852 | 1.63 (1.38 to 1.88) | 0 |
| YLDs (Years Lived with Disability) | Turkmenistan | Both | 5 | 1990 | 2021 | 4.719663353 | 1.02 (0.6 to 1.45) | 0 |
| YLDs (Years Lived with Disability) | Turkmenistan | Female | 5 | 1990 | 2021 | 4.514438641 | 0.94 (0.53 to 1.35) | 0 |
| YLDs (Years Lived with Disability) | Turkmenistan | Male | 5 | 1990 | 2021 | 6.451847921 | 1.09 (0.76 to 1.43) | 0 |
| YLDs (Years Lived with Disability) | Tuvalu | Both | 4 | 1990 | 2021 | 22.338360575013 | 0.95 (0.86 to 1.03) | 0 |
| YLDs (Years Lived with Disability) | Tuvalu | Female | 3 | 1990 | 2021 | 24.313911321355 | 1.1 (1.01 to 1.19) | 0 |
| YLDs (Years Lived with Disability) | Tuvalu | Male | 3 | 1990 | 2021 | 11.925969532121 | 0.82 (0.68 to 0.95) | 0 |
| YLDs (Years Lived with Disability) | Uganda | Both | 5 | 1990 | 2021 | 20.276876002198 | 0.69 (0.62 to 0.76) | 0 |
| YLDs (Years Lived with Disability) | Uganda | Female | 5 | 1990 | 2021 | 16.661612518256 | 0.83 (0.74 to 0.93) | 0 |
| YLDs (Years Lived with Disability) | Uganda | Male | 5 | 1990 | 2021 | 11.847457662221 | 0.64 (0.54 to 0.75) | 0 |
| YLDs (Years Lived with Disability) | Ukraine | Both | 5 | 1990 | 2021 | -0.100897513 | -0.01 (-0.19 to 0.17) | 0.92 |
| YLDs (Years Lived with Disability) | Ukraine | Female | 4 | 1990 | 2021 | -0.985477637 | -0.1 (-0.31 to 0.1) | 0.324 |
| YLDs (Years Lived with Disability) | Ukraine | Male | 4 | 1990 | 2021 | 4.455297234 | 0.52 (0.29 to 0.75) | 0 |
| YLDs (Years Lived with Disability) | United Arab Emirates | Both | 5 | 1990 | 2021 | 19.655806933946 | 0.86 (0.78 to 0.95) | 0 |
| YLDs (Years Lived with Disability) | United Arab Emirates | Female | 5 | 1990 | 2021 | 19.163748460769 | 1.37 (1.23 to 1.51) | 0 |
| YLDs (Years Lived with Disability) | United Arab Emirates | Male | 5 | 1990 | 2021 | 21.390542849636 | 1.21 (1.1 to 1.32) | 0 |
| YLDs (Years Lived with Disability) | United Kingdom | Both | 5 | 1990 | 2021 | 27.944253553192 | 2.42 (2.25 to 2.6) | 0 |
| YLDs (Years Lived with Disability) | United Kingdom | Female | 4 | 1990 | 2021 | 39.132301352854 | 2.46 (2.33 to 2.58) | 0 |
| YLDs (Years Lived with Disability) | United Kingdom | Male | 5 | 1990 | 2021 | 62.734907813958 | 2.82 (2.73 to 2.91) | 0 |
| YLDs (Years Lived with Disability) | United Republic of Tanzania | Both | 4 | 1990 | 2021 | 9.335013204 | 0.57 (0.45 to 0.69) | 0 |
| YLDs (Years Lived with Disability) | United Republic of Tanzania | Female | 5 | 1990 | 2021 | 6.270919264 | 0.58 (0.4 to 0.76) | 0 |
| YLDs (Years Lived with Disability) | United Republic of Tanzania | Male | 3 | 1990 | 2021 | 12.151534610532 | 0.54 (0.45 to 0.63) | 0 |
| YLDs (Years Lived with Disability) | United States Virgin Islands | Both | 3 | 1990 | 2021 | 18.766815133537 | 0.6 (0.53 to 0.66) | 0 |
| YLDs (Years Lived with Disability) | United States Virgin Islands | Female | 3 | 1990 | 2021 | 50.03202777 | 0.95 (0.91 to 0.99) | 0 |
| YLDs (Years Lived with Disability) | United States Virgin Islands | Male | 5 | 1990 | 2021 | 2.453195583 | 0.12 (0.02 to 0.21) | 0.014 |
| YLDs (Years Lived with Disability) | United States of America | Both | 3 | 1990 | 2021 | 43.966598913816 | 2.07 (1.97 to 2.16) | 0 |
| YLDs (Years Lived with Disability) | United States of America | Female | 4 | 1990 | 2021 | 45.038084822718 | 2.03 (1.94 to 2.12) | 0 |
| YLDs (Years Lived with Disability) | United States of America | Male | 4 | 1990 | 2021 | 35.629275853565 | 1.99 (1.88 to 2.1) | 0 |
| YLDs (Years Lived with Disability) | Uruguay | Both | 5 | 1990 | 2021 | 23.685078911561 | 1.58 (1.45 to 1.71) | 0 |
| YLDs (Years Lived with Disability) | Uruguay | Female | 5 | 1990 | 2021 | 18.888384519272 | 1.46 (1.31 to 1.62) | 0 |
| YLDs (Years Lived with Disability) | Uruguay | Male | 5 | 1990 | 2021 | 24.162182699857 | 1.81 (1.66 to 1.95) | 0 |
| YLDs (Years Lived with Disability) | Uzbekistan | Both | 4 | 1990 | 2021 | 2.372171887 | 0.5 (0.09 to 0.91) | 0.018 |
| YLDs (Years Lived with Disability) | Uzbekistan | Female | 5 | 1990 | 2021 | 4.409729001 | 0.39 (0.22 to 0.56) | 0 |
| YLDs (Years Lived with Disability) | Uzbekistan | Male | 5 | 1990 | 2021 | 9.682126323 | 0.66 (0.53 to 0.8) | 0 |
| YLDs (Years Lived with Disability) | Vanuatu | Both | 2 | 1990 | 2021 | 23.366884639704 | 0.5 (0.46 to 0.54) | 0 |
| YLDs (Years Lived with Disability) | Vanuatu | Female | 2 | 1990 | 2021 | 23.54232153 | 0.52 (0.47 to 0.56) | 0 |
| YLDs (Years Lived with Disability) | Vanuatu | Male | 2 | 1990 | 2021 | 8.512891627 | 0.43 (0.33 to 0.53) | 0 |
| YLDs (Years Lived with Disability) | Venezuela (Bolivarian Republic of) | Both | 3 | 1990 | 2021 | 14.279895584144 | 0.67 (0.58 to 0.76) | 0 |
| YLDs (Years Lived with Disability) | Venezuela (Bolivarian Republic of) | Female | 2 | 1990 | 2021 | 20.594894329756 | 0.84 (0.76 to 0.91) | 0 |
| YLDs (Years Lived with Disability) | Venezuela (Bolivarian Republic of) | Male | 4 | 1990 | 2021 | 7.403935418 | 0.48 (0.35 to 0.61) | 0 |
| YLDs (Years Lived with Disability) | Viet Nam | Both | 5 | 1990 | 2021 | 28.296670559403 | 1.45 (1.35 to 1.55) | 0 |
| YLDs (Years Lived with Disability) | Viet Nam | Female | 4 | 1990 | 2021 | 36.841766214639 | 1.63 (1.54 to 1.71) | 0 |
| YLDs (Years Lived with Disability) | Viet Nam | Male | 2 | 1990 | 2021 | 50.598931628442 | 1.1 (1.05 to 1.14) | 0 |
| YLDs (Years Lived with Disability) | Western Europe | Both | 3 | 1990 | 2021 | 35.953195840199 | 2.46 (2.33 to 2.6) | 0 |
| YLDs (Years Lived with Disability) | Western Europe | Female | 5 | 1990 | 2021 | 24.599207075321 | 2.34 (2.15 to 2.53) | 0 |
| YLDs (Years Lived with Disability) | Western Europe | Male | 3 | 1990 | 2021 | 21.193006515822 | 2.77 (2.51 to 3.03) | 0 |
| YLDs (Years Lived with Disability) | Western Sub-Saharan Africa | Both | 4 | 1990 | 2021 | 33.761896809068 | 0.4 (0.38 to 0.42) | 0 |
| YLDs (Years Lived with Disability) | Western Sub-Saharan Africa | Female | 5 | 1990 | 2021 | 15.443758671819 | 0.45 (0.39 to 0.5) | 0 |
| YLDs (Years Lived with Disability) | Western Sub-Saharan Africa | Male | 5 | 1990 | 2021 | 20.184781811064 | 0.31 (0.28 to 0.35) | 0 |
| YLDs (Years Lived with Disability) | Yemen | Both | 4 | 1990 | 2021 | 34.671275390874 | 1.04 (0.98 to 1.1) | 0 |
| YLDs (Years Lived with Disability) | Yemen | Female | 5 | 1990 | 2021 | 16.605471505731 | 1.03 (0.91 to 1.16) | 0 |
| YLDs (Years Lived with Disability) | Yemen | Male | 4 | 1990 | 2021 | 13.333925471681 | 0.81 (0.69 to 0.93) | 0 |
| YLDs (Years Lived with Disability) | Zambia | Both | 5 | 1990 | 2021 | 23.026102613147 | 1.25 (1.14 to 1.36) | 0 |
| YLDs (Years Lived with Disability) | Zambia | Female | 5 | 1990 | 2021 | 17.429236395785 | 1.28 (1.13 to 1.42) | 0 |
| YLDs (Years Lived with Disability) | Zambia | Male | 5 | 1990 | 2021 | 19.82660284 | 1.14 (1.03 to 1.25) | 0 |
| YLDs (Years Lived with Disability) | Zimbabwe | Both | 4 | 1990 | 2021 | 0.389571929 | 0.02 (-0.09 to 0.14) | 0.697 |
| YLDs (Years Lived with Disability) | Zimbabwe | Female | 4 | 1990 | 2021 | 1.757804892 | 0.12 (-0.01 to 0.25) | 0.079 |
| YLDs (Years Lived with Disability) | Zimbabwe | Male | 4 | 1990 | 2021 | -5.031583794 | -0.25 (-0.34 to -0.15) | 0 |

# **TableS4 Correlation Coefficients (r) and P-Values Between Age-Standardized Prevalence Rates (ASPR), Age-Standardized Years Lived with Disability Rates (ASYR), and Socio-Demographic Index (SDI) by Global Burden of Disease Region, 1990–2021**

| **Location** | **r_Prevalence** | **P_Value** | **r_YLDs** | **P_Value** |
| --- | --- | --- | --- | --- |
| Andean Latin America | 0.979 | 2.37e-22 | 0.98 | 1.08e-22 |
| Australasia | 0.949 | 1.52e-16 | 0.948 | 2.03e-16 |
| Caribbean | 0.897 | 3.63e-12 | 0.898 | 3.32e-12 |
| Central Asia | 0.631 | 0.000108 | 0.637 | 8.95e-05 |
| Central Europe | 0.93 | 1.47e-14 | 0.93 | 1.31e-14 |
| Central Latin America | 0.969 | 1.1e-19 | 0.969 | 9.93e-20 |
| Central Sub-Saharan Africa | 0.951 | 6.76e-17 | 0.951 | 8.55e-17 |
| East Asia | 0.943 | 6.3e-16 | 0.948 | 2.11e-16 |
| Eastern Europe | 0.874 | 6.72e-11 | 0.874 | 6.73e-11 |
| Eastern Sub-Saharan Africa | 0.982 | 2.2e-23 | 0.983 | 1.04e-23 |
| Global | 0.992 | 1.78e-28 | 0.992 | 2.86e-28 |
| High-income Asia Pacific | 0.928 | 1.99e-14 | 0.929 | 1.65e-14 |
| High-income North America | 0.977 | 1.31e-21 | 0.977 | 1.25e-21 |
| North Africa and Middle East | 0.997 | 5.03e-72 | 0.997 | 5.4e-72 |
| Oceania | 0.953 | 3.68e-17 | 0.955 | 2.16e-17 |
| South Asia | 0.993 | 1.75e-58 | 0.993 | 1.44e-58 |
| Southeast Asia | 0.985 | 2.14e-24 | 0.985 | 1.37e-24 |
| Southern Latin America | 0.919 | 1.24e-13 | 0.919 | 1.19e-13 |
| Southern Sub-Saharan Africa | 0.772 | 2.31e-07 | 0.773 | 2.15e-07 |
| Tropical Latin America | 0.989 | 1.59e-26 | 0.989 | 1.99e-26 |
| Western Europe | 0.975 | 2.8e-21 | 0.975 | 3.07e-21 |
| Western Sub-Saharan Africa | 0.782 | 1.25e-07 | 0.803 | 3.26e-08 |

# TableS5 Cross-National Inequality in Age-Standardized Prevalence of Heart Failure Attributable to Atrial Fibrillation and Flutter: Absolute Differences.

| **Region** | **Measure** | **Sex** | **Year** | **rlm** | **rlm_lower** | **rlm_upper** |
| --- | --- | --- | --- | --- | --- | --- |
| All included | YLDs (Years Lived with Disability) | Male | 1990 | 0.242163485119031 | 0.17720412683606 | 0.307122843402002 |
| All included | Prevalence | Male | 1990 | 2.64716996467389 | 1.92617363400076 | 3.36816629534701 |
| All included | YLDs (Years Lived with Disability) | Female | 1990 | 0.41022219287712 | 0.324128483220638 | 0.496315902533602 |
| All included | Prevalence | Female | 1990 | 4.54283851265854 | 3.57569333549375 | 5.50998368982333 |
| All included | YLDs (Years Lived with Disability) | Both | 1990 | 0.334368471195463 | 0.258446705545241 | 0.410290236845685 |
| All included | Prevalence | Both | 1990 | 3.6973958988241 | 2.84451803022617 | 4.55027376742203 |
| All included | YLDs (Years Lived with Disability) | Male | 2021 | 1.14694190864068 | 0.941881191581393 | 1.35200262569997 |
| All included | Prevalence | Male | 2021 | 12.7761381800029 | 10.4706102065536 | 15.0816661534521 |
| All included | YLDs (Years Lived with Disability) | Female | 2021 | 1.57968153805672 | 1.33522931590969 | 1.82413376020375 |
| All included | Prevalence | Female | 2021 | 17.6077996087133 | 14.8706836359677 | 20.3449155814588 |
| All included | YLDs (Years Lived with Disability) | Both | 2021 | 1.35277132606046 | 1.12990135774323 | 1.57564129437769 |
| All included | Prevalence | Both | 2021 | 15.039660943365 | 12.5365856936841 | 17.5427361930459 |
| Low SDI | YLDs (Years Lived with Disability) | Male | 1990 | -0.011839346 | -0.09068112 | 0.0670024279074788 |
| Low SDI | Prevalence | Male | 1990 | -0.145935779 | -1.042144867 | 0.750273308009751 |
| Low SDI | YLDs (Years Lived with Disability) | Female | 1990 | -0.014624448 | -0.100497445 | 0.0712485499173529 |
| Low SDI | Prevalence | Female | 1990 | -0.182349877 | -1.154202721 | 0.789502967115686 |
| Low SDI | YLDs (Years Lived with Disability) | Both | 1990 | -0.010939406 | -0.091990573 | 0.0701117607091624 |
| Low SDI | Prevalence | Both | 1990 | -0.139432297 | -1.063851251 | 0.78498665734813 |
| Low SDI | YLDs (Years Lived with Disability) | Male | 2021 | 0.0233273513870611 | -0.044823433 | 0.0914781353021189 |
| Low SDI | Prevalence | Male | 2021 | 0.260045856746723 | -0.511151943 | 1.03124365627682 |
| Low SDI | YLDs (Years Lived with Disability) | Female | 2021 | 0.0334939852178866 | -0.065660571 | 0.132648541404078 |
| Low SDI | Prevalence | Female | 2021 | 0.370569420850588 | -0.756985665 | 1.49812450629805 |
| Low SDI | YLDs (Years Lived with Disability) | Both | 2021 | 0.0303835910657833 | -0.044505427 | 0.105272609231036 |
| Low SDI | Prevalence | Both | 2021 | 0.340261011540524 | -0.502379472 | 1.18290149502222 |
| Low-middle SDI | YLDs (Years Lived with Disability) | Male | 1990 | -0.011380157 | -0.057443987 | 0.0346836727044063 |
| Low-middle SDI | Prevalence | Male | 1990 | -0.136082777 | -0.655300462 | 0.383134907958872 |
| Low-middle SDI | YLDs (Years Lived with Disability) | Female | 1990 | 0.0450158084443838 | -0.025770563 | 0.115802179631691 |
| Low-middle SDI | Prevalence | Female | 1990 | 0.497035510004508 | -0.317552141 | 1.3116231613025 |
| Low-middle SDI | YLDs (Years Lived with Disability) | Both | 1990 | 0.0158936167660074 | -0.039621625 | 0.0714088584830929 |
| Low-middle SDI | Prevalence | Both | 1990 | 0.167508506148689 | -0.459834965 | 0.794851977421207 |
| Low-middle SDI | YLDs (Years Lived with Disability) | Male | 2021 | 0.0151272330463728 | -0.117785795 | 0.148040261139284 |
| Low-middle SDI | Prevalence | Male | 2021 | 0.162292653164862 | -1.297615651 | 1.62220095697941 |
| Low-middle SDI | YLDs (Years Lived with Disability) | Female | 2021 | 0.0675601327102647 | -0.069436609 | 0.204556874588607 |
| Low-middle SDI | Prevalence | Female | 2021 | 0.732737219234365 | -0.795532684 | 2.26100712249484 |
| Low-middle SDI | YLDs (Years Lived with Disability) | Both | 2021 | 0.0345511140416353 | -0.098693741 | 0.167795969478796 |
| Low-middle SDI | Prevalence | Both | 2021 | 0.368288155975455 | -1.138955532 | 1.87553184422662 |
| Middle SDI | YLDs (Years Lived with Disability) | Male | 1990 | 0.102955928220041 | -0.026627393 | 0.232539249579405 |
| Middle SDI | Prevalence | Male | 1990 | 1.15407721593799 | -0.309305106 | 2.61745953748507 |
| Middle SDI | YLDs (Years Lived with Disability) | Female | 1990 | 0.1043695528986 | -0.036635888 | 0.245374993773635 |
| Middle SDI | Prevalence | Female | 1990 | 1.16137978117942 | -0.437350421 | 2.76010998340899 |
| Middle SDI | YLDs (Years Lived with Disability) | Both | 1990 | 0.0990084733471469 | -0.034336058 | 0.232353004872408 |
| Middle SDI | Prevalence | Both | 1990 | 1.10796374915073 | -0.404152282 | 2.62007978027316 |
| Middle SDI | YLDs (Years Lived with Disability) | Male | 2021 | 0.255975675918685 | -0.058537331 | 0.570488683304897 |
| Middle SDI | Prevalence | Male | 2021 | 2.87648247916437 | -0.684039319 | 6.43700427734184 |
| Middle SDI | YLDs (Years Lived with Disability) | Female | 2021 | 0.304953929863801 | -0.068131117 | 0.678038976448804 |
| Middle SDI | Prevalence | Female | 2021 | 3.39367659907622 | -0.929211064 | 7.7165642617047 |
| Middle SDI | YLDs (Years Lived with Disability) | Both | 2021 | 0.283782245877593 | -0.049632164 | 0.617196655778547 |
| Middle SDI | Prevalence | Both | 2021 | 3.16930300245549 | -0.589542305 | 6.9281483098586 |
| High-middle SDI | YLDs (Years Lived with Disability) | Male | 1990 | 0.273209238005019 | 0.0173758246242345 | 0.529042651385803 |
| High-middle SDI | Prevalence | Male | 1990 | 2.98428168934027 | 0.186420234208479 | 5.78214314447206 |
| High-middle SDI | YLDs (Years Lived with Disability) | Female | 1990 | 0.413901585930138 | 0.0461630468078275 | 0.781640125052448 |
| High-middle SDI | Prevalence | Female | 1990 | 4.58422154687416 | 0.464255989198218 | 8.70418710455011 |
| High-middle SDI | YLDs (Years Lived with Disability) | Both | 1990 | 0.357216007258373 | 0.0659796416551495 | 0.648452372861597 |
| High-middle SDI | Prevalence | Both | 1990 | 3.93794251282631 | 0.707203985817911 | 7.1686810398347 |
| High-middle SDI | YLDs (Years Lived with Disability) | Male | 2021 | 0.0926036398664679 | -0.357530564 | 0.542737843913156 |
| High-middle SDI | Prevalence | Male | 2021 | 1.02011347939542 | -3.971887045 | 6.0121140038704 |
| High-middle SDI | YLDs (Years Lived with Disability) | Female | 2021 | 0.0410563962598707 | -0.578145508 | 0.660258300649471 |
| High-middle SDI | Prevalence | Female | 2021 | 0.431355338130512 | -6.620324681 | 7.48303535740893 |
| High-middle SDI | YLDs (Years Lived with Disability) | Both | 2021 | 0.0780678848952161 | -0.420713166 | 0.576848935895316 |
| High-middle SDI | Prevalence | Both | 2021 | 0.81372906196439 | -4.698084533 | 6.32554265659439 |
| High SDI | YLDs (Years Lived with Disability) | Male | 1990 | 0.511812424019986 | 0.274089671203559 | 0.749535176836413 |
| High SDI | Prevalence | Male | 1990 | 5.6516202566181 | 2.98644763871432 | 8.31679287452188 |
| High SDI | YLDs (Years Lived with Disability) | Female | 1990 | 1.08602791865468 | 0.559611082502748 | 1.61244475480661 |
| High SDI | Prevalence | Female | 1990 | 12.0289719530582 | 6.15368320002524 | 17.9042607060911 |
| High SDI | YLDs (Years Lived with Disability) | Both | 1990 | 0.8047465457826 | 0.462157985335165 | 1.14733510623003 |
| High SDI | Prevalence | Both | 1990 | 8.89762954239256 | 5.06267186370468 | 12.7325872210804 |
| High SDI | YLDs (Years Lived with Disability) | Male | 2021 | 1.54728554233434 | 0.770576796942167 | 2.32399428772651 |
| High SDI | Prevalence | Male | 2021 | 17.1154920528896 | 8.01329054159679 | 26.2176935641825 |
| High SDI | YLDs (Years Lived with Disability) | Female | 2021 | 1.38251102738936 | 0.176400030128743 | 2.58862202464998 |
| High SDI | Prevalence | Female | 2021 | 15.2432548759548 | 1.81693877900952 | 28.6695709729001 |
| High SDI | YLDs (Years Lived with Disability) | Both | 2021 | 1.43537911713353 | 0.37536848338766 | 2.49538975087941 |
| High SDI | Prevalence | Both | 2021 | 15.9142469669373 | 4.37724835808957 | 27.4512455757851 |
| Eastern Sub-Saharan Africa | YLDs (Years Lived with Disability) | Male | 1990 | 0.0732321819956488 | -0.023860672 | 0.170325036082087 |
| Eastern Sub-Saharan Africa | Prevalence | Male | 1990 | 0.826236862499881 | -0.246897969 | 1.89937169410069 |
| Eastern Sub-Saharan Africa | YLDs (Years Lived with Disability) | Female | 1990 | 0.0618661702052747 | -0.013001888 | 0.136734227965201 |
| Eastern Sub-Saharan Africa | Prevalence | Female | 1990 | 0.689871366908355 | -0.171874139 | 1.55161687278551 |
| Eastern Sub-Saharan Africa | YLDs (Years Lived with Disability) | Both | 1990 | 0.068782697936594 | -0.01534359 | 0.152908986245653 |
| Eastern Sub-Saharan Africa | Prevalence | Both | 1990 | 0.774784874107976 | -0.175631605 | 1.72520135364638 |
| Eastern Sub-Saharan Africa | YLDs (Years Lived with Disability) | Male | 2021 | 0.0695985605388559 | -0.023369243 | 0.162566364537884 |
| Eastern Sub-Saharan Africa | Prevalence | Male | 2021 | 0.761390163549752 | -0.375988261 | 1.89876858831189 |
| Eastern Sub-Saharan Africa | YLDs (Years Lived with Disability) | Female | 2021 | 0.132954736393505 | 0.061296152145798 | 0.204613320641212 |
| Eastern Sub-Saharan Africa | Prevalence | Female | 2021 | 1.46721920771773 | 0.723051204478859 | 2.2113872109566 |
| Eastern Sub-Saharan Africa | YLDs (Years Lived with Disability) | Both | 2021 | 0.0957729182775751 | 0.00584778873373694 | 0.185698047821413 |
| Eastern Sub-Saharan Africa | Prevalence | Both | 2021 | 1.07196797773566 | 0.0625685093749384 | 2.08136744609638 |
| Western Sub-Saharan Africa | YLDs (Years Lived with Disability) | Male | 1990 | -0.018629592 | -0.11592077 | 0.0786615862423896 |
| Western Sub-Saharan Africa | Prevalence | Male | 1990 | -0.206064231 | -1.311574123 | 0.89944566023974 |
| Western Sub-Saharan Africa | YLDs (Years Lived with Disability) | Female | 1990 | 0.103603335942465 | 0.00959385933812759 | 0.197612812546803 |
| Western Sub-Saharan Africa | Prevalence | Female | 1990 | 1.16997968611557 | 0.10957705565842 | 2.23038231657271 |
| Western Sub-Saharan Africa | YLDs (Years Lived with Disability) | Both | 1990 | 0.0409911947881698 | -0.060448536 | 0.142430925398656 |
| Western Sub-Saharan Africa | Prevalence | Both | 1990 | 0.460680129914327 | -0.688204964 | 1.60956522393923 |
| Western Sub-Saharan Africa | YLDs (Years Lived with Disability) | Male | 2021 | 0.0398066940781201 | -0.075610537 | 0.155223925408219 |
| Western Sub-Saharan Africa | Prevalence | Male | 2021 | 0.454181026178032 | -0.864308309 | 1.77267036150949 |
| Western Sub-Saharan Africa | YLDs (Years Lived with Disability) | Female | 2021 | 0.131949102073491 | -0.032928554 | 0.296826758302956 |
| Western Sub-Saharan Africa | Prevalence | Female | 2021 | 1.49646539546372 | -0.366726828 | 3.35965761868891 |
| Western Sub-Saharan Africa | YLDs (Years Lived with Disability) | Both | 2021 | 0.0878510310430496 | -0.050918339 | 0.226620401518824 |
| Western Sub-Saharan Africa | Prevalence | Both | 2021 | 1.00039177941529 | -0.57944533 | 2.58022888922025 |
| Central Sub-Saharan Africa | YLDs (Years Lived with Disability) | Male | 1990 | 0.0808373521057974 | -0.001937592 | 0.163612296201838 |
| Central Sub-Saharan Africa | Prevalence | Male | 1990 | 0.918663190113544 | -0.046659159 | 1.88398553906502 |
| Central Sub-Saharan Africa | YLDs (Years Lived with Disability) | Female | 1990 | 0.283421402167174 | 0.00314734795874255 | 0.563695456375606 |
| Central Sub-Saharan Africa | Prevalence | Female | 1990 | 3.18375643178995 | -0.012320119 | 6.37983298269346 |
| Central Sub-Saharan Africa | YLDs (Years Lived with Disability) | Both | 1990 | 0.183896763458086 | -0.039443441 | 0.407236967883011 |
| Central Sub-Saharan Africa | Prevalence | Both | 1990 | 2.11689468604847 | -0.520305246 | 4.7540946178383 |
| Central Sub-Saharan Africa | YLDs (Years Lived with Disability) | Male | 2021 | 0.139731415359779 | -0.001131381 | 0.280594211570869 |
| Central Sub-Saharan Africa | Prevalence | Male | 2021 | 1.57996655729551 | -0.012263602 | 3.17219671671944 |
| Central Sub-Saharan Africa | YLDs (Years Lived with Disability) | Female | 2021 | 0.252650626204117 | 0.188106850231851 | 0.317194402176383 |
| Central Sub-Saharan Africa | Prevalence | Female | 2021 | 2.83270945187534 | 2.07969554360454 | 3.58572336014614 |
| Central Sub-Saharan Africa | YLDs (Years Lived with Disability) | Both | 2021 | 0.185215411928851 | 0.0521977112024086 | 0.318233112655293 |
| Central Sub-Saharan Africa | Prevalence | Both | 2021 | 2.078566065 | 0.62488860677077 | 3.53224352326722 |
| North Africa and Middle East | YLDs (Years Lived with Disability) | Male | 1990 | 0.038539088943097 | 0.00555153379109897 | 0.071526644095095 |
| North Africa and Middle East | Prevalence | Male | 1990 | 0.417501869802464 | 0.0628200846117701 | 0.772183654993158 |
| North Africa and Middle East | YLDs (Years Lived with Disability) | Female | 1990 | 0.0605503436545768 | 0.00930368266238327 | 0.11179700464677 |
| North Africa and Middle East | Prevalence | Female | 1990 | 0.676503422100592 | 0.106908467133102 | 1.24609837706808 |
| North Africa and Middle East | YLDs (Years Lived with Disability) | Both | 1990 | 0.0479926692852578 | 0.00421081542233395 | 0.0917745231481816 |
| North Africa and Middle East | Prevalence | Both | 1990 | 0.534586150657131 | 0.0364144089947127 | 1.03275789231955 |
| North Africa and Middle East | YLDs (Years Lived with Disability) | Male | 2021 | 0.186384356986674 | 0.0164516206304183 | 0.35631709334293 |
| North Africa and Middle East | Prevalence | Male | 2021 | 2.04347813010608 | 0.14381194582247 | 3.94314431438969 |
| North Africa and Middle East | YLDs (Years Lived with Disability) | Female | 2021 | 0.223359472813348 | 0.0273418418166453 | 0.419377103810051 |
| North Africa and Middle East | Prevalence | Female | 2021 | 2.48460972286147 | 0.296993485486225 | 4.67222596023672 |
| North Africa and Middle East | YLDs (Years Lived with Disability) | Both | 2021 | 0.203657659008213 | 0.0161984168149508 | 0.391116901201475 |
| North Africa and Middle East | Prevalence | Both | 2021 | 2.25412023938753 | 0.165809830617766 | 4.3424306481573 |
| Oceania | YLDs (Years Lived with Disability) | Male | 1990 | 0.0927708948597782 | -0.010019411 | 0.195561201208215 |
| Oceania | Prevalence | Male | 1990 | 1.0411479313521 | -0.111251534 | 2.19354739668591 |
| Oceania | YLDs (Years Lived with Disability) | Female | 1990 | 0.199144157763033 | 0.0487833662835681 | 0.349504949242499 |
| Oceania | Prevalence | Female | 1990 | 2.2211507577058 | 0.545944466234731 | 3.89635704917686 |
| Oceania | YLDs (Years Lived with Disability) | Both | 1990 | 0.138871458245271 | 0.0155332193002597 | 0.262209697190282 |
| Oceania | Prevalence | Both | 1990 | 1.55074553312086 | 0.171135770958415 | 2.9303552952833 |
| Oceania | YLDs (Years Lived with Disability) | Male | 2021 | 0.720940651510195 | 0.0462221703746331 | 1.39565913264576 |
| Oceania | Prevalence | Male | 2021 | 8.04860385813411 | 0.481329793506208 | 15.615877922762 |
| Oceania | YLDs (Years Lived with Disability) | Female | 2021 | 1.51075170457699 | 0.488549698353634 | 2.53295371080035 |
| Oceania | Prevalence | Female | 2021 | 17.0738113465959 | 5.48673561429749 | 28.6608870788944 |
| Oceania | YLDs (Years Lived with Disability) | Both | 2021 | 1.10734291308909 | 0.303036751163945 | 1.91164907501423 |
| Oceania | Prevalence | Both | 2021 | 12.4709184815136 | 3.37987009949356 | 21.5619668635336 |
| South Asia | YLDs (Years Lived with Disability) | Male | 1990 | 0.010128013183219 | -0.148136893 | 0.168392919222937 |
| South Asia | Prevalence | Male | 1990 | 0.111309933018313 | -1.697917171 | 1.92053703738046 |
| South Asia | YLDs (Years Lived with Disability) | Female | 1990 | 0.0376333163308001 | -0.020059376 | 0.0953260083145329 |
| South Asia | Prevalence | Female | 1990 | 0.427334979071469 | -0.229680096 | 1.08435005414745 |
| South Asia | YLDs (Years Lived with Disability) | Both | 1990 | 0.0240004306741529 | -0.068878581 | 0.116879442013238 |
| South Asia | Prevalence | Both | 1990 | 0.270618574118567 | -0.79811221 | 1.3393493583772 |
| South Asia | YLDs (Years Lived with Disability) | Male | 2021 | -0.142303553 | -0.778501848 | 0.493894742037803 |
| South Asia | Prevalence | Male | 2021 | -1.60478974 | -8.85576215 | 5.64618267033566 |
| South Asia | YLDs (Years Lived with Disability) | Female | 2021 | 0.0463746937040846 | -0.45887072 | 0.551620107815605 |
| South Asia | Prevalence | Female | 2021 | 0.551466745255286 | -5.163167144 | 6.2661006340462 |
| South Asia | YLDs (Years Lived with Disability) | Both | 2021 | -0.045183229 | -0.568340011 | 0.477973552565454 |
| South Asia | Prevalence | Both | 2021 | -0.494873234 | -6.425040172 | 5.4352937032517 |
| Caribbean | YLDs (Years Lived with Disability) | Male | 1990 | 0.220894106452309 | -0.069682409 | 0.511470622265602 |
| Caribbean | Prevalence | Male | 1990 | 2.43573588679128 | -0.867982569 | 5.73945434220725 |
| Caribbean | YLDs (Years Lived with Disability) | Female | 1990 | 0.468841737444476 | 0.10721951070928 | 0.830463964179673 |
| Caribbean | Prevalence | Female | 1990 | 5.22957815128943 | 1.18752091091565 | 9.27163539166322 |
| Caribbean | YLDs (Years Lived with Disability) | Both | 1990 | 0.332245412132823 | -0.028563713 | 0.693054537688736 |
| Caribbean | Prevalence | Both | 1990 | 3.67208218986098 | -0.420462791 | 7.76462717096436 |
| Caribbean | YLDs (Years Lived with Disability) | Male | 2021 | 1.20359018379407 | 0.175840711476142 | 2.231339656 |
| Caribbean | Prevalence | Male | 2021 | 13.4446390889666 | 1.87684134238702 | 25.0124368355463 |
| Caribbean | YLDs (Years Lived with Disability) | Female | 2021 | 1.59682443761432 | 0.427660393167964 | 2.76598848206068 |
| Caribbean | Prevalence | Female | 2021 | 17.8400713744385 | 4.77223599801815 | 30.9079067508589 |
| Caribbean | YLDs (Years Lived with Disability) | Both | 2021 | 1.46490544161582 | 0.311703428373667 | 2.61810745485798 |
| Caribbean | Prevalence | Both | 2021 | 16.3889849474363 | 3.4302570208432 | 29.3477128740294 |
| Southeast Asia | YLDs (Years Lived with Disability) | Male | 1990 | 0.0645342935359768 | 0.0217511465582533 | 0.1073174405137 |
| Southeast Asia | Prevalence | Male | 1990 | 0.730861929069152 | 0.23267036414378 | 1.22905349399452 |
| Southeast Asia | YLDs (Years Lived with Disability) | Female | 1990 | 0.136907945479291 | 0.0310241753577544 | 0.242791715600828 |
| Southeast Asia | Prevalence | Female | 1990 | 1.56121451127849 | 0.370155411063397 | 2.75227361149359 |
| Southeast Asia | YLDs (Years Lived with Disability) | Both | 1990 | 0.119301618111677 | 0.0865045674920036 | 0.152098668731351 |
| Southeast Asia | Prevalence | Both | 1990 | 1.35844273349914 | 0.987748453910931 | 1.72913701308734 |
| Southeast Asia | YLDs (Years Lived with Disability) | Male | 2021 | 0.263338024766392 | 0.083388812081319 | 0.443287237451465 |
| Southeast Asia | Prevalence | Male | 2021 | 2.97440304615606 | 0.978514812133556 | 4.97029128017856 |
| Southeast Asia | YLDs (Years Lived with Disability) | Female | 2021 | 0.43376916309118 | 0.167299456166897 | 0.700238870015463 |
| Southeast Asia | Prevalence | Female | 2021 | 4.89624273081008 | 1.87647925485269 | 7.91600620676747 |
| Southeast Asia | YLDs (Years Lived with Disability) | Both | 2021 | 0.352226170984732 | 0.171141465586832 | 0.533310876382633 |
| Southeast Asia | Prevalence | Both | 2021 | 3.97831247694871 | 1.92326934967057 | 6.03335560422686 |
| Southern Sub-Saharan Africa | YLDs (Years Lived with Disability) | Male | 1990 | 0.265220006820721 | 0.0865134367326597 | 0.443926576908782 |
| Southern Sub-Saharan Africa | Prevalence | Male | 1990 | 3.00084750150998 | 0.984434206413547 | 5.01726079660641 |
| Southern Sub-Saharan Africa | YLDs (Years Lived with Disability) | Female | 1990 | 0.353176543900085 | -0.011633313 | 0.717986400427903 |
| Southern Sub-Saharan Africa | Prevalence | Female | 1990 | 3.98608212710986 | -0.130238642 | 8.10240289590863 |
| Southern Sub-Saharan Africa | YLDs (Years Lived with Disability) | Both | 1990 | 0.306315951032233 | 0.0292411320743973 | 0.583390769990068 |
| Southern Sub-Saharan Africa | Prevalence | Both | 1990 | 3.46070569255849 | 0.331722317774613 | 6.58968906734237 |
| Southern Sub-Saharan Africa | YLDs (Years Lived with Disability) | Male | 2021 | 0.533218794742122 | 0.328008951457342 | 0.738428638026903 |
| Southern Sub-Saharan Africa | Prevalence | Male | 2021 | 5.95084890868132 | 3.64956341804127 | 8.25213439932138 |
| Southern Sub-Saharan Africa | YLDs (Years Lived with Disability) | Female | 2021 | 1.00071836474723 | 0.776736468667975 | 1.22470026082648 |
| Southern Sub-Saharan Africa | Prevalence | Female | 2021 | 11.2549881470607 | 8.65649878046221 | 13.8534775136591 |
| Southern Sub-Saharan Africa | YLDs (Years Lived with Disability) | Both | 2021 | 0.770751943454762 | 0.579050708602597 | 0.962453178306926 |
| Southern Sub-Saharan Africa | Prevalence | Both | 2021 | 8.64371578668566 | 6.47223092888533 | 10.815200644486 |
| Central Latin America | YLDs (Years Lived with Disability) | Male | 1990 | 0.181729971997381 | 0.0363111387708035 | 0.327148805223959 |
| Central Latin America | Prevalence | Male | 1990 | 2.09660503067587 | 0.483125093401677 | 3.71008496795007 |
| Central Latin America | YLDs (Years Lived with Disability) | Female | 1990 | 0.155643162772052 | 0.0216378524329306 | 0.289648473111172 |
| Central Latin America | Prevalence | Female | 1990 | 1.75686769410888 | 0.208378776918933 | 3.30535661129883 |
| Central Latin America | YLDs (Years Lived with Disability) | Both | 1990 | 0.185444447683365 | 0.06590063412759 | 0.30498826123914 |
| Central Latin America | Prevalence | Both | 1990 | 2.10491875945082 | 0.797648603950526 | 3.41218891495111 |
| Central Latin America | YLDs (Years Lived with Disability) | Male | 2021 | 0.713415164476712 | 0.434120420378024 | 0.9927099085754 |
| Central Latin America | Prevalence | Male | 2021 | 8.07640848280252 | 4.75468875433871 | 11.3981282112663 |
| Central Latin America | YLDs (Years Lived with Disability) | Female | 2021 | 0.87245240694971 | 0.405556996560452 | 1.33934781733897 |
| Central Latin America | Prevalence | Female | 2021 | 9.8329335412523 | 4.39107256834855 | 15.274794514156 |
| Central Latin America | YLDs (Years Lived with Disability) | Both | 2021 | 0.799501130000246 | 0.553861557155428 | 1.04514070284506 |
| Central Latin America | Prevalence | Both | 2021 | 9.00702930914881 | 5.82730836867207 | 12.1867502496256 |
| Central Asia | YLDs (Years Lived with Disability) | Male | 1990 | 0.0395979990304263 | 0.00969340515027716 | 0.0695025929105754 |
| Central Asia | Prevalence | Male | 1990 | 0.427847630621321 | 0.0953001202425641 | 0.760395141000078 |
| Central Asia | YLDs (Years Lived with Disability) | Female | 1990 | 0.154727135263103 | 0.0241774537943718 | 0.285276816731834 |
| Central Asia | Prevalence | Female | 1990 | 1.71194743904699 | 0.25986723026098 | 3.164027648 |
| Central Asia | YLDs (Years Lived with Disability) | Both | 1990 | 0.0992925041319596 | 0.018793099279379 | 0.17979190898454 |
| Central Asia | Prevalence | Both | 1990 | 1.09127495422558 | 0.197015027884874 | 1.98553488056629 |
| Central Asia | YLDs (Years Lived with Disability) | Male | 2021 | 0.2668573134351 | 0.010670314039357 | 0.523044312830843 |
| Central Asia | Prevalence | Male | 2021 | 2.95323655006575 | 0.112337192540005 | 5.7941359075915 |
| Central Asia | YLDs (Years Lived with Disability) | Female | 2021 | 0.413628822954184 | 0.0962776114121001 | 0.730980034496268 |
| Central Asia | Prevalence | Female | 2021 | 4.59329250676645 | 1.05686356848882 | 8.12972144504408 |
| Central Asia | YLDs (Years Lived with Disability) | Both | 2021 | 0.337444374105184 | 0.0548756129869926 | 0.620013135223375 |
| Central Asia | Prevalence | Both | 2021 | 3.72741535387648 | 0.598277490518506 | 6.85655321723445 |
| East Asia | YLDs (Years Lived with Disability) | Male | 1990 | 0.185080949293878 | -0.469916375 | 0.84007827398574 |
| East Asia | Prevalence | Male | 1990 | 2.06658440720711 | -5.133410739 | 9.26657955330283 |
| East Asia | YLDs (Years Lived with Disability) | Female | 1990 | 0.185189957718591 | -0.119605259 | 0.48998517413625 |
| East Asia | Prevalence | Female | 1990 | 2.05131877081808 | -1.406754007 | 5.50939154908828 |
| East Asia | YLDs (Years Lived with Disability) | Both | 1990 | 0.19185943528424 | -0.269025855 | 0.652744725250661 |
| East Asia | Prevalence | Both | 1990 | 2.13351320258208 | -2.983008205 | 7.25003461015796 |
| East Asia | YLDs (Years Lived with Disability) | Male | 2021 | 1.61125521135455 | -0.887343331 | 4.1098537532475 |
| East Asia | Prevalence | Male | 2021 | 17.9774073428333 | -9.968488095 | 45.9233027807467 |
| East Asia | YLDs (Years Lived with Disability) | Female | 2021 | 1.85921305792851 | -0.95047782 | 4.66890393592754 |
| East Asia | Prevalence | Female | 2021 | 20.7286104000999 | -10.16410424 | 51.6213250428607 |
| East Asia | YLDs (Years Lived with Disability) | Both | 2021 | 1.73734294091949 | -0.958401547 | 4.43308742890053 |
| East Asia | Prevalence | Both | 2021 | 19.3764989752076 | -10.51405099 | 49.2670489367058 |
| Andean Latin America | YLDs (Years Lived with Disability) | Male | 1990 | 0.205338157012449 | -0.356908518 | 0.767584832217459 |
| Andean Latin America | Prevalence | Male | 1990 | 2.30403171940352 | -4.070474992 | 8.67853843124277 |
| Andean Latin America | YLDs (Years Lived with Disability) | Female | 1990 | 0.04525901240665 | -0.561921678 | 0.652439703026123 |
| Andean Latin America | Prevalence | Female | 1990 | 0.484908212424551 | -6.341996684 | 7.31181310872377 |
| Andean Latin America | YLDs (Years Lived with Disability) | Both | 1990 | 0.124781522374412 | -0.459863395 | 0.709426439981362 |
| Andean Latin America | Prevalence | Both | 1990 | 1.3885865835093 | -5.211187266 | 7.98836043252065 |
| Andean Latin America | YLDs (Years Lived with Disability) | Male | 2021 | 1.208447004 | 0.71879855448338 | 1.69809545269062 |
| Andean Latin America | Prevalence | Male | 2021 | 13.5945358558541 | 8.12202651006361 | 19.0670452016446 |
| Andean Latin America | YLDs (Years Lived with Disability) | Female | 2021 | 1.15999000028408 | 0.769231038427972 | 1.55074896214019 |
| Andean Latin America | Prevalence | Female | 2021 | 13.1142547110971 | 8.50160564601388 | 17.7269037761803 |
| Andean Latin America | YLDs (Years Lived with Disability) | Both | 2021 | 1.18365245035843 | 0.744874197449615 | 1.62243070326724 |
| Andean Latin America | Prevalence | Both | 2021 | 13.348021221369 | 8.32099842936881 | 18.3750440133691 |
| Central Europe | YLDs (Years Lived with Disability) | Male | 1990 | 0.114254120287703 | -0.030316771 | 0.2588250115534 |
| Central Europe | Prevalence | Male | 1990 | 1.25632177989454 | -0.340628426 | 2.85327198542612 |
| Central Europe | YLDs (Years Lived with Disability) | Female | 1990 | 0.358158587119517 | 0.00433067171036594 | 0.711986502528668 |
| Central Europe | Prevalence | Female | 1990 | 4.01486062318838 | -0.04995634 | 8.07967758611103 |
| Central Europe | YLDs (Years Lived with Disability) | Both | 1990 | 0.23950020604625 | -0.025671589 | 0.50467200131574 |
| Central Europe | Prevalence | Both | 1990 | 2.67607001089192 | -0.280096235 | 5.63223625702965 |
| Central Europe | YLDs (Years Lived with Disability) | Male | 2021 | 1.03829586645174 | 0.360919413351902 | 1.71567231955159 |
| Central Europe | Prevalence | Male | 2021 | 11.4970118392046 | 3.97513210760892 | 19.0188915708003 |
| Central Europe | YLDs (Years Lived with Disability) | Female | 2021 | 1.89074036453877 | 0.941226204652069 | 2.84025452442546 |
| Central Europe | Prevalence | Female | 2021 | 21.0620108259306 | 10.4620652895002 | 31.661956362361 |
| Central Europe | YLDs (Years Lived with Disability) | Both | 2021 | 1.48139187047857 | 0.579632941700412 | 2.38315079925673 |
| Central Europe | Prevalence | Both | 2021 | 16.5008046236867 | 6.39203279447104 | 26.6095764529023 |
| Eastern Europe | YLDs (Years Lived with Disability) | Male | 1990 | 0.0396126174290737 | -0.010821058 | 0.0900462930777917 |
| Eastern Europe | Prevalence | Male | 1990 | 0.440006639663465 | -0.142365967 | 1.02237924584895 |
| Eastern Europe | YLDs (Years Lived with Disability) | Female | 1990 | 0.0952521048316394 | 0.0237694196464384 | 0.16673479001684 |
| Eastern Europe | Prevalence | Female | 1990 | 1.05659610044451 | 0.264072594898259 | 1.84911960599077 |
| Eastern Europe | YLDs (Years Lived with Disability) | Both | 1990 | 0.0735565208033001 | -0.006724369 | 0.153837411092656 |
| Eastern Europe | Prevalence | Both | 1990 | 0.784778218860605 | -0.319471596 | 1.8890280339658 |
| Eastern Europe | YLDs (Years Lived with Disability) | Male | 2021 | 0.434536738877383 | 0.187451425631096 | 0.68162205212367 |
| Eastern Europe | Prevalence | Male | 2021 | 4.83097874016817 | 2.0358886510012 | 7.62606882933515 |
| Eastern Europe | YLDs (Years Lived with Disability) | Female | 2021 | 1.0876014614293 | 0.589076890592997 | 1.5861260322656 |
| Eastern Europe | Prevalence | Female | 2021 | 12.2819811444186 | 7.82664316447699 | 16.7373191243602 |
| Eastern Europe | YLDs (Years Lived with Disability) | Both | 2021 | 0.778416789471522 | 0.506480864183245 | 1.0503527147598 |
| Eastern Europe | Prevalence | Both | 2021 | 8.74260042471514 | 5.00524054144295 | 12.4799603079873 |
| Southern Latin America | YLDs (Years Lived with Disability) | Male | 1990 | -0.14375283 | -0.152809823 | -0.134695837 |
| Southern Latin America | Prevalence | Male | 1990 | -1.575468816 | -1.761537353 | -1.389400278 |
| Southern Latin America | YLDs (Years Lived with Disability) | Female | 1990 | -0.206239361 | -0.681155553 | 0.268676830874048 |
| Southern Latin America | Prevalence | Female | 1990 | -2.304194668 | -7.582599584 | 2.97421024865955 |
| Southern Latin America | YLDs (Years Lived with Disability) | Both | 1990 | -0.17635379 | -0.415815488 | 0.0631079086253759 |
| Southern Latin America | Prevalence | Both | 1990 | -1.955381029 | -4.575176396 | 0.664414338179977 |
| Southern Latin America | YLDs (Years Lived with Disability) | Male | 2021 | 0.431536596035897 | -0.785641973 | 1.64871516468505 |
| Southern Latin America | Prevalence | Male | 2021 | 4.82752700437849 | -8.674105748 | 18.3291597564713 |
| Southern Latin America | YLDs (Years Lived with Disability) | Female | 2021 | -0.047139786 | -1.214902802 | 1.12062323025739 |
| Southern Latin America | Prevalence | Female | 2021 | -0.521076178 | -13.64500032 | 12.6028479687743 |
| Southern Latin America | YLDs (Years Lived with Disability) | Both | 2021 | 0.182069602356962 | -1.01550028 | 1.37963948496397 |
| Southern Latin America | Prevalence | Both | 2021 | 2.03923994840391 | -11.33319896 | 15.4116788543679 |
| Western Europe | YLDs (Years Lived with Disability) | Male | 1990 | 0.21668991277774 | -0.061739337 | 0.495119162715476 |
| Western Europe | Prevalence | Male | 1990 | 2.38908692760799 | -0.713727861 | 5.49190171655812 |
| Western Europe | YLDs (Years Lived with Disability) | Female | 1990 | 0.947484400623884 | 0.129778305573566 | 1.7651904956742 |
| Western Europe | Prevalence | Female | 1990 | 10.6268658465702 | 1.61223938619333 | 19.6414923069471 |
| Western Europe | YLDs (Years Lived with Disability) | Both | 1990 | 0.662769481456125 | 0.180931023848899 | 1.14460793906335 |
| Western Europe | Prevalence | Both | 1990 | 7.38363788556172 | 2.0070059242241 | 12.7602698468993 |
| Western Europe | YLDs (Years Lived with Disability) | Male | 2021 | 0.644706065622512 | -0.524378509 | 1.81379064048794 |
| Western Europe | Prevalence | Male | 2021 | 7.00848603764632 | -6.627934419 | 20.64490649 |
| Western Europe | YLDs (Years Lived with Disability) | Female | 2021 | 0.356943178399328 | -1.609461835 | 2.32334819135782 |
| Western Europe | Prevalence | Female | 2021 | 4.07985893762642 | -18.07798228 | 26.2377001592805 |
| Western Europe | YLDs (Years Lived with Disability) | Both | 2021 | 0.51294372753798 | -0.84494508 | 1.87083253521365 |
| Western Europe | Prevalence | Both | 2021 | 5.61336068205025 | -9.515128444 | 20.7418498081542 |
| High-income North America | YLDs (Years Lived with Disability) | Male | 1990 | 0.569912223032074 | 0.343106185036941 | 0.796718261027206 |
| High-income North America | Prevalence | Male | 1990 | 6.29325598273013 | 3.73065918974995 | 8.85585277571031 |
| High-income North America | YLDs (Years Lived with Disability) | Female | 1990 | 1.11799586091471 | 0.93409173036508 | 1.30189999146434 |
| High-income North America | Prevalence | Female | 1990 | 12.4008103952781 | 10.292723059726 | 14.5088977308301 |
| High-income North America | YLDs (Years Lived with Disability) | Both | 1990 | 0.850293367081473 | 0.638482513638288 | 1.06210422052466 |
| High-income North America | Prevalence | Both | 1990 | 9.41774434833683 | 7.01007534791816 | 11.8254133487555 |
| High-income North America | YLDs (Years Lived with Disability) | Male | 2021 | 2.48436608273293 | 1.95250853949542 | 3.01622362597045 |
| High-income North America | Prevalence | Male | 2021 | 27.4726569809839 | 21.8986349308495 | 33.0466790311183 |
| High-income North America | YLDs (Years Lived with Disability) | Female | 2021 | 3.95818519033337 | 2.73538090925656 | 5.18098947141018 |
| High-income North America | Prevalence | Female | 2021 | 43.96912689 | 30.6986230208493 | 57.2396307514306 |
| High-income North America | YLDs (Years Lived with Disability) | Both | 2021 | 3.23222950145961 | 2.35341883321646 | 4.11104016970275 |
| High-income North America | Prevalence | Both | 2021 | 35.8423184339817 | 26.4022196862934 | 45.28241718 |
| High-income Asia Pacific | YLDs (Years Lived with Disability) | Male | 1990 | 0.661870158146342 | 0.574324892384685 | 0.749415423907999 |
| High-income Asia Pacific | Prevalence | Male | 1990 | 7.24545930354014 | 6.26732622436497 | 8.22359238271531 |
| High-income Asia Pacific | YLDs (Years Lived with Disability) | Female | 1990 | 0.640167788815212 | 0.576312700333214 | 0.704022877297211 |
| High-income Asia Pacific | Prevalence | Female | 1990 | 7.03721524106534 | 6.34133352279899 | 7.73309695933169 |
| High-income Asia Pacific | YLDs (Years Lived with Disability) | Both | 1990 | 0.653150154122924 | 0.622720325932388 | 0.683579982313459 |
| High-income Asia Pacific | Prevalence | Both | 1990 | 7.16528530388185 | 6.82468951134208 | 7.50588109642162 |
| High-income Asia Pacific | YLDs (Years Lived with Disability) | Male | 2021 | 1.93620359208687 | 0.502861746889683 | 3.36954543728406 |
| High-income Asia Pacific | Prevalence | Male | 2021 | 21.4865740458959 | 5.50226145984996 | 37.4708866319418 |
| High-income Asia Pacific | YLDs (Years Lived with Disability) | Female | 2021 | 2.59966876758977 | 0.533522795892607 | 4.66581473928694 |
| High-income Asia Pacific | Prevalence | Female | 2021 | 28.9529594298171 | 5.68224587257657 | 52.2236729870576 |
| High-income Asia Pacific | YLDs (Years Lived with Disability) | Both | 2021 | 2.26881046168455 | 0.520072819891719 | 4.01754810347739 |
| High-income Asia Pacific | Prevalence | Both | 2021 | 25.2292919935475 | 5.6161020318314 | 44.8424819552636 |
| Sub-Saharan Africa | YLDs (Years Lived with Disability) | Male | 1990 | 0.012872131715706 | -0.045538666 | 0.071282929462826 |
| Sub-Saharan Africa | Prevalence | Male | 1990 | 0.139288629331064 | -0.524663966 | 0.803241225051425 |
| Sub-Saharan Africa | YLDs (Years Lived with Disability) | Female | 1990 | 0.107841342132405 | 0.0347265651858434 | 0.180956119078967 |
| Sub-Saharan Africa | Prevalence | Female | 1990 | 1.19456205978314 | 0.365969475785425 | 2.02315464378085 |
| Sub-Saharan Africa | YLDs (Years Lived with Disability) | Both | 1990 | 0.058228410570399 | -0.002249928 | 0.118706749122352 |
| Sub-Saharan Africa | Prevalence | Both | 1990 | 0.641781746736974 | -0.044115787 | 1.32767928094782 |
| Sub-Saharan Africa | YLDs (Years Lived with Disability) | Male | 2021 | 0.0549789164526105 | -0.008775439 | 0.118733271569705 |
| Sub-Saharan Africa | Prevalence | Male | 2021 | 0.611138031679791 | -0.106112749 | 1.32838881187878 |
| Sub-Saharan Africa | YLDs (Years Lived with Disability) | Female | 2021 | 0.21422307355376 | 0.129686932674664 | 0.298759214432857 |
| Sub-Saharan Africa | Prevalence | Female | 2021 | 2.37643419301772 | 1.42757774782932 | 3.32529063820611 |
| Sub-Saharan Africa | YLDs (Years Lived with Disability) | Both | 2021 | 0.129337460222839 | 0.061047470620072 | 0.197627449825606 |
| Sub-Saharan Africa | Prevalence | Both | 2021 | 1.43544435955602 | 0.643723787413689 | 2.22716493169834 |
| Southeast Asia, east Asia, and Oceania | YLDs (Years Lived with Disability) | Male | 1990 | 0.0669321911202304 | 0.0328037514730706 | 0.10106063076739 |
| Southeast Asia, east Asia, and Oceania | Prevalence | Male | 1990 | 0.752020760768736 | 0.364908476615029 | 1.13913304492244 |
| Southeast Asia, east Asia, and Oceania | YLDs (Years Lived with Disability) | Female | 1990 | 0.14251572262423 | 0.0781288300782089 | 0.206902615170251 |
| Southeast Asia, east Asia, and Oceania | Prevalence | Female | 1990 | 1.60054428769182 | 0.866585211601911 | 2.33450336378172 |
| Southeast Asia, east Asia, and Oceania | YLDs (Years Lived with Disability) | Both | 1990 | 0.105488595891329 | 0.0734641797336954 | 0.137513012048963 |
| Southeast Asia, east Asia, and Oceania | Prevalence | Both | 1990 | 1.17754144640258 | 0.793805840934723 | 1.56127705187044 |
| Southeast Asia, east Asia, and Oceania | YLDs (Years Lived with Disability) | Male | 2021 | 0.349821960252465 | 0.232979310318274 | 0.466664610186656 |
| Southeast Asia, east Asia, and Oceania | Prevalence | Male | 2021 | 3.91677199555537 | 2.65629354200662 | 5.17725044910412 |
| Southeast Asia, east Asia, and Oceania | YLDs (Years Lived with Disability) | Female | 2021 | 0.594420992774792 | 0.319461312465763 | 0.86938067308382 |
| Southeast Asia, east Asia, and Oceania | Prevalence | Female | 2021 | 6.68660028380241 | 3.79840136324543 | 9.57479920435939 |
| Southeast Asia, east Asia, and Oceania | YLDs (Years Lived with Disability) | Both | 2021 | 0.467787705714607 | 0.287030361546875 | 0.648545049882339 |
| Southeast Asia, east Asia, and Oceania | Prevalence | Both | 2021 | 5.25689900966605 | 3.20282770466265 | 7.31097031466945 |
| Latin America and Caribbean | YLDs (Years Lived with Disability) | Male | 1990 | 0.157796019740644 | 0.0214739310102719 | 0.294118108471015 |
| Latin America and Caribbean | Prevalence | Male | 1990 | 1.73933717934044 | 0.140691605849437 | 3.33798275283145 |
| Latin America and Caribbean | YLDs (Years Lived with Disability) | Female | 1990 | 0.248827818584194 | 0.110625974478956 | 0.387029662689433 |
| Latin America and Caribbean | Prevalence | Female | 1990 | 2.76413477489902 | 1.22411916067773 | 4.30415038912031 |
| Latin America and Caribbean | YLDs (Years Lived with Disability) | Both | 1990 | 0.212431255145288 | 0.0686970934760495 | 0.356165416814526 |
| Latin America and Caribbean | Prevalence | Both | 1990 | 2.37128021039937 | 0.74863344932884 | 3.99392697146991 |
| Latin America and Caribbean | YLDs (Years Lived with Disability) | Male | 2021 | 0.538189520581416 | 0.19993921293418 | 0.876439828228652 |
| Latin America and Caribbean | Prevalence | Male | 2021 | 6.00009814675039 | 2.17730767829381 | 9.82288861520697 |
| Latin America and Caribbean | YLDs (Years Lived with Disability) | Female | 2021 | 0.719351602826978 | 0.353739145929893 | 1.08496405972406 |
| Latin America and Caribbean | Prevalence | Female | 2021 | 8.03622248448019 | 3.90556313229802 | 12.1668818366624 |
| Latin America and Caribbean | YLDs (Years Lived with Disability) | Both | 2021 | 0.636693689318733 | 0.280853969006216 | 0.992533409631251 |
| Latin America and Caribbean | Prevalence | Both | 2021 | 7.10820248432883 | 3.07651976815851 | 11.1398852004991 |
| Central Europe, eastern Europe, and central Asia | YLDs (Years Lived with Disability) | Male | 1990 | 0.194968122394222 | 0.101577832649902 | 0.288358412138541 |
| Central Europe, eastern Europe, and central Asia | Prevalence | Male | 1990 | 2.15737071863743 | 1.12637894915808 | 3.18836248811677 |
| Central Europe, eastern Europe, and central Asia | YLDs (Years Lived with Disability) | Female | 1990 | 0.536850091624874 | 0.411918729198507 | 0.661781454051241 |
| Central Europe, eastern Europe, and central Asia | Prevalence | Female | 1990 | 5.99600001523116 | 4.57509720427158 | 7.41690282619073 |
| Central Europe, eastern Europe, and central Asia | YLDs (Years Lived with Disability) | Both | 1990 | 0.36787919808006 | 0.260511312619615 | 0.475247083540505 |
| Central Europe, eastern Europe, and central Asia | Prevalence | Both | 1990 | 4.10556002481974 | 2.93427814767109 | 5.27684190196839 |
| Central Europe, eastern Europe, and central Asia | YLDs (Years Lived with Disability) | Male | 2021 | 0.957652883128164 | 0.682859699630665 | 1.23244606662566 |
| Central Europe, eastern Europe, and central Asia | Prevalence | Male | 2021 | 10.6296350697876 | 7.60209247070022 | 13.657177668875 |
| Central Europe, eastern Europe, and central Asia | YLDs (Years Lived with Disability) | Female | 2021 | 1.87566898582689 | 1.5542610200512 | 2.19707695160257 |
| Central Europe, eastern Europe, and central Asia | Prevalence | Female | 2021 | 20.9160763132287 | 17.1673373420351 | 24.6648152844223 |
| Central Europe, eastern Europe, and central Asia | YLDs (Years Lived with Disability) | Both | 2021 | 1.44182412157788 | 1.17347297384492 | 1.71017526931084 |
| Central Europe, eastern Europe, and central Asia | Prevalence | Both | 2021 | 16.0525010410759 | 13.0932176743849 | 19.011784407767 |
| High income | YLDs (Years Lived with Disability) | Male | 1990 | 0.341980731399735 | 0.0550306507349298 | 0.628930812064541 |
| High income | Prevalence | Male | 1990 | 3.77011718364447 | 0.617740956443883 | 6.92249341084507 |
| High income | YLDs (Years Lived with Disability) | Female | 1990 | 0.864142769666636 | 0.278348699919232 | 1.44993683941404 |
| High income | Prevalence | Female | 1990 | 9.60178136635603 | 3.07611647607429 | 16.1274462566378 |
| High income | YLDs (Years Lived with Disability) | Both | 1990 | 0.609996439178278 | 0.196060315299988 | 1.02393256305657 |
| High income | Prevalence | Both | 1990 | 6.76869456210415 | 2.14321235258159 | 11.3941767716267 |
| High income | YLDs (Years Lived with Disability) | Male | 2021 | 0.910543400734079 | 0.026714262846232 | 1.79437253862193 |
| High income | Prevalence | Male | 2021 | 9.97456253993092 | 0.222618501825453 | 19.7265065780364 |
| High income | YLDs (Years Lived with Disability) | Female | 2021 | 0.856039132461124 | -0.583003152 | 2.29508141724156 |
| High income | Prevalence | Female | 2021 | 9.48744789877438 | -6.08184111 | 25.0567369070997 |
| High income | YLDs (Years Lived with Disability) | Both | 2021 | 0.859574153618213 | -0.221285192 | 1.94043349880252 |
| High income | Prevalence | Both | 2021 | 9.4828028745983 | -2.568156028 | 21.5337617774965 |

**TableS6 Decomposition Analysis of Changes in the Burden of Heart Failure Attributable to Atrial Fibrillation and Flutter, 1990–2021: Contributions of Aging, Population Growth, and Epidemiological Changes by Socio-Demographic Index (SDI) Level, Sex, and Measure (Prevalence and Years Lived with Disability [YLDs])**

| **Location** | **Sex** | **Measure** | **Overll difference** | **Aging** | **Population** | **Epidemiological change** |
| --- | --- | --- | --- | --- | --- | --- |
| Global | Male | YLDs (Years Lived with Disability) | 20399.46 | 4988.12 (24.45%) | 8292.54 (40.65%) | 7118.79 (34.9%) |
| Global | Female | YLDs (Years Lived with Disability) | 28928.09 | 5858.37 (20.25%) | 12965.97 (44.82%) | 10103.76 (34.93%) |
| Global | Both | YLDs (Years Lived with Disability) | 49327.55 | 10962.88 (22.22%) | 21223.87 (43.03%) | 17140.8 (34.75%) |
| Global | Male | Prevalence | 227567.29 | 56172.78 (24.68%) | 92268.81 (40.55%) | 79125.69 (34.77%) |
| Global | Female | Prevalence | 324008.85 | 66306.9 (20.46%) | 144832.08 (44.7%) | 112869.86 (34.84%) |
| Global | Both | Prevalence | 551576.14 | 123765.13 (22.44%) | 236708.02 (42.91%) | 191102.98 (34.65%) |
| High-middle SDI | Male | YLDs (Years Lived with Disability) | 3522.14 | 979.04 (27.8%) | 1182.19 (33.56%) | 1360.92 (38.64%) |
| High-middle SDI | Female | YLDs (Years Lived with Disability) | 6130.44 | 1490.66 (24.32%) | 2196.75 (35.83%) | 2443.02 (39.85%) |
| High-middle SDI | Both | YLDs (Years Lived with Disability) | 9652.58 | 2507.63 (25.98%) | 3398.8 (35.21%) | 3746.15 (38.81%) |
| High-middle SDI | Male | Prevalence | 39166.01 | 10964.95 (28%) | 13102.38 (33.45%) | 15098.67 (38.55%) |
| High-middle SDI | Female | Prevalence | 68682.57 | 16875.61 (24.57%) | 24526.57 (35.71%) | 27280.39 (39.72%) |
| High-middle SDI | Both | Prevalence | 107848.58 | 28265.64 (26.21%) | 37852.68 (35.1%) | 41730.26 (38.69%) |
| High SDI | Male | YLDs (Years Lived with Disability) | 10182.15 | 3057.7 (30.03%) | 2743.99 (26.95%) | 4380.46 (43.02%) |
| High SDI | Female | YLDs (Years Lived with Disability) | 13413.7 | 3476.19 (25.92%) | 3768.28 (28.09%) | 6169.23 (45.99%) |
| High SDI | Both | YLDs (Years Lived with Disability) | 23595.85 | 6481.57 (27.47%) | 6599.53 (27.97%) | 10514.74 (44.56%) |
| High SDI | Male | Prevalence | 113208.07 | 34189.62 (30.2%) | 30416.69 (26.87%) | 48601.76 (42.93%) |
| High SDI | Female | Prevalence | 149518.58 | 39097.36 (26.15%) | 41935.8 (28.05%) | 68485.42 (45.8%) |
| High SDI | Both | Prevalence | 262726.65 | 72687.99 (27.67%) | 73331.13 (27.91%) | 116707.53 (44.42%) |
| Low-middle SDI | Male | YLDs (Years Lived with Disability) | 1732.12 | 259.13 (14.96%) | 945.37 (54.58%) | 527.61 (30.46%) |
| Low-middle SDI | Female | YLDs (Years Lived with Disability) | 1941.54 | 387.23 (19.94%) | 1041.26 (53.63%) | 513.05 (26.43%) |
| Low-middle SDI | Both | YLDs (Years Lived with Disability) | 3673.65 | 660.67 (17.98%) | 1986.37 (54.07%) | 1026.61 (27.95%) |
| Low-middle SDI | Male | Prevalence | 19546.09 | 2968.18 (15.19%) | 10649.51 (54.48%) | 5928.4 (30.33%) |
| Low-middle SDI | Female | Prevalence | 21908.58 | 4431.78 (20.23%) | 11717.33 (53.48%) | 5759.47 (26.29%) |
| Low-middle SDI | Both | Prevalence | 41454.67 | 7566.27 (18.25%) | 22364.89 (53.95%) | 11523.51 (27.8%) |
| Low SDI | Male | YLDs (Years Lived with Disability) | 602.48 | 12.1 (2.01%) | 425.12 (70.56%) | 165.25 (27.43%) |
| Low SDI | Female | YLDs (Years Lived with Disability) | 586.85 | 25.16 (4.29%) | 447.38 (76.23%) | 114.31 (19.48%) |
| Low SDI | Both | YLDs (Years Lived with Disability) | 1189.33 | 37.64 (3.17%) | 872.3 (73.34%) | 279.39 (23.49%) |
| Low SDI | Male | Prevalence | 6794.05 | 159.96 (2.35%) | 4770.17 (70.21%) | 1863.93 (27.43%) |
| Low SDI | Female | Prevalence | 6604.23 | 301.04 (4.56%) | 5023.52 (76.07%) | 1279.68 (19.38%) |
| Low SDI | Both | Prevalence | 13398.29 | 465.53 (3.47%) | 9791.24 (73.08%) | 3141.52 (23.45%) |
| Middle SDI | Male | YLDs (Years Lived with Disability) | 4342.92 | 1326.02 (30.53%) | 1983.11 (45.66%) | 1033.8 (23.8%) |
| Middle SDI | Female | YLDs (Years Lived with Disability) | 6829.22 | 1851.38 (27.11%) | 3115.27 (45.62%) | 1862.58 (27.27%) |

# TableS7 Projected Global and China Burden of Heart Failure Attributable to Atrial Fibrillation and Flutter, 2022–2040

| **Year** | **Location** | **Prevalence Number(N)** | **Prevalence ASR**  **(per 100 000)** | **YLDs Number(N)** | **YLDs ASR (per 100 000)** |
| --- | --- | --- | --- | --- | --- |
| 2022 | China | 133232.69 (118113.82 to 148351.57) | 6.94 (6.15 to 7.73) | 11842.04 (10598.58 to 13088.96) | 0.62 (0.55 to 0.68) |
| 2022 | Global | 723936.27 (694659.7 to 753212.84) | 8.85 (8.49 to 9.21) | 64727.4 (61273.86 to 68180.94) | 0.79 (0.75 to 0.83) |
| 2023 | China | 141884.18 (124898.87 to 158869.49) | 7.07 (6.22 to 7.91) | 12546.69 (11091.56 to 14005.26) | 0.63 (0.55 to 0.7) |
| 2023 | Global | 743594.06 (708037.3 to 779150.81) | 8.82 (8.4 to 9.24) | 66477.07 (62421.37 to 70532.78) | 0.79 (0.74 to 0.84) |
| 2024 | China | 150827.74 (131331.1 to 170324.38) | 7.2 (6.27 to 8.14) | 13269.35 (11495.53 to 15046.57) | 0.64 (0.55 to 0.72) |
| 2024 | Global | 766390.2 (721116.1 to 811664.3) | 8.79 (8.27 to 9.31) | 68509.85 (63543.23 to 73476.47) | 0.78 (0.73 to 0.84) |
| 2025 | China | 160364.36 (137551.88 to 183176.84) | 7.35 (6.3 to 8.4) | 14037.76 (11828.6 to 16250.29) | 0.65 (0.54 to 0.75) |
| 2025 | Global | 790038.59 (731911.28 to 848165.91) | 8.76 (8.11 to 9.4) | 70624.64 (64456.9 to 76792.38) | 0.78 (0.71 to 0.85) |
| 2026 | China | 170550.57 (143505.32 to 197595.83) | 7.51 (6.32 to 8.71) | 14856.19 (12089.62 to 17626.19) | 0.66 (0.53 to 0.78) |
| 2026 | Global | 815239.74 (741266.96 to 889212.52) | 8.73 (7.93 to 9.52) | 72871.49 (65219.41 to 80523.56) | 0.78 (0.7 to 0.86) |
| 2027 | China | 182067.54 (149664.76 to 214470.31) | 7.69 (6.32 to 9.06) | 15780.28 (12316.64 to 19247.47) | 0.67 (0.52 to 0.82) |
| 2027 | Global | 842893.85 (750047.56 to 935740.13) | 8.7 (7.74 to 9.66) | 75319.87 (65895.73 to 84744.02) | 0.78 (0.68 to 0.87) |
| 2028 | China | 195223.68 (156161.04 to 234286.33) | 7.87 (6.29 to 9.45) | 16835.35 (12515.52 to 21158.88) | 0.68 (0.51 to 0.86) |
| 2028 | Global | 872148.43 (757529.98 to 986766.88) | 8.68 (7.54 to 9.82) | 77909.28 (66435.51 to 89383.04) | 0.77 (0.66 to 0.89) |
| 2029 | China | 208716.21 (161827.78 to 255604.63) | 8.07 (6.25 to 9.88) | 17916.56 (12592.39 to 23244.61) | 0.7 (0.49 to 0.9) |
| 2029 | Global | 901419.94 (762327.49 to 1040512.39) | 8.66 (7.32 to 9.99) | 80511.59 (66727.8 to 94295.38) | 0.77 (0.64 to 0.9) |
| 2030 | China | 222819.81 (166797.47 to 278842.16) | 8.28 (6.19 to 10.36) | 19052.23 (12556.76 to 25551.77) | 0.71 (0.47 to 0.96) |
| 2030 | Global | 930920.58 (764596.17 to 1097244.99) | 8.63 (7.09 to 10.17) | 83148.59 (66787.84 to 99509.35) | 0.77 (0.62 to 0.92) |
| 2031 | China | 237789.72 (171148.9 to 304430.54) | 8.5 (6.12 to 10.88) | 20264.31 (12406.31 to 28126.57) | 0.73 (0.45 to 1.01) |
| 2031 | Global | 961819.68 (765176.14 to 1158463.23) | 8.61 (6.85 to 10.37) | 85912.19 (66678.01 to 105146.38) | 0.77 (0.6 to 0.94) |
| 2032 | China | 254875.55 (175606.52 to 334144.58) | 8.74 (6.02 to 11.45) | 21654.46 (12176.25 to 31137.13) | 0.75 (0.42 to 1.07) |
| 2032 | Global | 995829.72 (765169.89 to 1226489.56) | 8.59 (6.6 to 10.58) | 88939.06 (66481.7 to 111396.41) | 0.77 (0.57 to 0.96) |
| 2033 | China | 275060.15 (180555.65 to 369564.65) | 8.98 (5.89 to 12.07) | 23304.68 (11867.69 to 34746.32) | 0.77 (0.39 to 1.14) |
| 2033 | Global | 1032366.16 (763861.71 to 1300870.6) | 8.57 (6.34 to 10.8) | 92199.89 (66151.6 to 118248.18) | 0.77 (0.55 to 0.98) |
| 2034 | China | 296180.78 (184263.21 to 408098.36) | 9.25 (5.75 to 12.74) | 25046.65 (11345.68 to 38752.52) | 0.79 (0.36 to 1.22) |
| 2034 | Global | 1069012.43 (759234.53 to 1378790.32) | 8.55 (6.07 to 11.02) | 95494.59 (65520.24 to 125468.94) | 0.76 (0.52 to 1) |
| 2035 | China | 317913.46 (186347.49 to 449479.43) | 9.52 (5.58 to 13.46) | 26864.08 (10569.42 to 43164.35) | 0.81 (0.32 to 1.3) |
| 2035 | Global | 1105434.48 (750929.51 to 1459939.46) | 8.52 (5.79 to 11.26) | 98793.02 (64552.46 to 133033.59) | 0.76 (0.5 to 1.03) |
| 2036 | China | 340365.26 (186686.47 to 494044.05) | 9.81 (5.37 to 14.24) | 28765.56 (9503.04 to 48035.38) | 0.83 (0.27 to 1.39) |
| 2036 | Global | 1142591.71 (739438.47 to 1545744.95) | 8.5 (5.5 to 11.5) | 102163.53 (63275.19 to 141051.88) | 0.76 (0.47 to 1.05) |
| 2037 | China | 364792.64 (185717.34 to 543868.25) | 10.11 (5.14 to 15.08) | 30853.33 (8129.69 to 53588.71) | 0.86 (0.22 to 1.49) |
| 2037 | Global | 1182582.67 (725795.59 to 1639369.76) | 8.49 (5.21 to 11.77) | 105769.55 (61759.49 to 149779.6) | 0.76 (0.44 to 1.08) |
| 2038 | China | 392496.14 (183681.94 to 601312.25) | 10.42 (4.87 to 15.98) | 33235.89 (6417.34 to 60097.11) | 0.89 (0.17 to 1.6) |
| 2038 | Global | 1224937.21 (709330.91 to 1740543.51) | 8.48 (4.91 to 12.04) | 109596.18 (59958.8 to 159233.56) | 0.76 (0.42 to 1.1) |
| 2039 | China | 420714.25 (178836.25 to 662596.21) | 10.75 (4.56 to 16.94) | 35702.66 (5176.67 to 67276.07) | 0.92 (0.13 to 1.73) |
| 2039 | Global | 1266606.48 (687976.97 to 1845235.99) | 8.46 (4.6 to 12.33) | 113394.83 (57703.79 to 169085.87) | 0.76 (0.39 to 1.13) |
| 2040 | China | 448929.45 (170744.16 to 727121.8) | 11.09 (4.2 to 17.98) | 38230.51 (3602.68 to 75152.43) | 0.95 (0.09 to 1.87) |
| 2040 | Global | 1307469.42 (661547.36 to 1953391.48) | 8.45 (4.28 to 12.63) | 117155.11 (54971.45 to 179338.78) | 0.76 (0.36 to 1.16) |

# FigureS1 Global Burden of Heart Failure Attributed to Atrial fibrillation and Atrial Flutter, 1990

**A** ASR of Prevalence in 1990; **B** ASR of YLDs (Years Lived with Disability) in 1990;


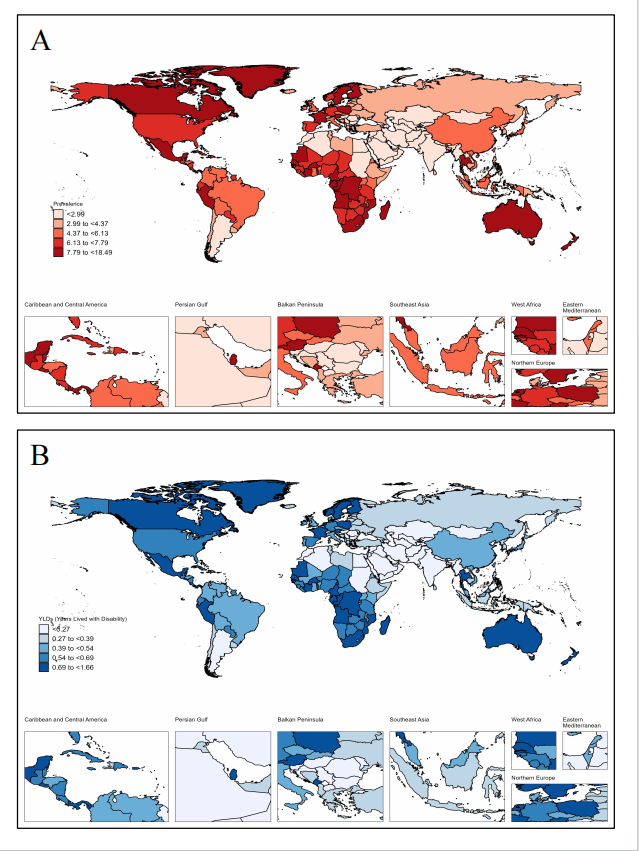

Supplement: xvag094_Supplementary_Data [file xvag094_supplementary_data.docx]
